# Rhodanine and 2,4-dioxothiazolidine-based amides: synthesis and evaluation as dual-action cholinesterase inhibitors and antioxidants

**DOI:** 10.1039/d6md00502k

**Published:** 2026-08-18

**Authors:** Enikő Šikorová, Adéla Kyslíková, Markéta Švarcová, Jiří Palarčík, Šárka Štěpánková, Martin Krátký

**Affiliations:** a Department of Organic and Bioorganic Chemistry, Faculty of Pharmacy in Hradec Králové, Charles University Akademika Heyrovského 1203 500 03 Hradec Králové Czech Republic martin.kratky@faf.cuni.cz; b Institute of Environmental and Chemical Engineering, Faculty of Chemical Technology, University of Pardubice Studentská 573 532 10 Pardubice Czech Republic; c Department of Biological and Biochemical Sciences, Faculty of Chemical Technology, University of Pardubice Studentská 573 532 10 Pardubice Czech Republic sarka.stepankova@upce.cz

## Abstract

This study presents the synthesis and biological evaluation of substituted 2-(2,4-dioxothiazolidin-3-yl)-*N*-phenylacetamides and their thioxo analogues as potential multitarget agents for neurodegenerative diseases. A diverse library of amides was designed to explore structure–activity relationships and synthesized using two complementary approaches: a carbodiimide-mediated coupling and a microwave-assisted procedure. The compounds were screened for acetylcholinesterase (AChE) and butyrylcholinesterase (BChE) inhibition and antioxidant activity. Several derivatives displayed micromolar dual inhibition, with 2-(4-oxo-2-thioxothiazolidin-3-yl)-*N*-(3,4,5-trichlorophenyl)acetamide (compound 1h) emerging as the most effective BChE inhibitor (IC_50_ = 2.91 μM), which is also one of the most potent AChE inhibitors in this series. It acts as a specific, reversible, and competitive inhibitor of both BChE and AChE. Polyhalogenated analogues consistently outperformed their mono- and di-substituted counterparts, highlighting the critical role of lipophilic electron-withdrawing substituents. Within this series, rhodanines were generally preferred over thiazolidine-2,4-diones. Our results have demonstrated that rational modifications of the rhodanine and thiazolidine-2,4-dione cores support further investigation of selected derivatives as multitarget cholinesterase inhibitors with complementary antioxidant properties.

## Abstract 2

This study presents the synthesis and biological evaluation of substituted 2-(2,4-dioxothiazolidin-3-yl)-*N*-phenylacetamides and their thioxo analogues as potential multitarget agents for neurodegenerative diseases.

## Graphical abstract

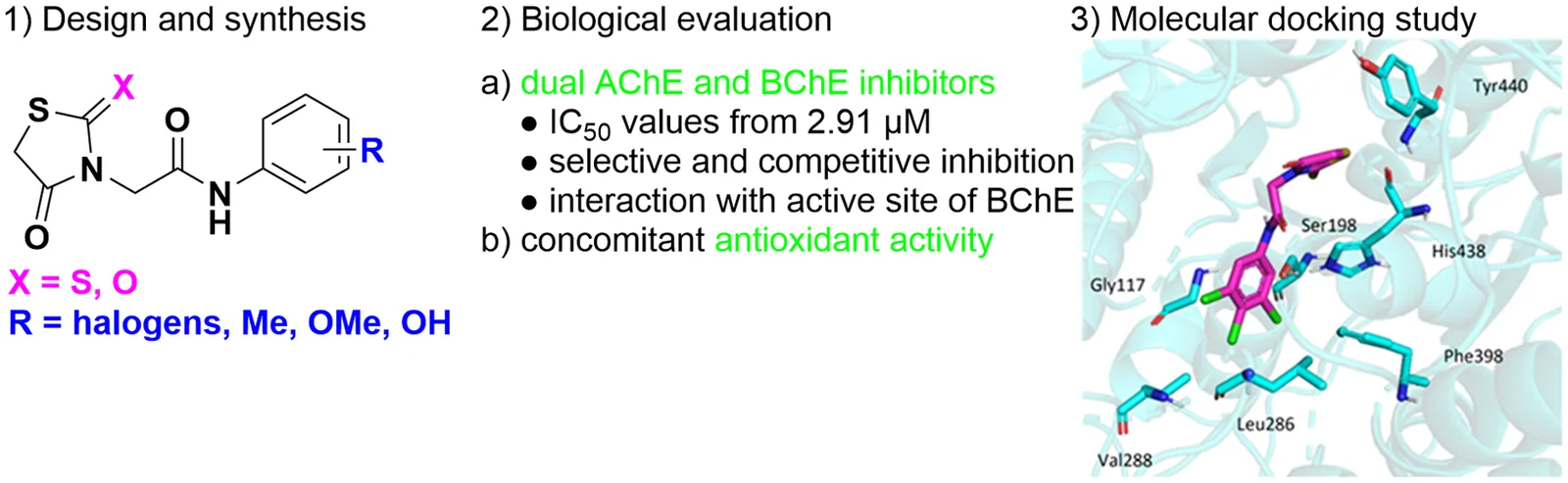

## Introduction

Rhodanine (2-thioxothiazolidin-4-one) is a five-membered heterocyclic compound containing a thiazolidine core substituted with a carbonyl and a thiocarbonyl group. Recognized as a “privileged” scaffold, its significance in medicinal chemistry and drug development arises from its versatility, particularly its ability to undergo diverse chemical modifications, mainly at the *N*-3 and *C*-5 positions, enabling the design of novel bioactive compounds.^1–4^ Notably, the rhodanine scaffold has a high potential for forming hydrogen bonds and polar interactions with potential targets, making it attractive for multitarget drug design, particularly for diseases with complex pathogenesis.^2^

Since the 1960s, numerous studies have highlighted the diverse biological activities of rhodanine derivatives, with a primary focus on their broad-spectrum antimicrobial properties and role in diabetes regulation, alongside their anticancer, antioxidant and anti-inflammatory effects.^1,2,4–7^

Initially, rhodanine derivatives featuring a *C*-5-exocyclic double bond attracted significant interest as promising drug candidates.^6^ However, concerns arose regarding their classification as pan-assay interference compounds (PAINS), primarily due to issues such as spectrophotometric colour interference, aggregation, chelation, and potential non-specific reactivity of the exocyclic double bond as a Michael acceptor.^1,5,6,8^ While more recent findings suggest that these concerns may not be as universal or severe as initially thought—demonstrating specific inhibitory effects of 5-ylidene rhodanines without covalent binding and negligible thiol reactivity in some instances—the controversy surrounding their reliability in drug discovery remains.^8,9^

Thiazolidine-2,4-dione is a structurally related oxo isostere of rhodanine.^10^ Its derivatives also exhibit a broad spectrum of biological activities, *e.g.*, antidiabetic, anti-inflammatory, antioxidant, and antitumor effects, with potential applications in treating obesity and as antagonists of thyroid hormone receptors.^10–14^ Furthermore, its analogues, pioglitazone and rosiglitazone, are currently used in clinical practice to treat diabetes mellitus.^10,13^ Recently, 5-arylidene-thiazolidine-2,4-dione derivatives have been reported as potent dual acetylcholinesterase (AChE) and butyrylcholinesterase (BChE) inhibitors with adjunctive antioxidant activity^15^ as well as neuroprotective effects.^16^

Cholinesterase inhibitors play a crucial role in the treatment of various pathologies, including neurodegenerative disorders, myasthenia gravis, glaucoma, and gastrointestinal and urinary conditions, as well as serving as antidotes.^17,18^ Among these, their application in Alzheimer's disease (AD) is particularly significant. AD is the most common form of dementia. More than 55 million people worldwide live with dementia, and it is one of the leading causes of death globally. Moreover, according to WHO projections, the number of individuals suffering from AD is expected to rise to 139 million by 2050.^19^ AD is a complex disorder characterized primarily by extensive neuronal loss, accompanied by multiple pathophysiological processes. These include acetylcholine neurotransmission deficiencies, amyloid-β plaque accumulation, tau protein pathologies, neuroinflammation, and oxidative stress.^20^

Since reactive oxygen species (ROS) and other free radicals contribute significantly to the neurotoxicity in AD,^21,22^ developing compounds with dual action, acting both as antioxidants and cholinesterase inhibitors, represents a promising therapeutic strategy for clinical use. The multitarget-directed ligand (MTDL) drug design approach offers distinct advantages in addressing the multifactorial nature of AD.^23^ By targeting multiple pathways simultaneously, MTDLs have the potential to enhance therapeutic efficacy synergistically, reduce the progression of disease symptoms, and minimize side effects compared to single-target drugs.^23,24^ This innovative strategy holds significant promise for improving neuroprotection and expanding clinical treatment options for AD.

As part of our ongoing research on novel AChE and BChE inhibitors, we designed and synthesized a new series of rhodanine derivatives, building on our previous work on rhodanine-based compounds.^3,7,25^ The present study aimed at a systematic structure–activity relationship (SAR) exploration of less explored *C*-5-unsubstituted *N*-3-acetamide rhodanine and thiazolidinedione derivatives, which avoid the exocyclic double bond associated with PAINS-related concerns of frequently investigated *C*-5-exocyclic substituted rhodanines. Building on our expertise in the rational design of novel cholinesterase inhibitors, we selected *N*-(4-chlorophenyl)-2-(4-oxo-2-thioxothiazolidin-3-yl)acetamide (1a; Fig. 1), previously identified by our group as a potent dual AChE/BChE inhibitor,^3,7^ as a lead structure. The present library was designed to systematically assess how the position and extent of halogen substitution, other aromatic substitution patterns, and C=S/C=O isosteric replacement affect AChE/BChE inhibition and antioxidant activity within a closely related series. Structurally, the present series is characterized by a fixed *N*-3-(CH_2_–CONH–Ar) connectivity, while the *C*-5 position remains unsubstituted. This approach enabled direct comparison of structurally matched analogues and the identification of previously unavailable SAR within this chemotype. Fig. 1 depicts the design of these targeted derivatives. Subsequently, these newly synthesized analogues were evaluated for their potential as enzyme inhibitors and antioxidants.

**Fig. 1:**
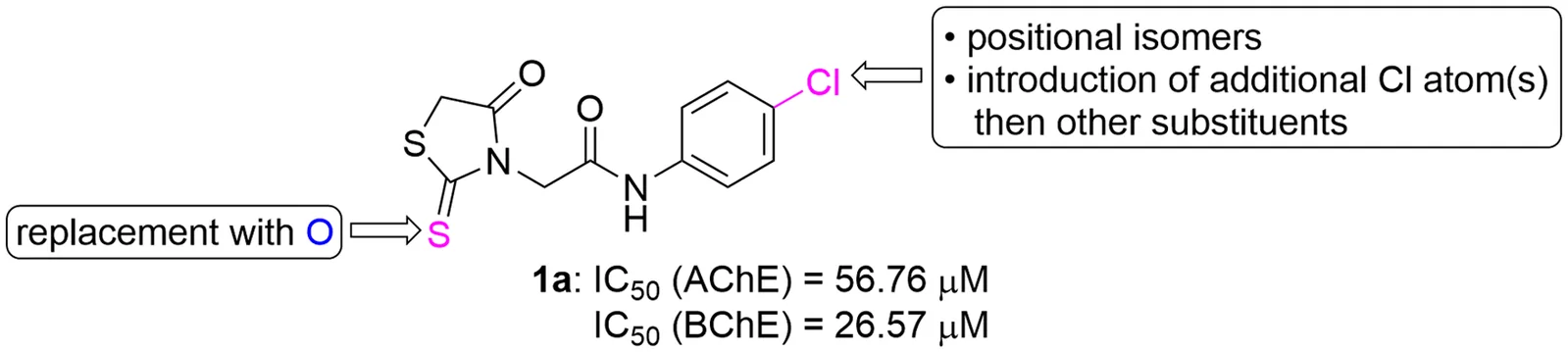
Structure and activity of parent hit 1a and design of its novel/structurally matched analogues.

## Experimental

### Chemistry

#### General

All chemicals were obtained from Sigma-Aldrich/Merck KGaA (Darmstadt, Germany), Penta Chemicals Unlimited (Prague, Czech Republic), and Lach-Ner (Neratovice, Czech Republic) and were used without further purification, except for hexane, which was purified by distillation. The progress of reactions and product purity were monitored using thin-layer chromatography (TLC). TLC was performed on silica gel 60 F254 plates with a layer thickness of 0.2 mm (Merck, Darmstadt, Germany) and visualized under UV light (A. Krüss Optronic, Hamburg, Germany) at 254 and 366 nm. The eluents used were dichloromethane/methanol (93 : 7, v/v).

Melting points were determined using a Büchi B-560 apparatus (BÜCHI Labortechnik AG, Flawil, Switzerland) and are reported without correction. The structures of the rhodanine derivatives were confirmed by ^1^H and ^13^C NMR spectroscopy. NMR spectra were recorded in dimethyl sulfoxide (DMSO-*d*_6_) and acetone-*d*_6_ at room temperature using either a JNM-ECZ 600R instrument (600 MHz for ^1^H and 151 MHz for ^13^C; JEOL, Tokyo, Japan) or a Varian VNMR S500 instrument (Varian, Palo Alto, CA, USA). Chemical shifts (*δ*; ppm) are referenced indirectly to tetramethylsilane *via* the residual solvent signals (DMSO: *δ* = 2.50 for ^1^H, *δ* = 39.51 for ^13^C; acetone: *δ* = 29.8 and 206.3 ppm for ^13^C). Coupling constants (*J*) are reported in Hz. The NMR spectra were analyzed using the MestReNova software (Mestrelab Research, Santiago de Compostela, Spain).

Infrared spectra were recorded using a Nicolet 6700 FT-IR spectrometer (Thermo Fisher Scientific, Waltham, MA, USA) within the range of 650–4000 cm^−1^, employing the attenuated total reflectance (ATR) technique (Ge crystal). Elemental analysis was performed on a Vario MICRO Cube Elemental Analyzer (Elementar Analysensysteme, Hanau, Germany), with calculated and found values expressed as percentages. Yields are presented as the ratio of isolated yield to theoretical yield. The experimental data are provided in SI.

All chemical structures and reaction schemes were created using ChemDraw Ultra 20.0 (CambridgeSoft, Cambridge, MA, USA).

### Synthesis of amides 1 and 2

#### Method A^3^

One equivalent of 2-(4-oxo-2-(thio/oxo)thiazolidin-3-yl)acetic acid (1 mmol) was combined with 1.1 equivalents of 1-hydroxybenzotriazole hydrate (HOBt; 153.6 mg) and one equivalent of the corresponding aniline in a 1 : 1 mixture of ethyl acetate and dichloromethane (10 mL). Once the components dissolved completely, *N*-(3-dimethylaminopropyl)-*N*′-ethylcarbodiimide hydrochloride (EDC; 1.2 equivalents, 233.8 mg) was added in a single portion. The reaction mixture was stirred at room temperature for at least 8 hours, with progress monitored by TLC.

After completion, the solvent was evaporated to dryness, and the residue was suspended in ethyl acetate. The resulting suspension was sequentially washed twice with 0.1 M aqueous HCl, 5% aqueous sodium bicarbonate, and brine. The organic layer was dried over anhydrous sodium sulfate, filtered, and concentrated under reduced pressure to induce crystallization. To promote precipitation, *n*-hexane was added, and the mixture was kept at +4 °C for 24 hours. The precipitates were filtered off, yielding the desired amides 1 and 2. If necessary, the products were recrystallized from ethyl acetate.

#### Method B

One equivalent of 2-(4-oxo-2-(thio/oxo)thiazolidin-3-yl)acetic acid (1 mmol) was reacted in the presence of 3 equivalents of phosphorus trichloride (3 mmol; 262 μL) in chlorobenzene (15 mL) under microwave irradiation for 30 min. After the reaction was completed, 1.1 equivalents of the appropriate aniline (1.1 mmol) were added in one portion, and the reaction mixture was stirred again under microwave irradiation (530 W, 600 rpm, open reaction vessels, MicroSYNTH, Milestone) until the reaction was completed (*ca.* 30 min). No fixed temperature program was applied; reactions were conducted under fixed-power microwave irradiation. Then, chlorobenzene was evaporated under reduced pressure. The residue was dissolved or suspended in ethyl acetate and washed twice with 0.1 M aqueous HCl, 5% aqueous sodium bicarbonate, followed by brine. The organic layer was dried over anhydrous sodium sulfate and filtered. The filtrate was concentrated under reduced pressure to initiate crystallization. Hexane was added to promote the precipitation, and the precipitates were filtered off to afford the amides.

### Inhibition of acetylcholinesterase and butyrylcholinesterase

The ability of investigated derivatives to inhibit AChE from *Electrophorus electricus* L. (eeAChE) and BChE from equine serum (eqBChE) was screened spectrophotometrically using a modified Ellman's method.^25^ The inhibitory activity of the investigated compounds was expressed as IC_50_ (*i.e.*, 50% inhibitory concentration). The selectivity index (SI), a ratio of IC_50_ value for AChE/IC_50_ for BChE, was also calculated. Rivastigmine and donepezil, drugs used to alleviate symptoms of AD, were used as standards. All the enzymes, substrates, and drugs were purchased from Merck (Darmstadt, Germany).

Further experiments were then carried out with the most effective BChE inhibitor, 1h.

It was verified whether preincubation of the inhibitor with the enzyme affects the reaction rate. The procedure was similar to the IC_50_ determination, with the only difference being that the enzyme was preincubated with compound 1h for 5 min before the reaction rate was determined. For both enzymes, 1h concentration was chosen corresponding to the obtained IC_50_ value (*i.e.*, 30 μM for AChE and 3 μM for BChE). The reaction rate where the enzyme was not preincubated with the inhibitor was compared with that where the preincubation was performed.

#### Reversibility of inhibition

We further verified the reversibility of the inhibition of 1h when inhibiting both AChE and BChE. Three different concentrations of inhibitor were chosen depending on IC_50_ values. The aim was to observe the effect of the inhibitor on enzyme activity over time. The procedure was described in detail previously.^25^ The information about the reversibility or irreversibility of the process is provided by the dependence of the percentage of residual enzyme activity on time.

#### Type of inhibition

The kinetic studies of AChE and BChE inhibition caused by 1h were performed using a modified Ellman's method. The procedure was similar to that for the determination of IC_50_. The concentration of the substrate (acetylthiocholine, ATCh/butyrylthiocholine, BTCh) in the final reaction mixture was 20, 40, 60, and 80 μM; the concentration of DTNB was 0.1 mM, and the enzyme activity was 0.2 U mL^−1^. Four different inhibitor concentrations were chosen for each substrate concentration (according to the IC_50_ values). From the obtained absorbance (*A*; 412 nm) *versus* time (*t*), uninhibited and inhibited reaction rates were calculated. Then, a Lineweaver–Burk plot was constructed, and the values of *K*_m_ and *V*_m_ were calculated. Based on that, the type of inhibition was identified.

#### Non-specific interactions

In the previous study,^25^ we verified, among other things, the possibility of non-specific interactions of rhodanines with AChE. In this study, we extended it to include BChE. A series of experiments using Tween 20 as a non-ionic surfactant was performed. The effect of Tween 20 on BChE activity was evaluated at final Tween 20 concentrations of 0.0001, 0.001, and 0.01%. To determine the possible inhibition of BChE by Tween 20, the reaction rates of BTCh hydrolysis catalyzed by BChE in the absence and presence of Tween 20 (concentration range of 0.1–5%) were determined under the same conditions as for screening the investigated inhibitors.

The effect of Tween 20 on the inhibitory activity of 1h was also evaluated. The final concentration of 1h in the reaction mixture was 30 μM (for inhibition of AChE) and 3 μM (for inhibition of BChE), and various concentrations of Tween 20 were used (0, 0.0001, 0.001, 0.01, 0.1, and 1%). Reaction rates in the presence of surfactant were determined following a similar procedure to the IC_50_ determination.

### Particle size measurement and colour interference verification

The particle size was measured to verify the possible clustering of the components of the reaction mixtures. First, the particle size measurement was performed for the reaction mixture containing 0.1 M phosphate buffer (pH 7.4), AChE or BChE (the enzyme activity in the total reaction mixture was 0.2 U mL^−1^), and 1h (the concentration of inhibitor in the total reaction mixture was 30 μM for AChE and 3 μM for BChE). Then, the particle size was measured in the reaction mixtures additionally containing Tween 20 (concentrations of Tween 20 in the final reaction mixture were 0.01% and 0.001%, respectively). Average particle size was measured by dynamic light scattering using Zetasizer Nano ZS (Malvern Panalytical Ltd., Malvern, United Kingdom). Measurements were performed at 25 °C, with a scattering angle of 173°, using disposable sizing cuvettes. Each measurement was performed in ten replicates.

The possible colour interference of 1h was verified simultaneously with the inhibition assays. The measurement of absorbance changes over time to determine the reaction rate was performed on the Hewlett-Packard 8453 diode array spectrophotometer. At the same time, the entire absorption spectrum was recorded at each time point. We were primarily interested in possible colour interference at a wavelength of 412 nm (corresponding to the absorption maximum of 5-sulfanyl-2-nitrobenzoic acid ion). The concentration of 1h in the final reaction mixture was 30 μM for AChE and 3 μM for BChE.

### Total antioxidant capacity

Total antioxidant capacity (TAC) was determined using a commercially available kit, the Antioxidant Assay kit (Catalogue Number MAK334, Sigma-Aldrich). This kit is suitable for determining antioxidants in various biological samples (*e.g.*, serum, plasma, urine), food, and beverages. It can also be utilized in drug discovery or pharmacology to study the effect of drugs on TAC.

The Antioxidant Assay kit measures TAC in which an antioxidant reduces a Cu^2+^ ion to a Cu^+^ ion, forming a coloured complex with a dye reagent. The colour intensity measured at 570 nm is proportional to TAC in the sample. The kit has a linear detection range from 1.5 to 1000 μM Trolox equivalents.

Trolox standards and reaction mix were prepared according to the instructions for the Antioxidant Assay kit.^26^ Trolox standards were used in concentrations of 0, 300, 600, and 1000 μM. The concentrations of investigated derivatives were chosen based on the determined IC_50_ values for cholinesterase inhibition. A concentration equal to the IC_50_ value and 10 times higher for a specific derivative was always used. These concentrations were selected to assess antioxidant capacity at exposure levels related to the BChE-inhibitory activity of each compound rather than to compare their intrinsic antioxidant potency. Investigated derivatives and Trolox were diluted with ultrapure water (conductivity 0.057 μS cm^−1^; Barnstead MicroPure Water Purification System, Thermo Scientific).

Trolox standards, the studied derivatives, and blanks were always assayed in duplicate in separate wells of a transparent, flat-bottom 96-well plate. Each well contained 20 μL of suitably diluted Trolox or tested derivative or ultrapure water and 100 μL of the reaction mixture. After 10 min incubation at room temperature, the absorbance was measured at 570 nm on a BioTek Synergy H1 Multimode Reader (Agilent). The dependence of absorbance *A*_570_ on the concentration of Trolox standard was plotted, and the slope of the standard curve was determined. The total antioxidant capacity of investigated derivatives was calculated as follows:
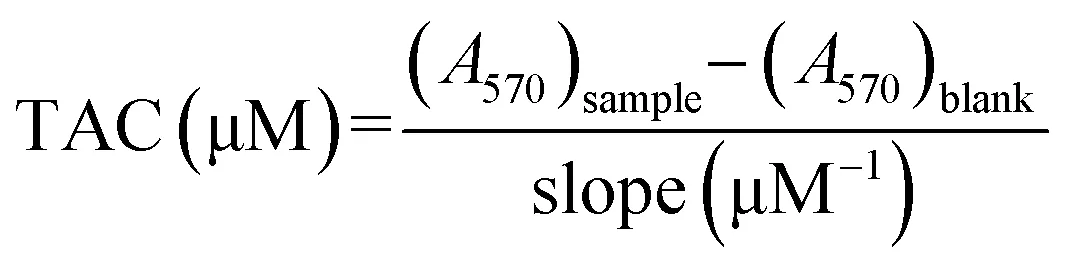


### Molecular docking

Crystallographic structures of human AChE and BChE were retrieved from the Protein Data Bank (https://www.rcsb.org; PDB codes 4PQE and 1P0I). The 3D structures of the ligands were prepared using Chem3D Pro 19.1 (ChemBioOffice 2019 Package, CambridgeSoft, Cambridge, MA, USA). During the preparation process, all water molecules were removed from the enzyme structures, and both enzymes and ligands were optimized using the UCSF Chimera software package (Amber force field).^27^ Docking studies were conducted with AutoDock Vina^28^ and AutoDock 4.2 (ref. 29) utilizing the Lamarckian genetic algorithm. The 3D affinity grid box was configured to encompass complete active and peripheral sites of AChE and BChE. The grid dimensions for AutoDock Vina were set to 20 × 20 × 20 grid points in the *x*-, *y*- and *z*-axes with a spacing of 1 Å, while for AutoDock 4, the dimensions were 40 × 40 × 40 grid points with a spacing of 0.4 Å. Graphic visualizations of the ligand–enzyme interactions were generated using PyMOL software (The PyMOL Molecular Graphics System, Version 1.5, Schrödinger, LLC, New York, USA).

## Results and discussion

### Design and synthesis

Twenty-nine *N*-phenylamides of rhodanine (1) and thiazolidinediones (2) were designed based on the previously reported biological activity of *N*-(4-chlorophenyl)-2-(4-oxo-2-thioxothiazolidin-3-yl)acetamide 1a, which demonstrated antimycobacterial activity^7^ as well as dual inhibition of cholinesterases.^3^ Given its promising bioactivity, its chemical space was expanded by synthesizing positional isomers and analogues bearing additional chlorine substituents (1b–1j). Furthermore, we prepared oxo analogues of rhodanine, 2-(2,4-dioxothiazolidin-3-yl)-*N*-phenylacetamides (2a–2j), allowing direct evaluation of the C=S/C=O replacement in matched pairs.

Following the identification of 3,4,5-trichloroanilide 1h as a potent enzyme inhibitor, we further explored 3,4,5-trisubstituted derivatives, systematically replacing chlorine with fluorine, bromine, methyl, hydroxy, or methoxy groups (1k–2o) to investigate their impact on bioactivity. To the best of our knowledge, except for the hit 1a, compounds 1b–1o and 2a–2o have not been previously reported.

These amides were synthesized using two different synthetic protocols. One approach involved a carbodiimide-mediated coupling reaction between rhodanine-3-acetic acid or 2,4-dioxothiazolidine-3-acetic acid and an equimolar amount of the respective aniline, using a mild excess of HOBt and EDC, following a previously published protocol^3^ (Method A, Fig. 2). Due to solubility issues, the reaction was conducted in a dichloromethane/ethyl acetate mixture. While this approach is established for rhodanine-3-acetic acid derivatives, to the best of our knowledge, its application to the corresponding 2,4-dioxothiazolidine analogue has not been reported previously. Alternatively, a second method utilizing microwave irradiation (MW) was employed, in which the corresponding acid was first activated with phosphorus trichloride, followed by the addition of the appropriate aniline, yielding the desired amides (Method B, Fig. 2). This method was helpful, especially for polyhalogenated anilines with low nucleophilicity or sterically hindered anilines (generally, 2,6-substituted ones), for which Method A gave very low yields (<10%) or failed to provide the desired products. The use of PCl_3_ for this type of amide bond formation has previously been reported only for a single reaction performed in pyridine without MW. The final products were isolated and purified *via* extraction and crystallization, giving isolated yields of 36–69%. For rhodanine-3-acetic acid, the corresponding acid chloride can also be generated *in situ* using oxalyl chloride.^30^

**Fig. 2:**
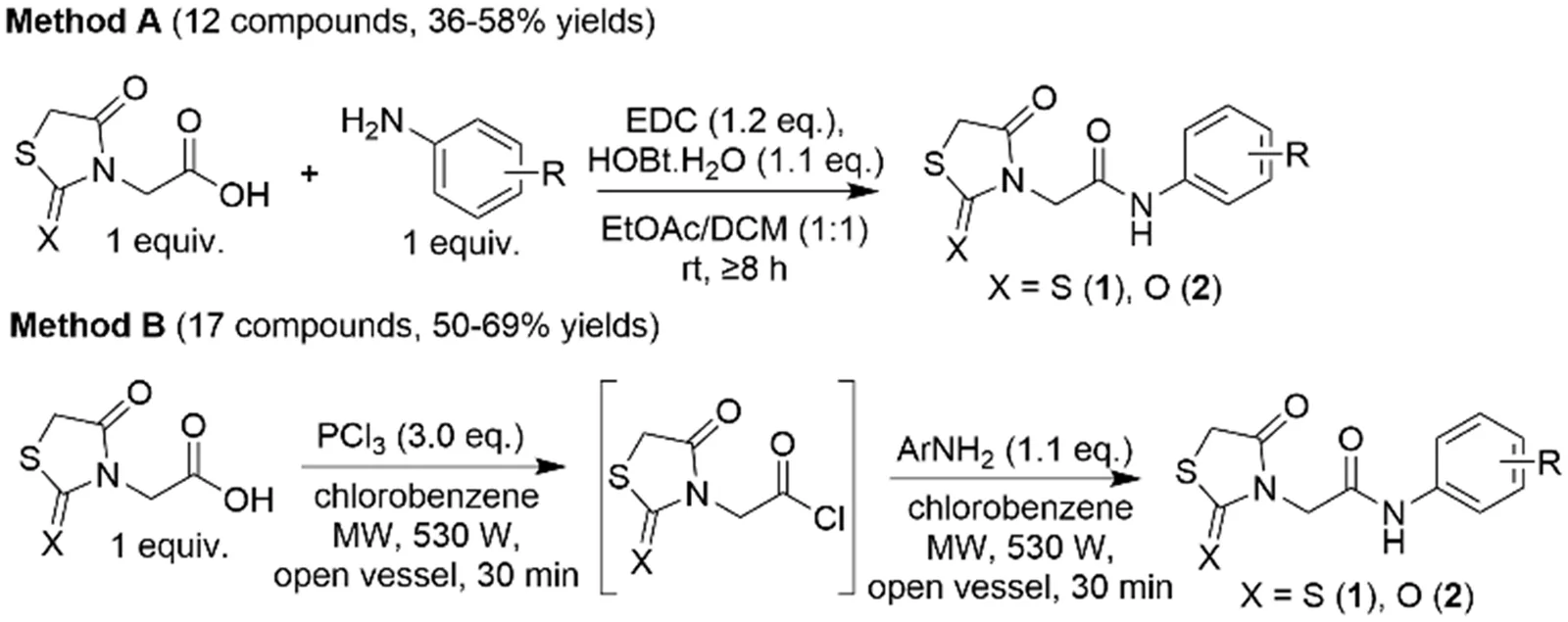
Synthesis of acetamides 1 and 2 [EDC: *N*-(3-dimethylaminopropyl)-*N*′-ethylcarbodiimide hydrochloride; HOBt: 1-hydroxybenzotriazole; DCM: dichloromethane; EtOAc: ethyl acetate; MW: microwave; R = H, Cl, F, Br, OH, CH_3_, OCH_3_].

### Inhibition of AChE and BChE

The inhibitory activity of the amides 1–2 against AChE and BChE was assessed spectrophotometrically using a modified Ellman's method,^31^ a standard approach for *in vitro* screening of potential cholinesterase inhibitors (Table 1). Enzymatic activity was measured indirectly by detecting the formation of 5-sulfanyl-2-nitrobenzoic acid, which results from the reaction of the thiol reagent 5,5′-dithiobis(2-nitrobenzoic acid) (DTNB) with thiocholine. Thiocholine is the hydrolytic product formed from the cleavage of ATCh and BTCh, catalysed by AChE and BChE, respectively. AChE was obtained from electric eel, while BChE was from equine serum.

**Table 1:** Inhibition of cholinesterases

Code	X	R	AChE	BChE	Selectivity
			IC_50_ (μM)	IC_50_ (μM)	(IC_50_ AChE/BChE)
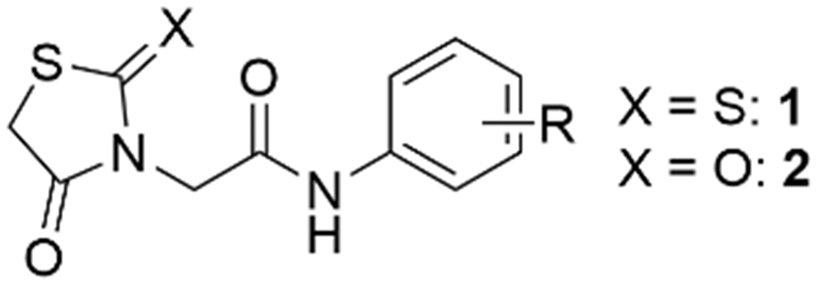					
1a^3^	S	4-Cl	56.76 ± 1.47	26.57 ± 0.29	2.1
2a	O	4-Cl	60.94 ± 0.55	56.56 ± 1.48	1.1
1b	S	3-Cl	146.63 ± 0.33	14.80 ± 0.70	**9.9**
2b	O	3-Cl	56.20 ± 1.06	21.83 ± 0.04	2.6
1c	S	2-Cl	93.67 ± 0.50	90.62 ± 3.14	1.0
2c	O	2-Cl	123.56 ± 5.84	57.80 ± 3.05	2.1
1d	S	3,4-Cl_2_	58.29 ± 2.05	**7.83 ± 0.14**	7.4
2d	O	3,4-Cl_2_	40.67 ± 1.31	12.69 ± 0.12	3.2
1e	S	3,5-Cl_2_	59.51 ± 2.32	**4.70 ± 0.08**	**12.7**
2e	O	3,5-Cl_2_	83.47 ± 2.87	**4.93 ± 0.21**	**16.9**
1f	S	2,6-Cl_2_	47.62 ± 1.19	69.87 ± 3.45	0.7
2f	O	2,6-Cl_2_	**25.86 ± 0.09**	**6.56 ± 0.73**	3.9
1g	S	2,4-Cl_2_	44.84 ± 1.75	108.72 ± 4.66	0.4
2g	O	2,4-Cl_2_	131.16 ± 7.63	>500	<0.3
1h	S	3,4,5-Cl_3_	**34.42 ± 0.01**	**2.91 ± 0.05**	**11.8**
2h	O	3,4,5-Cl_3_	172.98 ± 5.50	10.70 ± 0.63	**16.2**
1i	S	2,4,5-Cl_3_	**32.18 ± 0.65**	**9.35 ± 0.32**	3.4
2i	O	2,4,5-Cl_3_	53.53 ± 0.64	14.76 ± 0.43	3.6
1j	S	2,4,6-Cl_3_	53.86 ± 1.40	37.97 ± 1.69	1.4
2j	O	2,4,6-Cl_3_	43.01 ± 1.63	25.26 ± 1.09	1.7
1k	S	3,4,5-F_3_	55.03 ± 1.07	28.32 ± 0.08	1.9
2k	O	3,4,5-F_3_	45.22 ± 0.15	63.16 ± 2.64	0.7
1l	S	3,4,5-(OMe)_3_	75.14 ± 2.14	107.84 ± 4.69	0.7
2l	O	3,4,5-(OMe)_3_	58.16 ± 1.17	121.66 ± 1.04	0.5
1m	S	3,4,5-Me_3_	46.36 ± 2.86	16.91 ± 0.55	2.7
2m	O	3,4,5-Me_3_	72.67 ± 0.58	20.15 ± 0.35	3.6
1n	S	3,5-Cl_2_-4-OH	58.33 ± 0.20	40.76 ± 1.55	1.4
2n	O	3,5-Cl_2_-4-OH	63.07 ± 1.13	59.81 ± 2.18	1.1
1o	S	4-Br-3,5-F_2_	**36.40 ± 0.29**	**4.50 ± 0.06**	8.1
2o	O	4-Br-3,5-F_2_	52.80 ± 0.88	**8.64 ± 0.13**	6.1
**RIV**	—	—	56.10 ± 1.41	38.40 ± 1.97	1.5
**Donepezil**			0.015 ± 0.0001	0.031 ± 0.001	2.1

Inhibitory activities are presented as IC_50_ values, representing the concentrations required to achieve 50% inhibition of enzymatic activity. Additionally, we calculated SI to quantify the selectivity towards BChE, expressed as the ratio IC_50_ AChE/IC_50_ BChE. Rivastigmine, a well-known dual AChE and BChE inhibitor, and donepezil, a reversible inhibitor of AChE, were employed as reference standards for comparative purposes.

Except for 2g, all derivatives caused dual inhibition of both enzymes. When focusing on amides 1 and 2, BChE was inhibited more effectively with IC_50_ values ranging from 2.91 to >500 μM. The most potent inhibitors included 3,4,5-trichloroanilide of rhodanine-3-acetic acid 1h, followed closely by another trihalogenated anilide, 1o (4-Br-3,5-F_2_; IC_50_ = 4.50 μM), as well as both 3,5-dichloroanilides 1e and 2e (4.70 and 4.93 μM, respectively). Conversely, in addition to inactive anilide 2g, the weakest inhibition was observed for 2,4-dichloroanilides (1g, 2g) and 3,4,5-trimethoxyanilides 1l and 2l, which exhibited IC_50_ ≥ 107.84 μM. The inhibition of AChE by amides was observed at a higher concentration range of 25.86–172.98 μM, though the potency differences among derivatives were less pronounced than in the case of BChE. The most active AChE inhibitors were 2,6-dichloroanilide of thiazolidinedione-3-acetic acid 2f, followed by rhodanine-based trihalogenated anilides 1i (2,4,5-Cl_3_; IC_50_ = 32.18 μM), 1h (3,4,5-Cl_3_; IC_50_ = 34.42 μM), and 1o (4-Br-3,5-F_2_; IC_50_ = 36.40 μM). The group of weakest inhibitors included three thiazolidinediones and one rhodanine, specifically two monochlorinated derivatives (1b, 2c) and two polyhalogenated derivatives (2g, 2h), with IC_50_ values of 123.56–172.98 μM.

Replacing sulfur (derivatives 1) with oxygen (derivatives 2) in the thiazolidinone core generally led to lower inhibitory activity against BChE and, to a lesser extent and with less consistency, against AChE. This trend suggests that the sulfur atom enhances enzyme binding, potentially through increased hydrophobic interactions and electronic effects.

The presence, type, and position of halogen atoms on the phenyl ring significantly influenced inhibitory activity. Generally, multiple halogen substitutions, particularly trichlorination (1h, 1i), resulted in superior inhibition to mono- or di-chlorinated derivatives. Among monochlorinated amides, substitution at position 4 favoured the inhibition of AChE (1a), while substitution at position 3 was more effective for BChE inhibition (1b). Except for 2,4-dichlorinated congeners g, dichlorinated derivatives demonstrated improved inhibitory activity mainly against BChE. Moreover, potent inhibition of both enzymes was retained when chlorine was replaced with bromine or fluorine (1o and 2o). In contrast, replacing one chlorine with a hydroxyl group (1n and 2n) or substituting all 3,4,5-chlorines with fluorine, methyl, or especially methoxy groups (derivatives k, l, m, respectively) resulted in a significant decline in activity, especially in BChE inhibition. This pattern highlights the critical role of lipophilic electronegative substituents in enhancing enzyme binding.

To assess the selectivity of these inhibitors, we calculated selectivity indexes (SI), defined as the ratio IC_50_ for AChE/IC_50_ for BChE, indicating a significant preference for inhibiting AChE when SI is approximately 0.5 or lower. Three compounds inhibited AChE preferentially (1g, 2g, and 2l). In contrast, three compounds exhibited a balanced inhibition of both enzymes (2a, 1c, and 2n with SI ≈ 1), indicating dual inhibition potential. Several compounds (1b, 1e, 2e, 1h, and 2h; SI approximately 10 or higher) demonstrated strong selectivity for BChE over AChE, particularly those bearing multiple chlorines. For instance, rhodanine 1e (R = 3,5-Cl_2_) exhibited high SI (12.7), while its oxo analogue 2e had an even greater selectivity (SI = 16.9), *i.e.*, being the most selective compound in this study. The introduction of a third chlorine atom decreased selectivity modestly. In general, this suggests that these compounds might be theoretically beneficial in conditions where BChE inhibition is preferred, such as late-stage Alzheimer's disease. Additionally, dual inhibition of both cholinesterases can also be considered advantageous for the potential treatment of neurodegenerative diseases.^32^

Rivastigmine, a dual AChE and BChE inhibitor, showed IC_50_ values of 56.10 μM for AChE and 38.40 μM for BChE, with an SI of 1.5. Several derivatives showed substantially greater BChE inhibitory potency, including 1d, 1e, 2e, 2f, 1h, 1i, 1o, and 2o, which exhibited single-digit micromolar IC_50_ values. Most notably, compound 1h inhibited BChE with an IC_50_ of 2.91 μM, corresponding to an approximately 13.2-fold greater potency compared with rivastigmine under the same assay conditions. In contrast, improvements in AChE inhibition were more modest. Fourteen derivatives showed superior activity relative to rivastigmine, while others were comparable. The most active inhibitor, 2f (IC_50_ = 25.86 μM), showed an approximately 2.2-fold lower IC_50_ than rivastigmine, whereas 1h (IC_50_ = 34.42 μM) showed an approximately 1.6-fold difference. Thus, the improvement relative to rivastigmine was clearly more pronounced for BChE than for AChE.

Donepezil was markedly more potent under the same assay conditions (IC_50_ = 0.015 and 0.031 μM for AChE and BChE, respectively), with the best derivatives remaining approximately 1700-fold and 94-fold less potent. Thus, the present compounds should be regarded as micromolar cholinesterase inhibitors.

Having these results in hand, we conducted advanced experiments with *N*-trichlorophenyl amide 1h, one of the most potent dual inhibitors identified in this study. The first experiment aimed to determine whether this inhibitor binds to the enzymes reversibly or irreversibly.

The results revealed that 1h inhibits AChE and BChE through a reversible action. In reversible inhibition, the enzyme–inhibitor complex can dissociate, and enzyme activity is not progressively lost over time under the assay conditions. The dependence of enzyme activity on time clearly indicates this reversible binding mechanism for AChE and BChE. Fig. 3 illustrates the relationship between the logarithm of the percentage of residual enzyme activity and time for the inhibition of AChE and BChE using 1h at three different concentrations. This confirms the transient nature of the interaction between the cholinesterases and the inhibitor.

**Fig. 3:**
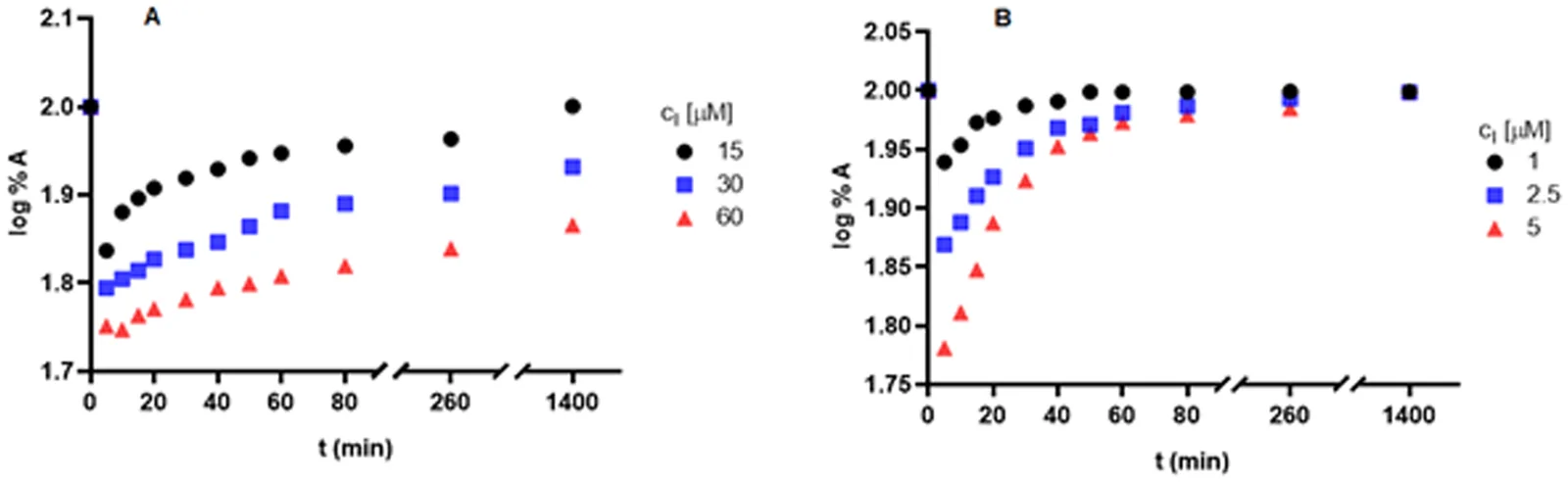
The dependence of the logarithm of the percentage of residual enzyme activity on time when using 1h inhibiting AChE (A) and BChE (B). The measurements were done in duplicate, and the points are expressed as mean values.

Furthermore, we determined the type of inhibition for both enzymes for the most effective BChE inhibitor (1h), which is also one of the highly active AChE inhibitors in this study. Enzyme inhibitors are classified as competitive, non-competitive, uncompetitive, or mixed. The type of inhibition can be identified using a Lineweaver–Burk plot^33^ by analysing two key kinetic parameters: the maximum velocity (*V*_m_) and the Michaelis constant (*K*_M_) of the inhibited and uninhibited reaction. The nature of these changes, along with the point of intersection of the lines in the Lineweaver–Burk plot, distinguishes the type of inhibition.

The type of inhibition was determined for the most potent derivative 1h using a Lineweaver–Burk plot. Since the lines in the Lineweaver–Burk plots intersect on the *y*-axis, and thus, *V*_m_ is identical for both the uninhibited and the inhibited reactions, it can be concluded that 1h acts as a competitive inhibitor of both AChE and BChE. The corresponding Lineweaver–Burk plots are presented in Fig. 4. Additionally, the inclusion of 0.001% Tween 20 in this assay did not affect the results, ensuring the reliability of the measurements.

**Fig. 4:**
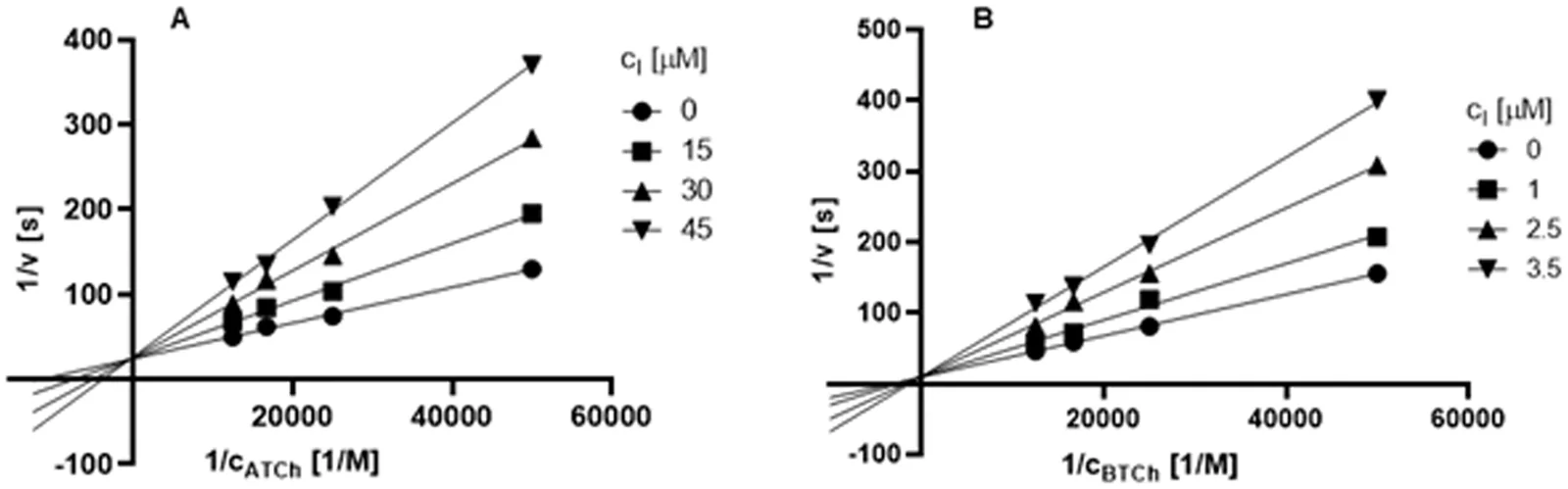
Lineweaver–Burk plot for 1h inhibiting eeAChE (A) and eqBChE (B). The measurements were done in duplicate, and the points are expressed as mean values. ATCh: acetylthiocholine, BTCh: butyrylthiocholine.

To ensure the reliability of the recorded data, we conducted a series of experiments to exclude potential non-specific interactions. Promiscuous inhibitors can be discriminated by the use of detergents and also a short compound preincubation test.^34^

First, we evaluated the effect of the surfactant Tween 20 on BChE activity. Similar to findings with AChE,^25^ it was confirmed that Tween 20, at concentrations ranging from 0.0001% to 1%, does not affect BChE activity (see SI, Table S1). Furthermore, Tween 20 does not inhibit BChE activity even at concentrations as high as 5% (Fig. S1).

We also investigated whether the presence of Tween 20 influenced the inhibition by 1h. The final reaction mixture contained Tween 20 at concentrations in the range 0.0001–1%, with 1h at 30 μM for AChE and 3 μM for BChE inhibition, respectively. A comparison of reaction rates with and without Tween 20 revealed that this surfactant does not affect the reaction rate. This observation suggests that aggregation between the inhibitor and the enzyme is unlikely. Moreover, this result aligns with the earlier findings, supporting the notion that competitive inhibitors, like 1h, are unlikely to act as non-specific aggregators.^35^ The determined values of the reaction rates observed for AChE and BChE inhibition by 1h in the absence and presence of Tween 20 are listed in Table S2 (SI).

We further verified whether preincubation of the enzyme with 1h affects the reaction rate. A comparison of reactions without preincubation and those with a 5-minute preincubation period showed no significant effect on the reaction rate (Table S3, SI).

Moreover, we measured particle sizes under various conditions to confirm that 1h is a true ChE inhibitor and that no clustering occurs in the reaction mixture. The reaction mixtures contained 0.1 M PBS (pH 7.4), AChE or BChE, 1h, and optionally Tween 20 at concentrations of 0.01% and 0.001%. The results demonstrated no evidence of aggregation, even in the absence of Tween 20.

Finally, we ensured there was no colour interference. Absorption spectra recorded during the reactions showed a single absorption maximum at 412 nm, corresponding to the ion of 5-sulfanyl-2-nitrobenzoic acid, for both AChE and BChE.

### Molecular docking

The active sites of both AChE and BChE are well-characterized.^36–39^ It is well established that the active site is located deep beneath the enzyme surface, near the bottom of a relatively narrow cavity. Hydrolysis is facilitated by the catalytic triad, consisting of residues Ser203, His447, and Glu334 in AChE and Ser198, His438, and Glu325 in BChE. Additionally, other regions of these enzymes play a crucial role. The peripheral anionic site (PAS), composed of residues Tyr72, Asp74, Tyr124, Trp286, and Tyr341, is also involved in ligand interactions.^39^ Ligand binding to PAS can influence AChE activity by inducing conformational changes in the active site.^40^

BChE, often described as non-specific, exhibits lower substrate selectivity. This is primarily attributed to differences in amino acid composition; some aromatic residues in AChE are replaced by smaller hydrophobic residues in BChE. Consequently, the enzyme cavity in BChE is more accessible to larger ligands and permits these ligands to adopt a broader range of conformations.^38,41^

The molecular modeling study was performed to rationalize *in vitro* results and SAR and to estimate possible binding modes of the prepared competitive inhibitors with both enzymes.

For AChE, the top-scored docking pose of compound 2f (the most potent inhibitor against AChE in the series) is depicted in Fig. 5. The molecule of the ligand is buried deep in the cavity, with the dichlorophenyl moiety heading towards the centre of the active-site gorge and the thiazolidine moiety heading to the entrance of the active gorge. The molecule is stabilized in its position by several interactions: namely, the dichlorophenyl moiety showed π-stacking with Trp86; the linker allows hydrogen bonds with Tyr124 and Tyr337, and the thiazolidine moiety with Ser125.

**Fig. 5:**
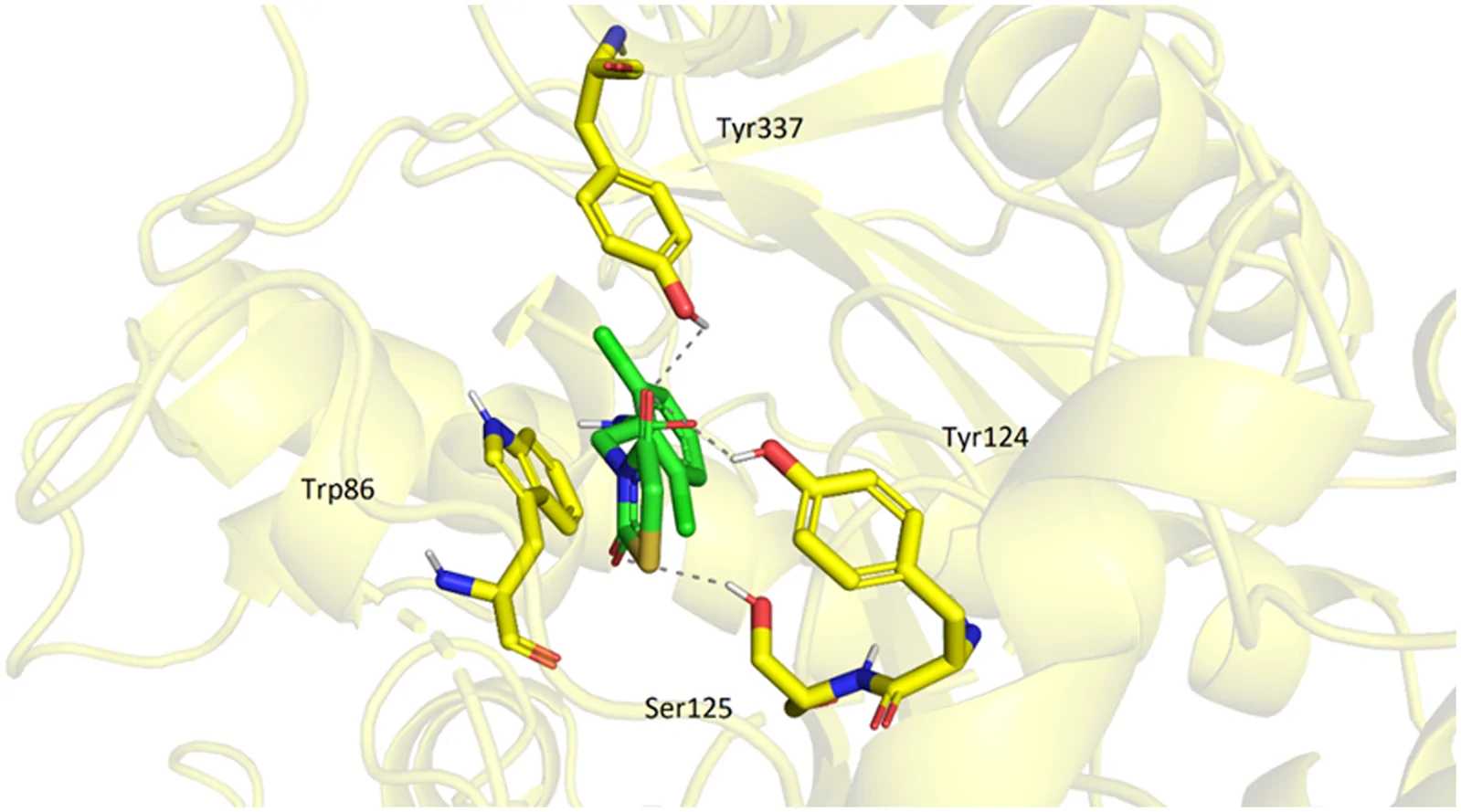
Conformation of compound 2f in the active site of AChE.

Differently from 2f, ligand 1h was localized near the entrance of the gorge, and its molecule was in an inverted position with the thiazolidine moiety heading towards the centre of the enzyme. Compound 1l showed a similar orientation in the enzyme but displayed lower binding affinity (binding energy of −8.1 kcal mol^−1^*vs.* −7.1 kcal mol^−1^ for 1h and 1l, respectively). The linkers of both molecules allowed interactions with Tyr337 and Tyr124. The substituted aryl moiety showed π-stacking with two aromatic residues (Tyr341 and Trp286, Fig. 6).

**Fig. 6:**
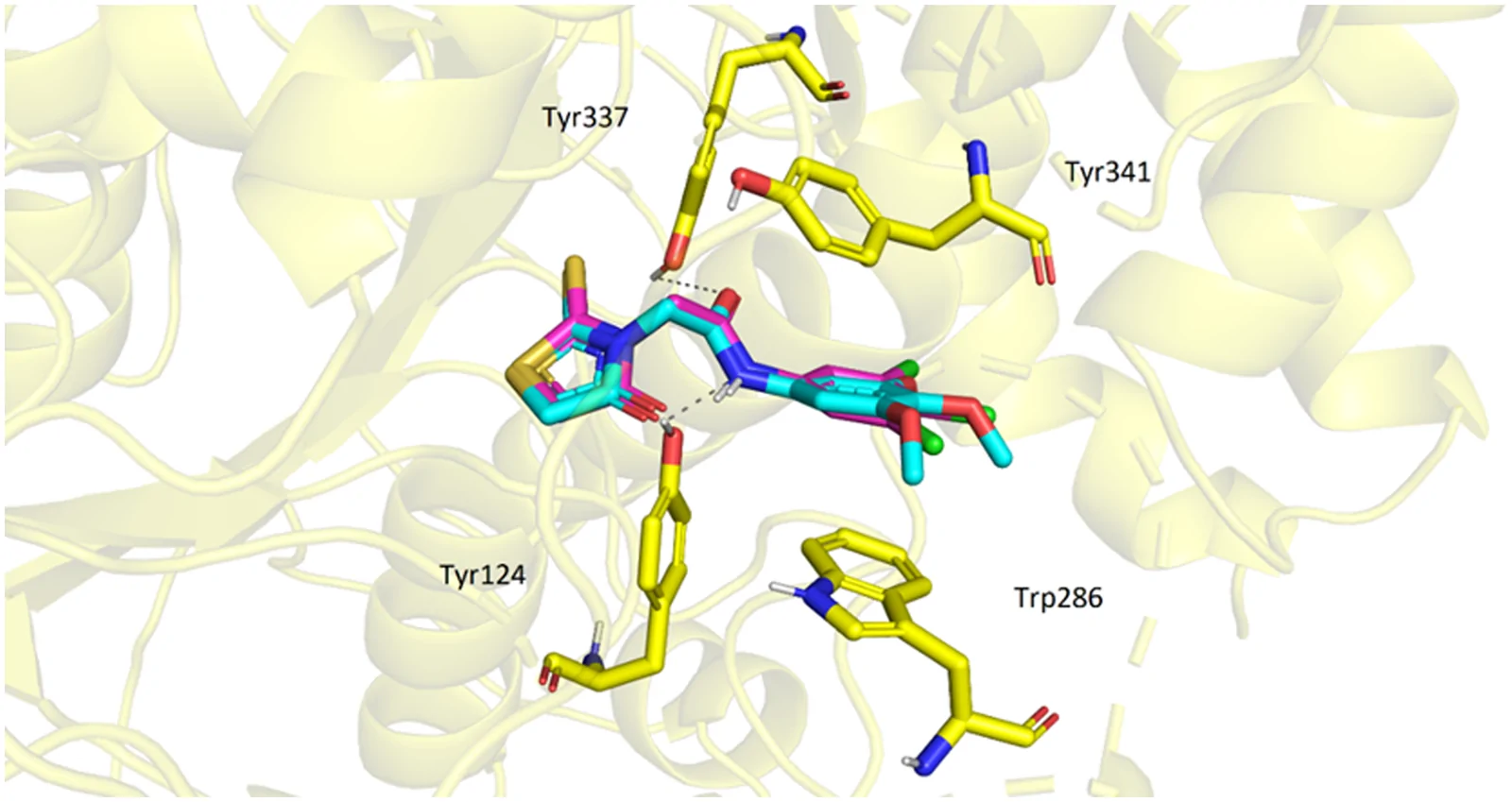
Ligand 1l (cyan) and ligand 1h (pink) in the active site of AChE.

Compounds 1l and 1h, which displayed similar orientations in the active site of AChE, showed different conformations in BChE. According to the binding scores, 1h is preferred over 1l by BChE (binding energy −7.8 kcal mol^−1^*vs.* −7.1 kcal mol^−1^, respectively), which corresponds with the results of *in vitro* testing. The top-scored conformation was buried deep within the cavity, localized near the active site Ser198 and His438. The trichlorophenyl part of the molecule was directed to the hydrophobic region formed by residues Gly117, Val288, Leu286, and Phe398. The thiazolidine moiety was further stabilized by T-stacking with Tyr440 (Fig. 7).

**Fig. 7:**
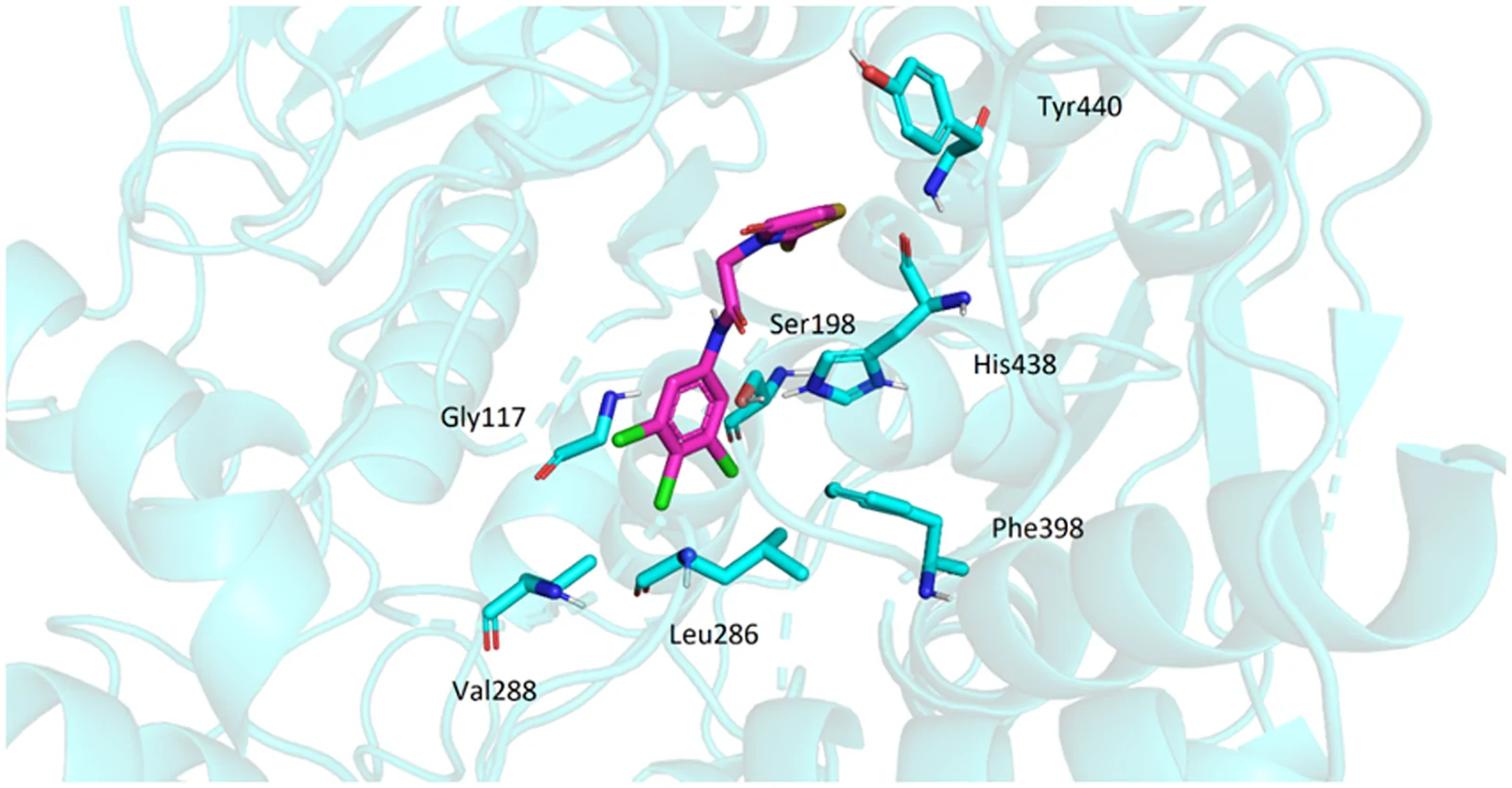
Ligand 1h and its interactions with the active site residues of BChE.

In contrast, 1l was located just below the surface of the enzyme, far from the active site triad. The molecule was oriented with its thiazolidine moiety pointing out of the enzyme cavity. This position allows the formation of a hydrogen bond with Pro285 and other van der Waals interactions with Gly116, Gly117, Trp231, Phe398, and Phe329 (Fig. 8).

**Fig. 8:**
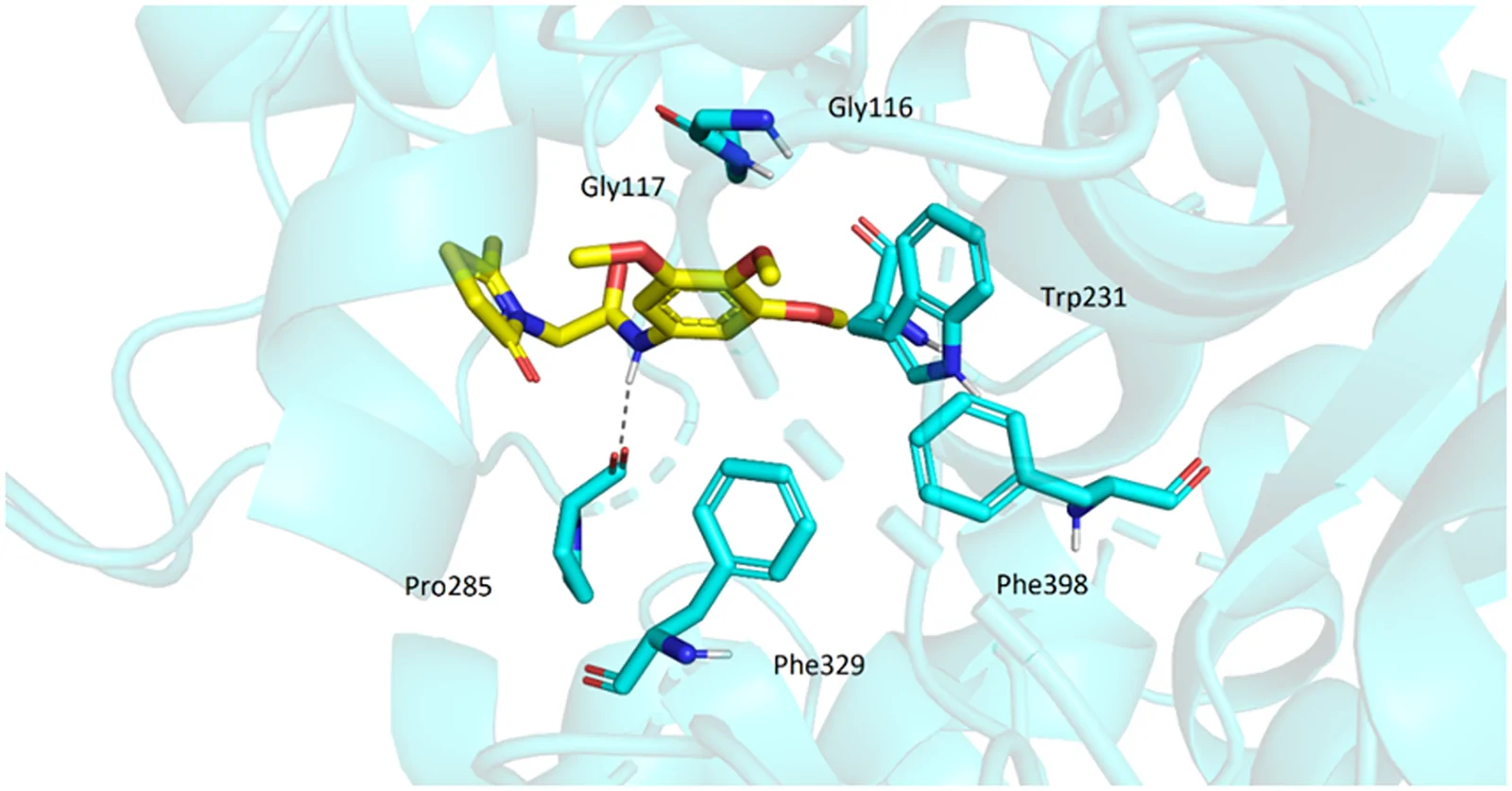
Ligand 1l (yellow) in the active site of BChE.

Compound 2f, also a potent inhibitor of BChE *in vitro*, was located deep in the cavity but relatively far from the catalytic triad (Fig. 9). The ligand formed interactions with residues Gly115, Gly116, Tyr128, and Trp430. Furthermore, a hydrogen bond with Thr120 and π–π interactions with Trp82 and Tyr440 were observed.

**Fig. 9:**
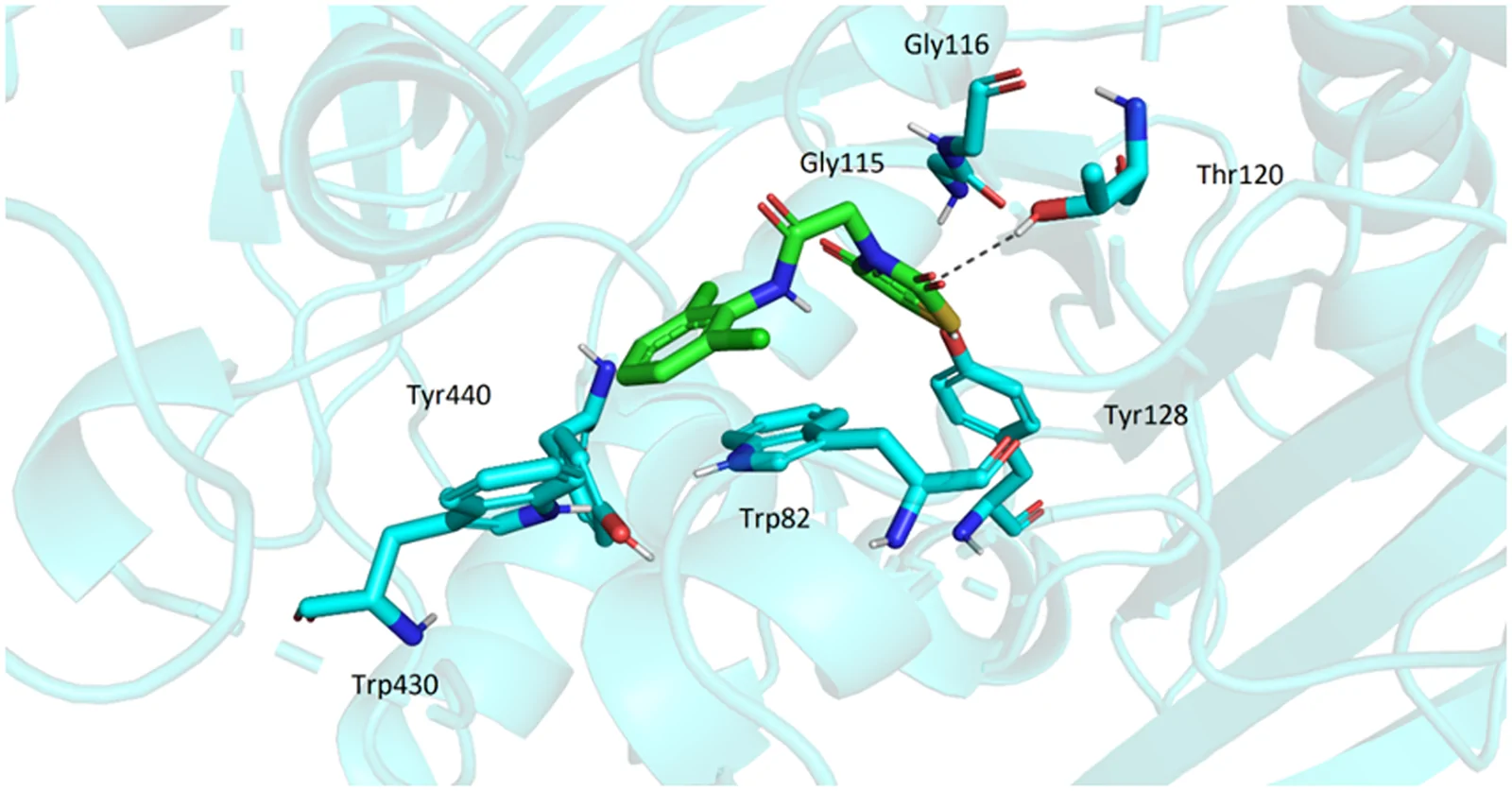
Ligand 2f in the active site of BChE, together with interacting residues.

### Total antioxidant capacity

Given the well-established link between oxidative stress, molecular and cellular damage, and AD,^22,42,43^ as well as the potential of antioxidants to mitigate AD symptoms^21^ (although their therapeutic efficacy remains debated^22^), compounds with multitarget action that combine antioxidant properties with AChE and BChE inhibition represent a promising neuroprotective strategy.

To further explore this approach, we evaluated the antioxidant properties of selected inhibitors, specifically rhodanines 1h, 1n, and 1o, and thiazolidinediones 2e and 2n. The selection criteria for these compounds were based on their inhibitory activity (1h, 1o, and 2e) and the presence of phenolic groups in 1n and 2n, commonly associated with natural and synthetic antioxidants. Antioxidant capacity (TAC) was determined using a commercially available and well-established Antioxidant Assay kit, which is based on the reduction of copper(ii) ions to copper(i) ions by antioxidants, forming a coloured complex with a dye reagent. The intensity of the colour is directly proportional to the TAC of the sample. As the antioxidant activity was considered complementary to enzyme inhibition, compound concentrations were selected based on their IC_50_ values for BChE inhibition: one concentration equal to the IC_50_ and another ten times higher.

The results are summarized in Table 2. All compounds exhibited measurable antioxidant capacity, which increased at the higher tested concentration. TAC values ranged from 18.0 to 648.5 μM at IC_50_ concentrations and 26.0 to 2033.5 μM at tenfold higher concentrations. The lowest absolute activity was observed for 2e [*N*-(3,5-dichlorophenyl)amide of thiazolidinedione-based acid]. In contrast, 3,4,5-trihalogenated phenylamides of rhodanine-3-acetic acid (1h and 1o) demonstrated the most pronounced relative increase in TAC upon a tenfold increase in concentration. Notably and as expected, *N*-(3,5-dichloro-4-hydroxyphenyl)amides exhibited the highest absolute TAC values (260–2033.5 μM). Notably, the thione-containing rhodanine 1n yielded higher TAC values than its ketone isostere 2n at both tested levels, despite being tested at lower concentrations. However, they were also tested at substantially higher concentrations than the more potent BChE inhibitors. Because the tested concentrations were defined individually, the absolute TAC values cannot be used to rank the intrinsic antioxidant potency of the compounds or to derive antioxidant SAR.

**Table 2:** Total antioxidant capacities

Code	Concentration [μM]	TAC [μM]
1h	2.9	44.0
	29	288.0
1n	40.7	**648.5**
	407	**2033.5**
1o	4.5	67.0
	45	326.5
2e	4.93	18.0
	49.3	26.0
2n	59.8	260.0
	598	1004.0

In summary, the tested compounds demonstrated antioxidant properties independent of their enzyme inhibitory activities, supporting their potential multitarget profile.

## Conclusions

In this study, we synthesized a series of new rhodanine and thiazolidinedione derivatives and thoroughly investigated their biological activities. Systematic variation of the aromatic substitution pattern together with matched C=S/C=O analogues provided new SAR information on the influence of halogenation, substituent properties, and heterocyclic-core composition on cholinesterase inhibition. The *N*-phenylamides were prepared in sufficient yields utilizing two synthetic protocols: a carbodiimide-mediated coupling and a MW-assisted procedure.

Enzyme inhibition assays revealed broad dual inhibition of AChE and BChE, with BChE generally inhibited at lower concentrations. Polyhalogenated derivatives, particularly 3,4,5-trichloroanilides and bromo/fluoro-substituted amides, emerged as the most potent inhibitors, highlighting the importance of lipophilic, electronegative substituents. Rhodanine-based compounds exhibited superior inhibitory potency compared to thiazolidinedione analogues, indicating a favourable role of the sulfur atom. Advanced experiments confirmed specific, reversible, and competitive inhibition of both BChE and AChE. The most promising compounds, particularly 1h, 1e, and 1o, demonstrated significant inhibition of both cholinesterases with high selectivity for BChE, making them candidates for further optimization. Selected derivatives additionally showed measurable antioxidant capacity. Taken together, these findings support the potential of the most active derivatives as MTDLs for further investigation in neurodegenerative disease research.

Overall, this study highlights *C*-5-unsubstituted *N*-3-acetamide rhodanines and thiazolidine-2,4-diones as a promising platform for further optimization of dual cholinesterase inhibitors with antioxidant properties.

Further evaluation using human enzymes and assessment of toxicity, CNS-relevant physicochemical, permeability, stability, and pharmacokinetic properties will be required to determine whether optimization of this chemotype can translate into therapeutically relevant compounds.

## Author contributions

Conceptualization, M. K.; methodology, E. Š., A. K., M. Š., J. P., Š. Š., and M. K.; investigation, E. Š., A. K., M. Š., J. P., Š. Š., and M. K.; writing – original draft preparation, E. Š., A. K., M. Š., Š. Š., and M. K.; writing – review and editing, Š. Š. and M. K.; supervision, Š. Š. and M. K.; funding acquisition, M. K. All authors have read and agreed to the published version of the manuscript.

## Conflicts of interest

There are no conflicts to declare.

## Supplementary Material

MD-017-D6MD00502K-s001

MD-017-D6MD00502K-s002

## Data Availability

The supporting data has been provided as part of the supplementary information (SI). Supplementary information: Tables S1–S3, Fig. S1, characterization of compounds, NMR spectra and IC_50_ graphs. See DOI: https://doi.org/10.1039/d6md00502k.
