# Supplementary material for: Rhodanine and 2,4-dioxothiazolidine-based amides: synthesis and evaluation as dual-action cholinesterase inhibitors and antioxidants
Source: RSC Med Chem. 2026 Aug 18;17(10):5058–68. doi: 10.1039/d6md00502k (PMC13635992; doi:10.1039/d6md00502k)
Supplement: Supplementary file 1 [file MD-017-D6MD00502K-s001.pdf]

Supplementary Information (SI) for RSC Medicinal Chemistry.
This journal is © The Royal Society of Chemistry 2026

Rhodanine and 2,4-Dioxothiazolidine-Based Amides: Synthesis and Evaluation as Dual-Action Cholinesterase Inhibitors and Antioxidants

Enikő Šikorová,[a] Adéla Šimková,[a] Markéta Švarcová,[a] Jiří Palarčík,[b] Šárka Štěpánková*[,c] and Martin Krátký*[a]

[a] Department of Organic and Bioorganic Chemistry, Faculty of Pharmacy in Hradec Králové, Charles University, Akademika Heyrovského 1203, 500 03 Hradec Králové, Czech Republic
[b] Institute of Environmental and Chemical Engineering, Faculty of Chemical Technology, University of Pardubice, Studentská 573, 532 10 Pardubice, Czech Republic
[c] Department of Biological and Biochemical Sciences, Faculty of Chemical Technology, University of Pardubice, Studentská 573, 532 10 Pardubice, Czech Republic
* Correspondence: martin.kratky@faf.cuni.cz; sarka.stepankova@upce.cz

1. Supplementary tables S1–S3
2. Supplementary figure Fig. S1
3. Characterization of the synthesized compounds
4. NMR spectra
5. The IC_{50} values graphs used for their calculations

1. Tables S1-S3

Table S1. Activity of BChE in the presence of various concentrations of Tween 20.

**concentration of Tween 20 [%]**	**BChE activity [U]**
0	78
0.0001	76
0.001	76
0.01	77

The activities are expressed as mean values of three independent measurements. BChE: butyrylcholinesterase.

Table S2. Reaction rates of inhibition of AChE and BChE by **1h** in the presence and absence of Tween 20.

**concentration of Tween 20 (%)**	**v [s^{-1}] AChE**	**v [s^{-1}] BChE**
0	0.00691	0.00649
0.0001	0.00683	0.00652
0.001	0.00700	0.00648
0.01	0.00689	0.00655
0.1	0.00690	0.00640
1	0.00701	0.00642

The reaction rates are expressed as mean values of at least two independent measurements. AChE: acetylcholinesterase.

Table S3. Reaction rates of AChE and BChE inhibition with and without preincubation of the enzyme with **1h**.

**Preincubation**	**v [s^{-1}] AChE**	**v [s^{-1}] BChE**
0 min	0.00691	0.00649
5 min	0.00706	0.00641

The reaction rates are expressed as mean values of at least two independent measurements.

2. Figure S1

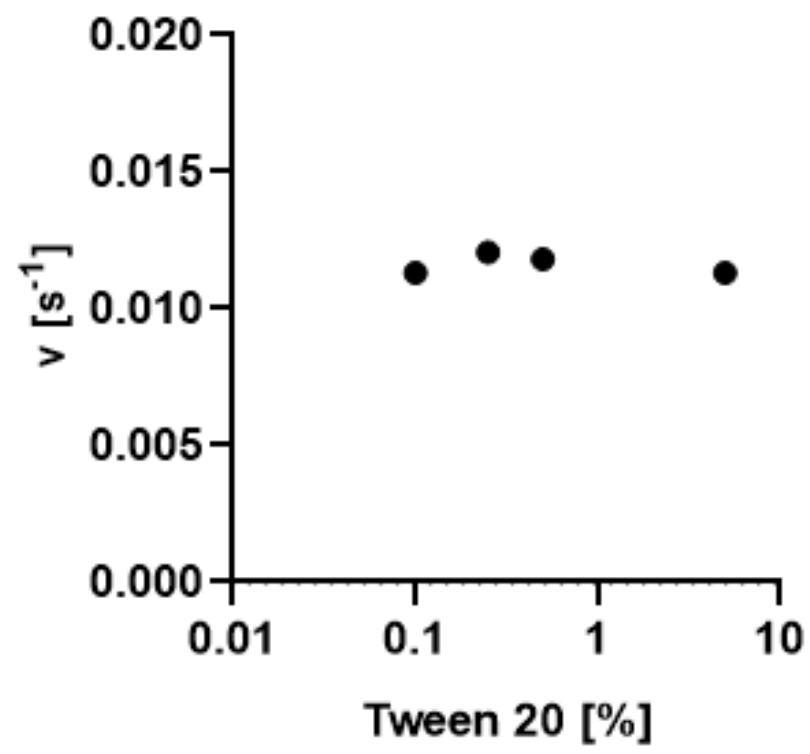

Figure S1. The effect of various concentrations of Tween 20 on the reaction rate of BChE (the reaction rate in the absence of Tween 20 was 0.01131 s^{-1}).
The measurements were done in duplicate, and the points are expressed as mean values.

3. Characterization of the synthesized compounds

N-(4-Chlorophenyl)-2-(2,4-dioxothiazolidin-3-yl)acetamide **2a**. Yellowish solid; yield 63% (method B); mp: 211.8–212.5 °C; R_f: 0.63. IR (cm^{-1}): 3327, 1749, 1677, 1599, 1542, 1491, 1402, 1377, 1328, 1301, 1286, 1250, 1210, 1161, 1133, 1092, 1011, 982, 962, 892, 843, 825, 787, 710, 695, 666, 620. ^{1}H NMR (500 MHz, DMSO-d_6): δ 10.47 (s, 1H, NH), 7.61 – 7.53 (m, 2H, H3, H5), 7.41 – 7.34 (m, 2H, H2, H6), 4.34 (s, 2H, NC$\underline{H_2}$CO), 4.33 (s, 2H, C$\underline{H_2}$S). ^{13}C NMR (126 MHz, DMSO-d_6): δ 172.01, 171.64, 164.09, 137.38, 128.81, 127.24, 120.69, 43.72, 34.00. Elemental analysis for $C_{11}H_9ClN_2O_3S$ (284.71): Calculated: C, 46.40; H, 3.19; N, 9.84. Found: C, 46.45; H, 3.21; N, 9.79.

N-(3-Chlorophenyl)-2-(4-oxo-2-thioxothiazolidin-3-yl)acetamide **1b**. Orange solid; yield 39% (method A); mp: 163.5–164.5 °C; R_f: 0.69. IR (cm^{-1}): 3237, 3188, 3079, 2974, 2928, 1739, 1667, 1594, 1547, 1481, 1431, 1400, 1357, 1335, 1316, 1274, 1216, 1166, 1162, 1043, 975, 900, 885, 777, 703, 676, 632. ^{1}H NMR (600 MHz, DMSO-d_6): δ 10.55 (s, 1H, NH), 7.73 (t, J = 2.1 Hz, 1H, H2), 7.41 – 7.38 (m, 1H, H6), 7.35 (t, J = 8.0 Hz, 1H, H5), 7.15 – 7.12 (m, 1H, H4), 4.71 (s, 2H, NC$\underline{H_2}$CO), 4.42 (s, 2H, C$\underline{H_2}$S). ^{13}C NMR (151 MHz, DMSO-d_6): δ 203.06, 173.90, 163.69, 139.81, 133.15, 130.58, 123.36, 118.61, 117.49, 46.50, 36.06. Elemental analysis for $C_{11}H_9ClN_2O_2S_2$ (300.77): Calculated: C, 43.93; H, 3.02; N, 9.31. Found: C, 43.90; H, 3.06; N, 9.40.

N-(3-Chlorophenyl)-2-(2,4-dioxothiazolidin-3-yl)acetamide **2b**. White solid; yield 44% (method A); mp: 143.5–144.0 °C; R_f: 0.56. IR (cm^{-1}): 3248, 3075, 1748, 1682, 1594, 1551, 1484, 1410, 1382, 1324, 1299, 1248, 1206, 1152, 1099, 1042, 988, 935, 898, 879, 789, 745, 707, 686, 666, 657, 642, 628, 616, 603. ^{1}H NMR (600 MHz, DMSO-d_6): δ 10.53 (s, 1H, NH), 7.74 (t, J = 2.0 Hz, 1H, H2), 7.40 (ddd, J = 8.1, 2.0, 1.1 Hz, 1H, H6), 7.35 (t, J = 8.1 Hz, 1H, H5), 7.14 (ddd, J = 8.1, 2.0, 1.1 Hz, 1H, H4), 4.35 (s, 2H, NC$\underline{H_2}$CO), 4.33 (s, 2H, C$\underline{H_2}$S). ^{13}C NMR (151 MHz, DMSO-d_6): δ 171.95, 171.58, 164.32, 139.80, 133.15, 130.59, 123.39, 118.63, 117.51, 43.73, 33.97. Elemental analysis for $C_{11}H_9ClN_2O_3S$ (284.71): Calculated: C, 46.40; H, 3.19; N, 9.84. Found: C, 46.34; H, 3.16; N, 9.89.

N-(2-Chlorophenyl)-2-(4-oxo-2-thioxothiazolidin-3-yl)acetamide **1c**. Yellow solid; yield 39% (method A); mp: 189.0–190.0 °C; R_f: 0.69. IR (cm^{-1}): 3281, 1736, 1669, 1589, 1533, 1471, 1443, 1406, 1359, 1336, 1288, 1240, 1217, 1200, 1180, 1157, 1115, 1043, 976, 953, 887, 831, 785, 764, 747, 712, 694, 660, 640. ^{1}H NMR (600 MHz, DMSO-d_6): δ 9.98 (s, 1H, NH), 7.65 (dd, J = 8.1, 1.6 Hz, 1H, H6), 7.51 (dd, J = 8.0, 1.5 Hz, 1H, H3), 7.32 (td, J = 7.7, 1.5 Hz, 1H, H5), 7.21 (td, J = 7.7, 1.6 Hz, 1H, H4), 4.78 (s, 2H, NC$\underline{H_2}$CO), 4.40 (s, 2H, C$\underline{H_2}$S). ^{13}C NMR (151 MHz, DMSO-d_6): δ 203.01, 173.92, 163.92, 134.24, 129.58, 127.48, 126.63, 126.40, 125.97, 46.31, 36.10. Elemental analysis for $C_{11}H_9ClN_2O_2S_2$ (300.77): Calculated: C, 43.93; H, 3.02; N, 9.31. Found: C, 43.96; H, 3.00; N, 9.35.

N-(2-Chlorophenyl)-2-(2,4-dioxothiazolidin-3-yl)acetamide **2c**. Light yellow to white solid; yield 41% (method A); mp: 205.6–206.0 °C; R_f: 0.56. IR (cm^{-1}): 3325, 2947, 1750, 1676, 1600, 1543, 1491, 1401, 1377, 1328, 1302, 1287, 1251, 1229, 1211, 1162, 1133, 1092, 1011, 984, 962, 892, 843, 825, 788, 767, 711, 696, 665, 649, 634, 619. ^{1}H NMR (600 MHz, DMSO-d_6): δ 9.95 (s, 1H, NH), 7.68 (dd, J = 8.1, 1.7 Hz, 1H, H6), 7.51 (dd, J = 8.0, 1.5 Hz, 1H, H3), 7.33 (td, J = 7.7, 1.5 Hz, 1H, H5), 7.21 (td, J = 7.7, 1.6 Hz, 1H, H4), 4.43 (s, 2H, NC$\underline{H_2}$CO), 4.32 (s, 2H, C$\underline{H_2}$S). ^{13}C NMR (151 MHz, DMSO-d_6): δ 171.94, 171.57, 164.53, 134.23, 129.59, 127.50, 126.62, 126.30, 125.89, 43.50, 34.00. Elemental analysis for $C_{11}H_9ClN_2O_3S$ (284.71): Calculated: C, 46.40; H, 3.19; N, 9.84. Found: C, 46.37; H, 3.23; N, 9.87.

N-(3,4-Dichlorophenyl)-2-(4-oxo-2-thioxothiazolidin-3-yl)acetamide **1d**. Light yellow to white solid; yield 46% (method A); mp: 172.7–173.2 °C; R_f: 0.65. IR (cm^{-1}): 2983, 2939, 1738, 1597, 1490, 1460, 1430, 1400, 1386, 1365, 1352, 1326, 1300, 1256, 1217, 1191, 1139, 1110, 1089, 1040, 1000, 976, 956, 936, 882, 858, 841, 768, 759, 746, 667, 638, 617. ^{1}H NMR (600 MHz, DMSO-d_6): δ 10.67 (s, 1H, NH), 7.91 (d, J = 2.5 Hz, 1H, H2), 7.58 (d, J = 8.8 Hz, 1H, H5), 7.43 (dd, J = 8.8, 2.5 Hz, 1H, H6), 4.72 (s, 2H, NC$\underline{H_2}$CO), 4.42 (s, 2H, C$\underline{H_2}$S). ^{13}C NMR (151 MHz, DMSO-d_6): δ 203.04, 173.88, 163.87, 138.44, 131.10, 130.82, 125.14, 120.36, 119.18, 46.51, 36.07. Elemental analysis for $C_{11}H_8Cl_2N_2O_2S_2$ (335.22): Calculated: C, 39.41; H, 2.41; N, 8.36. Found: C, 39.47; H, 2.38; N, 8.40.

N-(3,4-Dichlorophenyl)-2-(2,4-dioxothiazolidin-3-yl)acetamide **2d**. Sand yellow solid; yield 46% (method A); mp: 195.8–196.2 °C; R_f: 0.54. IR (cm^{-1}): 3321, 2841, 1748, 1731, 1704, 1681, 1647,

1587, 1526, 1508, 1489, 1472, 1456, 1398, 1362, 1339, 1325, 1288, 1237, 1207, 1161, 1134, 1028, 982, 895, 864, 824, 789, 764, 745, 720, 681, 663, 653, 644, 636, 623, 611. ^{1}H NMR (600 MHz, DMSO-*d*$_6$): δ 10.63 (s, 1H, NH), 7.92 (d, J = 2.4 Hz, 1H, H2), 7.58 (d, J = 8.8 Hz, 1H, H5), 7.44 (dd, J = 8.8, 2.5 Hz, 1H, H6), 4.35 (s, 2H, NC<u>H</u>$_2$CO), 4.33 (s, 2H, C<u>H</u>$_2$S). ^{13}C NMR (151 MHz, DMSO-*d*$_6$): δ 171.93, 171.56, 164.49, 138.42, 131.10, 130.83, 125.16, 120.38, 119.20, 43.73, 33.97. Elemental analysis for $C_{11}H_8Cl_2N_2O_3S$ (319.16): Calculated: C, 41.40; H, 2.53; N, 8.78. Found: C, 41.44; H, 2.60; N, 8.82.

N-(3,5-Dichlorophenyl)-2-(4-oxo-2-thioxothiazolidin-3-yl)acetamide **1e**. Light brown solid; yield 58% (method A); mp: 218.4–219.2 °C; R*f*: 0.70. IR (cm^{-1}): 3243, 3071, 1770, 1737, 1732, 1716, 1704, 1697, 1688, 1673, 1647, 1622, 1589, 1543, 1525, 1508, 1489, 1472, 1455, 1442, 1417, 1398, 1359, 1338, 1322, 1270, 1214, 1192, 1115, 1045, 977, 934, 881, 856, 807, 778, 751, 686, 671, 629, 620. ^{1}H NMR (600 MHz, DMSO-*d*$_6$): δ 10.72 (s, 1H, NH), 7.59 (d, J = 2.0 Hz, 2H, H2, H6), 7.31 (t, J = 2.0 Hz, 1H, H4), 4.72 (s, 2H, NC<u>H</u>$_2$CO), 4.42 (s, 2H, C<u>H</u>$_2$S). ^{13}C NMR (151 MHz, DMSO-*d*$_6$): δ 203.02, 173.85, 164.11, 140.63, 134.20, 122.93, 117.28, 46.55, 36.08. Elemental analysis for $C_{11}H_8Cl_2N_2O_2S_2$ (335.22): Calculated: C, 39.41; H, 2.41; N, 8.36. Found: C, 39.35; H, 2.45; N, 8.42.

N-(3,5-Dichlorophenyl)-2-(2,4-dioxothiazolidin-3-yl)acetamide **2e**. Brown-grey solid; yield 39% (method A); mp: 193.0–193.8 °C; R*f*: 0.56. IR (cm^{-1}): 3262, 3084, 1751, 1676, 1588, 1551, 1489, 1442, 1415, 1400, 1381, 1329, 1299, 1268, 1226, 1212, 1161, 1135, 1112, 1102, 983, 931, 886, 899, 857, 806, 789, 699, 666, 653, 644, 634, 627. ^{1}H NMR (600 MHz, DMSO-*d*$_6$): δ 10.68 (s, 1H, NH), 7.60 (d, J = 1.7 Hz, 2H, H2, H6), 7.30 (t, J = 1.9 Hz, 1H, H4), 4.36 (s, 2H, NC<u>H</u>$_2$CO), 4.33 (s, 2H, C<u>H</u>$_2$S). ^{13}C NMR (151 MHz, DMSO-*d*$_6$): δ 171.90, 171.52, 164.73, 140.61, 134.20, 122.95, 117.31, 43.77, 33.97. Elemental analysis for $C_{11}H_8Cl_2N_2O_3S$ (319.16): Calculated: C, 41.40; H, 2.53; N, 8.78. Found: C, 41.32; H, 2.55; N, 8.70.

N-(2,6-Dichlorophenyl)-2-(4-oxo-2-thioxothiazolidin-3-yl)acetamide **1f**. Yellow solid; yield 54% (method B); mp: 223.6–224.9 °C; R*f*: 0.81. IR (cm^{-1}): 3198, 2930, 1740, 1668, 1573, 1530, 1454, 1437, 1402, 1360, 1329, 1273, 1250, 1218, 1188, 1156, 1105, 1042, 981, 881, 841, 780, 676, 654, 633, 605. ^{1}H NMR (600 MHz, DMSO-*d*$_6$): δ 10.23 (s, 1H, NH), 7.53 (d, J = 8.1 Hz, 2H, H3, H5), 7.35 (t, J = 8.1 Hz, 1H, H4), 4.73 (s, 2H, NC<u>H</u>$_2$CO), 4.38 (s, 2H, C<u>H</u>$_2$S). ^{13}C NMR (151 MHz, DMSO-*d*$_6$): δ 202.75, 173.79, 163.65, 133.55, 132.16, 129.41, 128.54, 45.82, 36.13. Elemental analysis for $C_{11}H_8Cl_2N2O_2S_2$ (335.22): Calculated: C, 39.41; H, 2.41; N, 8.36. Found: C, 39.35; H, 2.45; N, 8.34.

N-(2,6-Dichlorophenyl)-2-(2,4-dioxothiazolidin-3-yl)acetamide **2f**. White solid; yield 51% (method B); mp: 236.1–237.2 °C; R*f*: 0.71. IR (cm^{-1}): 3218, 1747, 1719, 1681, 1613, 1578, 1540, 1455, 1437, 1406, 1397, 1380, 1329, 1275, 1252, 1212, 1166, 1103, 1084, 1048, 983, 969, 896, 852, 794, 781, 761, 708, 684, 663, 634, 619. ^{1}H NMR (600 MHz, DMSO-*d*$_6$): δ 10.24 (s, 1H, NH), 7.54 (d, J = 8.2 Hz, 2H, H3, H5), 7.36 (t, J = 8.1 Hz, 1H, H4), 4.36 (s, 2H, NC<u>H</u>$_2$CO), 4.30 (s, 2H, C<u>H</u>$_2$S). ^{13}C NMR (151 MHz, DMSO-*d*$_6$): δ 171.78, 171.40, 164.24, 133.50, 132.16, 129.45, 128.55, 42.99, 34.04. Elemental analysis for $C_{11}H_8Cl_2N_2O_3S$ (319.16): Calculated: C, 41.40; H, 2.53; N, 8.78. Found: C, 41.35; H, 2.47; N, 8.74.

N-(2,4-Dichlorophenyl)-2-(4-oxo-2-thioxothiazolidin-3-yl)acetamide **1g**. Light yellow to white solid; yield 47% (method A); mp: 162.3–163.6 °C; R*f*: 0.76. IR (cm^{-1}): 3245, 2794, 1740, 1669, 1596, 1538, 1490, 1461, 1431, 1402, 1365, 1327, 1300, 1256, 1219, 1193, 1141, 1088, 1040, 1000, 977, 936, 883, 842, 823, 769, 759, 745, 670, 638, 628, 616. ^{1}H NMR (600 MHz, DMSO-*d*$_6$): δ 10.07 (s, 1H, NH), 7.70 – 7.66 (m, 2H, H3, H6), 7.41 (dd, J = 8.8, 2.4 Hz, 1H, H5), 4.79 (s, 2H, NC<u>H</u>$_2$CO), 4.40 (s, 2H, C<u>H</u>$_2$S). ^{13}C NMR (151 MHz, DMSO-*d*$_6$): δ 203.01, 173.91, 164.12, 133.45, 129.71, 129.04, 127.64, 127.24, 126.91, 46.32, 36.12. Elemental analysis for $C_{11}H_8Cl_2N_2O_2S_2$ (335.22): Calculated: C, 39.41; H, 2.41; N, 8.36. Found: C, 39.32; H, 2.36; N, 8.33.

N-(2,4-Dichlorophenyl)-2-(2,4-dioxothiazolidin-3-yl)acetamide **2g**. White solid; yield 57% (method A); mp: >400 °C; R*f*: 0.55. IR (cm^{-1}): 3133, 1748, 1732, 1716, 1704, 1697, 1681, 1647, 1575, 1542, 1522, 1508, 1489, 1473, 1456, 1386, 1339, 1162, 836, 818, 800, 766, 743, 664, 630, 614. ^{1}H NMR (600 MHz, DMSO-*d*$_6$): δ 10.04 (s, 1H, NH), 7.72 (d, J = 8.8 Hz, 1H, H6), 7.68 (d, J = 2.4 Hz, 1H, H3), 7.41 (dd, J = 8.8, 2.4 Hz, 1H, H5), 4.43 (s, 2H, NC<u>H</u>$_2$CO), 4.32 (s, 2H, C<u>H</u>$_2$S). ^{13}C NMR (151 MHz, acetone-*d*$_6$): δ 171.93, 171.55, 164.72, 133.43, 129.68, 129.03, 127.65, 127.12, 126.81, 43.50,

34.00. Elemental analysis for $C_{11}H_8Cl_2N_2O_3S$ (319.16): Calculated: C, 41.40; H, 2.53; N, 8.78. Found: C, 41.49; H, 2.49; N, 8.83.

2-(4-Oxo-2-thioxothiazolidin-3-yl)-*N*-(3,4,5-trichlorophenyl)acetamide **1h**. Light brown solid; yield 36% (method A); mp: 220.9–221.6 °C; R*f*: 0.70. IR (cm^{-1}): 3332, 3170, 3105, 2962, 1726, 1701, 1587, 1526, 1490, 1458, 1438, 1407, 1382, 1366, 1350, 1298, 1288, 1221, 1191, 1151, 1113, 1088, 1043, 975, 946, 936, 880, 852, 812, 782, 769, 759, 745, 711, 688, 670, 658, 648, 633, 618. ^{1}H NMR (600 MHz, DMSO-*d6*): δ 10.80 (s, 1H, NH), 7.79 (s, 2H, H2, H6), 4.72 (s, 2H, NC$\underline{H_2}$CO), 4.42 (s, 2H, C$\underline{H_2}$S). ^{13}C NMR (151 MHz, DMSO-*d6*): δ 203.02, 173.85, 164.26, 138.34, 132.99, 123.87, 119.16, 46.56, 36.10. Elemental analysis for $C_{11}H_7Cl_3N_2O_2S_2$ (369.67): Calculated: C, 35.74; H, 1.91; N, 7.58. Found: C, 35.79; H, 2.01; N, 7.55.

2-(2,4-Dioxothiazolidin-3-yl)-*N*-(3,4,5-trichlorophenyl)acetamide **2h**. Light brown solid; yield 36% (method A); mp: 219.3–219.7 °C; R*f*: 0.57. IR (cm^{-1}): 3285, 3172, 3094, 2982, 2953, 1741, 1709, 1672, 1584, 1525, 1489, 1436, 1419, 1402, 1385, 1323, 1287, 1228, 1196, 1156, 1089, 992, 942, 889, 853, 815, 793, 714, 689, 658, 650. ^{1}H NMR (600 MHz, DMSO-*d6*): δ 10.76 (s, 1H, NH), 7.79 (s, 2H, H2, H6), 4.36 (s, 2H, NC$\underline{H_2}$CO), 4.33 (s, 2H, C$\underline{H_2}$S). ^{13}C NMR (151 MHz, DMSO-*d6*): δ 171.89, 171.51, 164.86, 138.32, 132.98, 123.88, 119.19, 43.77, 33.97. Elemental analysis for $C_{11}H_7Cl_3N_2O_3S$ (353.60): Calculated: C, 37.36; H, 2.00; N, 7.92. Found: C, 37.29; H, 2.07; N, 8.00.

2-(4-Oxo-2-thioxothiazolidin-3-yl)-*N*-(2,4,5-trichlorophenyl)acetamide **1i**. Yellowish solid; yield 67% (method B); mp: 201.8–202.4 °C; R*f*: 0.88. IR (cm^{-1}): 3247, 2929, 1737, 1673, 1575, 1524, 1458, 1398, 1355, 1322, 1271, 1252, 1226, 1193, 1130, 1111, 1079, 1040, 970, 937, 884, 856, 781, 751, 682, 648, 619. ^{1}H NMR (500 MHz, DMSO-*d6*): δ 10.24 (s, 1H, NH), 8.02 (s, 1H, H3), 7.93 (s, 1H, H6), 4.82 (s, 2H, NC$\underline{H_2}$CO), 4.41 (s, 2H, C$\underline{H_2}$S). ^{13}C NMR (126 MHz, DMSO-*d6*): δ 203.04, 173.92, 164.55, 134.45, 130.72, 129.89, 127.57, 125.71, 125.04, 46.39, 36.14. Elemental analysis for $C_{11}H_7Cl_3N_2O_2S_2$ (369.66): Calculated: C, 35.74; H, 1.91; N, 7.58. Found: C, 35.81; H, 1.95; N, 7.56.

2-(2,4-Dioxothiazolidin-3-yl)-*N*-(2,4,5-trichlorophenyl)acetamide **2i**. White solid; yield 69% (method B); mp: 202.3–203.1 °C; R*f*: 0.86. IR (cm^{-1}): 3251, 3091, 2942, 1754, 1685, 1577, 1524, 1659, 1400, 1375, 1359, 1323, 1271, 1253, 1211, 1161, 1130, 1078, 982, 967, 938, 877, 786, 753, 684, 656, 645, 623, 602. ^{1}H NMR (500 MHz, DMSO-*d6*): δ 10.19 (s, 1H, NH), 8.06 (s, 1H, H3), 7.93 (s, 1H, H6), 4.46 (s, 2H, NC$\underline{H_2}$CO), 4.33 (s, 2H, C$\underline{H_2}$S). ^{13}C NMR (126 MHz, DMSO-*d6*): δ 171.96, 171.57, 165.16, 134.44, 130.71, 129.91, 127.56, 125.71, 124.97, 43.58, 34.02. Elemental analysis for $C_{11}H_7Cl_3N_2O_3S$ (353.60): Calculated: C, 37.36; H, 2.00; N, 7.92. Found: C, 37.33; H, 1.97; N, 7.96.

2-(4-Oxo-2-thioxothiazolidin-3-yl)-*N*-(2,4,6-trichlorophenyl)acetamide **1j**. White solid; yield 60% (method B); mp: 254.9–255.8 °C; R*f*: 0.83. IR (cm^{-1}): 3245, 3088, 3001, 2939, 1720, 1677, 1652, 1564, 1526, 1451, 1401, 1389, 1377, 1361, 1337, 1272, 1249, 1226, 1197, 1141, 1130, 1120, 1078, 1045, 963, 890, 973, 863, 821, 807, 780, 755, 697, 660, 647, 622, 611. ^{1}H NMR (600 MHz, DMSO-*d6*): δ 10.29 (s, 1H, NH), 7.76 (s, 2H, H3, H5), 4.73 (s, 2H, NC$\underline{H_2}$CO), 4.38 (s, 2H, C$\underline{H_2}$S). ^{13}C NMR (151 MHz, DMSO-*d6*): δ 202.75, 173.78, 163.79, 134.32, 132.64, 131.57, 128.32, 45.81, 36.15. Elemental analysis for $C_{11}H_7Cl_3N_2O_2S_2$ (369.66): Calculated: C, 35.74; H, 1.91; N, 7.58. Found: C, 35.70; H, 1.94; N, 7.54.

2-(2,4-Dioxothiazolidin-3-yl)-*N*-(2,4,6-trichlorophenyl)acetamide **2j**. White solid; yield 54% (method B); mp: 241.4–242.5 °C; R*f*: 0.75. IR (cm^{-1}): 3210, 2940, 1761, 1683, 1564, 1533, 1449, 1407, 1384, 1348, 1274, 1210, 1162, 1082, 987, 894, 860, 820, 806, 786, 753, 698, 660, 620. ^{1}H NMR (600 MHz, DMSO-*d6*): δ 10.29 (s, 1H, NH), 7.77 (s, 2H, H3, H5), 4.36 (s, 2H, NC$\underline{H_2}$CO), 4.30 (s, 2H, C$\underline{H_2}$S). ^{13}C NMR (151 MHz, DMSO-*d6*): δ 171.78, 171.39, 164.37, 134.28, 132.68, 131.57, 128.34, 42.98, 34.05. Elemental analysis for $C_{11}H_7Cl_3N_2O_3S$ (353.60): Calculated: C, 37.36; H, 2.00; N, 7.92. Found: C, 37.40; H, 2.05; N, 7.94.

2-(4-Oxo-2-thioxothiazolidin-3-yl)-*N*-(3,4,5-trifluorophenyl)acetamide **1k**. Yellowish solid; yield 66% (method B); mp: 198.1–199.0 °C; R*f*: 0.77. IR (cm^{-1}): 3350, 3112, 2983, 2359, 2341, 1715, 1699, 1630, 1602, 1561, 1529, 1504, 1435, 1409, 1394, 1363, 1344, 1298, 1221, 1194, 1128, 1109, 1042, 996, 974, 952, 874, 839, 797, 782, 696, 651, 618, 609. ^{1}H NMR (600 MHz, DMSO-*d6*): δ 10.75 (s, 1H, NH), 7.45 – 7.39 (m, 2H, H2, H6), 4.71 (s, 2H, NC$\underline{H_2}$CO), 4.42 (s, 2H, C$\underline{H_2}$S). ^{13}C NMR (151 MHz, DMSO-*d6*): δ 203.00, 173.86, 164.05, 150.10 (ddd, J = 245.0, 10.1, 5.3 Hz), 135.85–133.96

(2C, m), 103.66–103.30 (m), 46.47, 36.07. Elemental analysis for $C_{11}H_7F_3N_2O_2S_2$ (320.30): Calculated: C, 41.25; H, 2.20; N, 8.75. Found: C, 41.24; H, 2.21; N, 8.79.

2-(2,4-Dioxothiazolidin-3-yl)-*N*-(3,4,5-trifluorophenyl)acetamide **2k**. White solid; yield 59% (method B); mp: 180.1–181.5 °C; R*f*: 0.63. IR (cm^{-1}): 3298, 3101, 2998, 1747, 1715, 1673, 1632, 1561, 1526, 1437, 1418, 1392, 1365, 1329, 1295, 1219, 1204, 1158, 1138, 1085, 1055, 1006, 977, 956, 892, 879, 844, 794, 767, 699, 661, 650, 642, 634, 615. ^{1}H NMR (600 MHz, DMSO-*d₆*): *δ* 10.71 (s, 1H, NH), 7.46 – 7.40 (m, 2H, H2, H6), 4.35 (s, 2H, N<u>CH₂</u>CO), 4.33 (s, 2H, <u>CH₂</u>S). ^{13}C NMR (151 MHz, DMSO-*d₆*): *δ* 171.91, 171.54, 164.67, 150.25 (ddd, *J* = 249.0, 10.1, 5.2 Hz), 135.88 – 133.93 (2C, m), 103.70–103.38 (m), 44.25, 34.52. Elemental analysis for $C_{11}H_7F_3N_2O_3S$ (304.24): C, 43.43; H, 2.32; N, 9.21. Found: C, 43.34; H, 2.31; N, 9.29.

2-(4-Oxo-2-thioxothiazolidin-3-yl)-*N*-(3,4,5-trimethoxyphenyl)acetamide **1l**. Yellowish solid; yield 52% (method B); mp: 221 °C (decomp.); R*f*: 0.61. IR (cm^{-1}): 3267, 3079, 2935, 2838, 1749, 1732, 1662, 1607, 1557, 1511, 1453, 1417, 1400, 1356, 1321, 1254, 1234, 1221, 1195, 1149, 1131, 1037, 993, 970, 953, 917, 882, 853, 820, 782, 697, 660, 626, 617, 602. ^{1}H NMR (600 MHz, DMSO-*d₆*): *δ* 10.32 (s, 1H, NH), 6.91 (s, 2H, H2, H6), 4.68 (s, 2H, NC<u>H₂</u>CO), 4.41 (s, 2H, C<u>H₂</u>S), 3.73 (s, 6H, OCH_3), 3.61 (s, 3H, OCH_3). ^{13}C NMR (151 MHz, DMSO-*d₆*): *δ* 203.07, 173.92, 163.03, 152.77, 134.60, 133.55, 96.66, 60.08, 56.64, 46.40, 35.99. Elemental analysis for $C_{14}H_{16}N_2O_5S_2$ (356.41): Calculated: C, 47.18; H, 4.53; N, 7.86. Found: C, 47.24; H, 4.51; N, 7.89.

2-(2,4-Dioxothiazolidin-3-yl)-*N*-(3,4,5-trimethoxyphenyl)acetamide **2l**. White solid; yield 58% (method B); mp: 198.9–199.6 °C; R*f*: 0.54. IR (cm^{-1}): 3288, 2938, 1745, 1688, 1658, 1607, 1553, 1511, 1469, 1455, 1417, 1406, 1372, 1350, 1323, 1253, 1235, 1186, 1161, 1144, 1130, 1075, 992, 961, 893, 882, 821, 789, 749, 701, 656, 642, 613. ^{1}H NMR (600 MHz, DMSO-*d₆*): *δ* 10.28 (s, 1H, NH), 6.92 (s, 2H, H2, H6), 4.33 (s, 2H, NC<u>H₂</u>CO), 4.31 (s, 2H, C<u>H₂</u>S), 3.73 (s, 6H, OCH_3), 3.61 (s, 3H, OCH_3). ^{13}C NMR (151 MHz, DMSO-*d₆*): *δ* 171.95, 171.59, 163.66, 152.77, 134.55, 133.58, 96.76, 60.08, 55.66, 43.65, 33.93. Elemental analysis for $C_{14}H_{16}N_2O_6S$ (340.35): Calculated: C, 49.41; H, 4.74; N, 8.23. Found: C, 49.44; H, 4.70; N, 8.27.

2-(4-Oxo-2-thioxothiazolidin-3-yl)-*N*-(3,4,5-trimethylphenyl)acetamide **1m**. Yellowish solid; yield 56% (method B); mp: 266.6–267.6 °C; R*f*: 0.83. IR (cm^{-1}): 3245, 3078, 2968, 2924, 1749, 1693, 1673, 1606, 1556, 1486, 1455, 1408, 1359, 1324, 1252, 1239, 1225, 1217, 1190, 1124, 1105, 1039, 977, 966, 885, 863, 780, 753, 729, 704, 678, 605. ^{1}H NMR (600 MHz, DMSO-*d₆*): *δ* 10.07 (s, 1H, NH), 7.16 (s, 2H, H2, H6), 4.66 (s, 2H, NC<u>H₂</u>CO), 4.39 (s, 2H, C<u>H₂</u>S), 2.19 (s, 6H, CH_3), 2.06 (s, 3H, CH_3). ^{13}C NMR (151 MHz, DMSO-*d₆*): *δ* 203.09, 173.95, 162.79, 136.20, 135.38, 129.88, 118.39, 46.42, 36.00, 20.36, 14.59. Elemental analysis for $C_{14}H_{16}N_2O_2S_2$ (308.41): Calculated: C, 54.52; H, 5.23; N, 9.08. Found: C, 54.58; H, 5.31; N, 9.02.

2-(2,4-Dioxothiazolidin-3-yl)-*N*-(3,4,5-trimethylphenyl)acetamide **2m**. White solid; yield 51% (method B); mp: 200.4–201.3 °C; R*f*: 0.71. IR (cm^{-1}): 3252, 3081, 2976, 2936, 1749, 1687, 1660, 1608, 1558, 1487, 1456, 1417, 1403, 1386, 1325, 1242, 1226, 1152, 1088, 990, 972, 900, 887, 866, 789, 765, 749, 708, 684, 654, 627. ^{1}H NMR (600 MHz, DMSO-*d₆*): *δ* 10.06 (s, 1H, NH), 7.17 (s, 2H, H2, H6), 4.31 (s, 2H, NC<u>H₂</u>CO), 4.29 (s, 2H, C<u>H₂</u>S), 2.19 (s, 6H, CH_3), 2.06 (s, 3H, CH_3). ^{13}C NMR (151 MHz, DMSO-*d₆*): *δ* 171.97, 171.62, 163.40, 136.21, 135.38, 129.91, 118.38, 43.65, 33.94, 20.37, 14.59. Elemental analysis for $C_{14}H_{16}N_2O_3S$ (292.35): Calculated: C, 57.52; H, 5.52; N, 9.58. Found: C, 57.48; H, 5.50; N, 9.51.

N-(3,5-Dichloro-4-hydroxyphenyl)-2-(4-oxo-2-thioxothiazolidin-3-yl)acetamide **1n**. Yellowish solid; yield 63% (method B); mp: 224 °C (decomp.); R*f*: 0.63. IR (cm^{-1}): 3250, 3181, 3117, 3071, 2974, 2931, 1742, 1714, 1671, 1598, 1548, 1490, 1406, 1359, 1330, 1247, 1216, 1189, 1145, 1044, 977, 864, 802, 711, 672, 651, 633, 608. ^{1}H NMR (500 MHz, DMSO-*d₆*): *δ* 10.42 (s, 1H, NH), 9.95 (s, 1H, OH), 7.53 (s, 2H, H2, H6), 4.67 (s, 2H, NC<u>H₂</u>CO), 4.41 (s, 2H, C<u>H₂</u>S). ^{13}C NMR (126 MHz, DMSO-*d₆*): *δ* 203.10, 173.94, 163.42, 145.20, 131.45, 122.33, 119.24, 46.44, 36.10. Elemental analysis for $C_{11}H_8Cl_2N_2O_3S_2$ (351.22): Calculated: C, 37.62; H, 2.30; N, 7.98. Found: C, 37.51; H, 2.35; N, 7.99.

N-(3,5-Dichloro-4-hydroxyphenyl)-2-(2,4-dioxothiazolidin-3-yl)acetamide **2n**. White solid; yield 54% (method B); mp: 216 °C (decomp.); R*f*: 0.51. IR (cm^{-1}): 3255, 3117, 2978, 1748, 1682, 1666,

1596, 1546, 1494, 1408, 1385, 1318, 1247, 1221, 1149, 986, 967, 948, 887, 869, 804, 715, 682, 667, 658, 642, 626, 619, 610. ^{1}H NMR (500 MHz, DMSO-d_6): δ 10.41 (s, 1H, NH), 9.96 (s, 1H, OH), 7.54 (s, 2H, H2, H6), 4.32 (s, 2H, NC$\underline{H_2}$CO), 4.30 (s, 2H, C$\underline{H_2}$S). ^{13}C NMR (126 MHz, DMSO-d_6): δ 171.99, 171.61, 164.03, 145.20, 131.45, 122.33, 119.25, 43.65, 34.00. Elemental analysis for $C_{11}H_8Cl_2N_2O_4S$ (335.16): Calculated: C, 39.42; H, 2.41; N, 8.36. Found: C, 39.38; H, 2.34; N, 8.31.

N-(4-Bromo-3,5-difluorophenyl)-2-(4-oxo-2-thioxothiazolidin-3-yl)acetamide **1o**. Yellowish solid; yield 50% (method B); mp: 225 °C (decomp.); R*f*: 0.83. IR (cm^{-1}): 3287, 2926, 1739, 1682, 1604, 1545, 1474, 1423, 1397, 1356, 1328, 1219, 1191, 1148, 1126, 1065, 1031, 1003, 978, 960, 880, 858, 781, 713, 694, 659, 647, 641, 628, 618, 611. ^{1}H NMR (600 MHz, DMSO-d_6): δ 11.33 (s, 1H, NH), 7.54 – 7.43 (m, 2H, H2, H6), 4.75 (s, 2H, NC$\underline{H_2}$CO), 4.42 (s, 2H, C$\underline{H_2}$S). ^{13}C NMR (151 MHz, DMSO-d_6): δ 202.99, 173.86, 164.24, 159.04 (dd, J = 243.4, 6.5 Hz), 139.80 (t, J = 13.0 Hz), 102.90 (d, J = 28.2 Hz), 90.02, 46.58, 36.08. Elemental analysis for $C_{11}H_7BrF_2N_2O_2S_2$ (381.21): Calculated: C, 34.66; H, 1.85; N, 7.35. Found: C, 34.68; H, 1.80; N, 7.36.

N-(4-Bromo-3,5-difluorophenyl)-2-(2,4-dioxothiazolidin-3-yl)acetamide **2o**. White solid; yield 57% (method B); mp: 249 °C (decomp.); R*f*: 0.68. IR (cm^{-1}): 3320, 3141, 2982, 2938, 1764, 1677, 1608, 1539, 1478, 1425, 1407, 1393, 1382, 1324, 1288, 1233, 1205, 1165, 1143, 1078, 1065, 1033, 1011, 992, 960, 891, 861, 790, 720, 694, 658, 637, 625. ^{1}H NMR (600 MHz, DMSO-d_6): δ 10.84 (s, 1H, NH), 7.47 – 7.38 (m, 2H, H2, H6), 4.37 (s, 2H, NC$\underline{H_2}$CO), 4.34 (s, 2H, C$\underline{H_2}$S). ^{13}C NMR (151 MHz, DMSO-d_6): δ 171.90, 171.53, 164.86, 159.07 (dd, J = 243.6, 6.3 Hz), 139.62 (t, J = 13.0 Hz), 102.99 (d, J = 28.6 Hz), 90.20, 43.78, 33.97. Elemental analysis for $C_{11}H_7BrF_2N_2O_3S$ (365.15): Calculated: C, 36.18; H, 1.93; N, 7.67. Found: C, 36.19; H, 1.90; N, 7.66.

4. NMR spectra

^{1}H NMR *N-(4-Chlorophenyl)-2-(2,4-dioxothiazolidin-3-yl)acetamide* (**2a**)

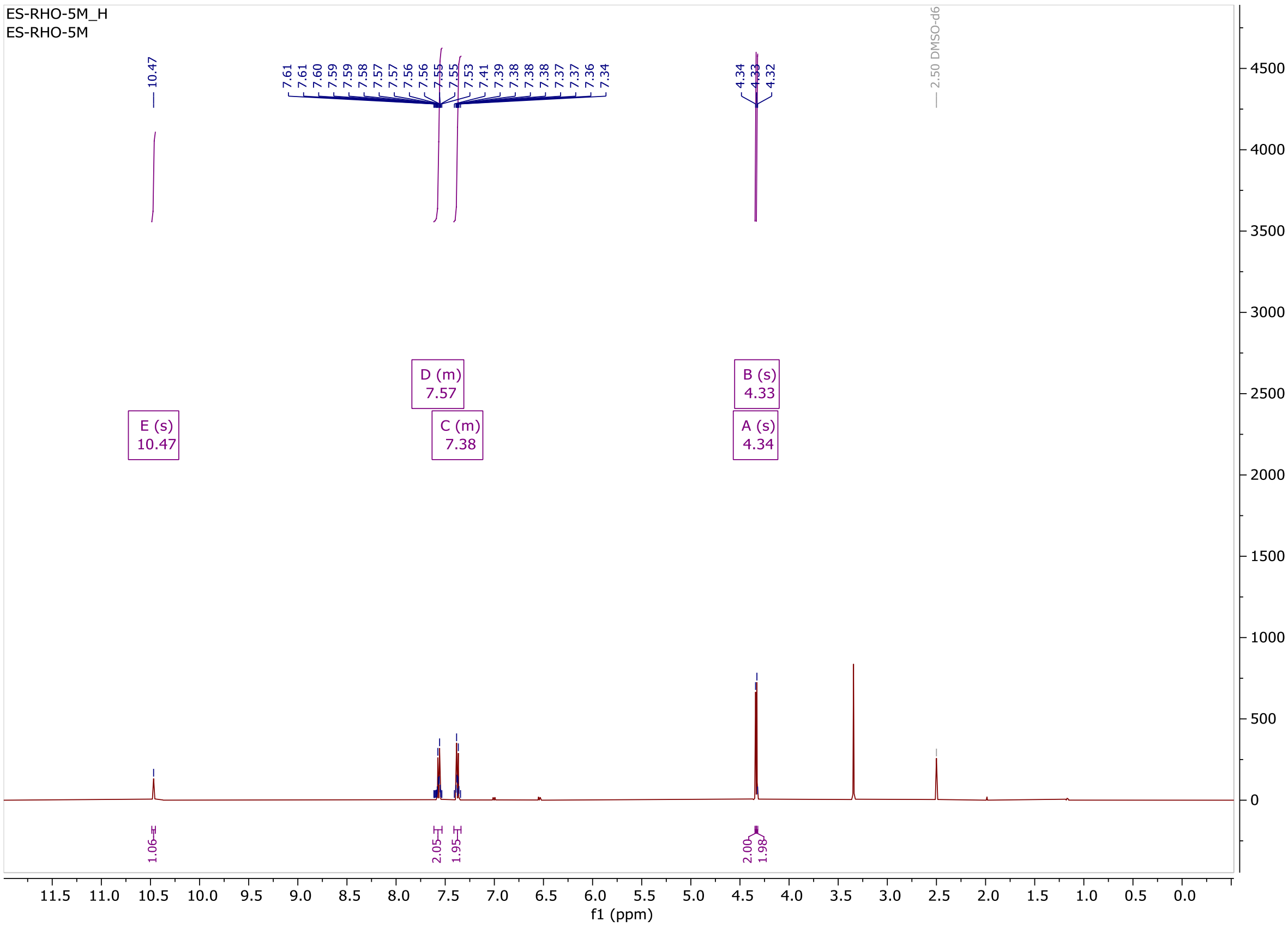

^{13}C NMR *N-(4-Chlorophenyl)-2-(2,4-dioxothiazolidin-3-yl)acetamide* (**2a**)

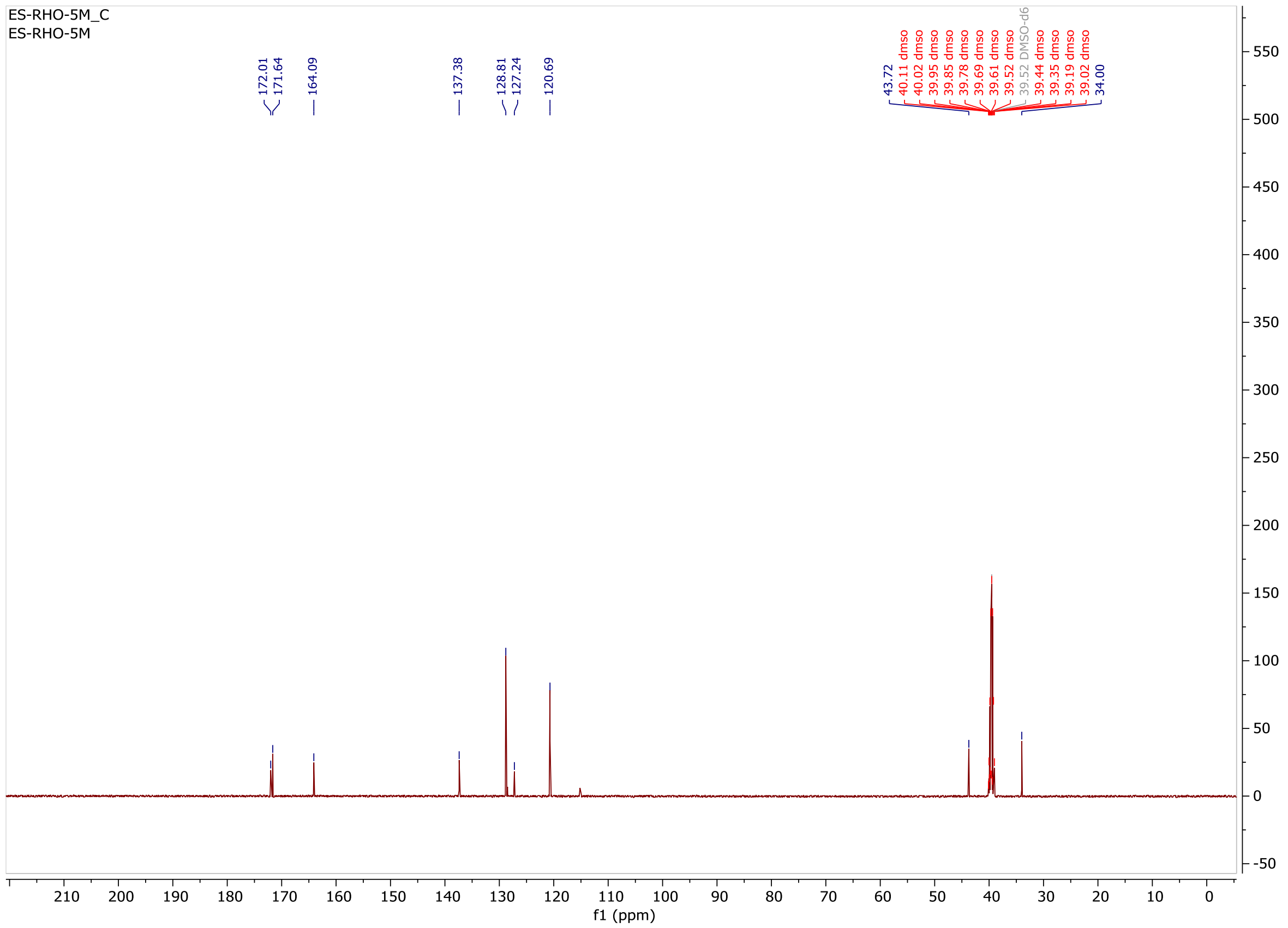

^{1}H NMR *N-(3-Chlorophenyl)-2-(4-oxo-2-thioxothiazolidin-3-yl)acetamide* (**1b**)

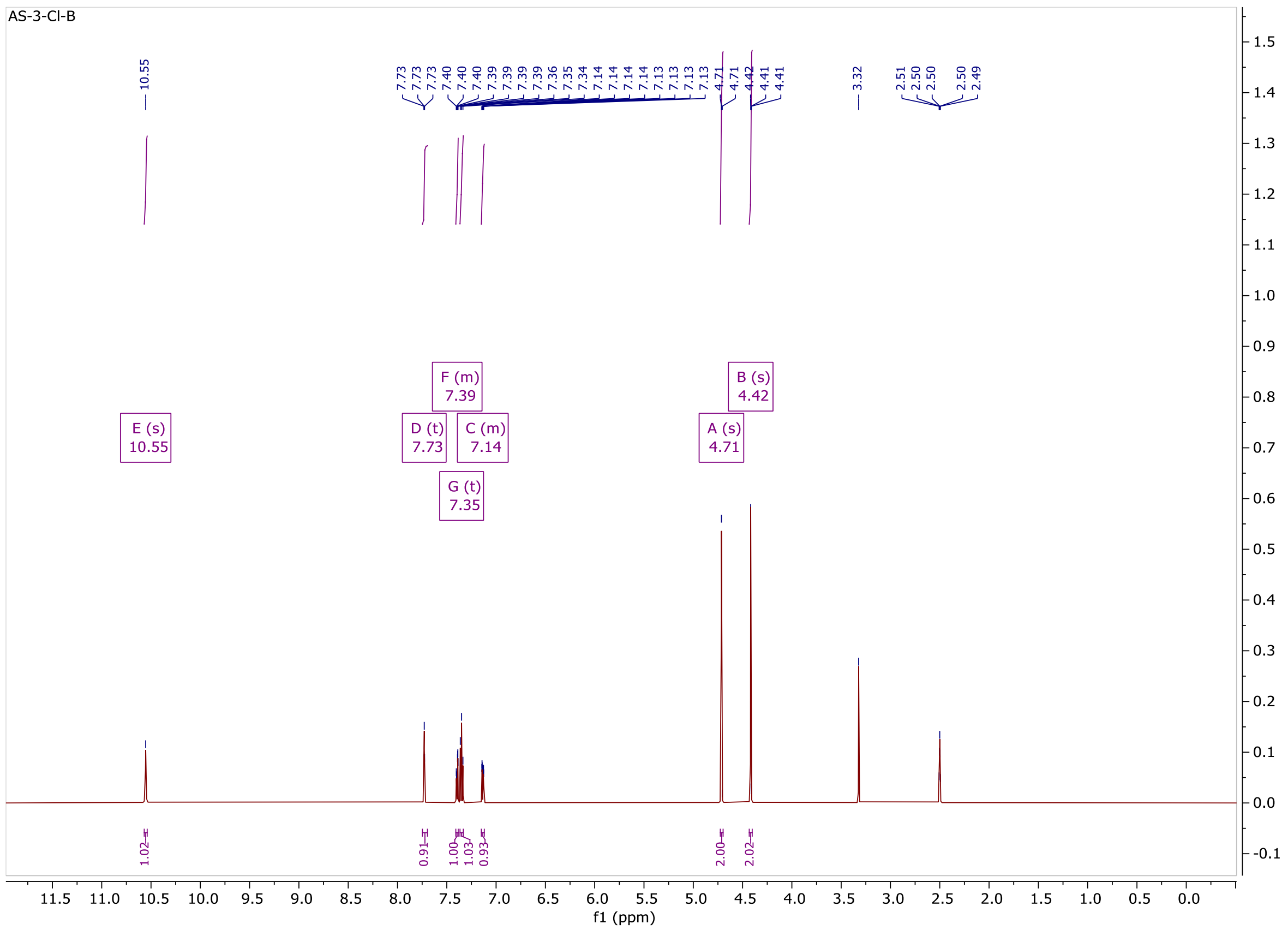

^{13}C NMR *N-(3-Chlorophenyl)-2-(4-oxo-2-thioxothiazolidin-3-yl)acetamide* (**1b**)

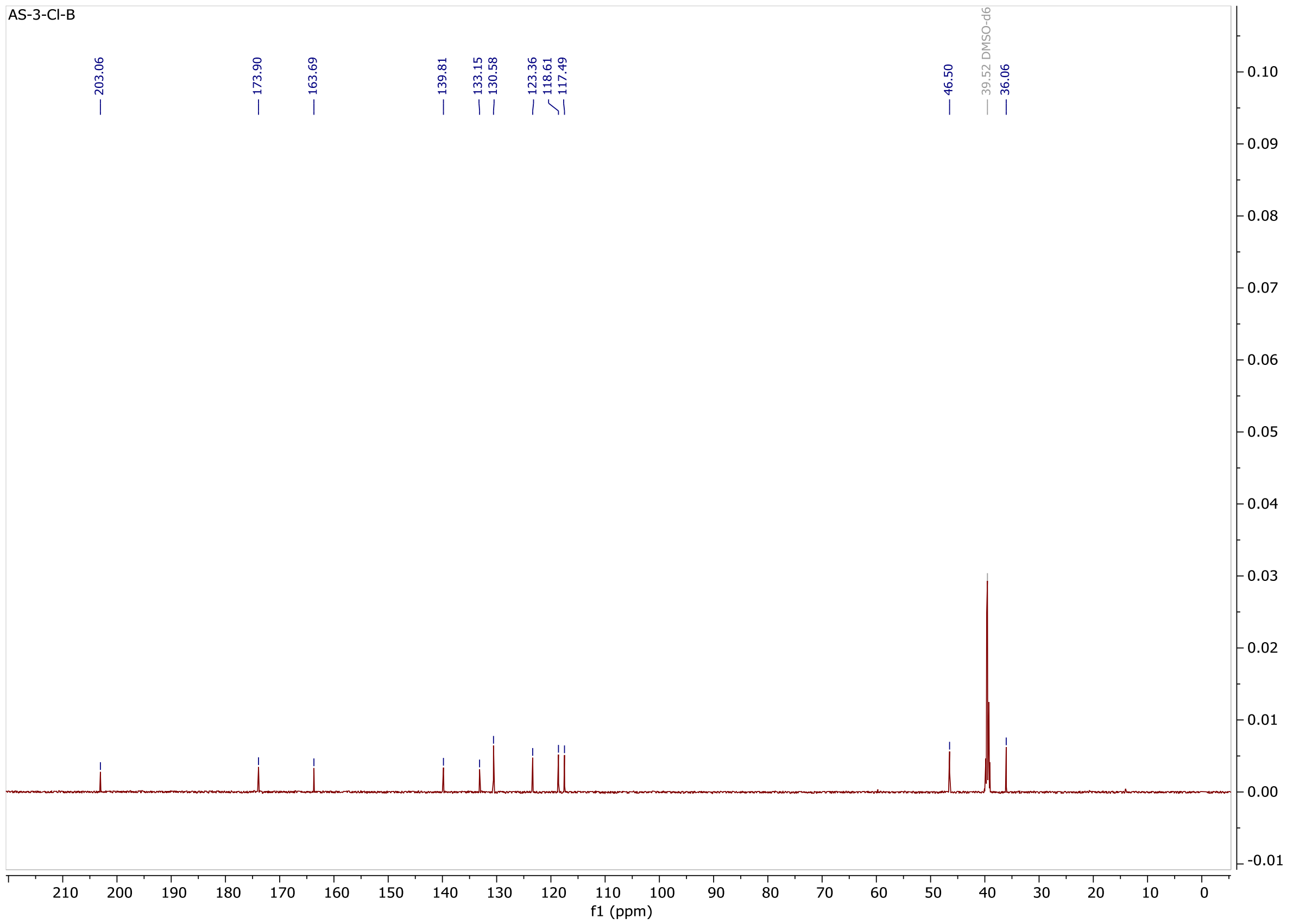

AS-3-Cl-B
203.06
173.90
163.69
139.81
133.15
130.58
123.36
118.61
117.49
46.50
39.52 DMSO-d6
36.06
0.10
0.09
0.08
0.07
0.06
0.05
0.04
0.03
0.02
0.01
0.00
-0.01
210
200
190
180
170
160
150
140
130
120
110
100
90
80
70
60
50
40
30
20
10
0
f1 (ppm)

^{1}H NMR *N-(3-Chlorophenyl)-2-(2,4-dioxothiazolidin-3-yl)acetamide* (**2b**)

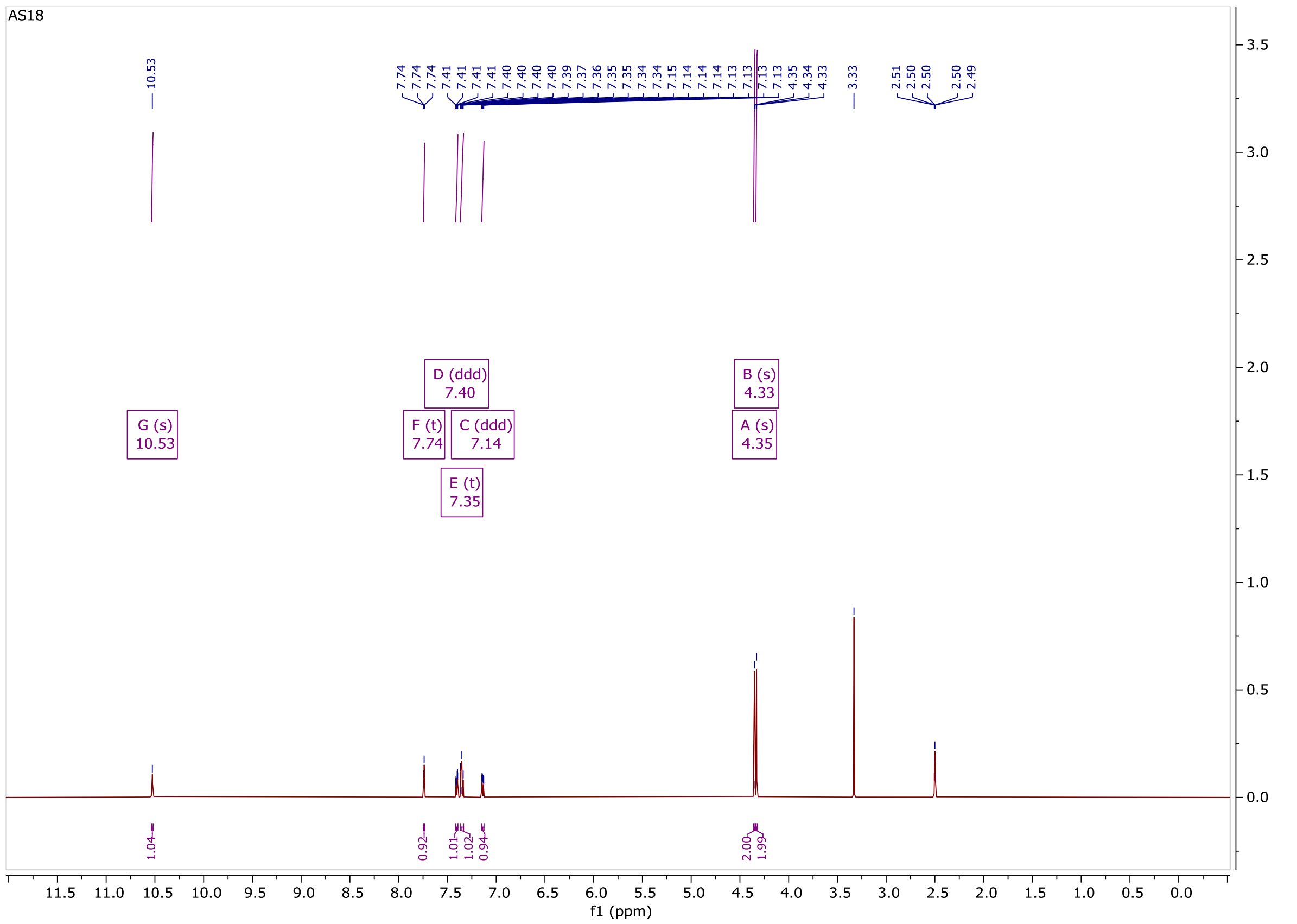

^{13}C NMR *N-(3-Chlorophenyl)-2-(2,4-dioxothiazolidin-3-yl)acetamide* (**2b**)

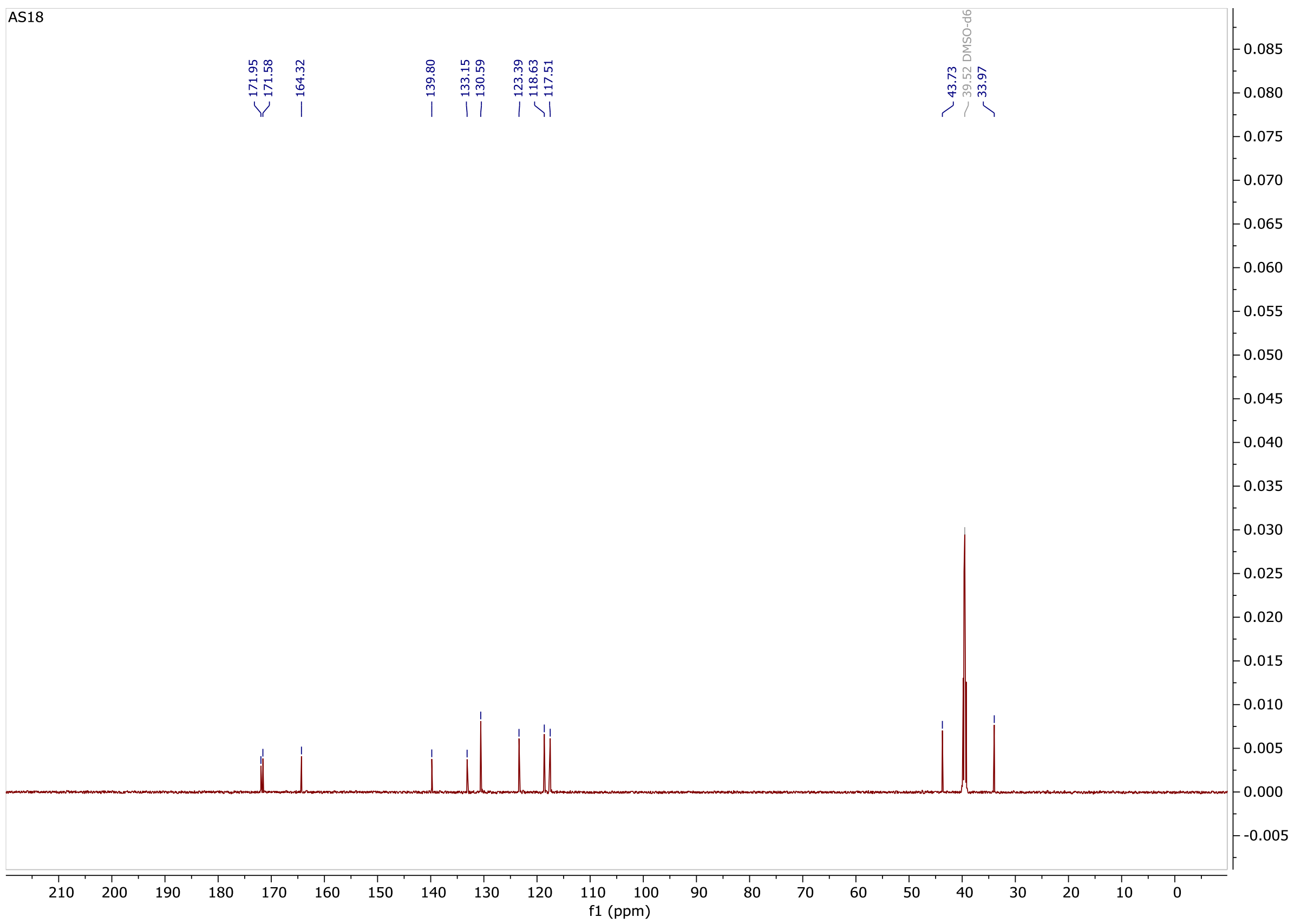

^{1}H NMR *N-(2-Chlorophenyl)-2-(4-oxo-2-thioxothiazolidin-3-yl)acetamide* (**1c**)

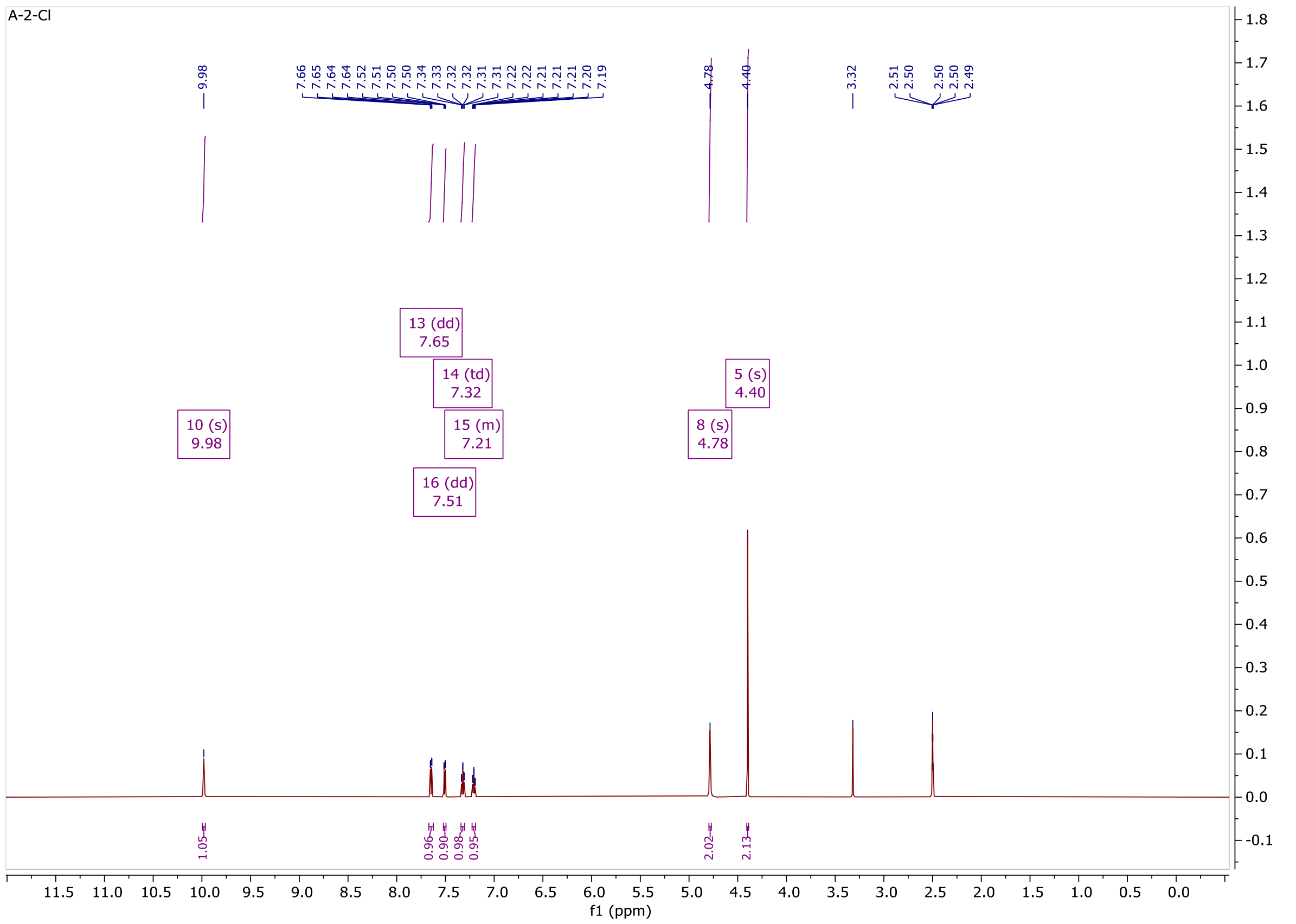

^{13}C NMR *N-(2-Chlorophenyl)-2-(4-oxo-2-thioxothiazolidin-3-yl)acetamide* (**1c**)

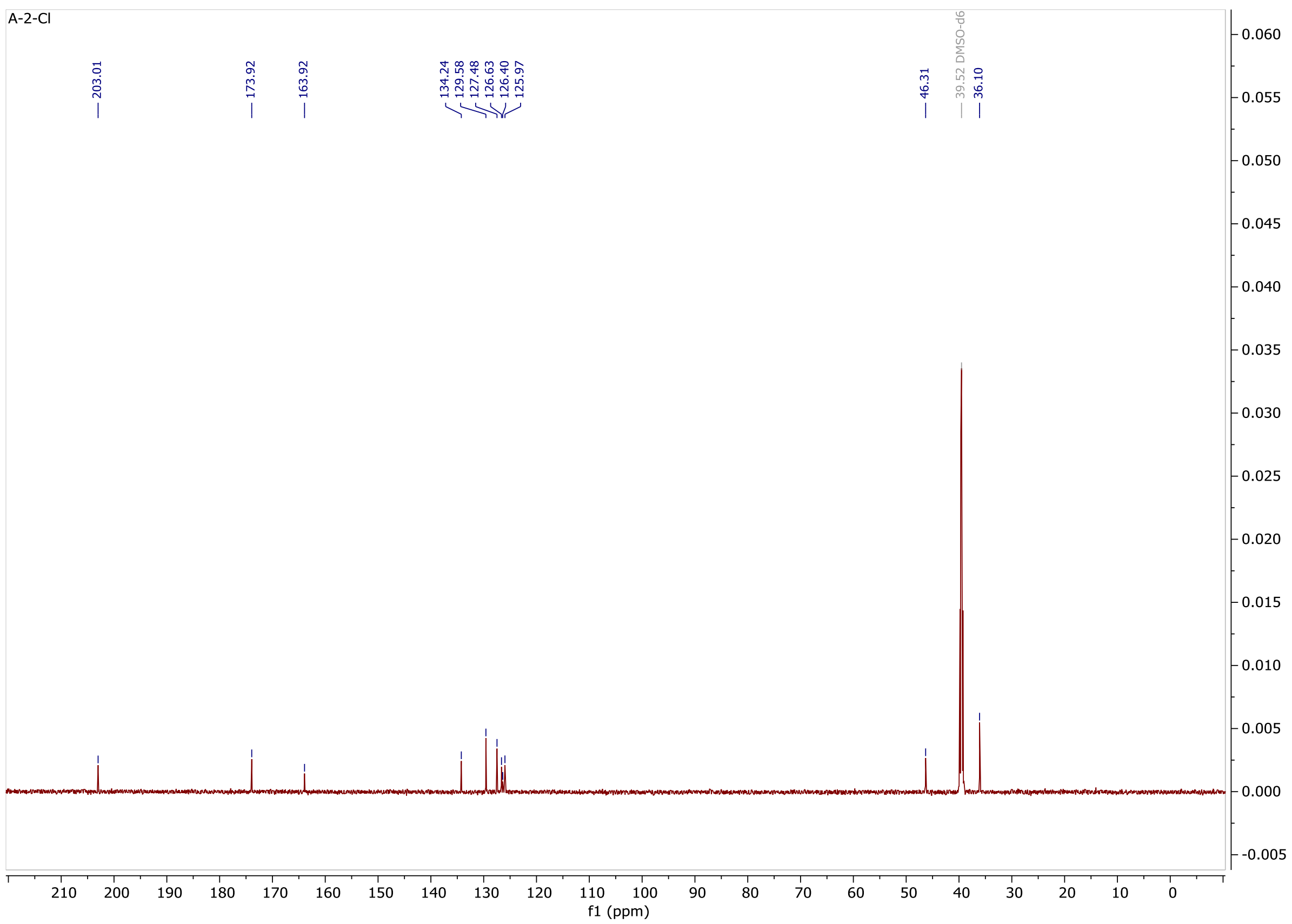

^{1}H NMR *N-(2-Chlorophenyl)-2-(2,4-dioxothiazolidin-3-yl)acetamide* (**2c**)

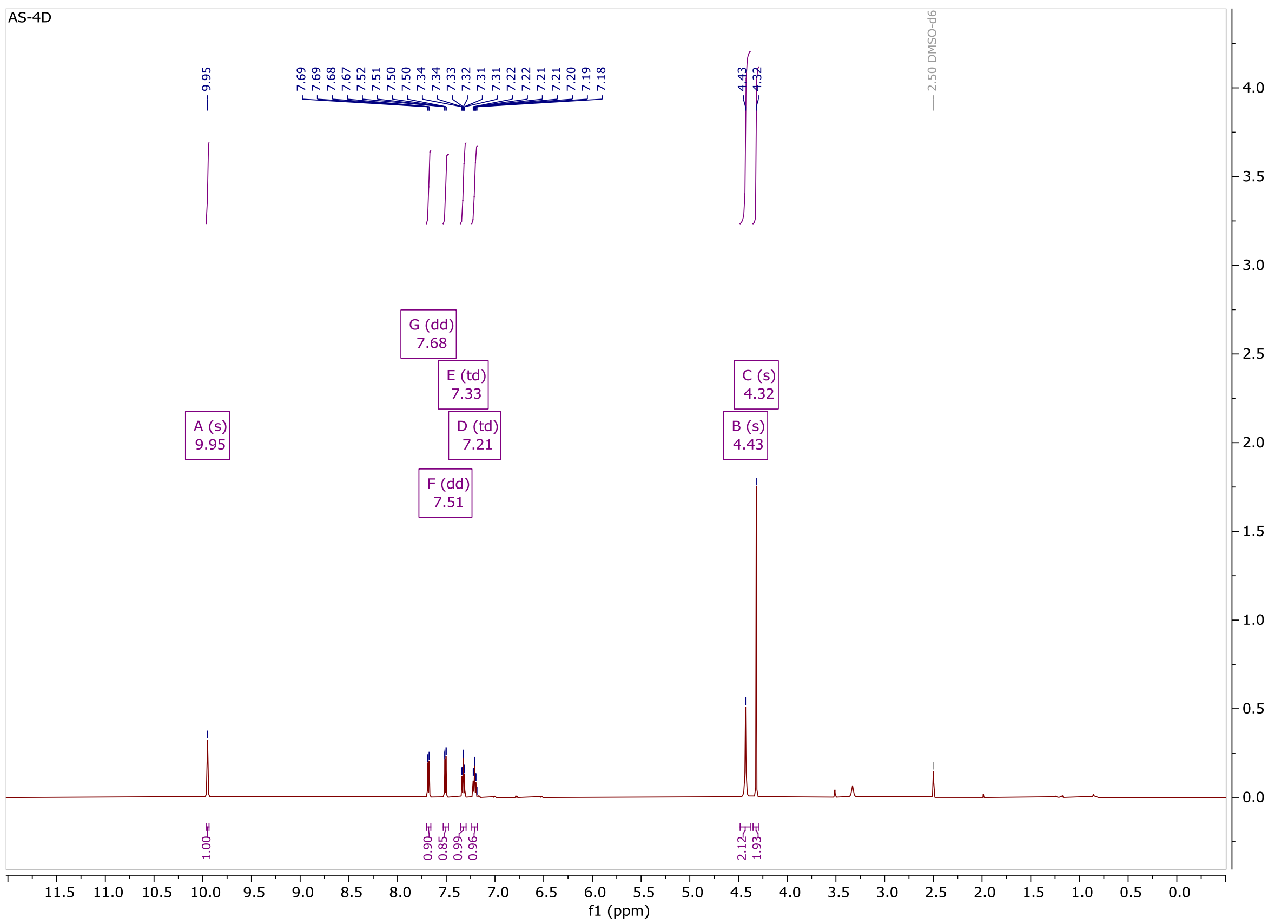

^{13}C NMR *N-(2-Chlorophenyl)-2-(2,4-dioxothiazolidin-3-yl)acetamide* (**2c**)

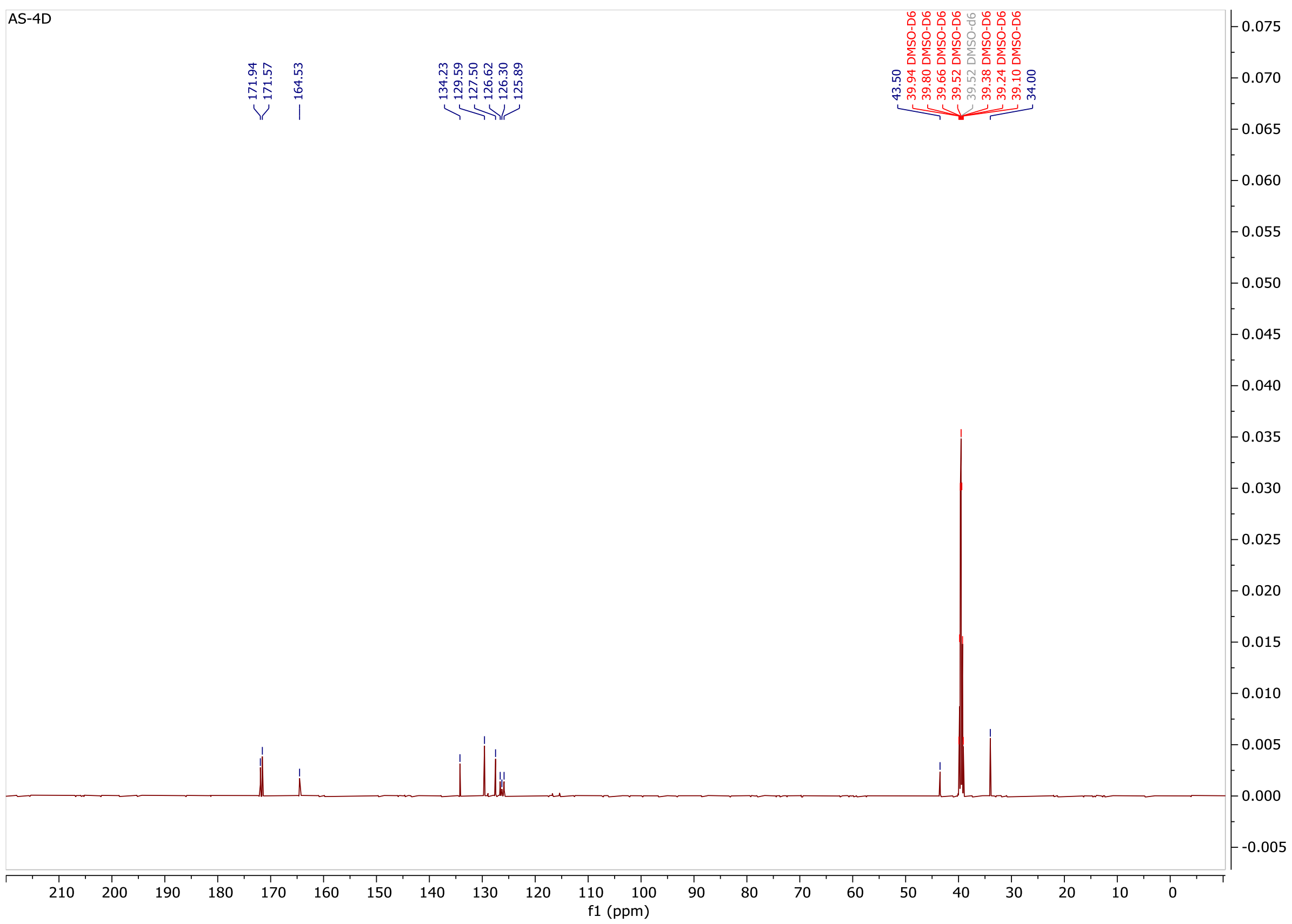

^{1}H NMR *N-(3,4-Dichlorophenyl)-2-(4-oxo-2-thioxothiazolidin-3-yl)acetamide* (**1d**)

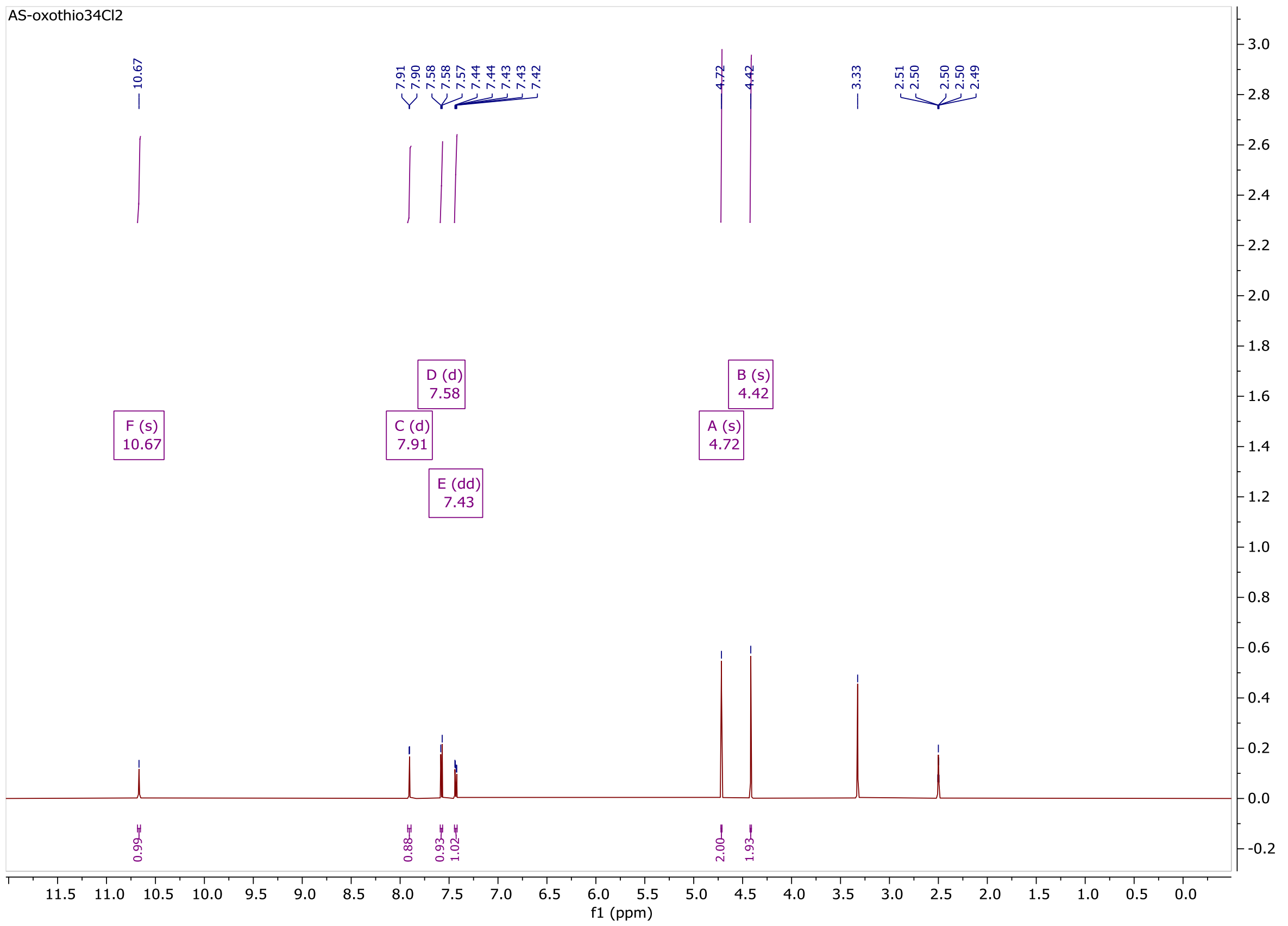

^{13}C NMR *N-(3,4-Dichlorophenyl)-2-(4-oxo-2-thioxothiazolidin-3-yl)acetamide* (**1d**)

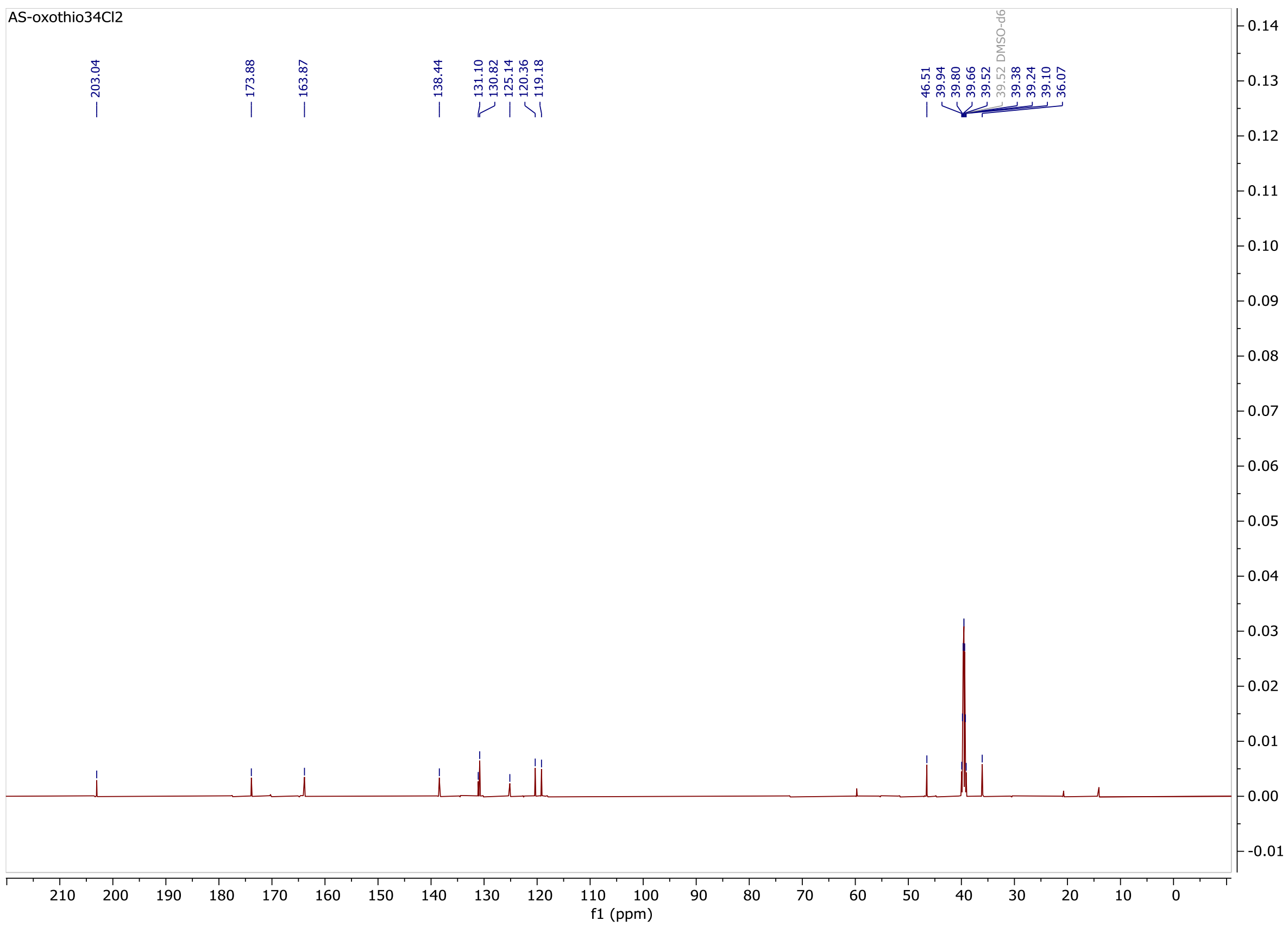

^{1}H NMR *N-(3,4-Dichlorophenyl)-2-(2,4-dioxothiazolidin-3-yl)acetamide* (**2d**)

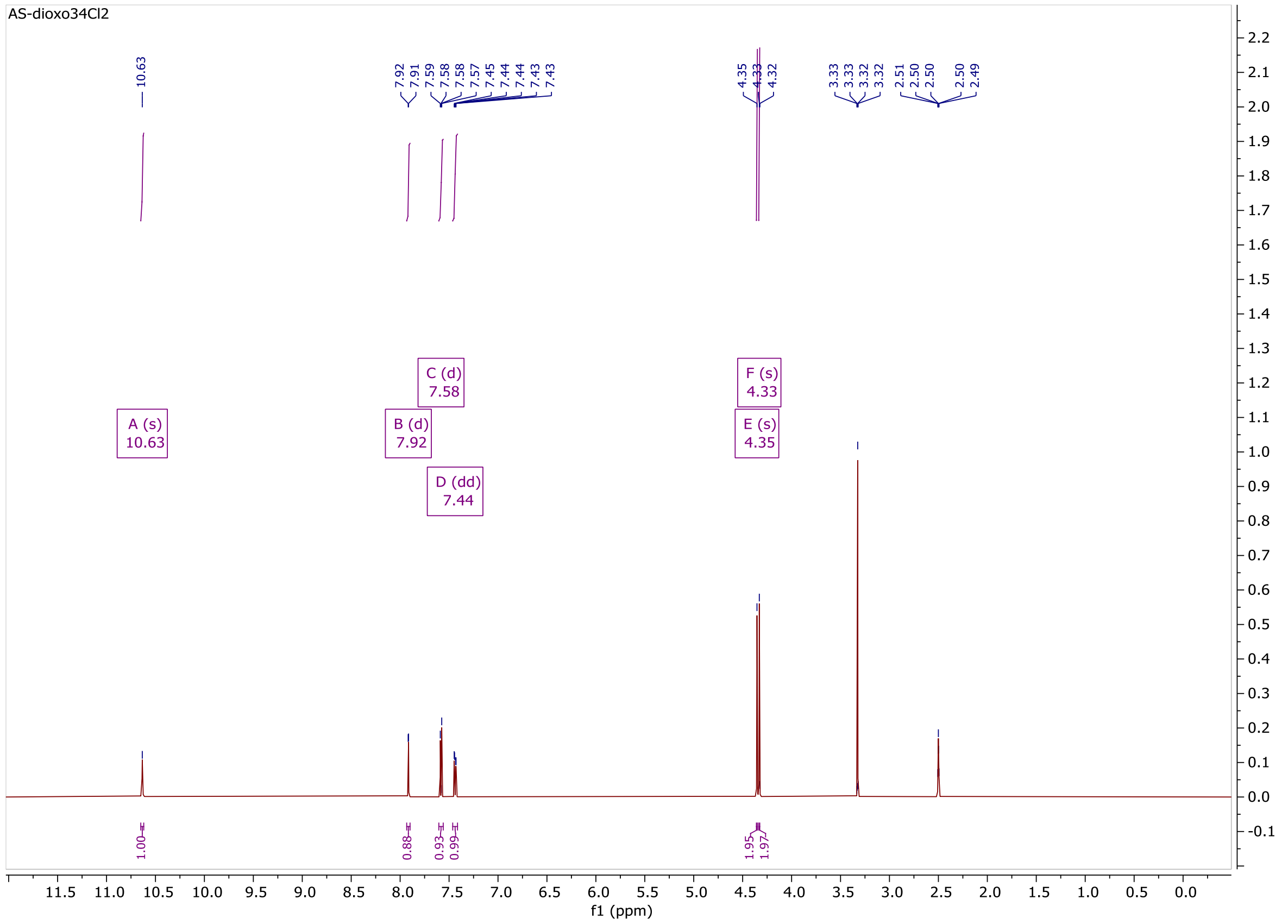

^{13}C NMR *N-(3,4-Dichlorophenyl)-2-(2,4-dioxothiazolidin-3-yl)acetamide* (**2d**)

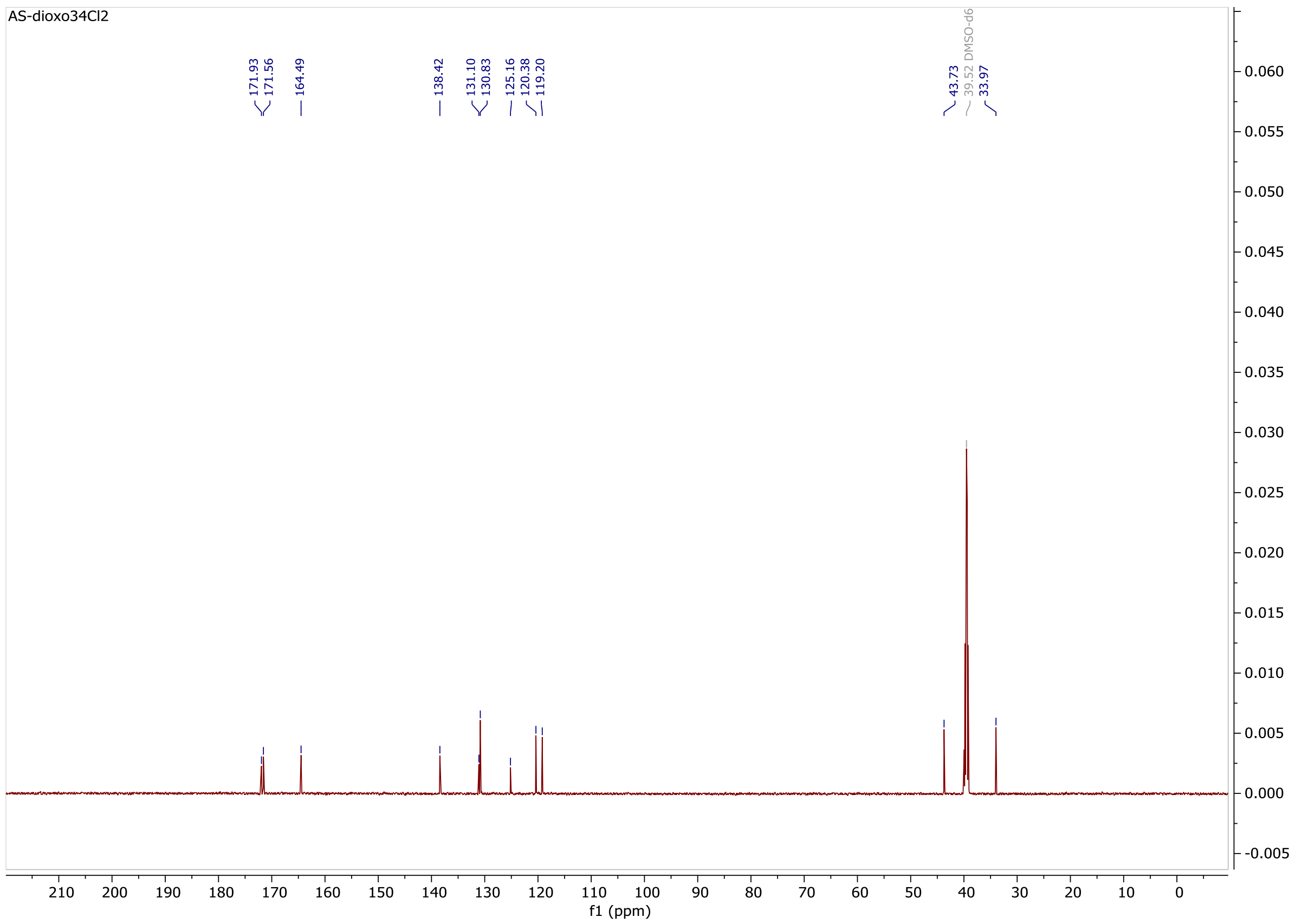

^{1}H NMR *N-(3,5-Dichlorophenyl)-2-(4-oxo-2-thioxothiazolidin-3-yl)acetamide* (**1e**)

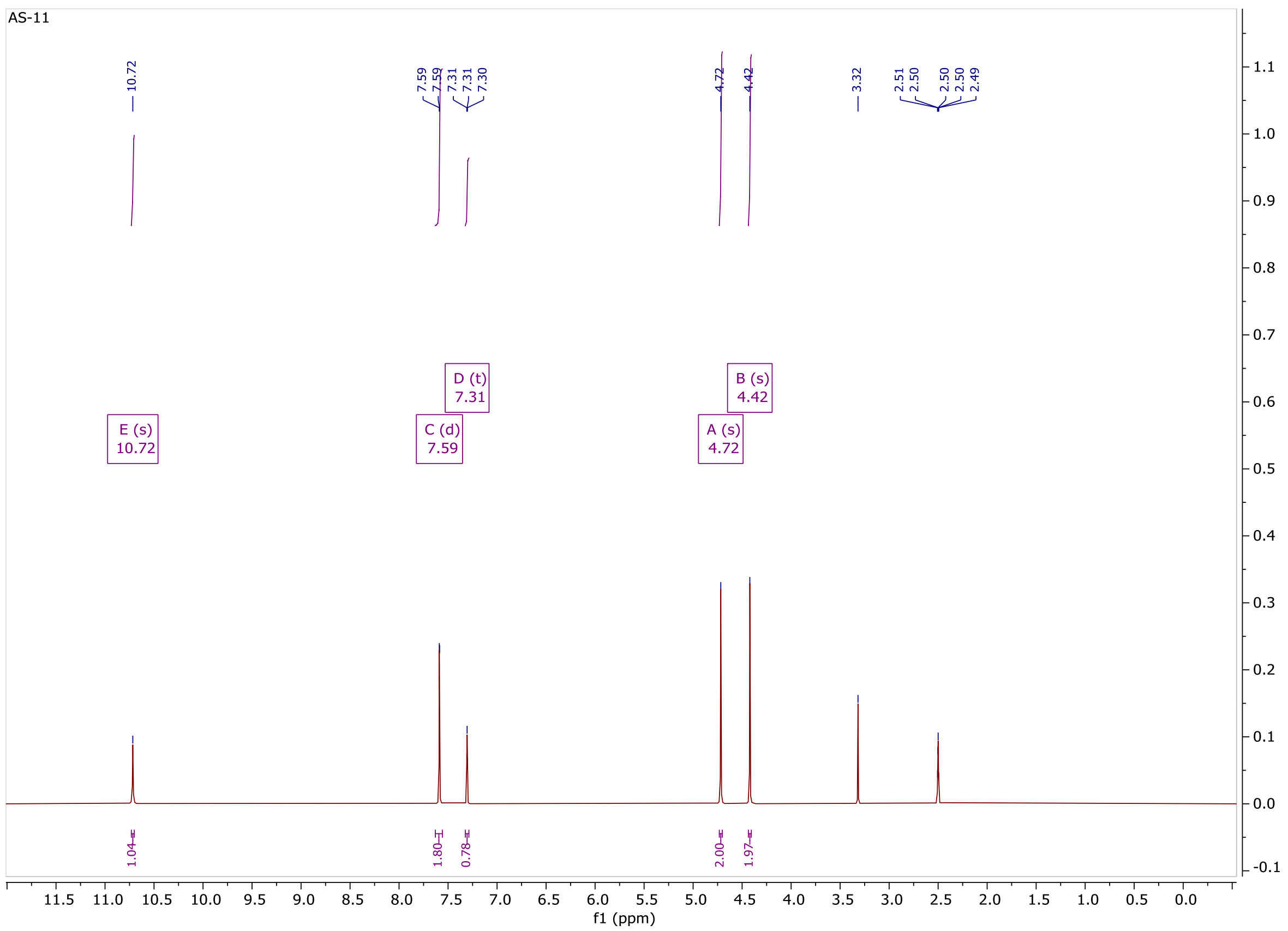

^{13}C NMR *N-(3,5-Dichlorophenyl)-2-(4-oxo-2-thioxothiazolidin-3-yl)acetamide* (**1e**)

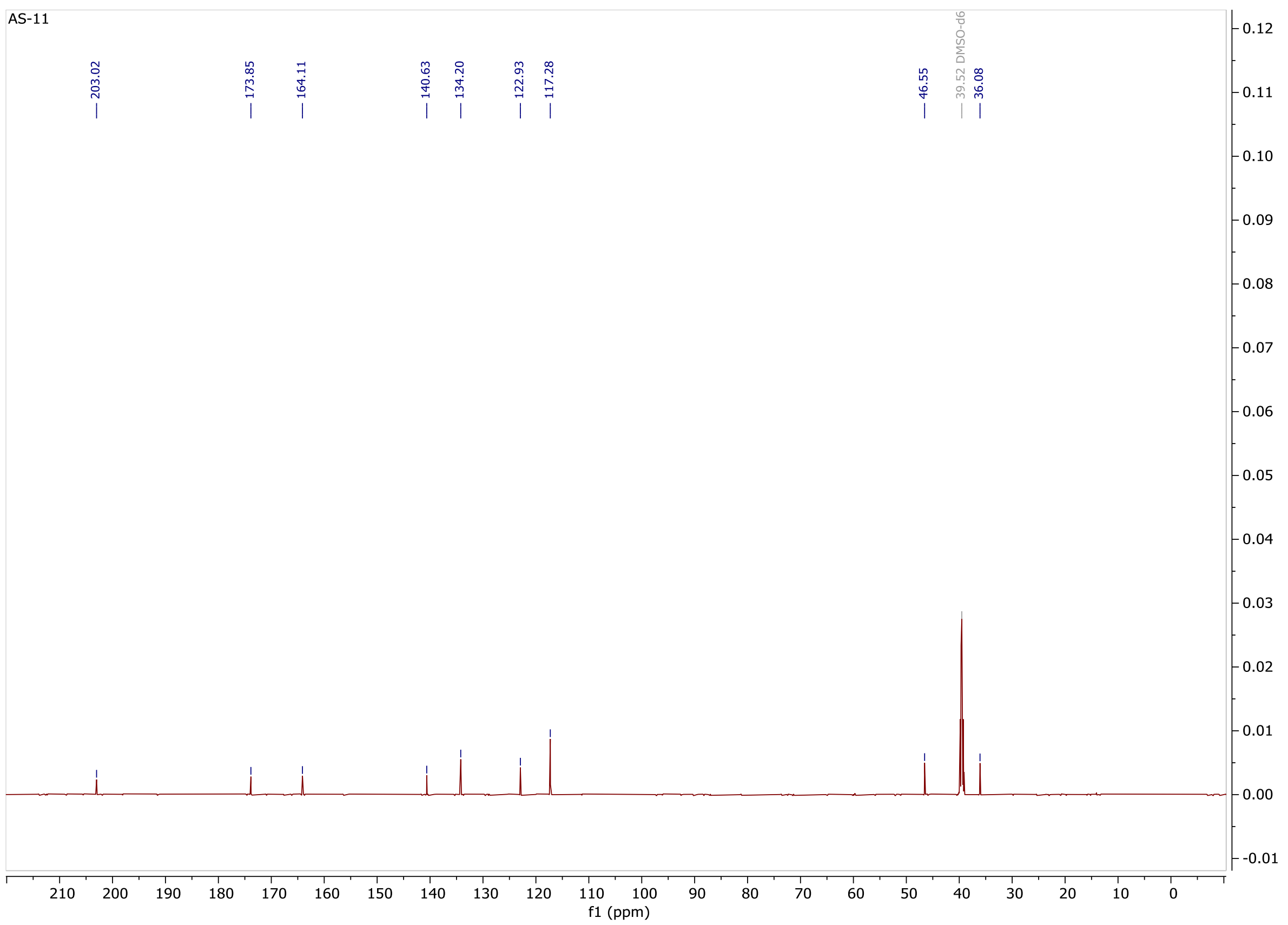

^{1}H NMR *N-(3,5-Dichlorophenyl)-2-(2,4-dioxothiazolidin-3-yl)acetamide* (**2e**)

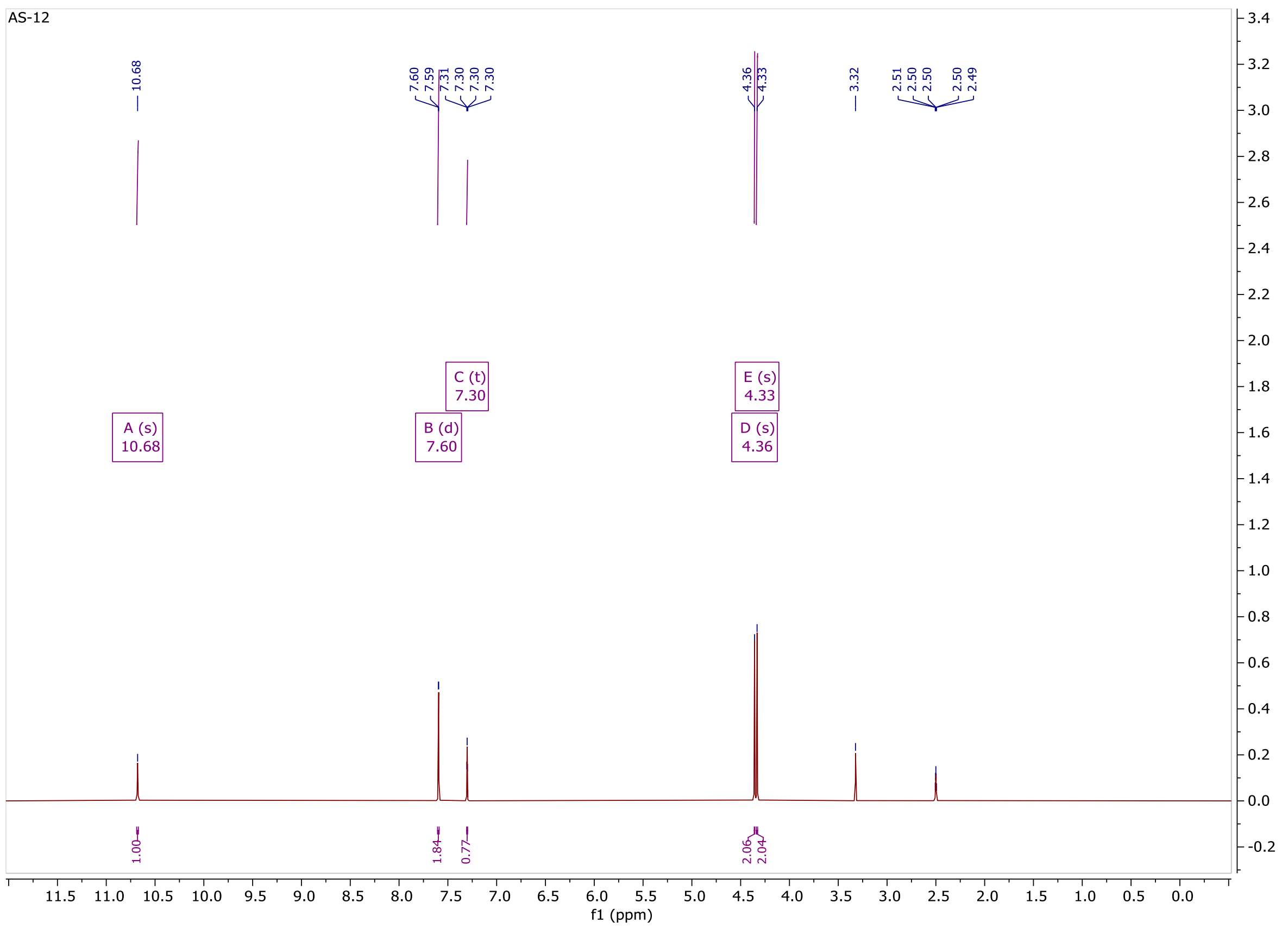

^{13}C NMR *N-(3,5-Dichlorophenyl)-2-(2,4-dioxothiazolidin-3-yl)acetamide* (**2e**)

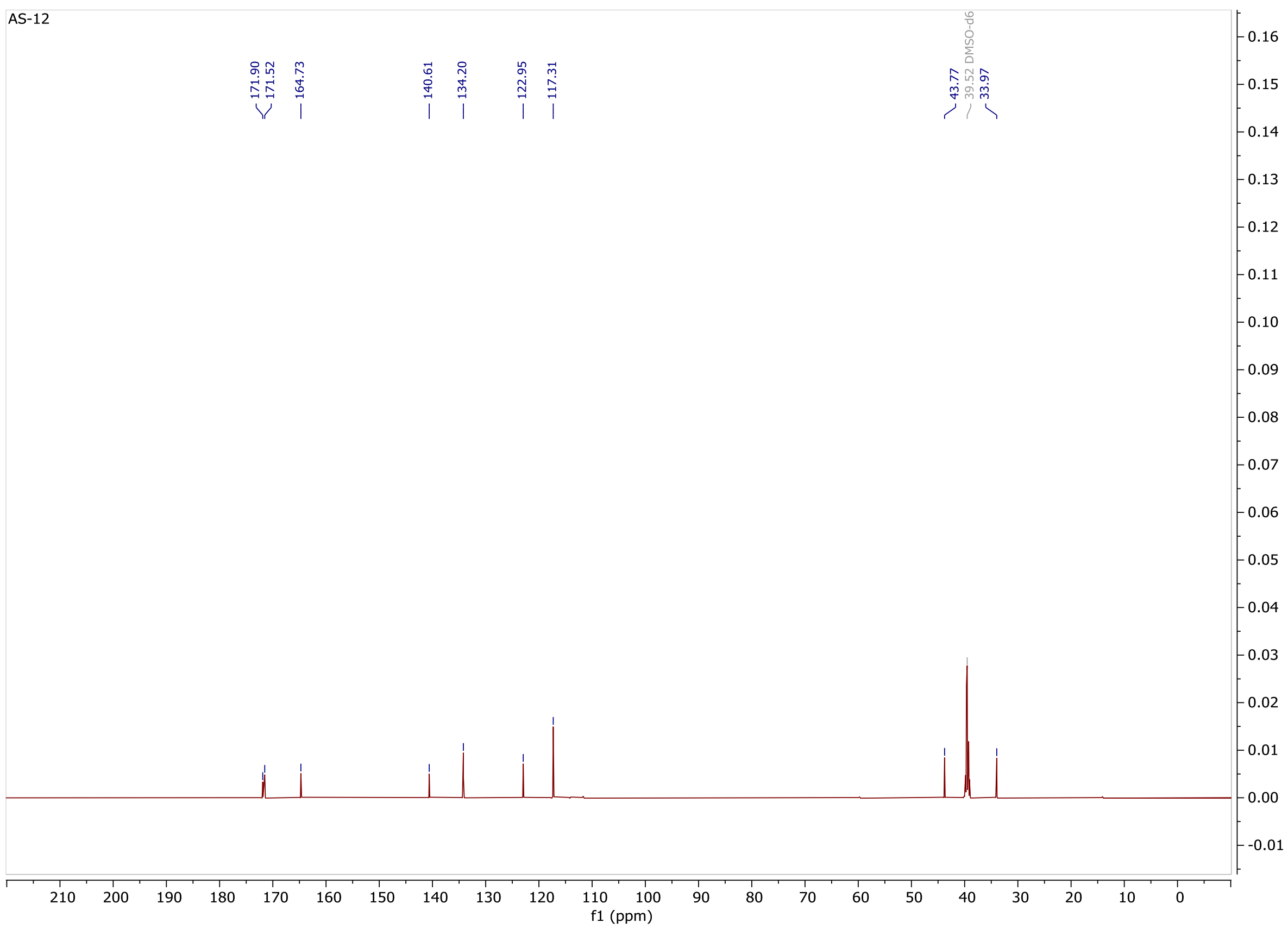

^{1}H NMR *N-(2,6-Dichlorophenyl)-2-(4-oxo-2-thioxothiazolidin-3-yl)acetamide* (**1f**)

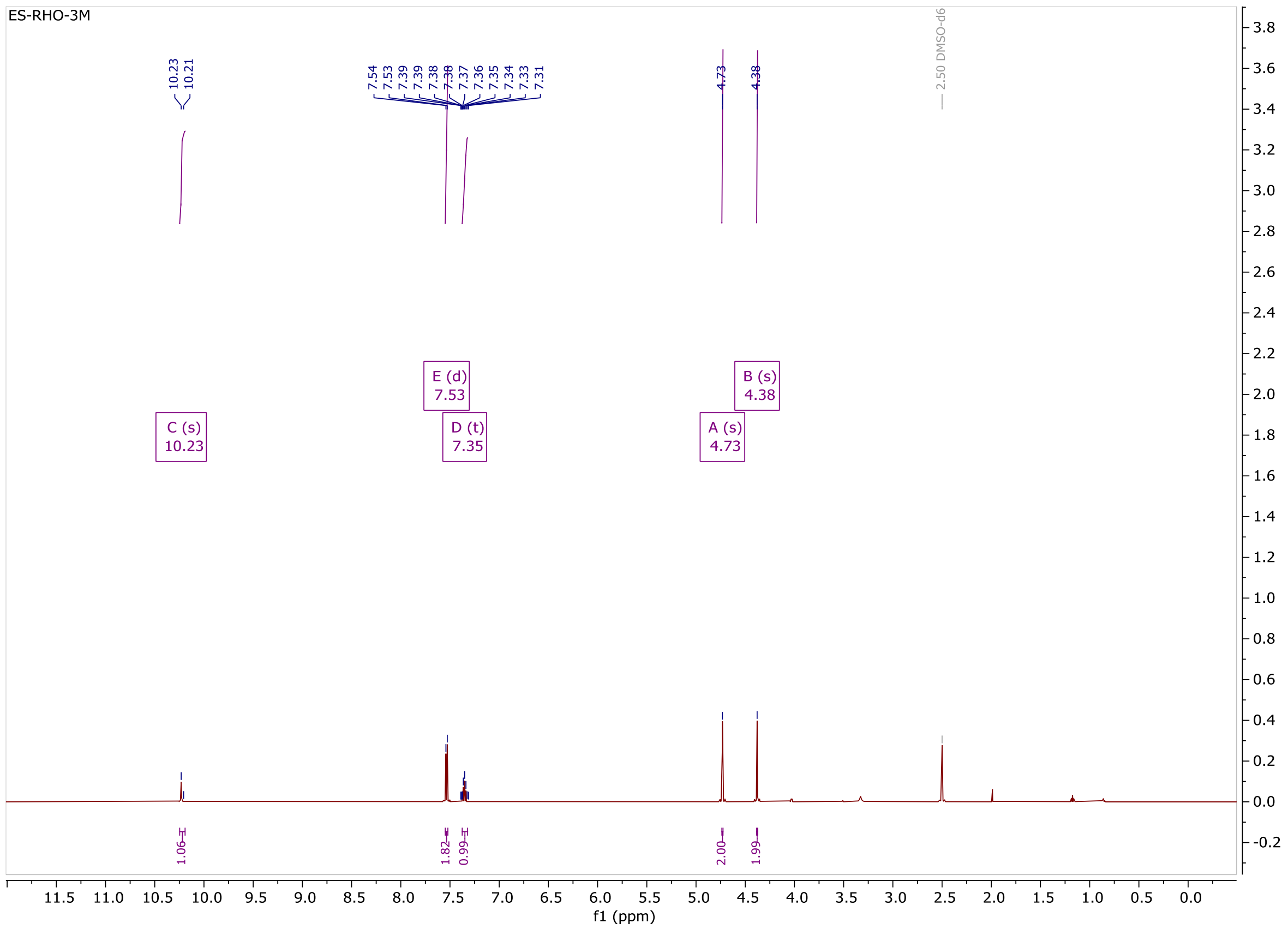

^{13}C NMR *N-(2,6-Dichlorophenyl)-2-(4-oxo-2-thioxothiazolidin-3-yl)acetamide* (**1f**)

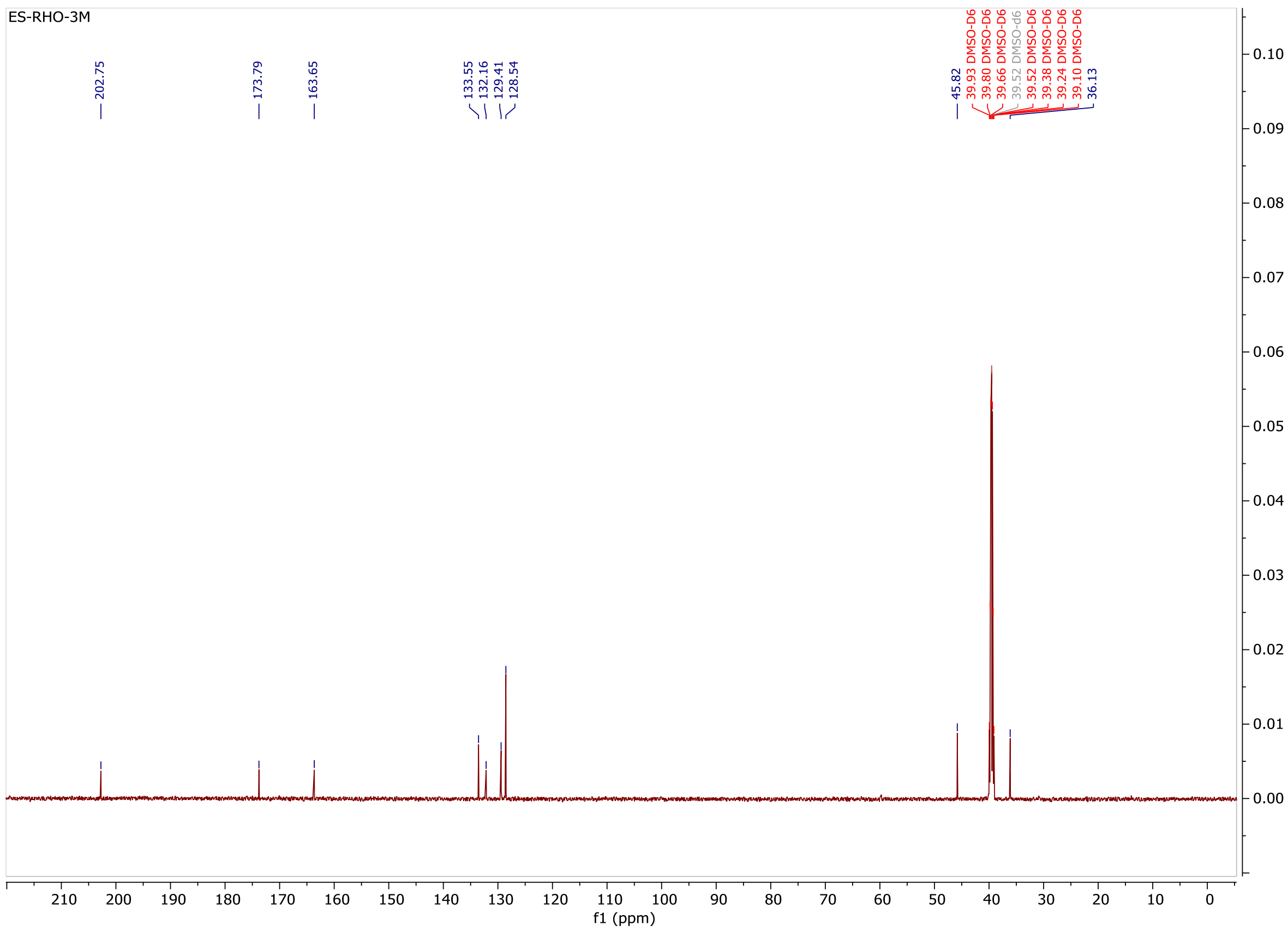

^{1}H NMR *N-(2,6-Dichlorophenyl)-2-(2,4-dioxothiazolidin-3-yl)acetamide* (**2f**)

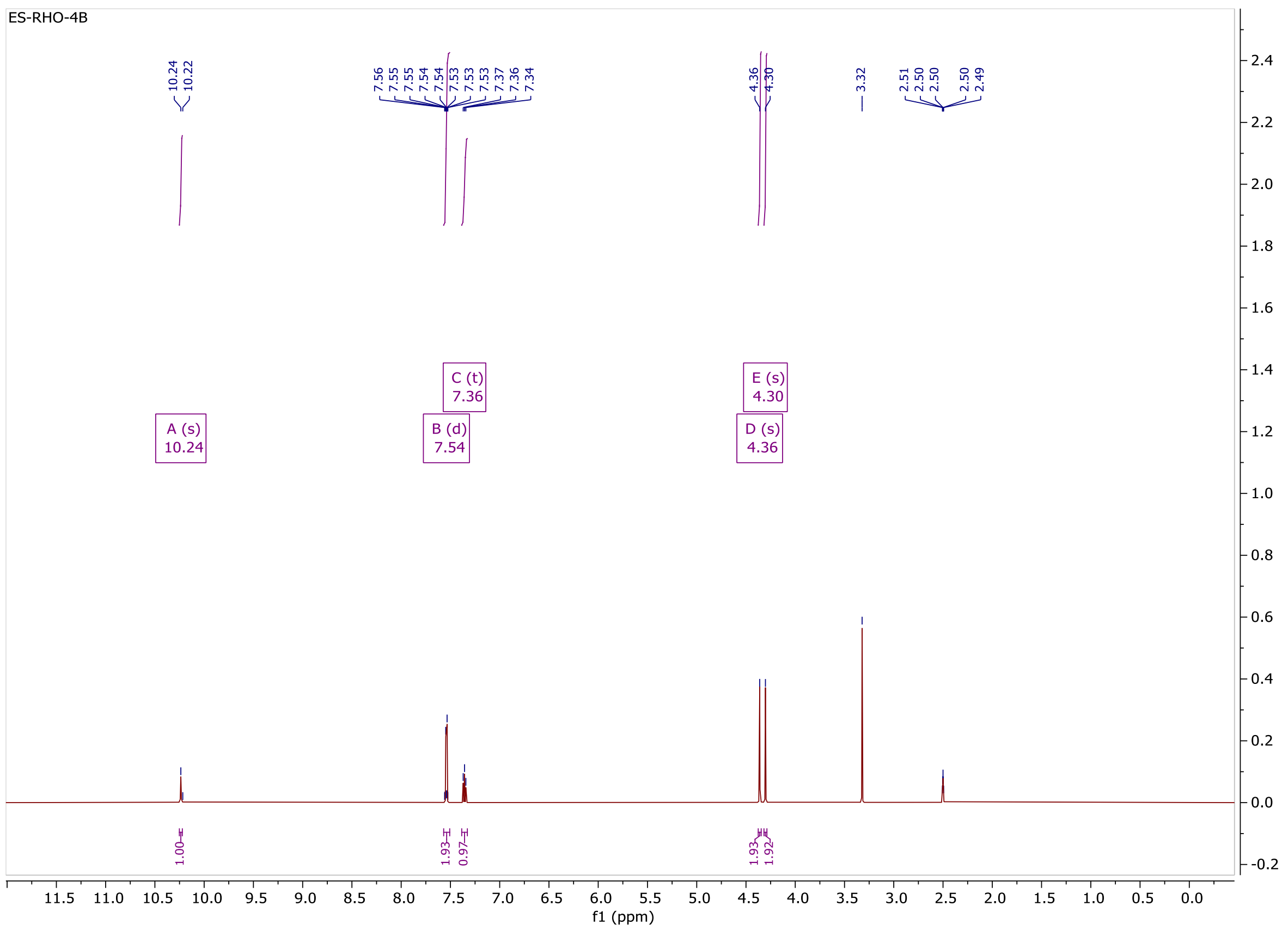

^{13}C NMR *N-(2,6-Dichlorophenyl)-2-(2,4-dioxothiazolidin-3-yl)acetamide* (**2f**)

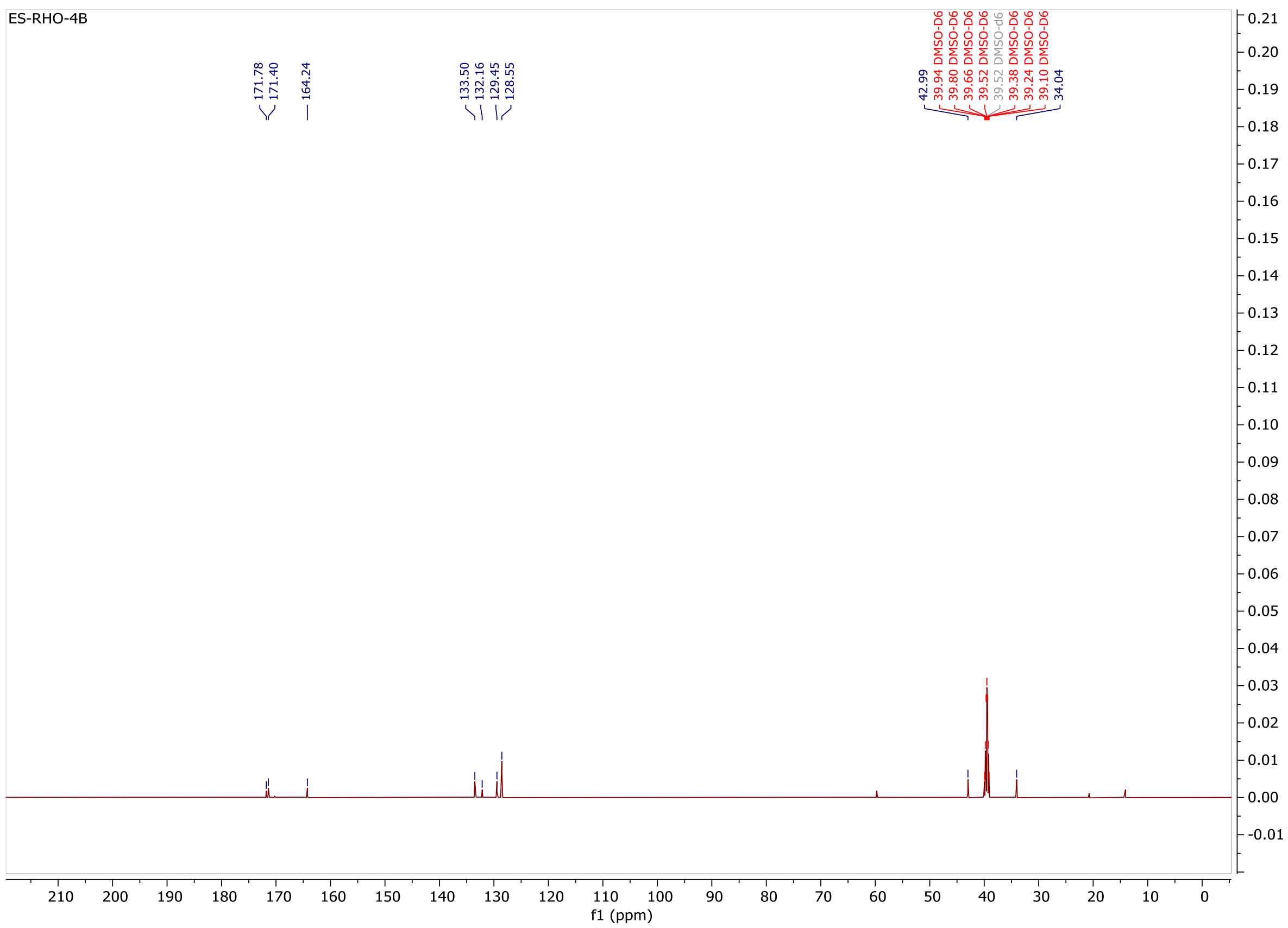

^{1}H NMR *N-(2,4-Dichlorophenyl)-2-(4-oxo-2-thioxothiazolidin-3-yl)acetamide* (**1g**)

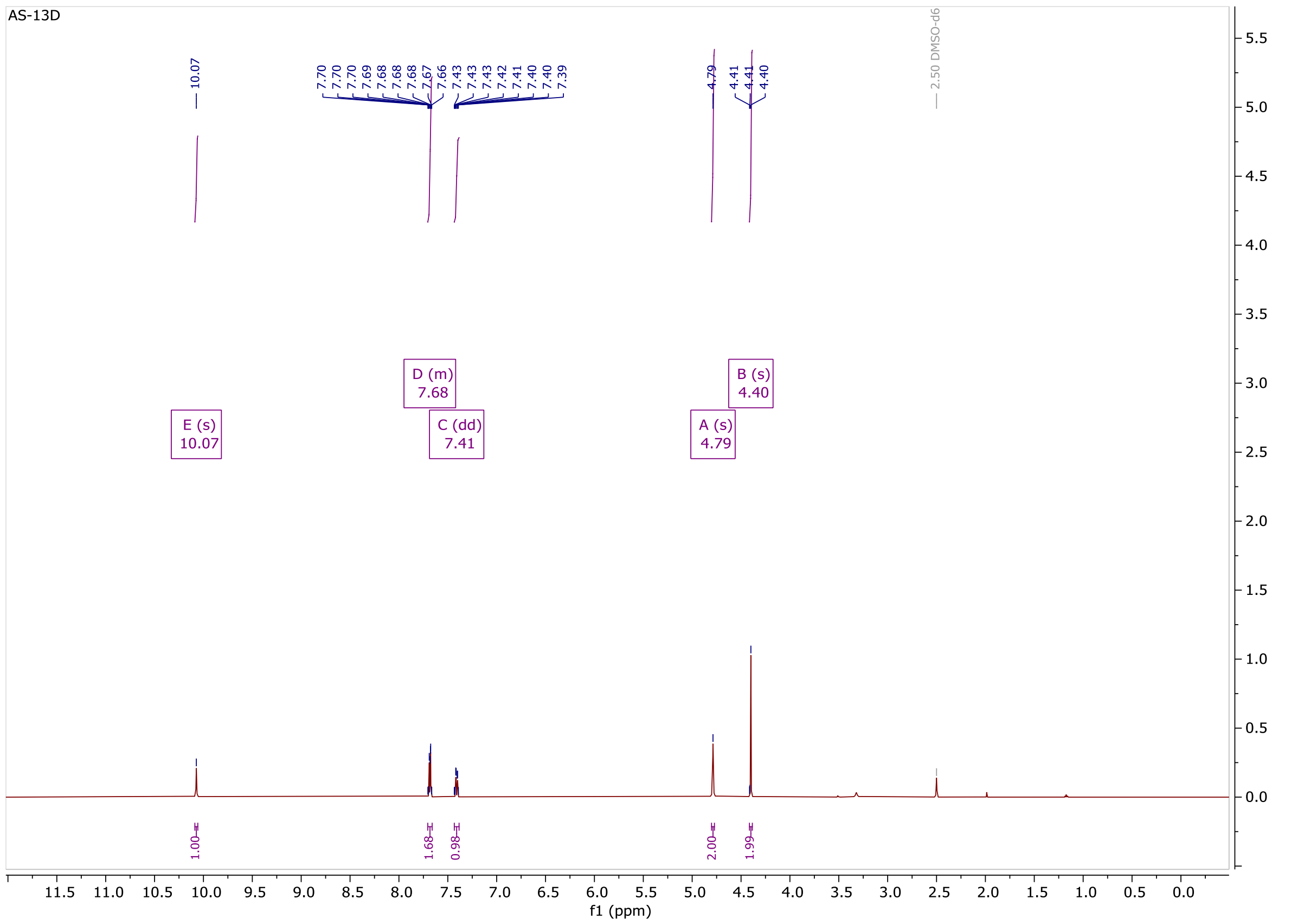

^{13}C NMR *N-(2,4-Dichlorophenyl)-2-(4-oxo-2-thioxothiazolidin-3-yl)acetamide* (**1g**)

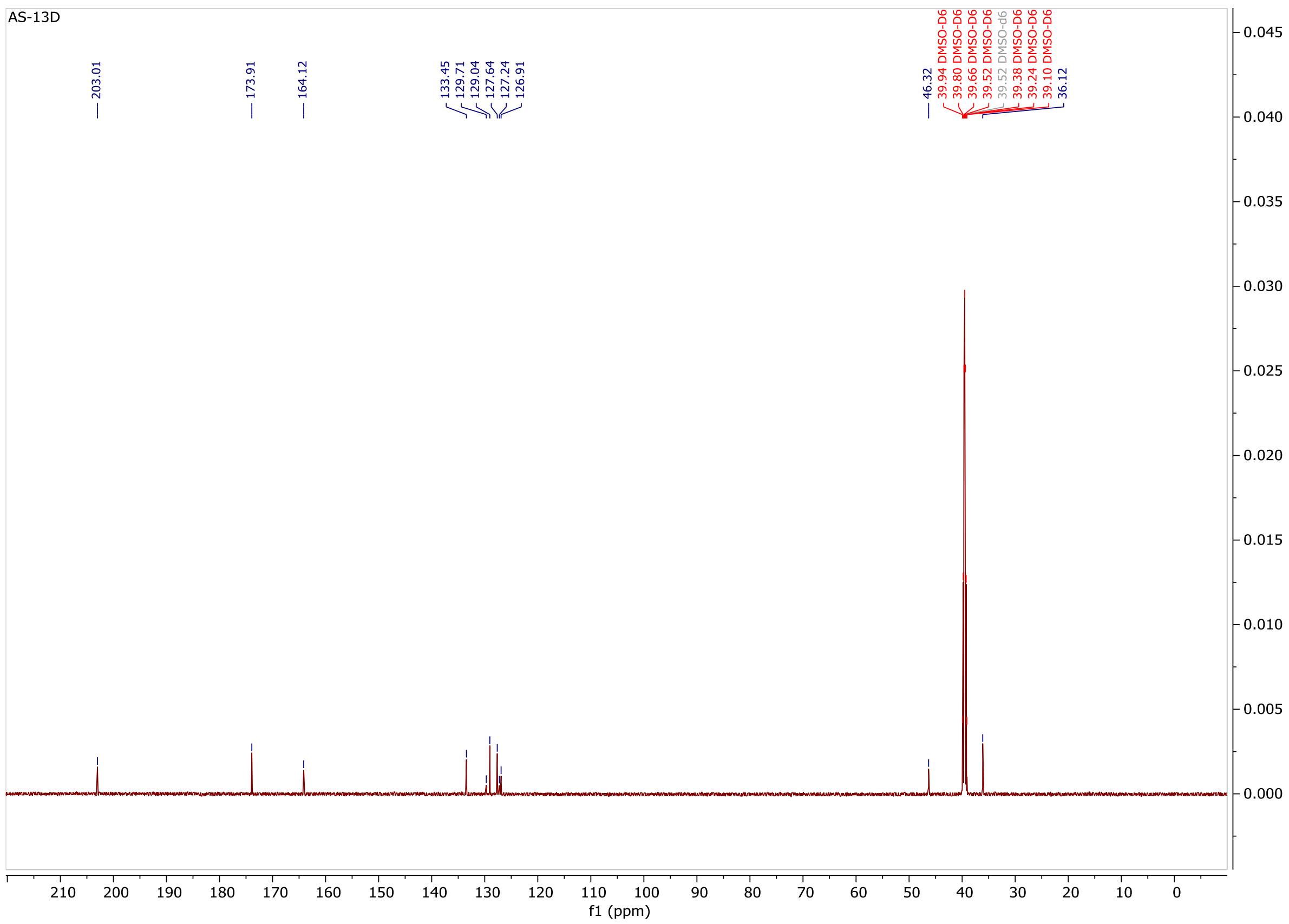

^{1}H NMR *N-(2,4-Dichlorophenyl)-2-(2,4-dioxothiazolidin-3-yl)acetamide* (**2g**)

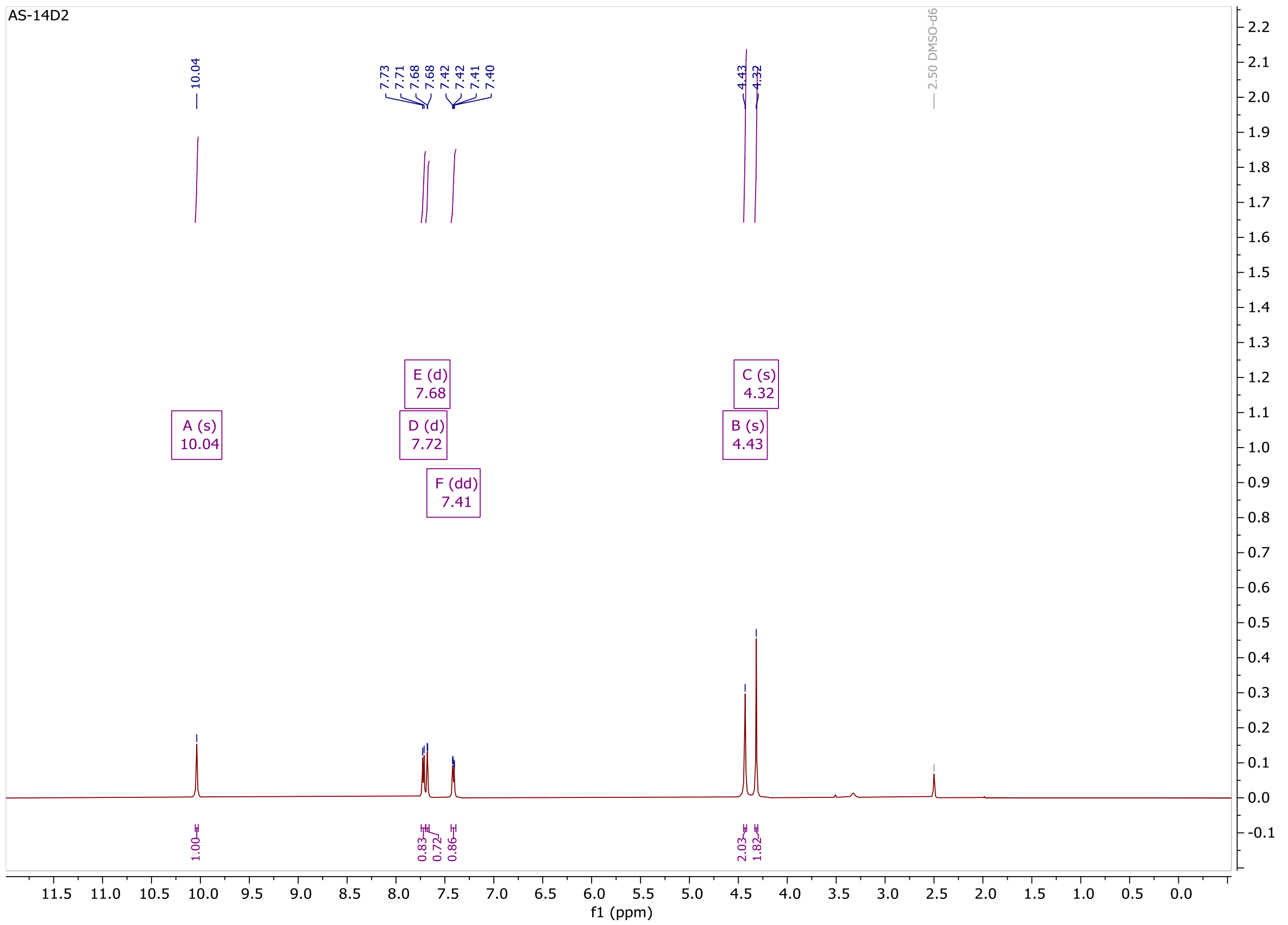

^{13}C NMR *N-(2,4-Dichlorophenyl)-2-(2,4-dioxothiazolidin-3-yl)acetamide* (**2g**)

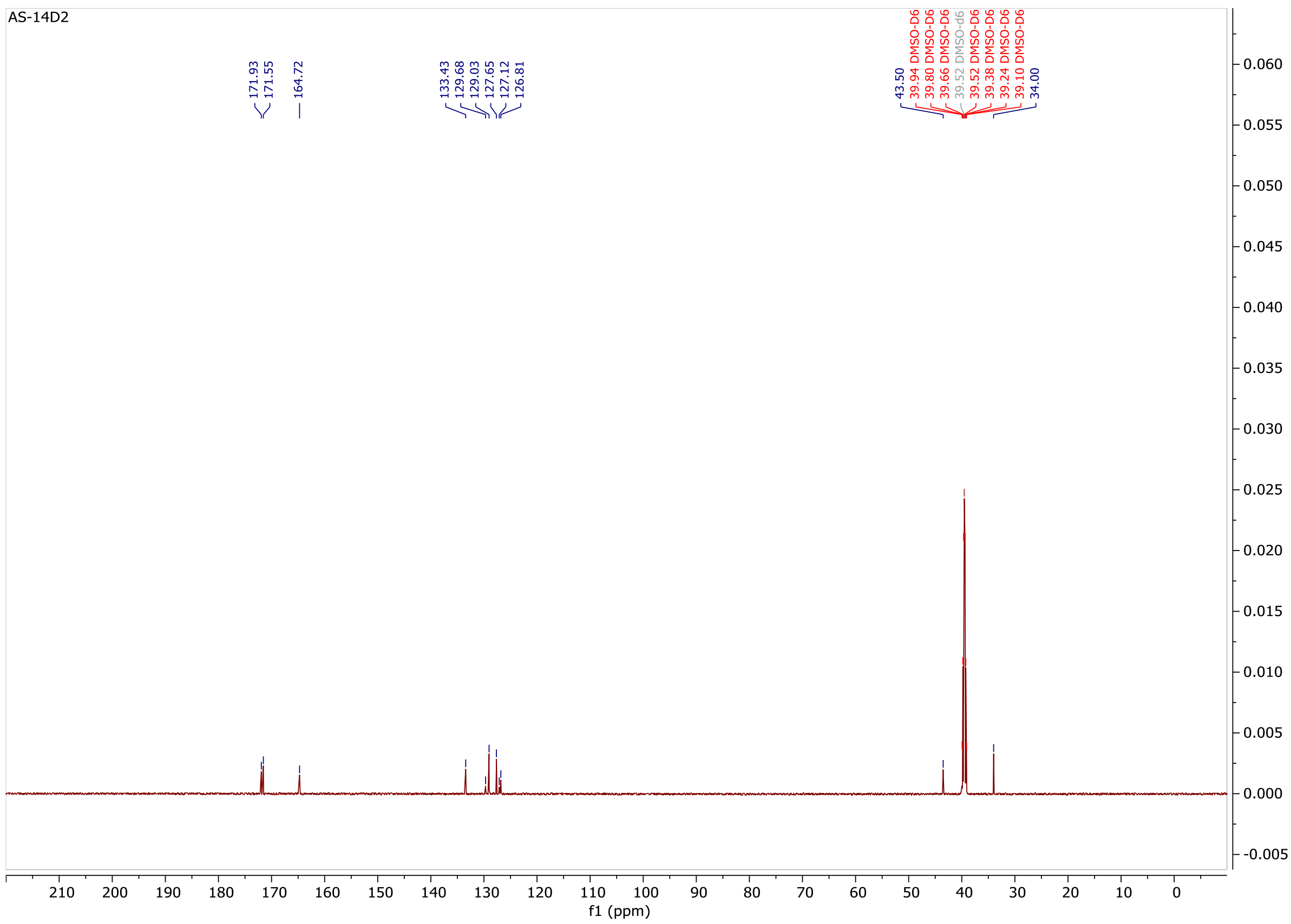

^{1}H NMR *2-(4-Oxo-2-thioxothiazolidin-3-yl)-N-(3,4,5-trichlorophenyl)acetamide* (**1h**)

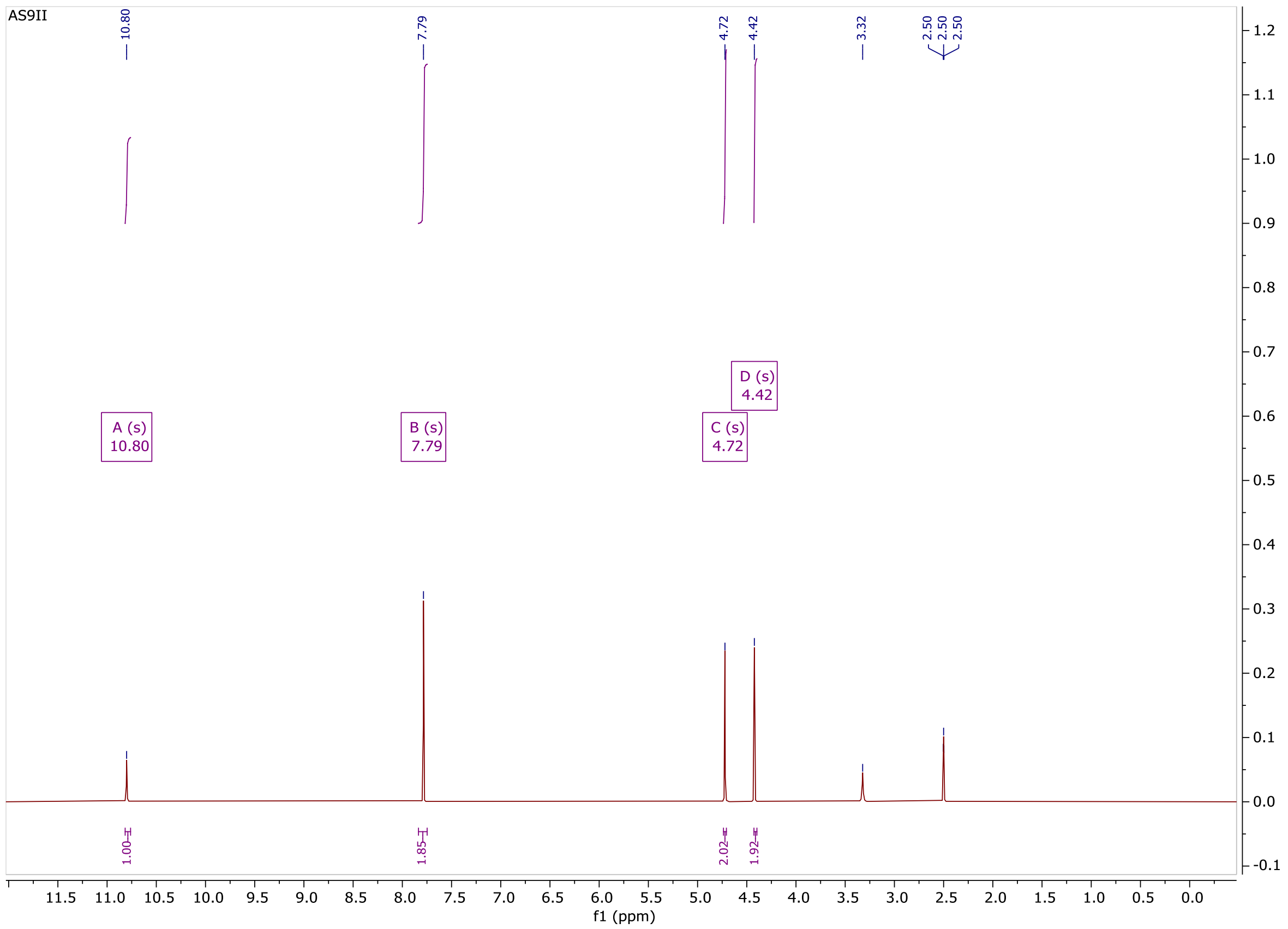

^{13}C NMR *2-(4-Oxo-2-thioxothiazolidin-3-yl)-N-(3,4,5-trichlorophenyl)acetamide* (**1h**)

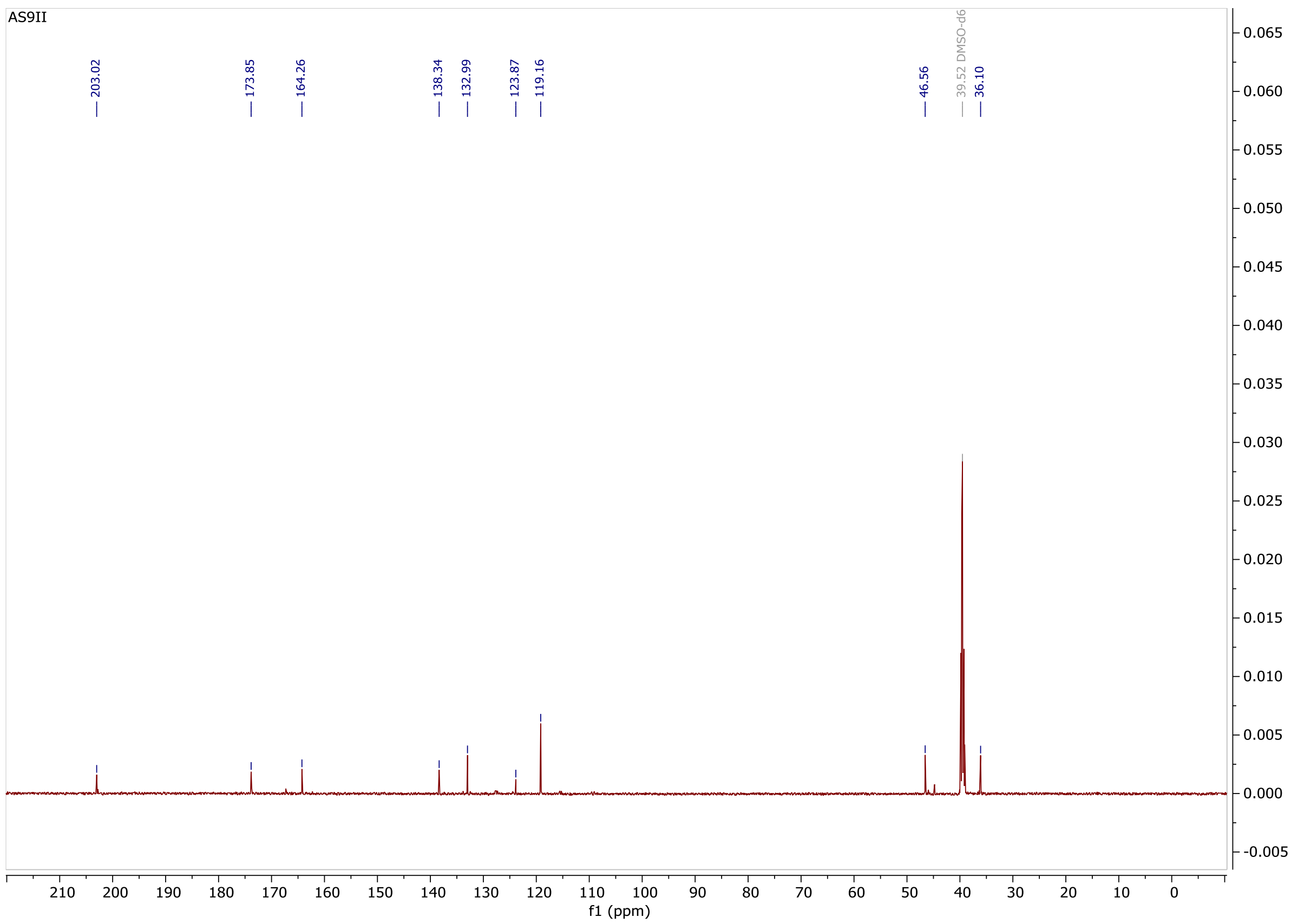

^{1}H NMR *2-(2,4-Dioxothiazolidin-3-yl)-N-(3,4,5-trichlorophenyl)acetamide* (**2h**)

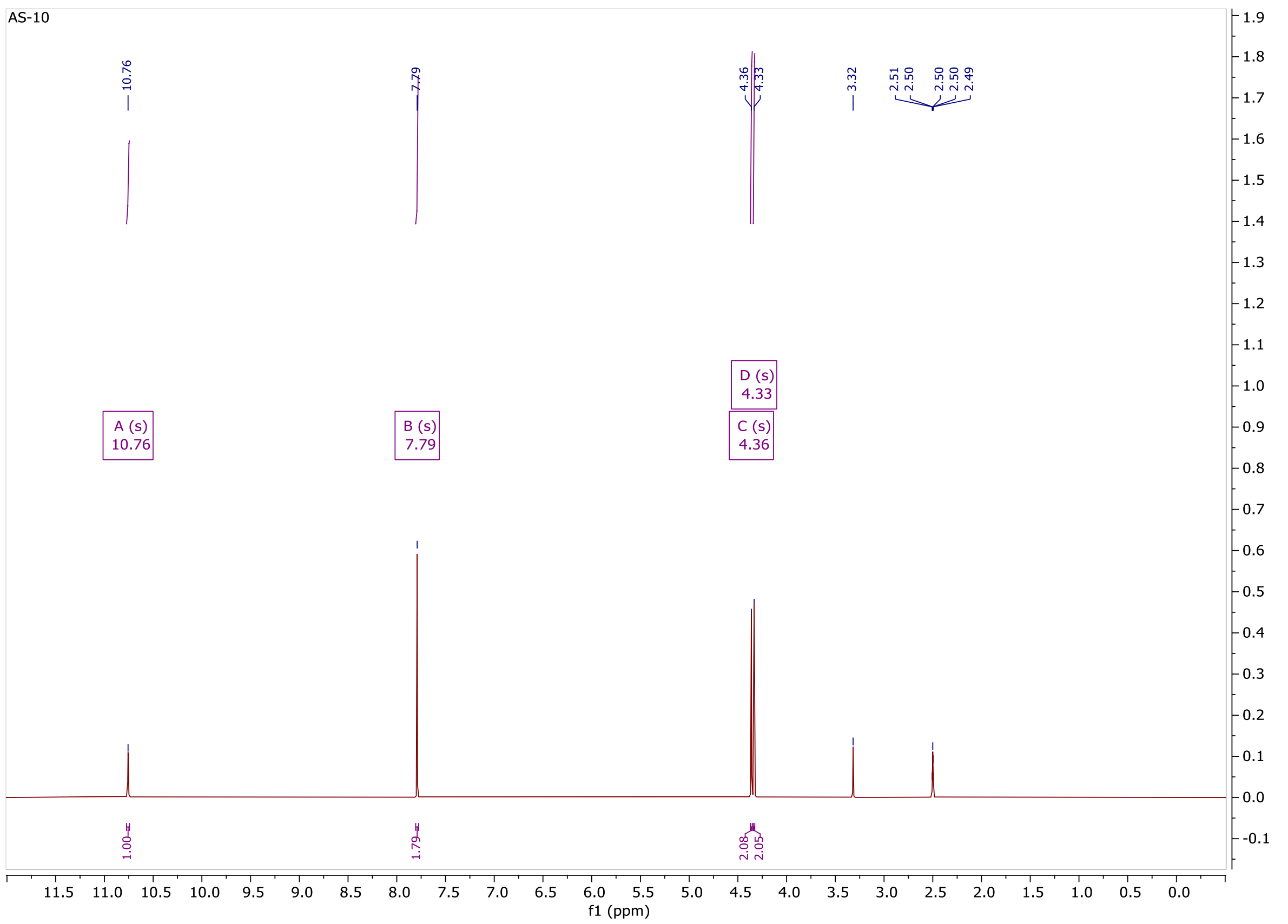

^{13}C NMR *2-(2,4-Dioxothiazolidin-3-yl)-N-(3,4,5-trichlorophenyl)acetamide* (**2h**)

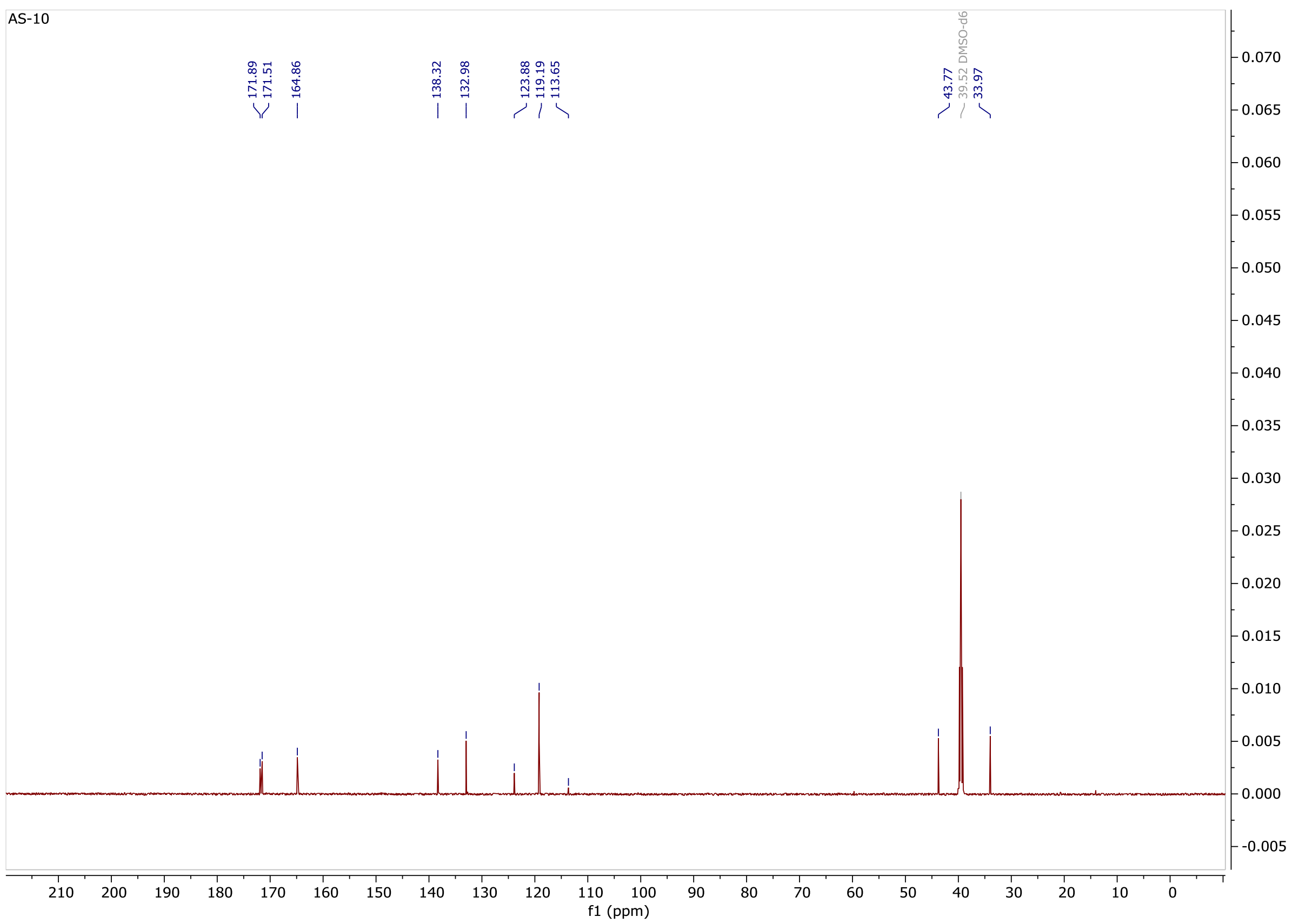

^{1}H NMR *2-(4-Oxo-2-thioxothiazolidin-3-yl)-N-(2,4,5-trichlorophenyl)acetamide* (**1i**)

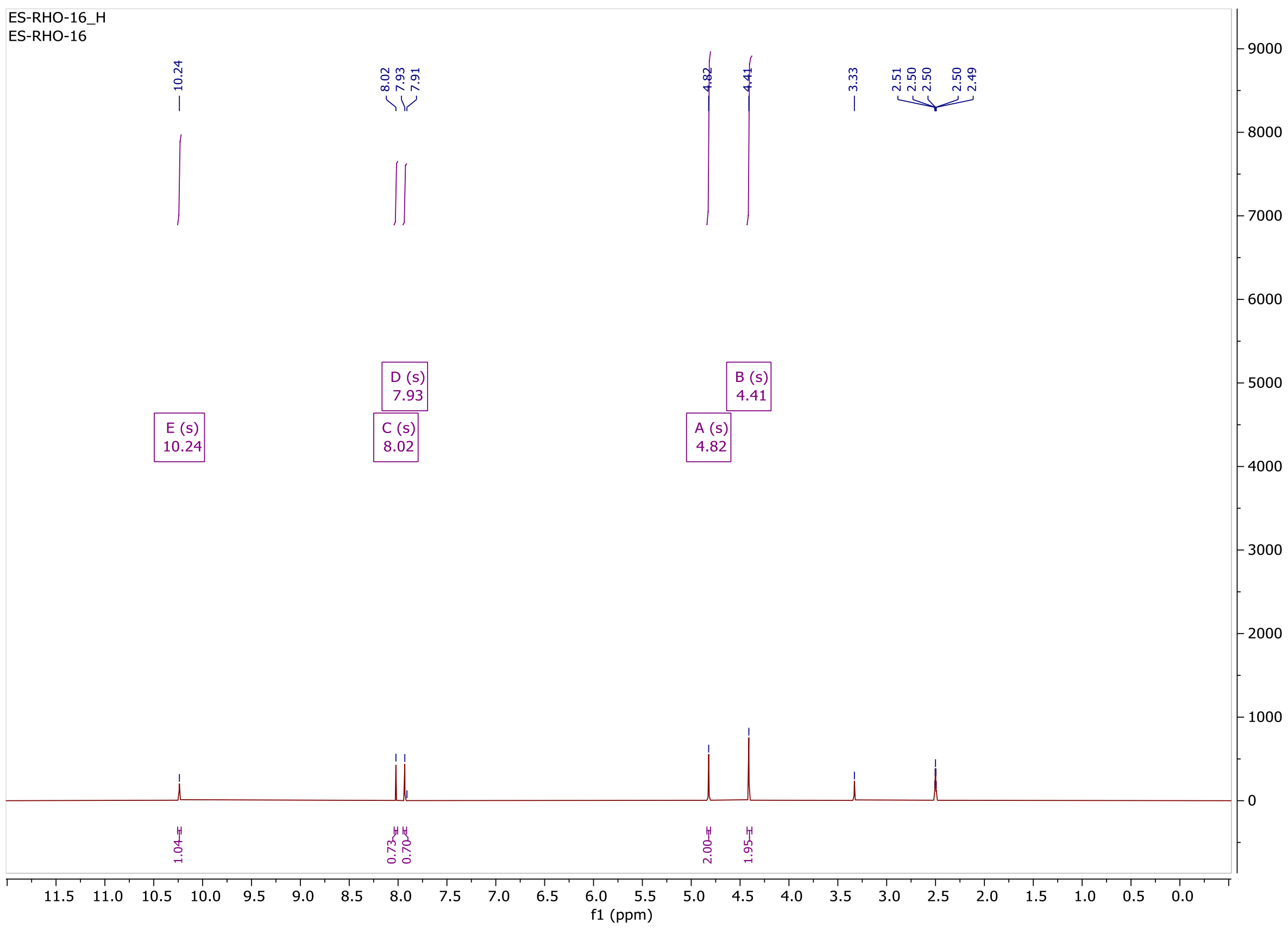

^{13}C NMR *2-(4-Oxo-2-thioxothiazolidin-3-yl)-N-(2,4,5-trichlorophenyl)acetamide* (**1i**)

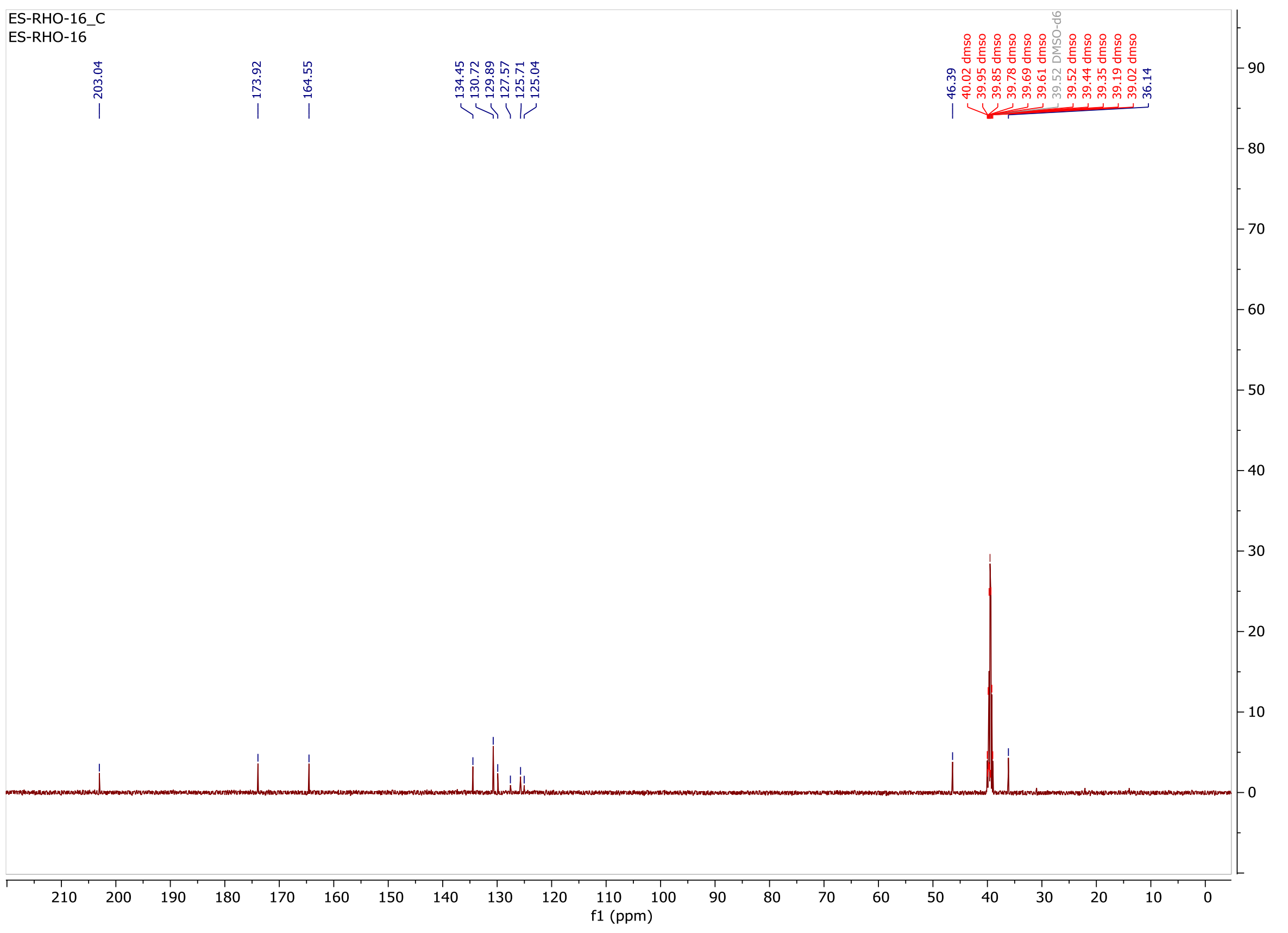

^{1}H NMR *2-(2,4-Dioxothiazolidin-3-yl)-N-(2,4,5-trichlorophenyl)acetamide* (**2i**)

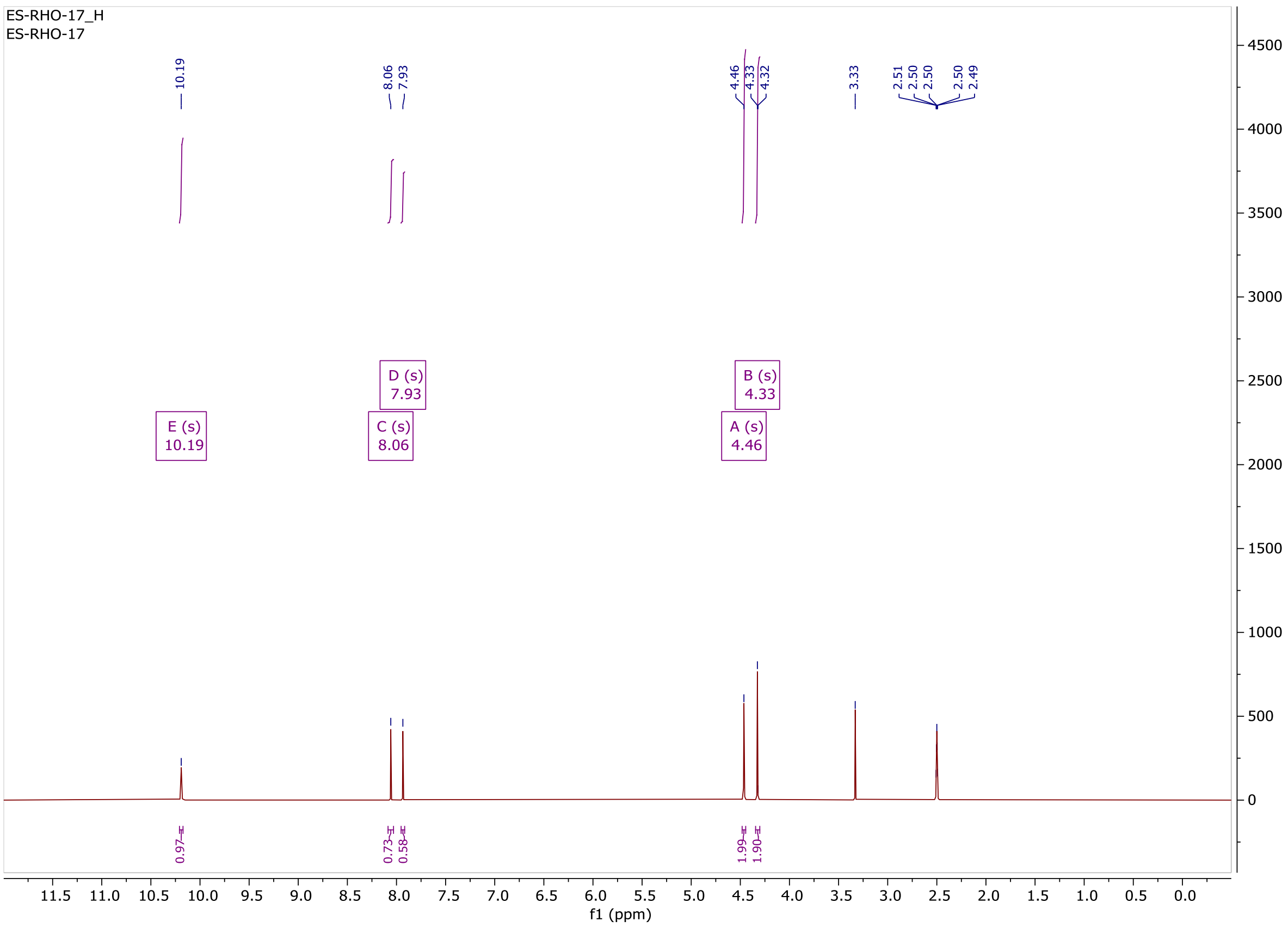

^{13}C NMR *2-(2,4-Dioxothiazolidin-3-yl)-N-(2,4,5-trichlorophenyl)acetamide* (**2i**)

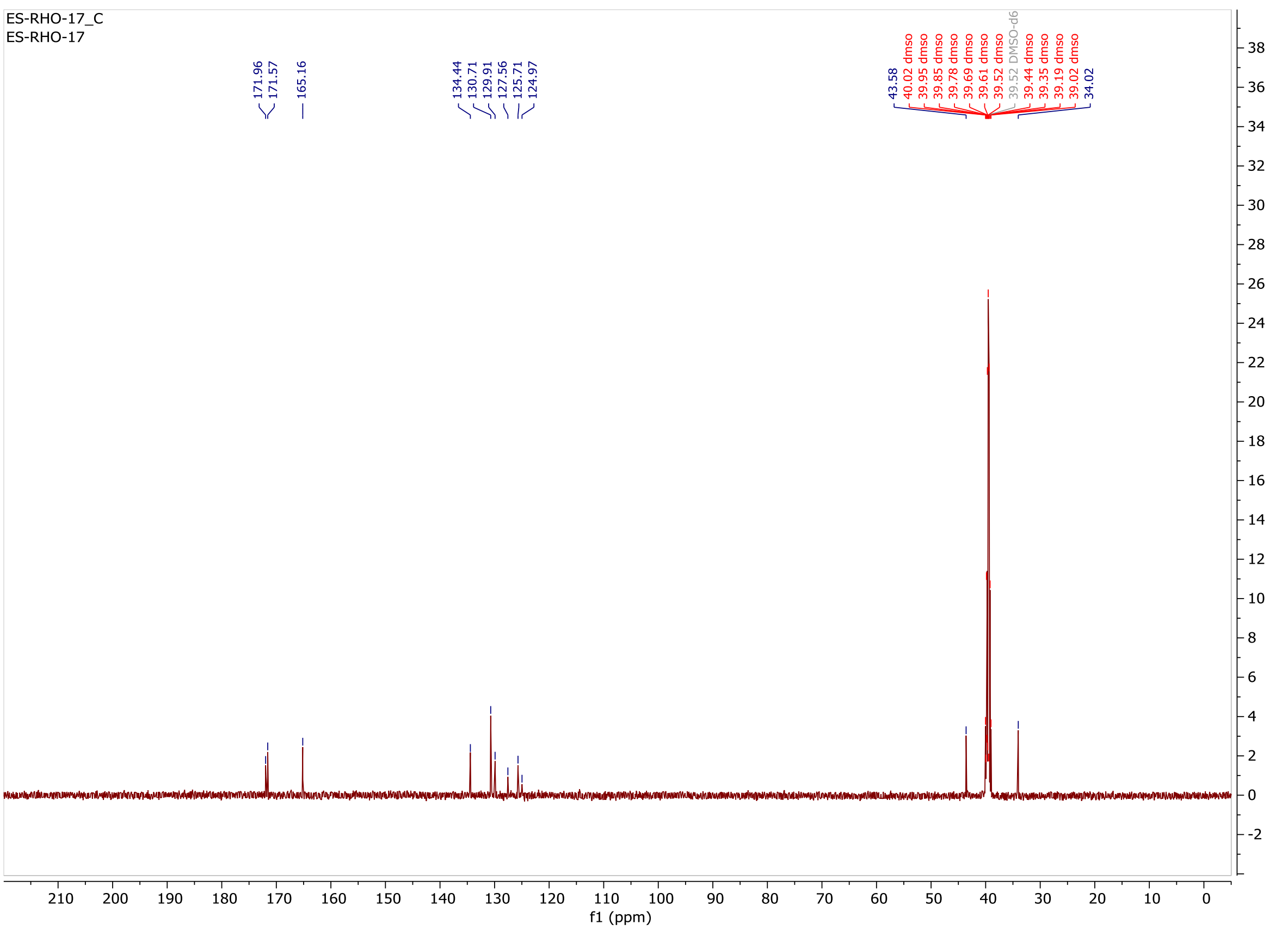

^{1}H NMR *2-(4-Oxo-2-thioxothiazolidin-3-yl)-N-(2,4,6-trichlorophenyl)acetamide* (**1j**)

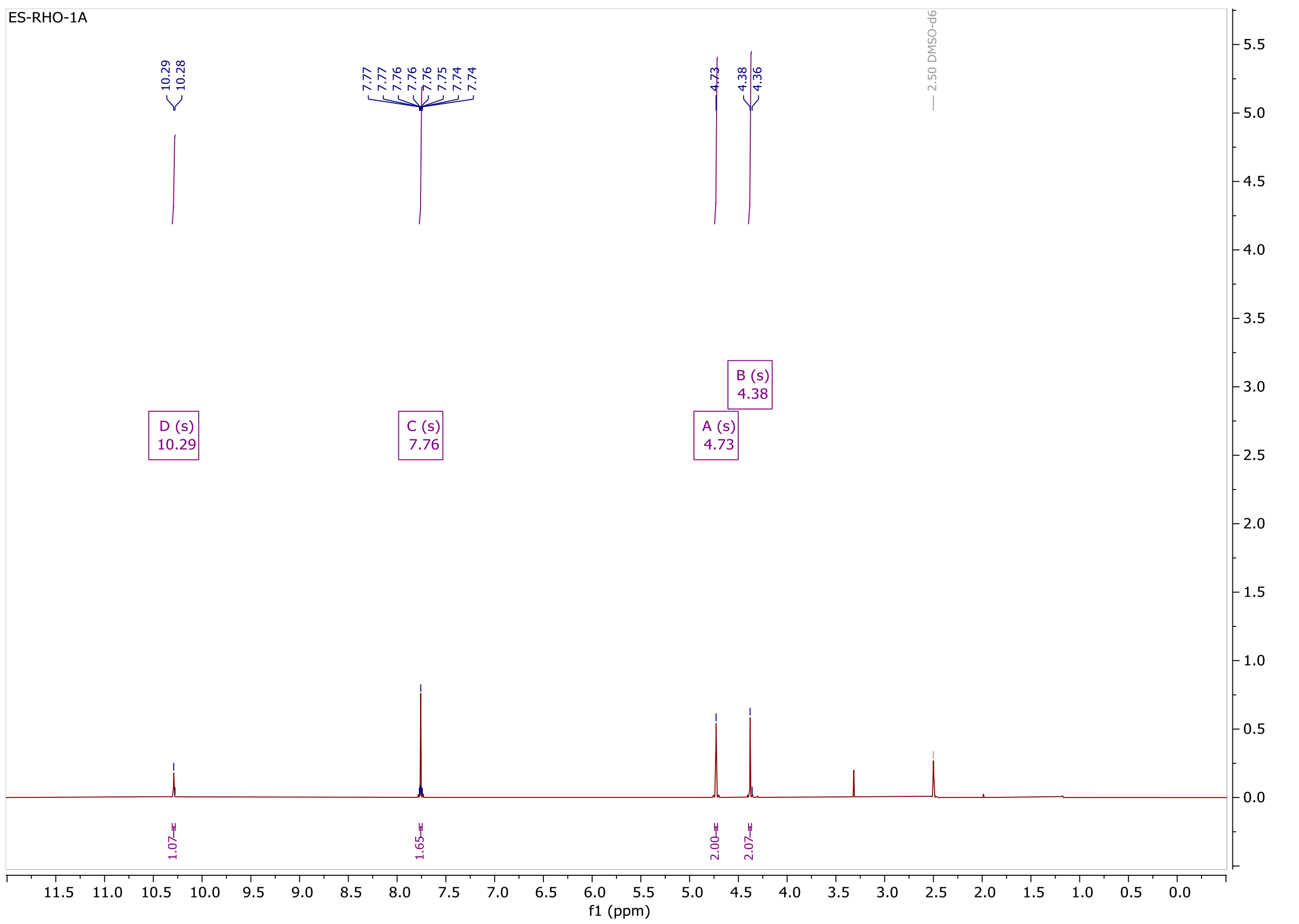

^{13}C NMR *2-(4-Oxo-2-thioxothiazolidin-3-yl)-N-(2,4,6-trichlorophenyl)acetamide* (**1j**)

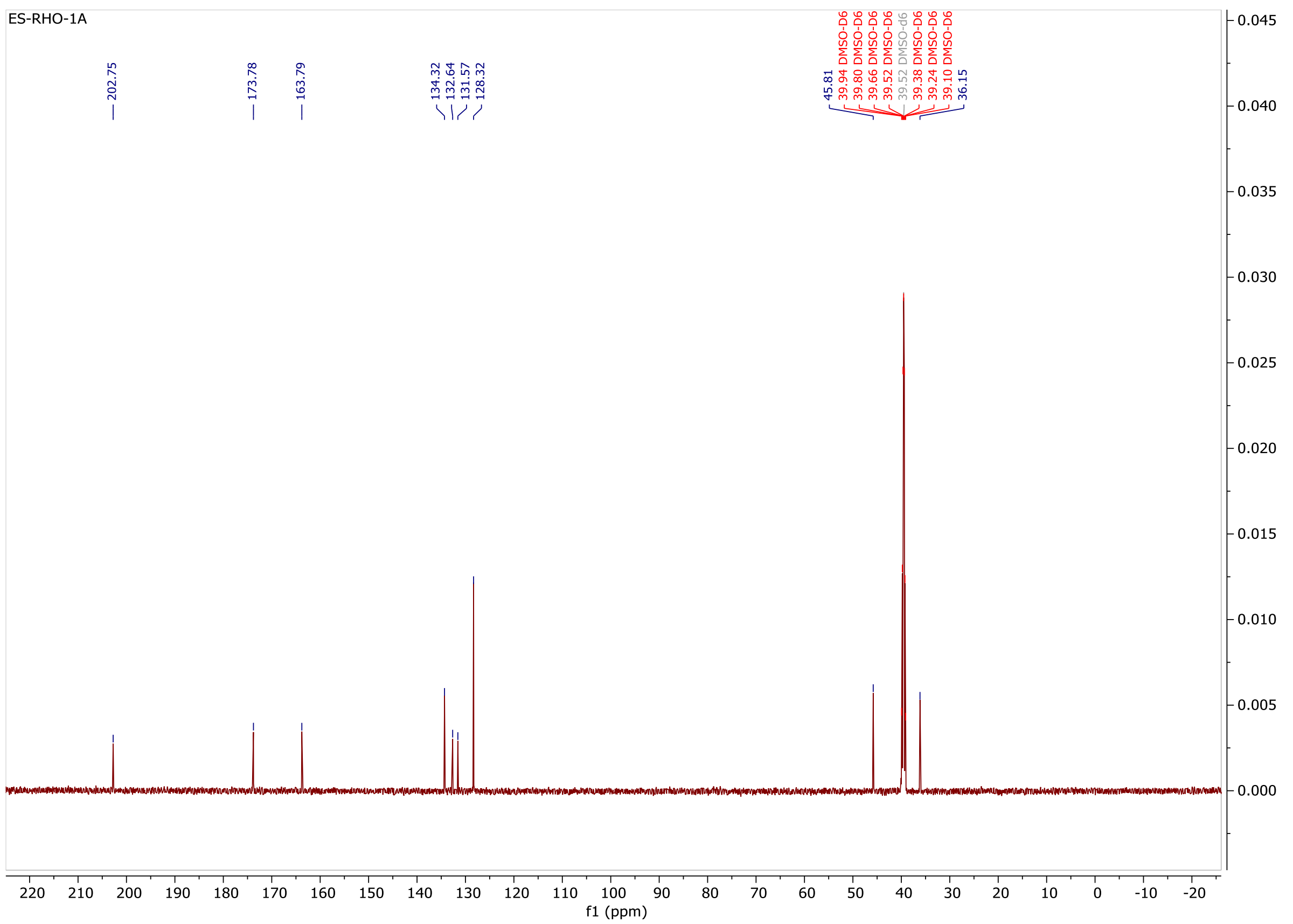

^{1}H NMR *2-(2,4-Dioxothiazolidin-3-yl)-N-(2,4,6-trichlorophenyl)acetamide* (**2j**)

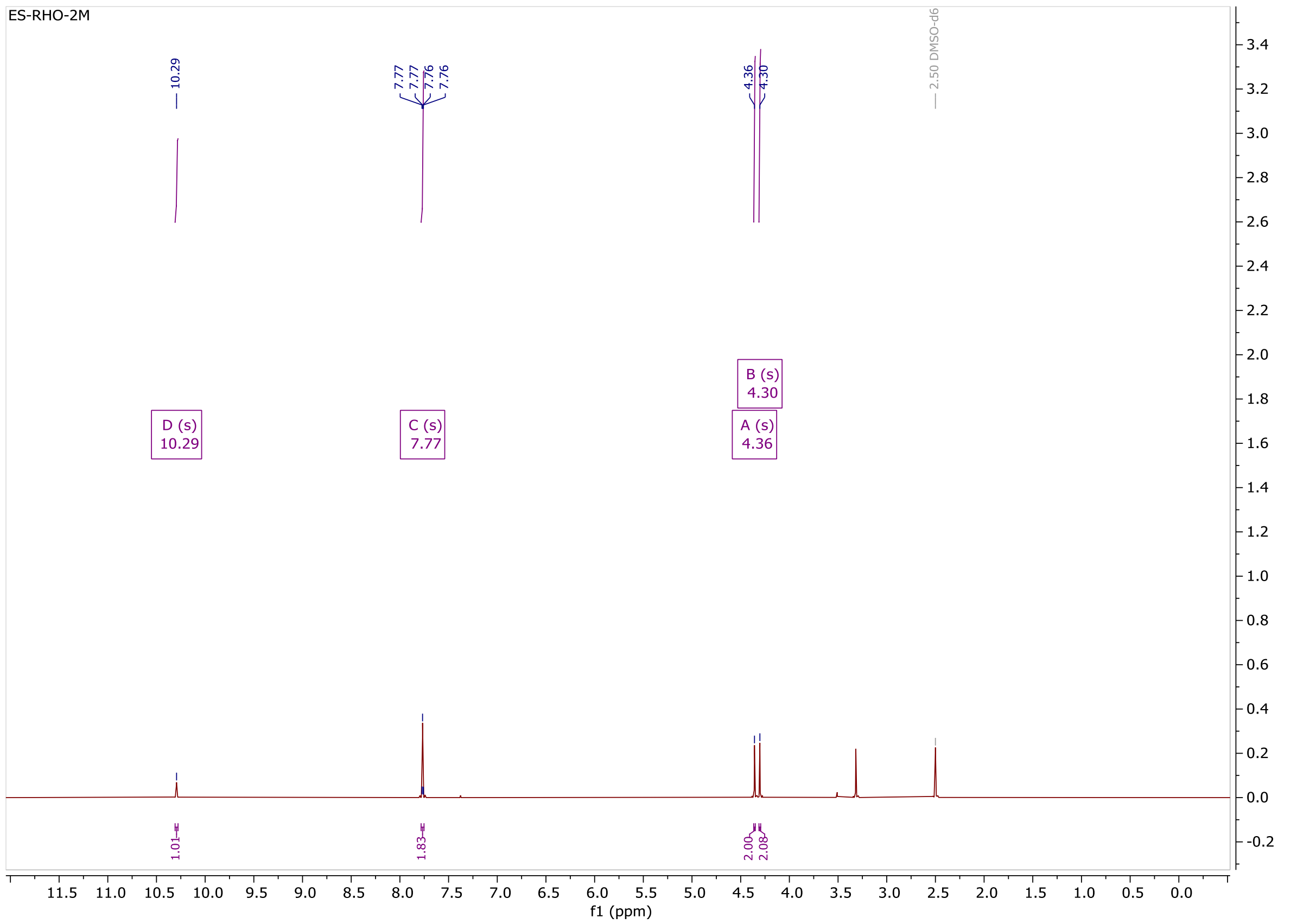

^{13}C NMR *2-(2,4-Dioxothiazolidin-3-yl)-N-(2,4,6-trichlorophenyl)acetamide* (**2j**)

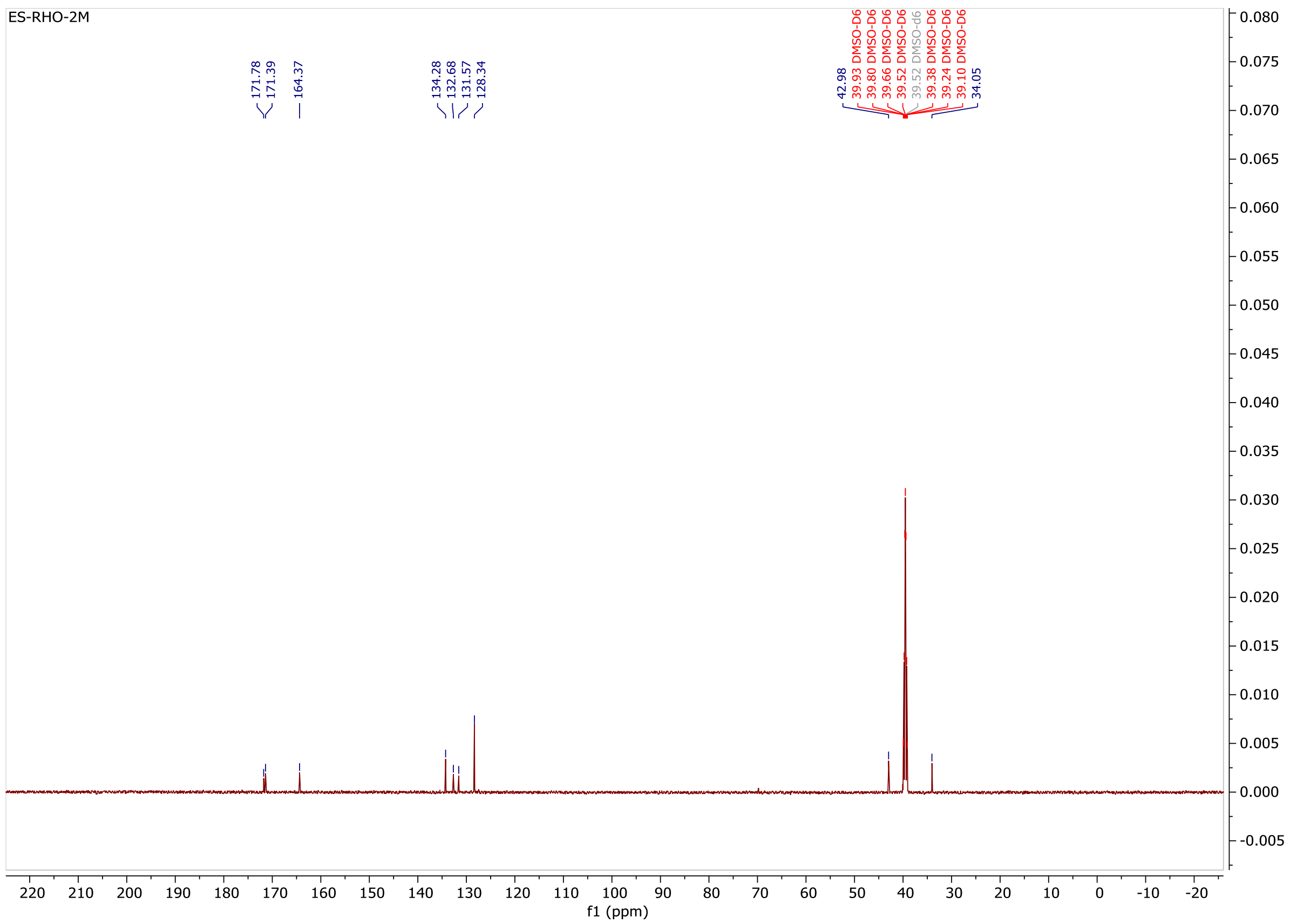

^{1}H NMR *2-(4-Oxo-2-thioxothiazolidin-3-yl)-N-(3,4,5-trifluorophenyl)acetamide* (**1k**)

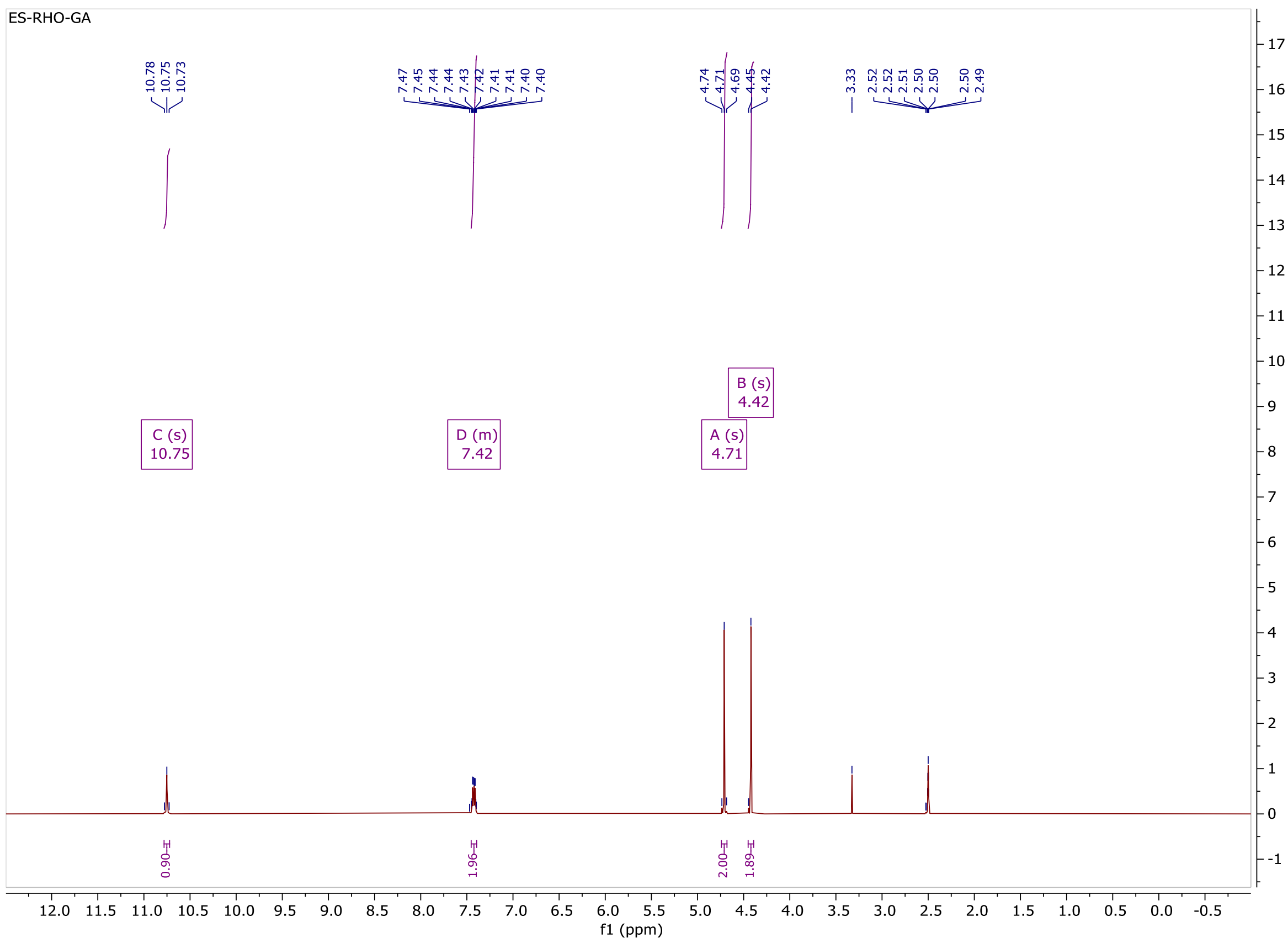

^{13}C NMR *2-(4-Oxo-2-thioxothiazolidin-3-yl)-N-(3,4,5-trifluorophenyl)acetamide* (**1k**)

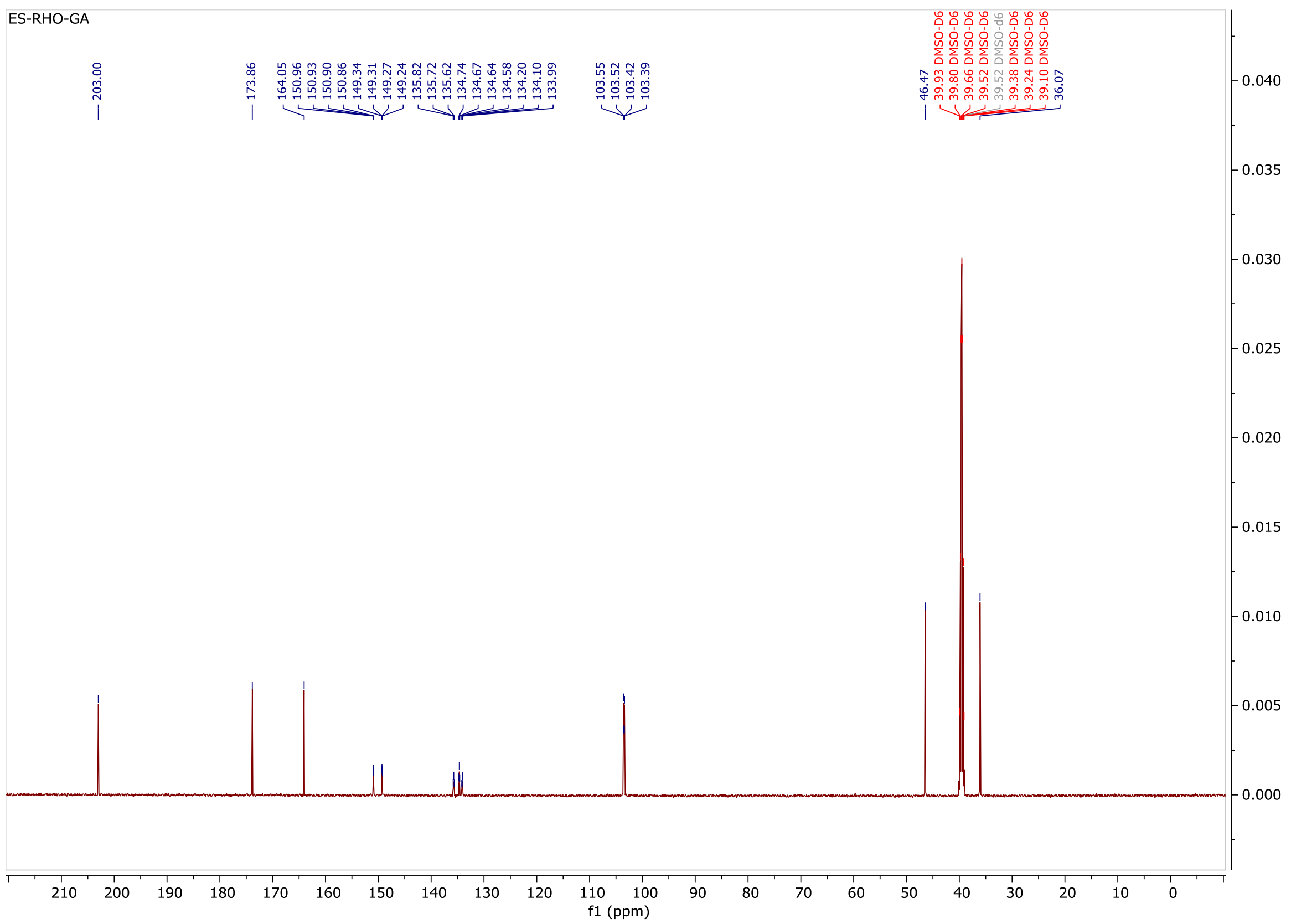

^{1}H NMR *2-(2,4-Dioxothiazolidin-3-yl)-N-(3,4,5-trifluorophenyl)acetamide* (**2k**)

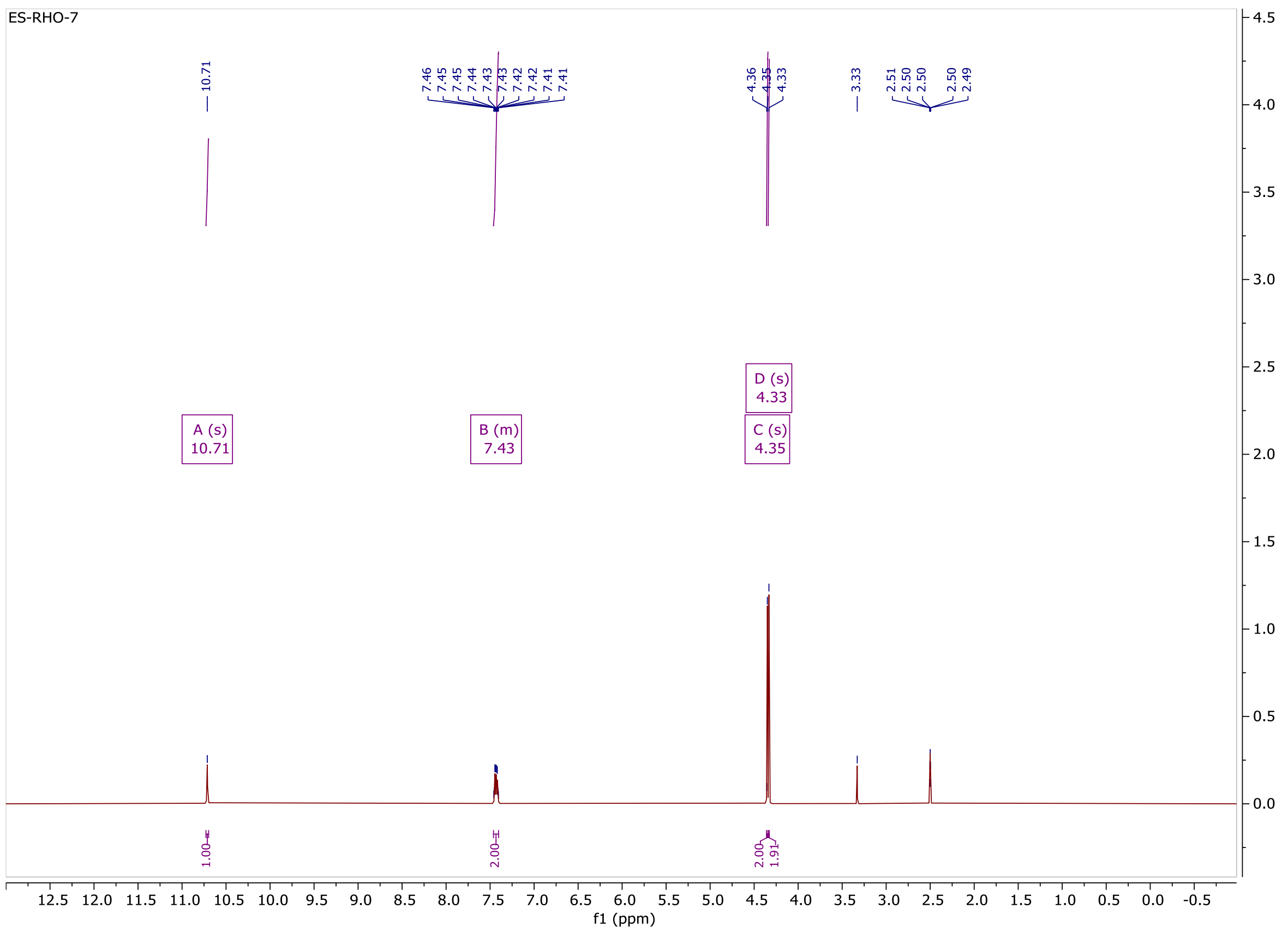

^{13}C NMR *2-(2,4-Dioxothiazolidin-3-yl)-N-(3,4,5-trifluorophenyl)acetamide* (**2k**)

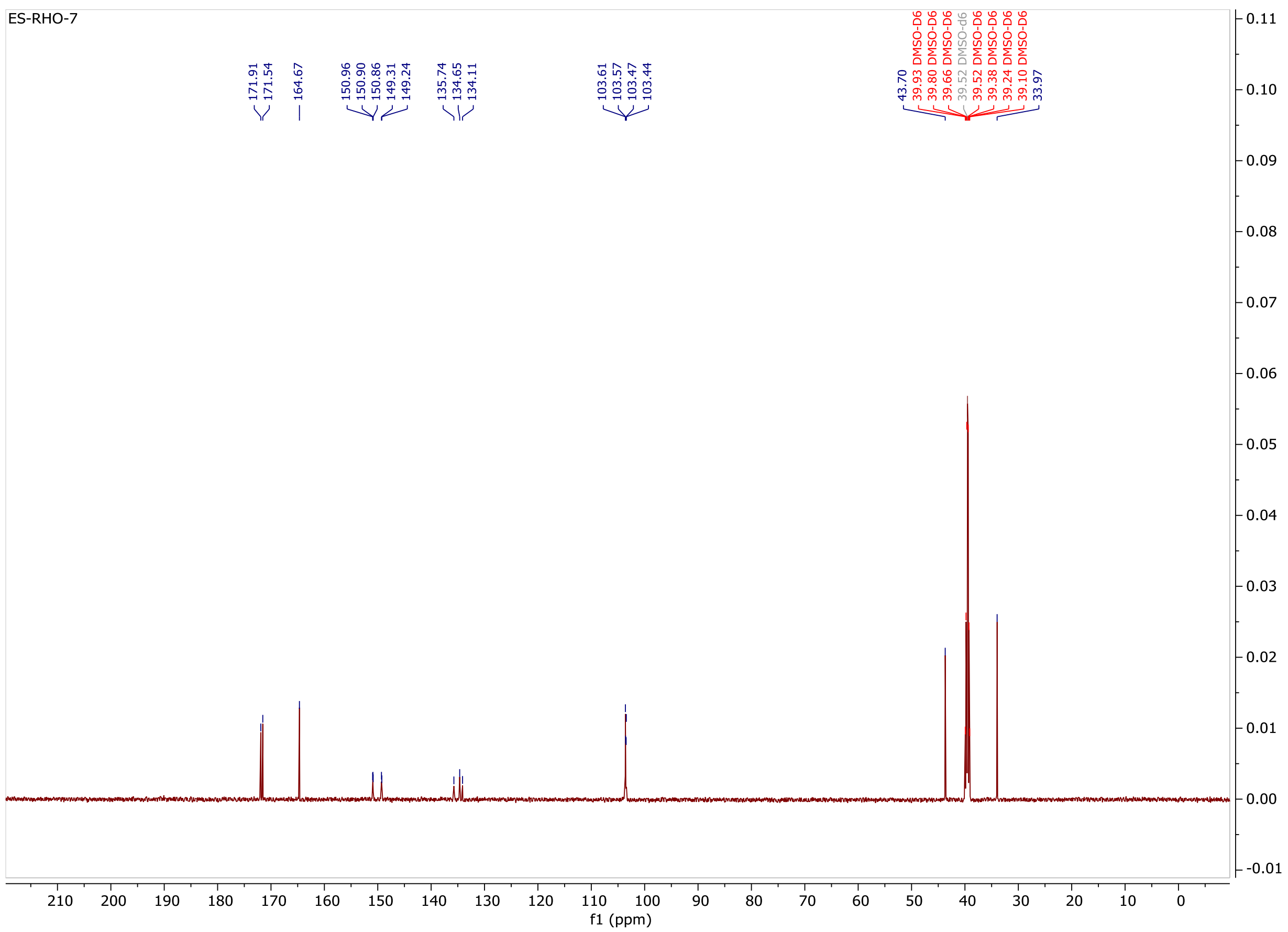

^{1}H NMR *2-(4-Oxo-2-thioxothiazolidin-3-yl)-N-(3,4,5-trimethoxyphenyl)acetamide* (**1l**)

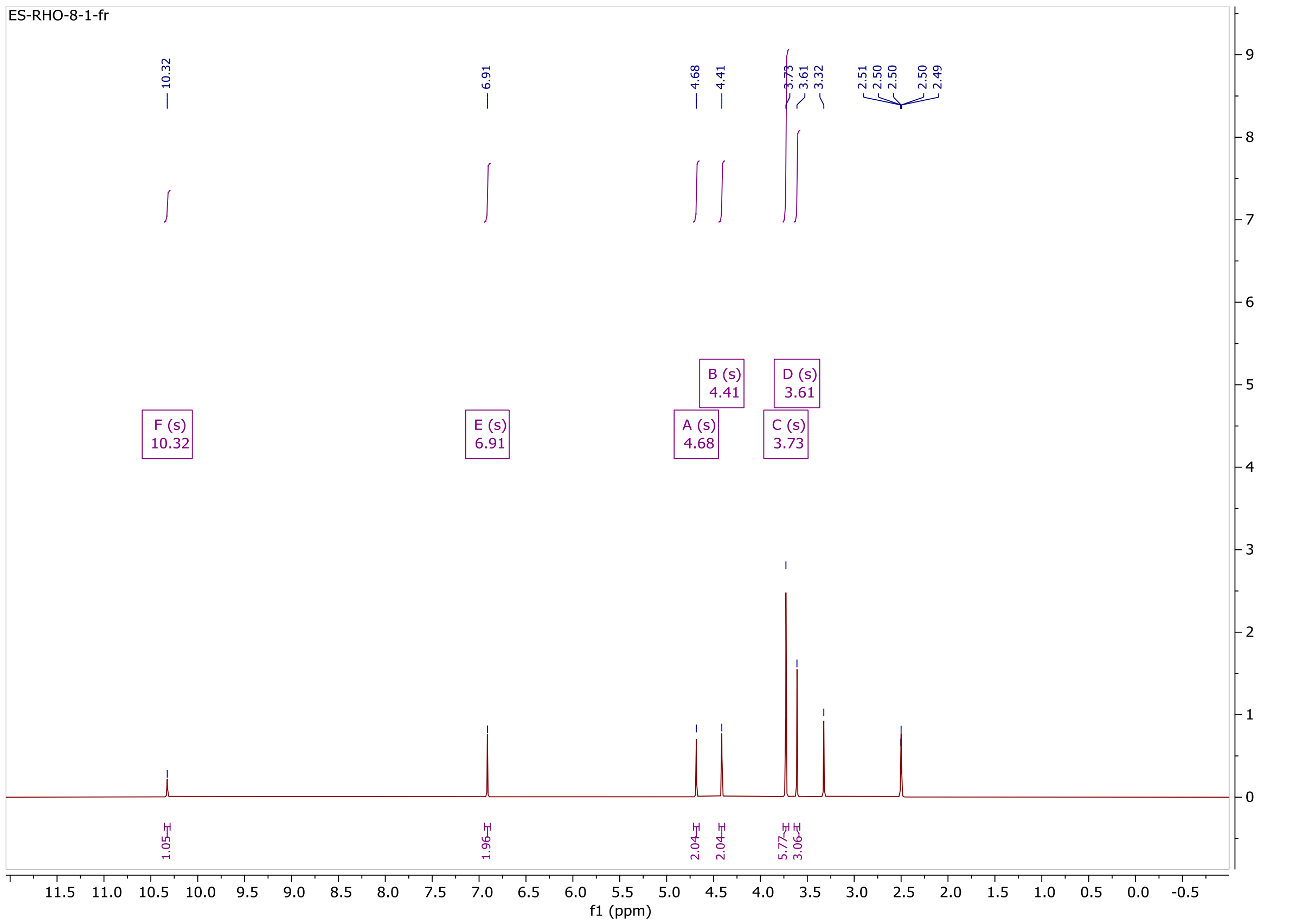

^{13}C NMR *2-(4-Oxo-2-thioxothiazolidin-3-yl)-N-(3,4,5-trimethoxyphenyl)acetamide* (**1l**)

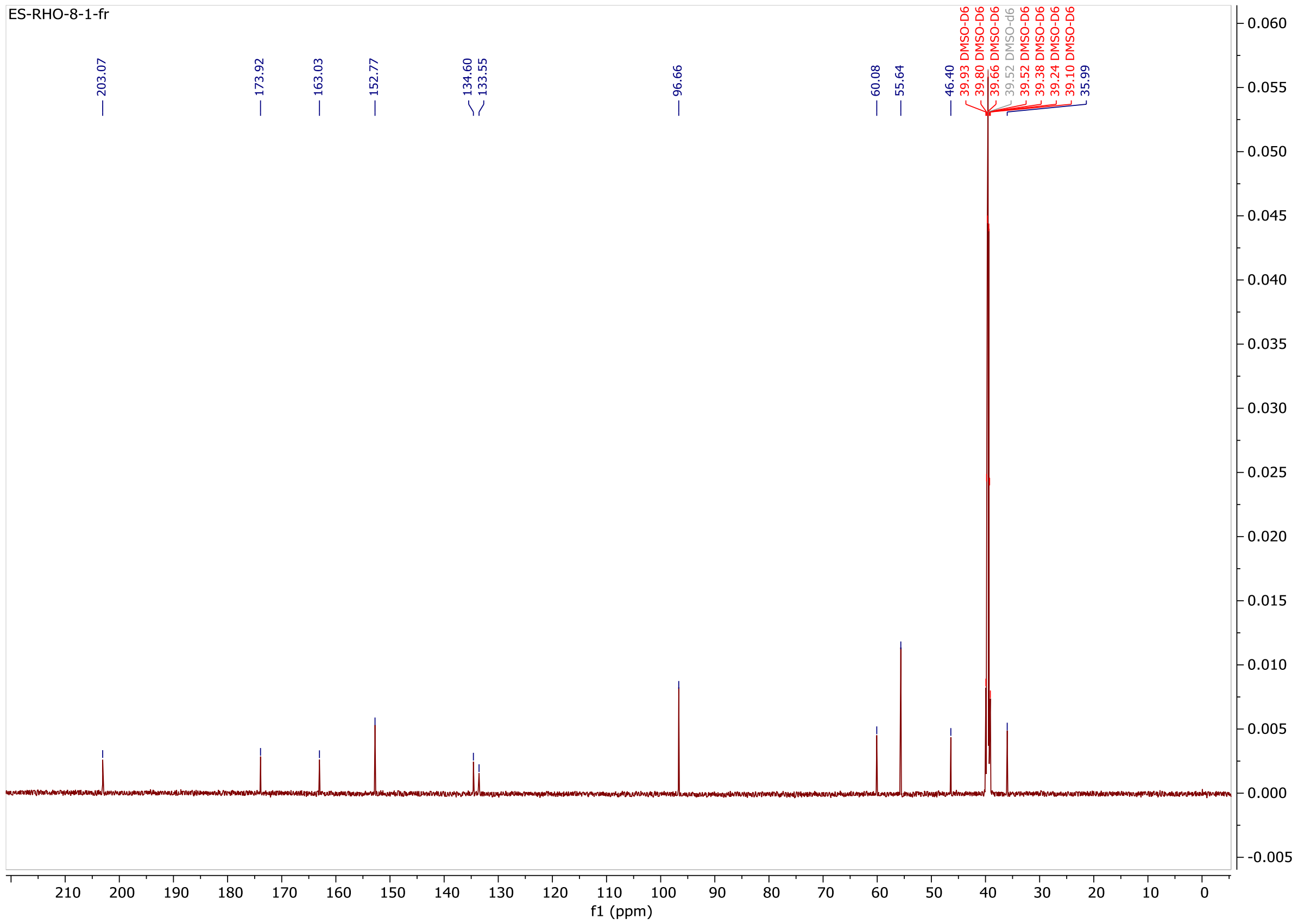

^{1}H NMR *2-(2,4-Dioxothiazolidin-3-yl)-N-(3,4,5-trimethoxyphenyl)acetamide* (**2l**)

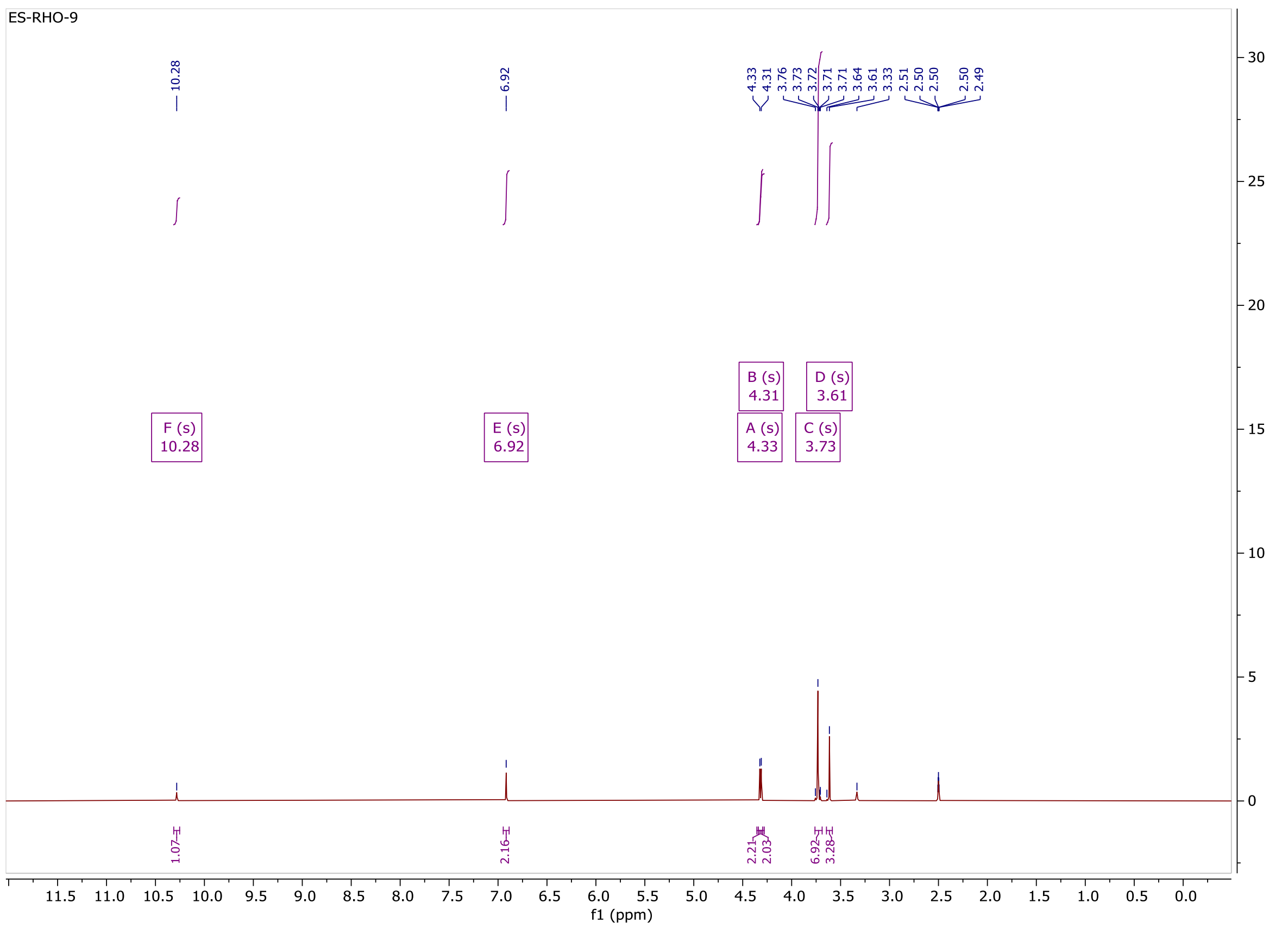

^{13}C NMR *2-(2,4-Dioxothiazolidin-3-yl)-N-(3,4,5-trimethoxyphenyl)acetamide* (**2l**)

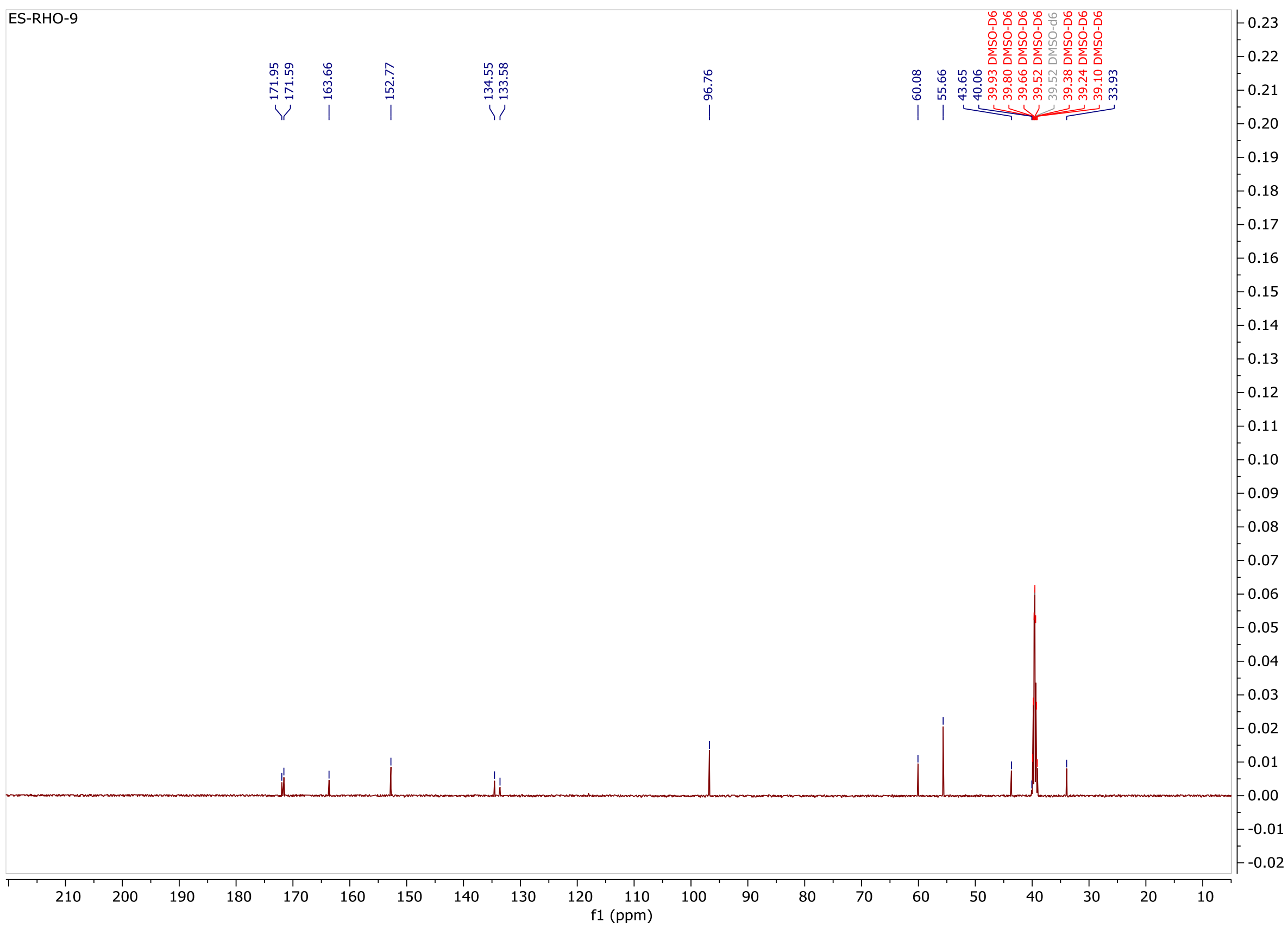

^{1}H NMR *2-(4-Oxo-2-thioxothiazolidin-3-yl)-N-(3,4,5-trimethylphenyl)acetamide* (**1m**)

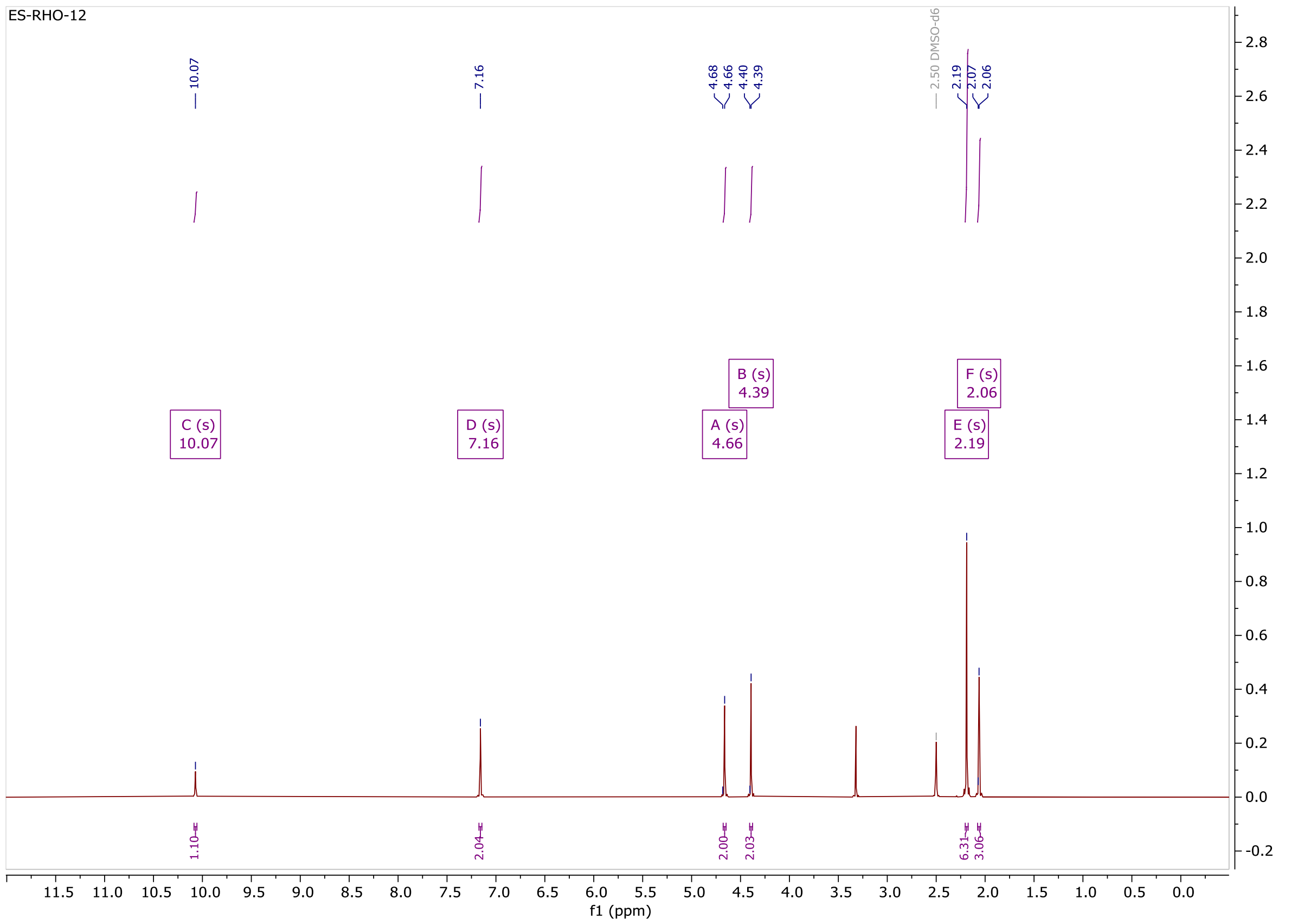

^{13}C NMR *2-(4-Oxo-2-thioxothiazolidin-3-yl)-N-(3,4,5-trimethylphenyl)acetamide* (**1m**)

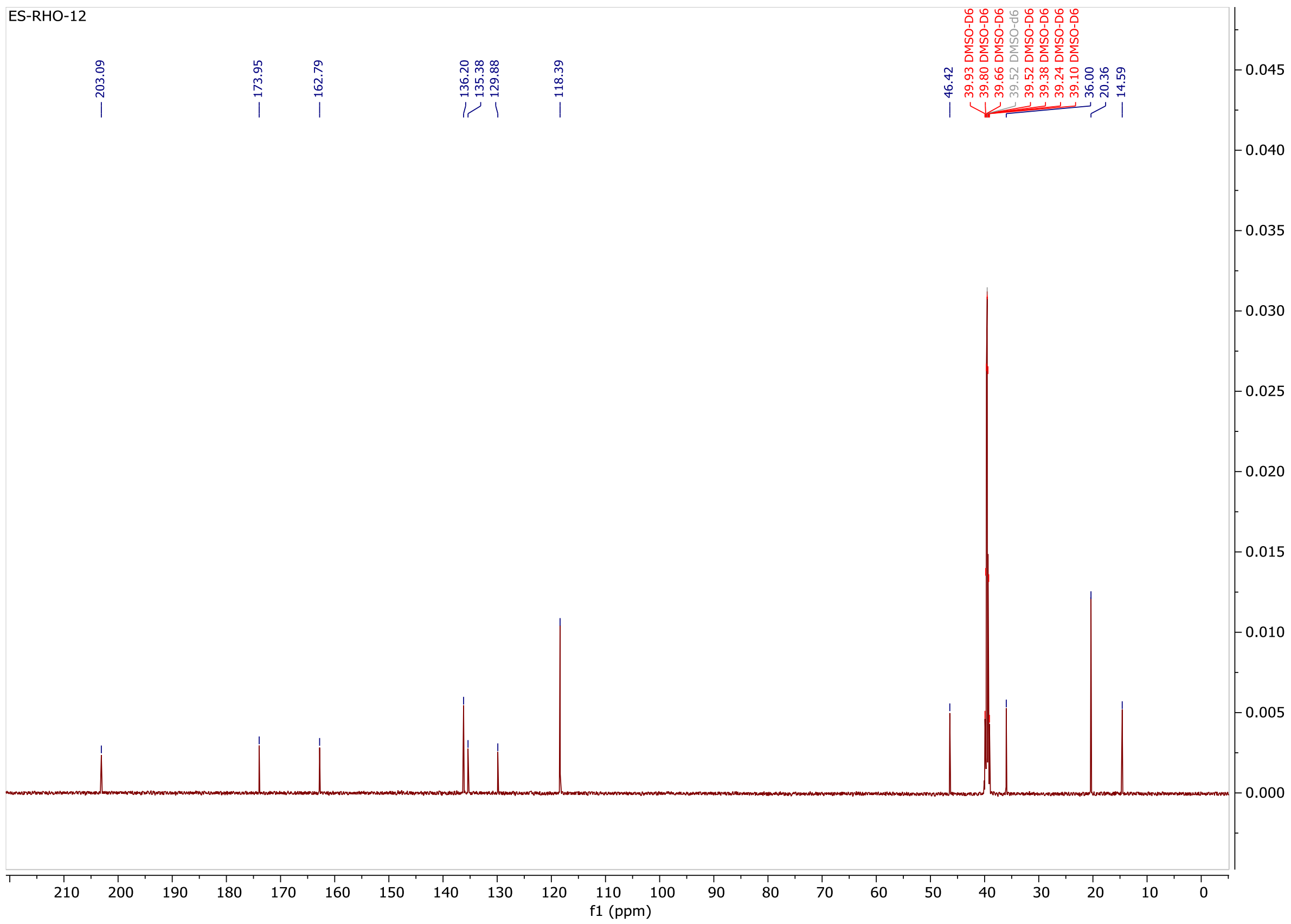

^{1}H NMR *2-(2,4-Dioxothiazolidin-3-yl)-N-(3,4,5-trimethylphenyl)acetamide* (**2m**)

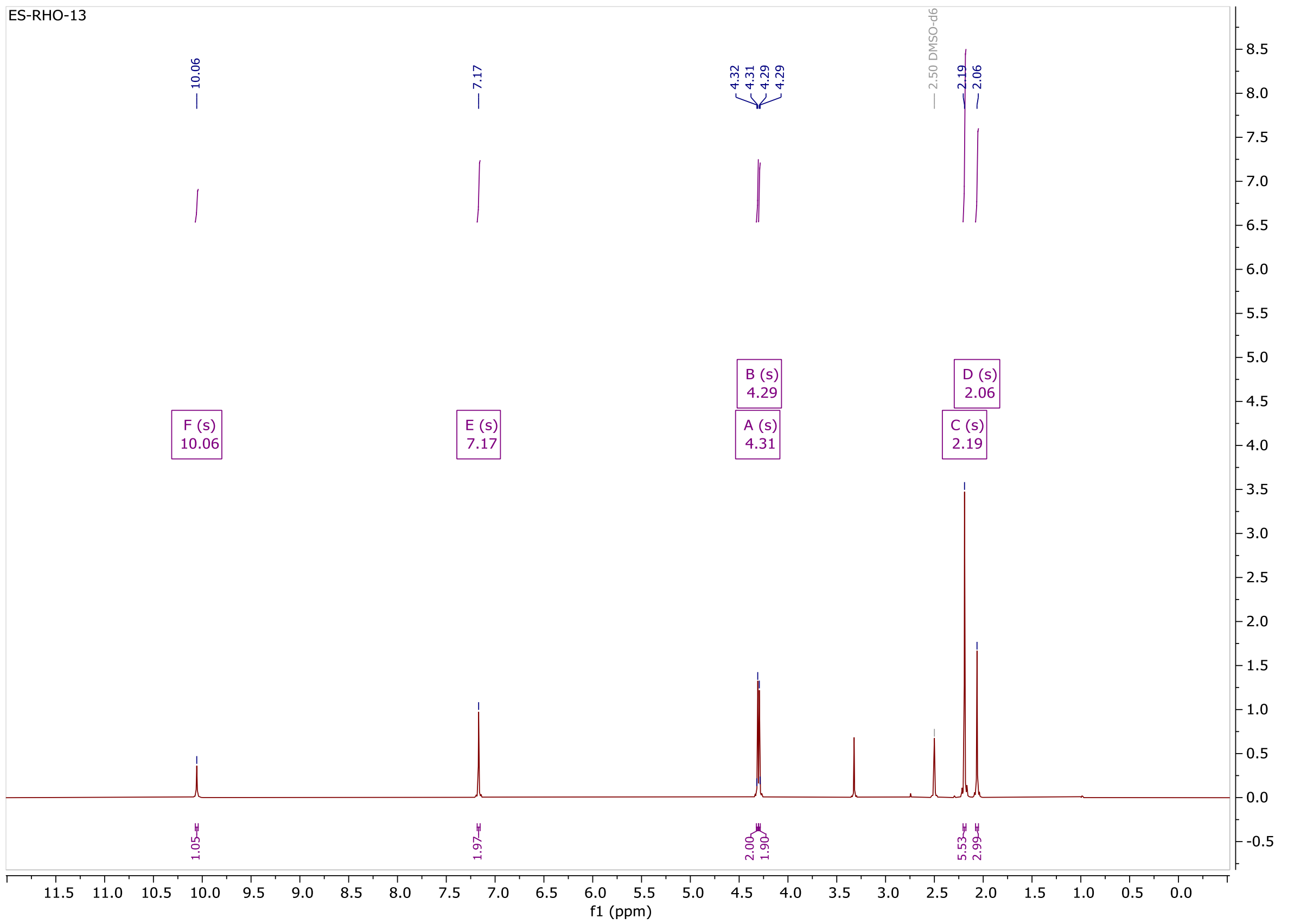

^{13}C NMR *2-(2,4-Dioxothiazolidin-3-yl)-N-(3,4,5-trimethylphenyl)acetamide* (**2m**)

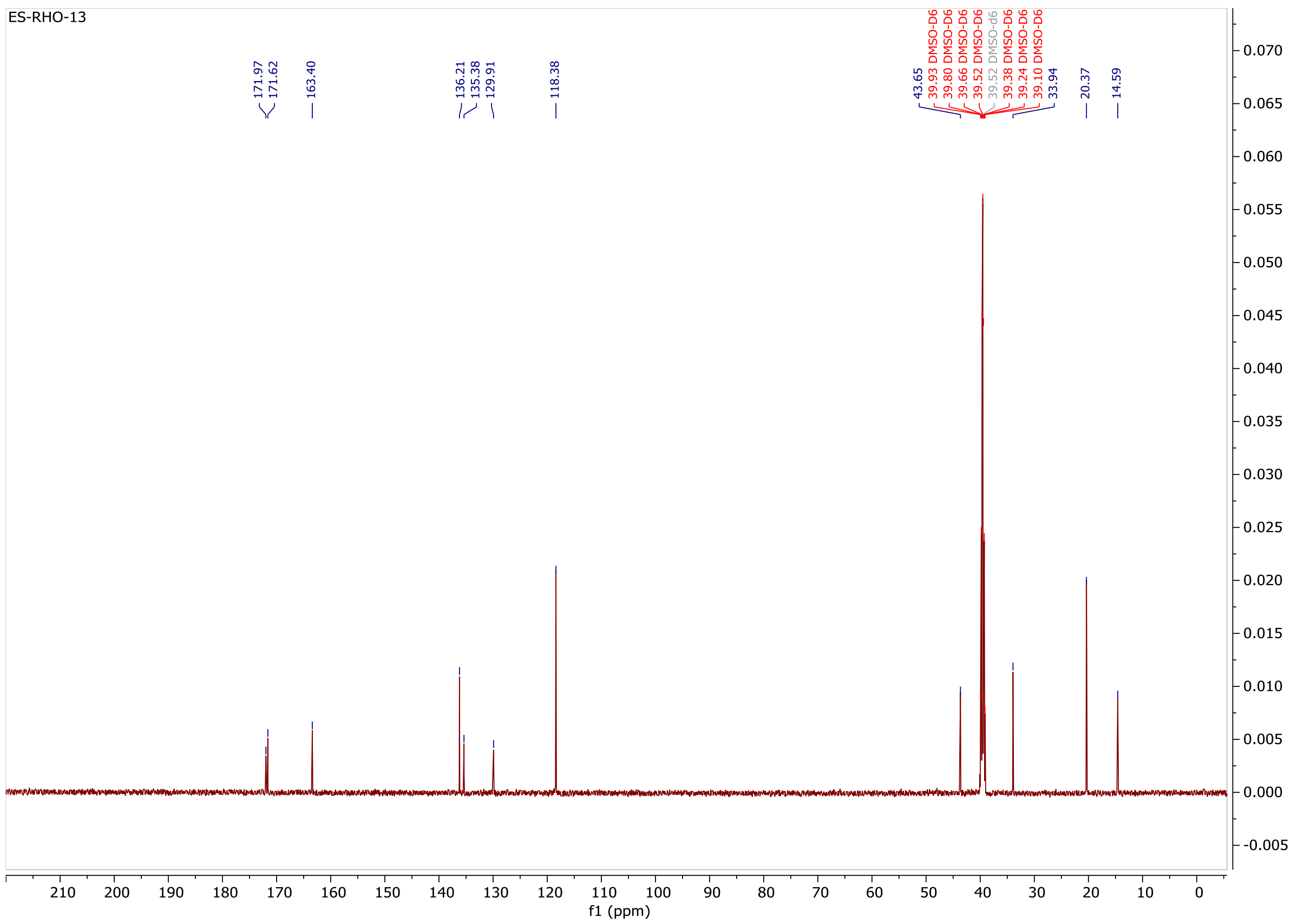

^{1}H NMR *N-(3,5-Dichloro-4-hydroxyphenyl)-2-(4-oxo-2-thioxothiazolidin-3-yl)acetamide* (**1n**)

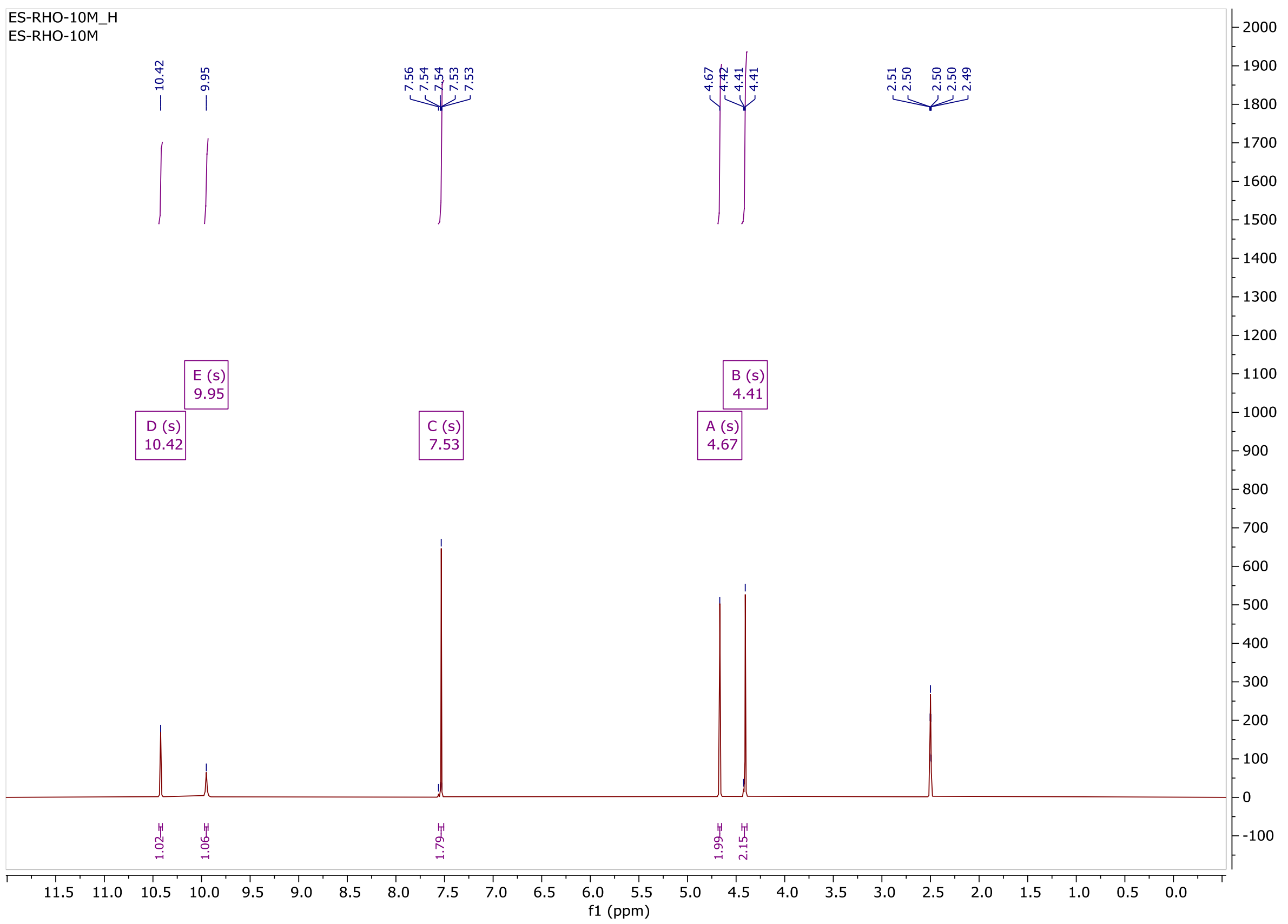

^{13}C NMR *N-(3,5-Dichloro-4-hydroxyphenyl)-2-(4-oxo-2-thioxothiazolidin-3-yl)acetamide* (**1n**)

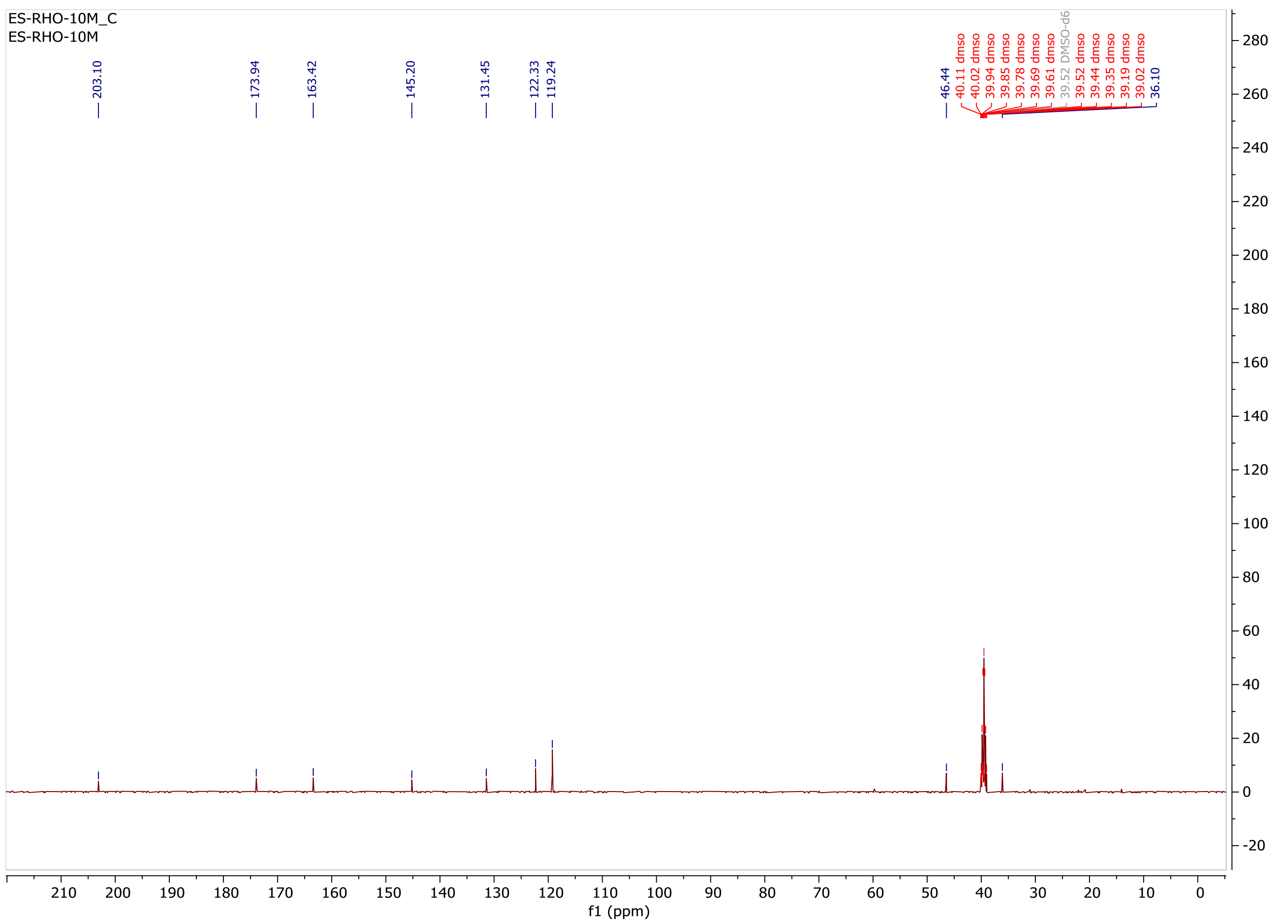

^{1}H NMR *N-(3,5-Dichloro-4-hydroxyphenyl)-2-(2,4-dioxothiazolidin-3-yl)acetamide* (**2n**)

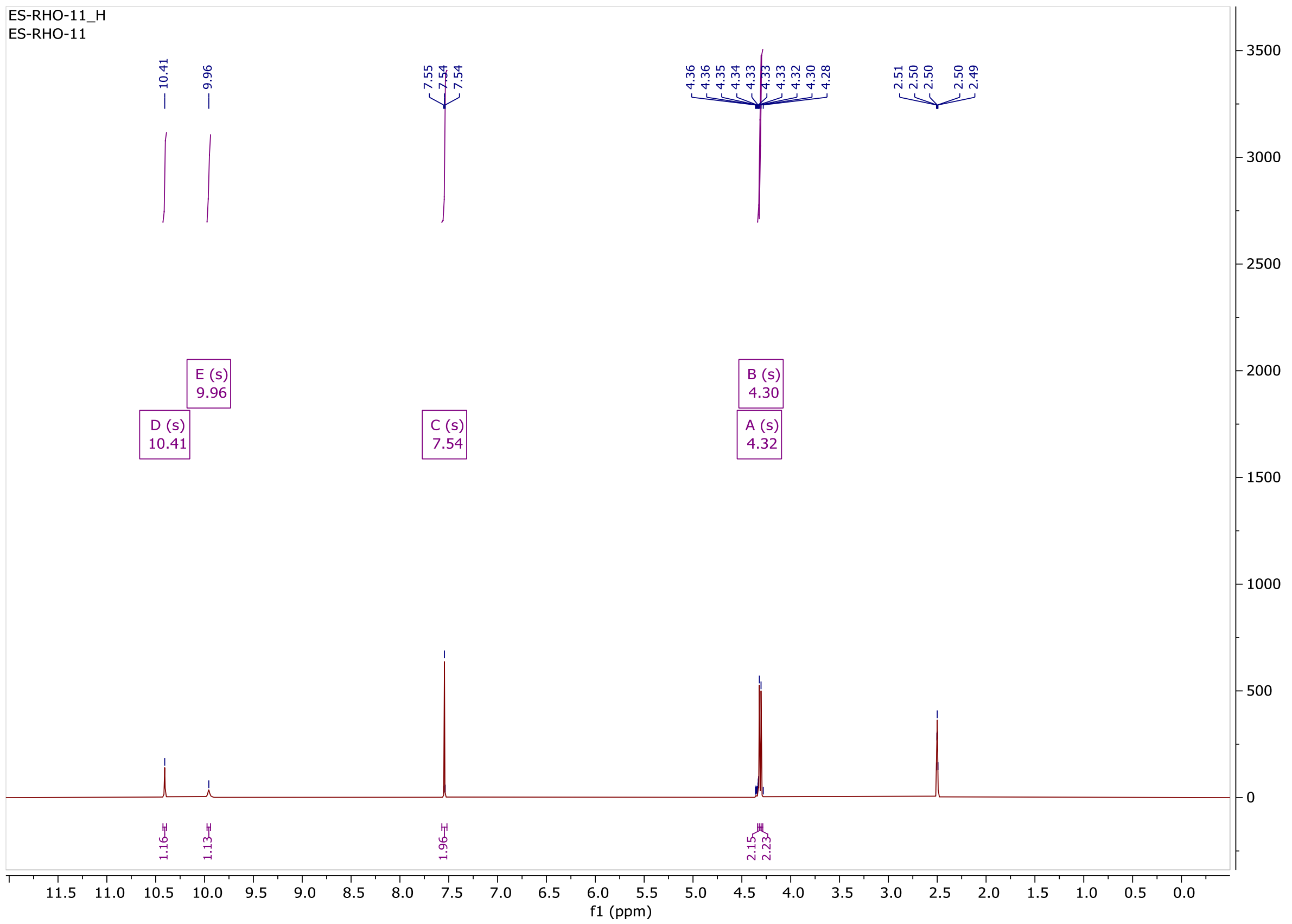

^{13}C NMR *N-(3,5-Dichloro-4-hydroxyphenyl)-2-(2,4-dioxothiazolidin-3-yl)acetamide* (**2n**)

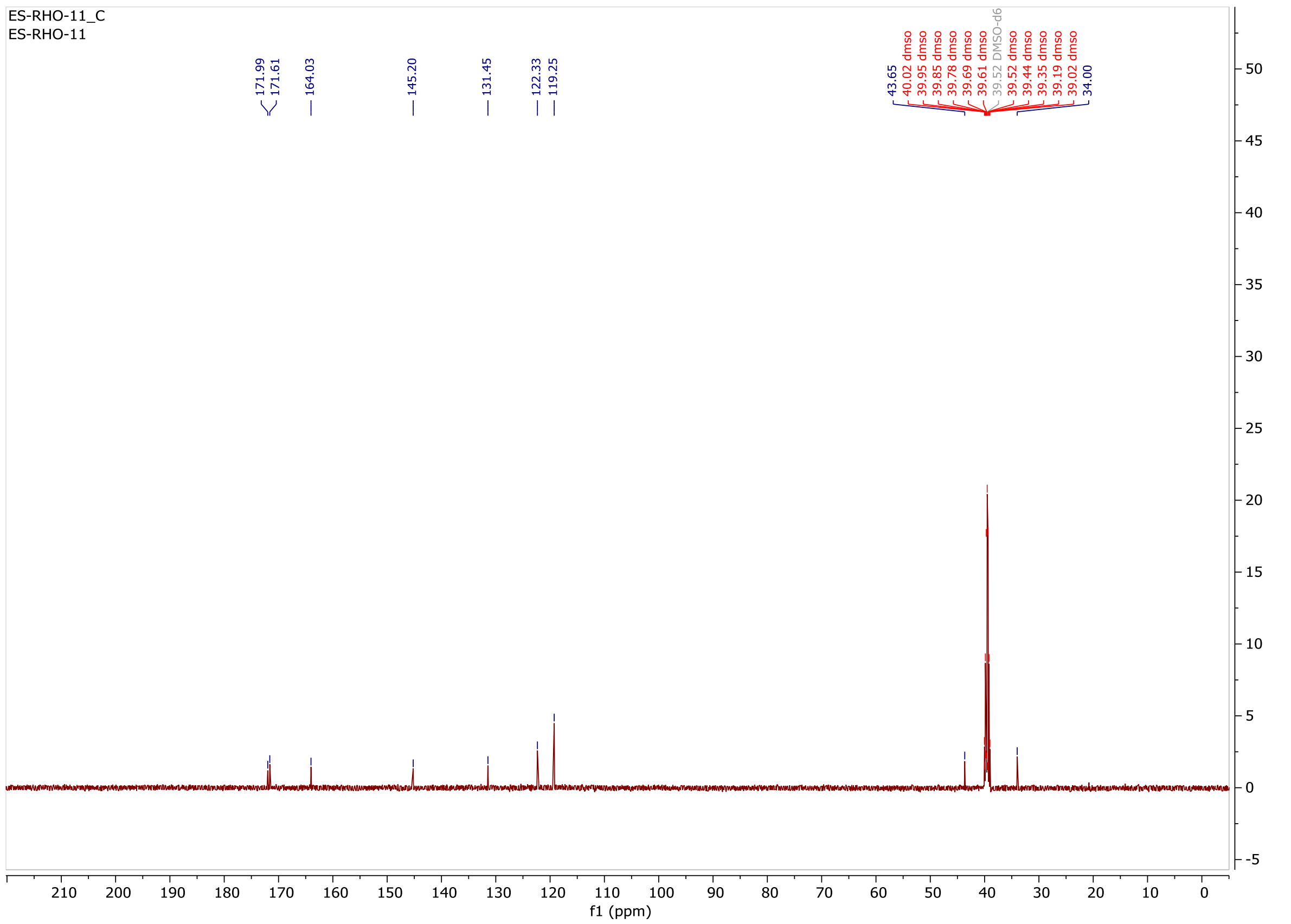

^{1}H NMR *N-(4-Bromo-3,5-difluorophenyl)-2-(4-oxo-2-thioxothiazolidin-3-yl)acetamide* (**1o**)

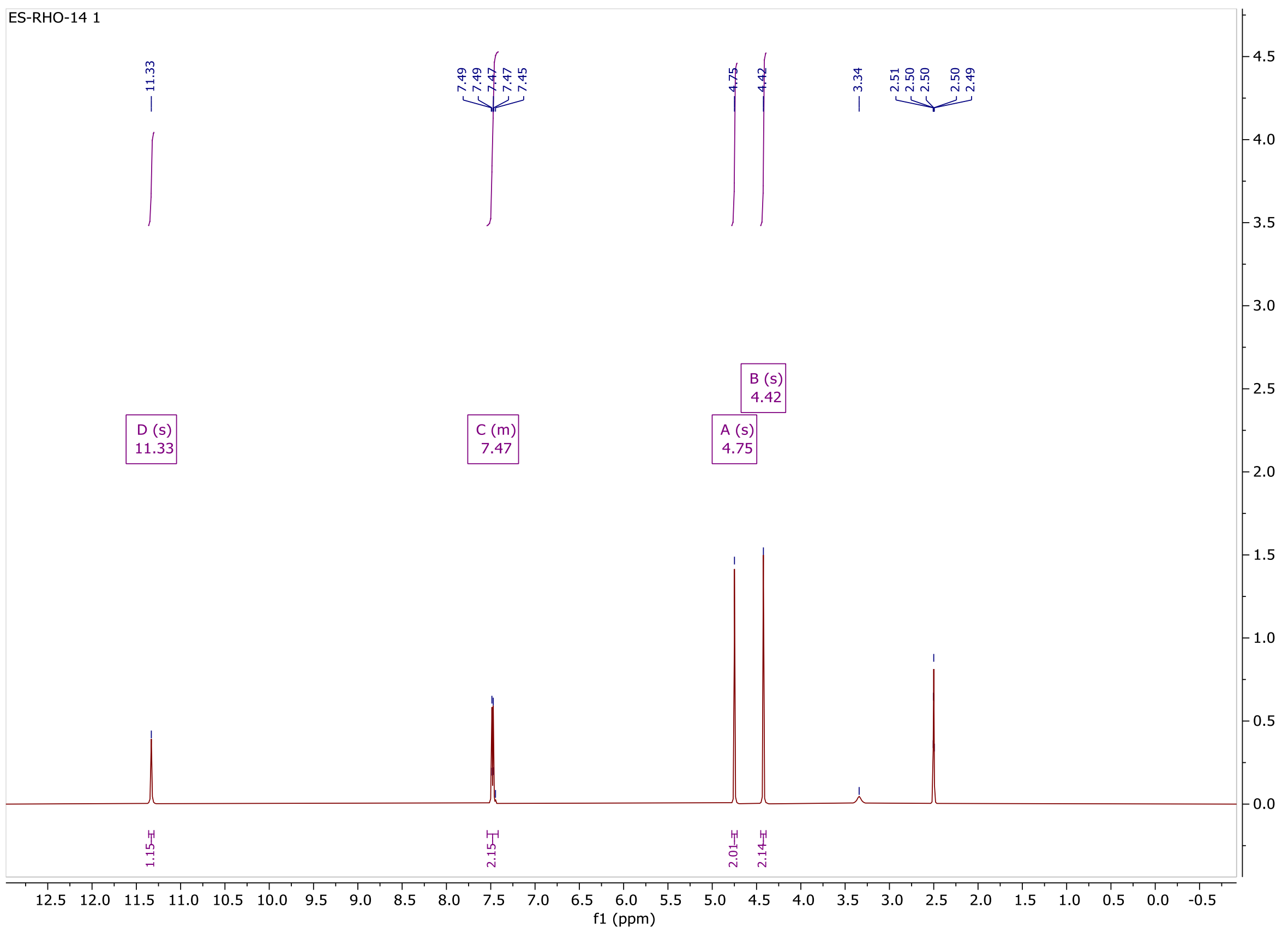

^{13}C NMR *N-(4-Bromo-3,5-difluorophenyl)-2-(4-oxo-2-thioxothiazolidin-3-yl)acetamide* (**1o**)

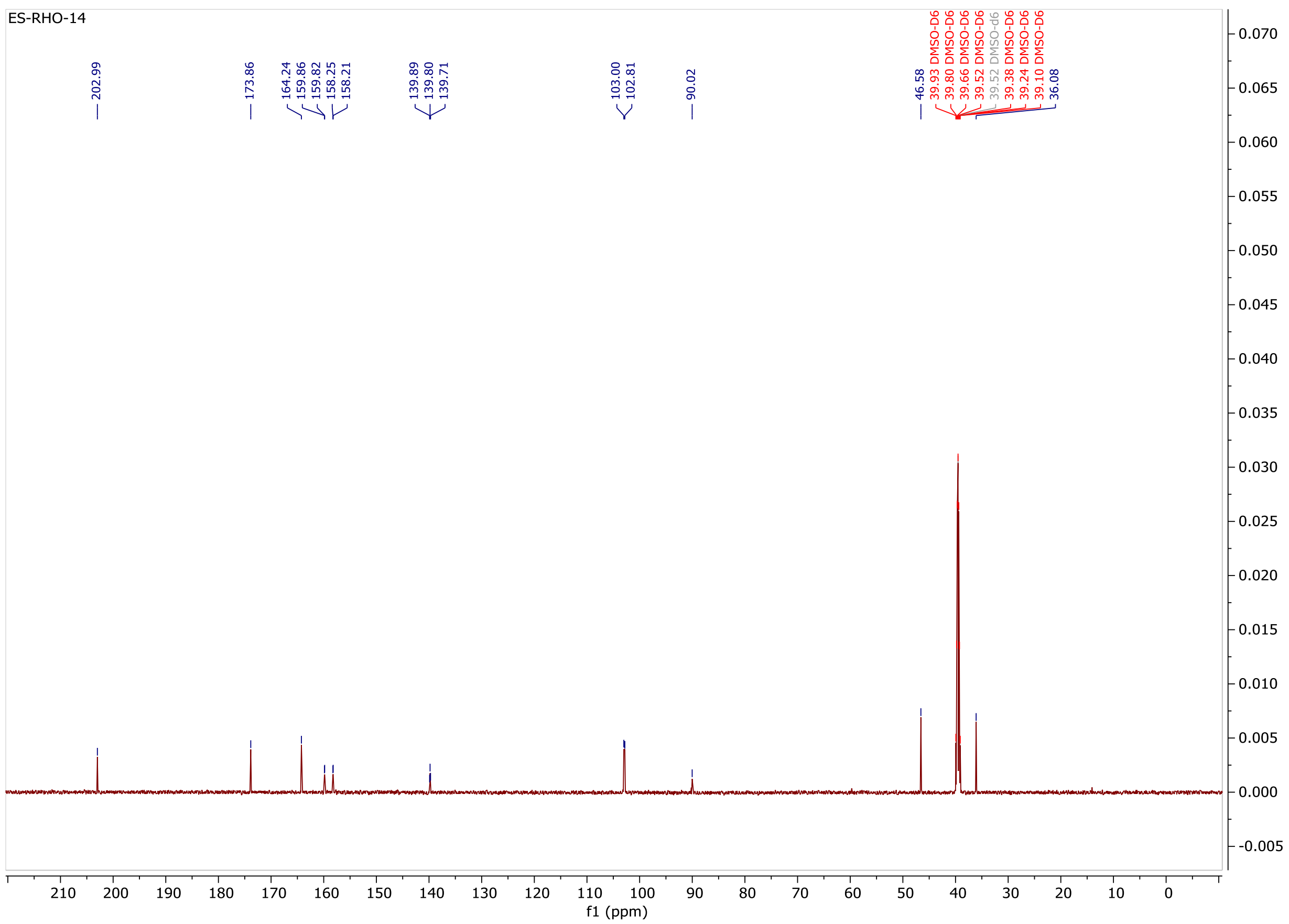

^{1}H NMR *N-(4-Bromo-3,5-difluorophenyl)-2-(2,4-dioxothiazolidin-3-yl)acetamide* (**2o**)

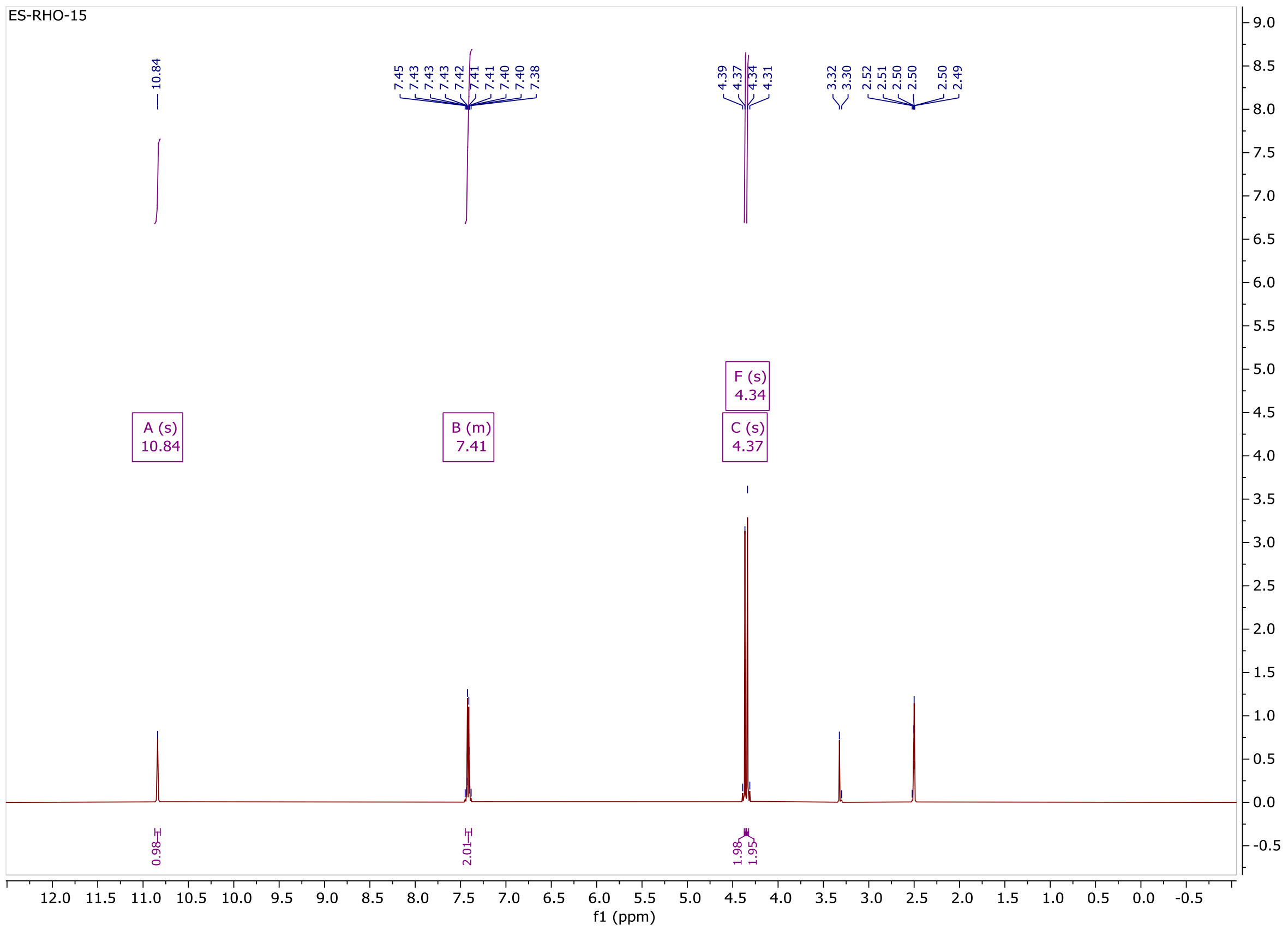

^{13}C NMR *N-(4-Bromo-3,5-difluorophenyl)-2-(2,4-dioxothiazolidin-3-yl)acetamide* (**2o**)

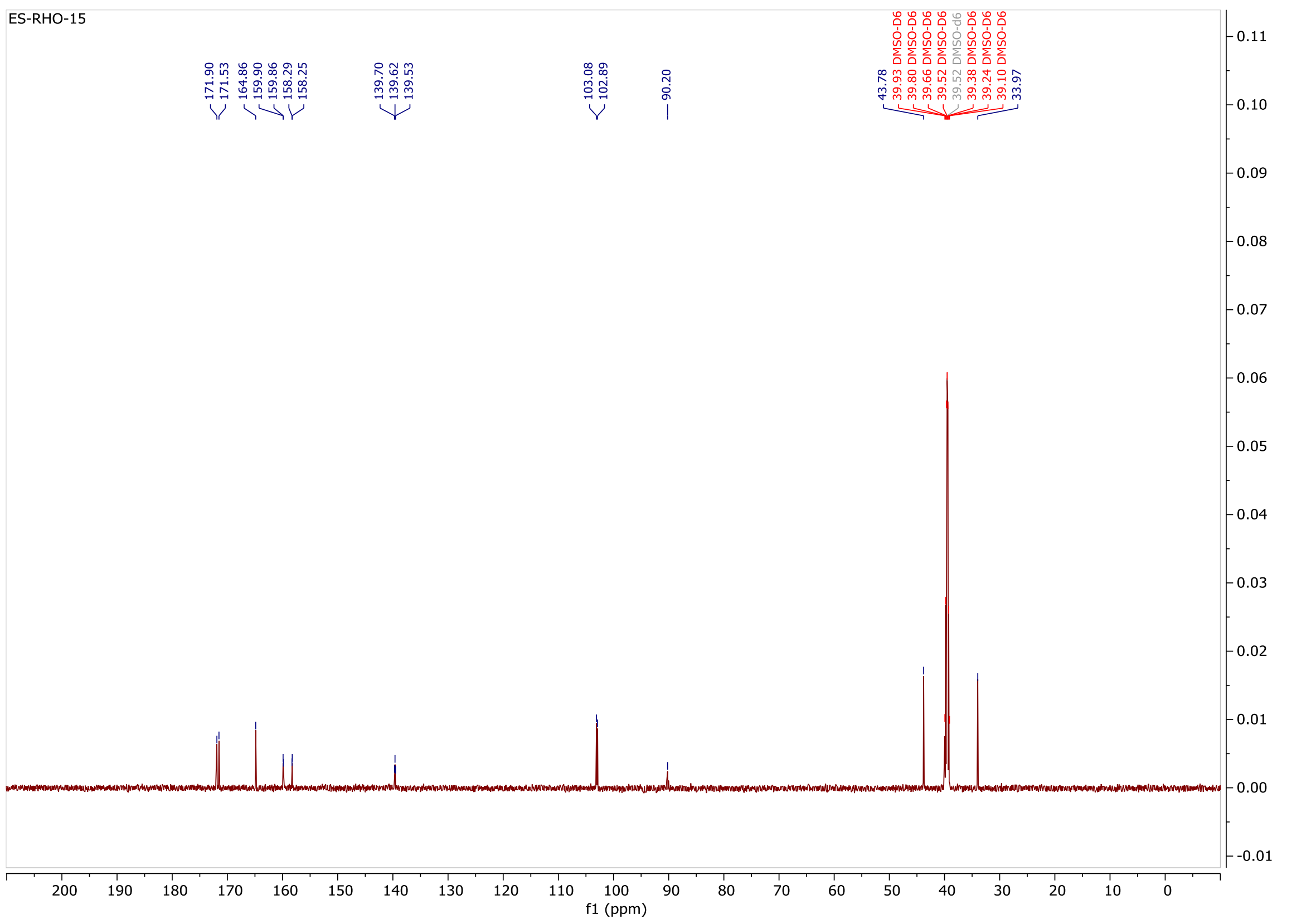

5. The IC_{50} values graphs used for their calculations

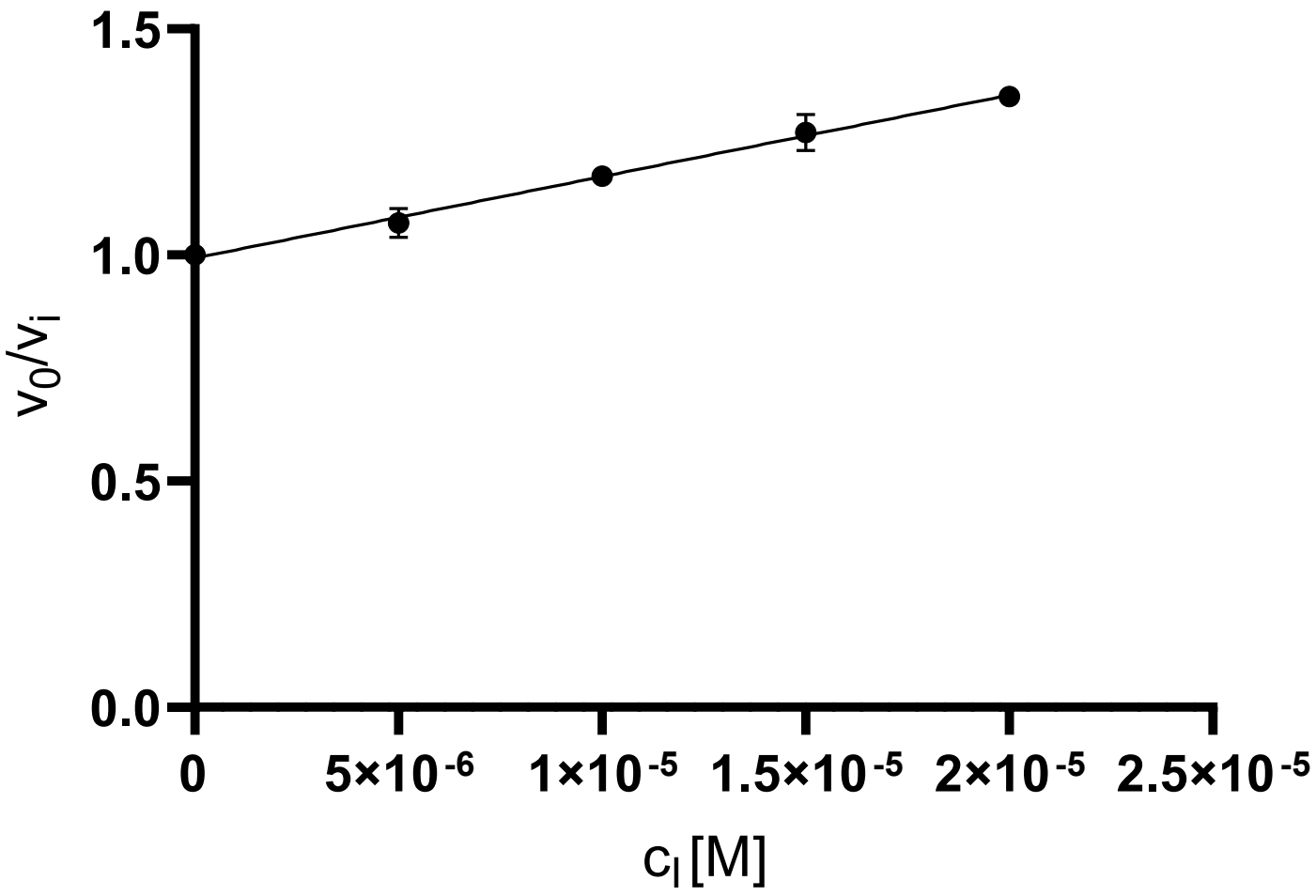

Figure S2. The dependence v_0/v_i vs. concentration of inhibitor **1a** inhibiting eeAChE.

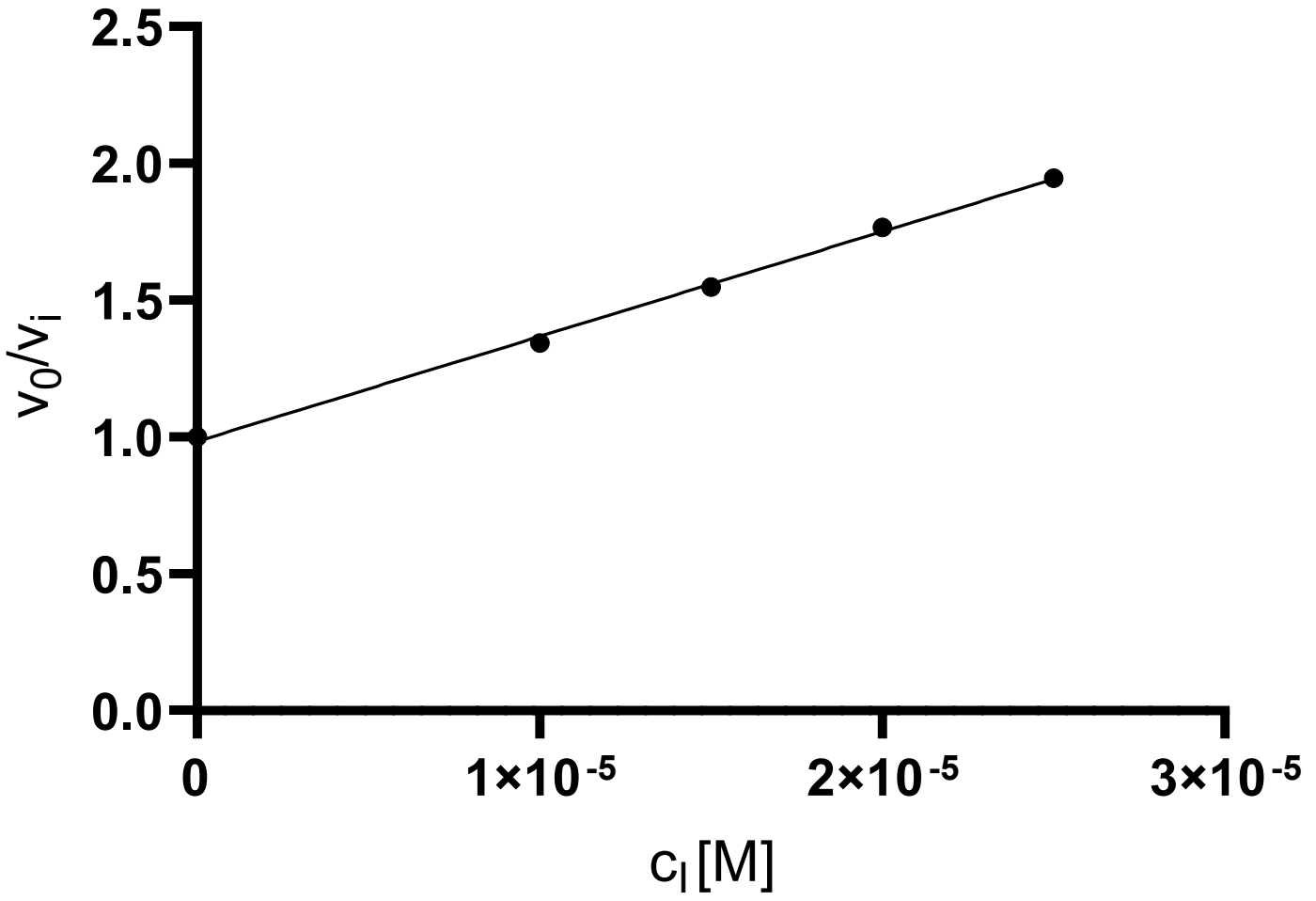

Figure S3. The dependence v_0/v_i vs. concentration of inhibitor **1a** inhibiting eqBChE.

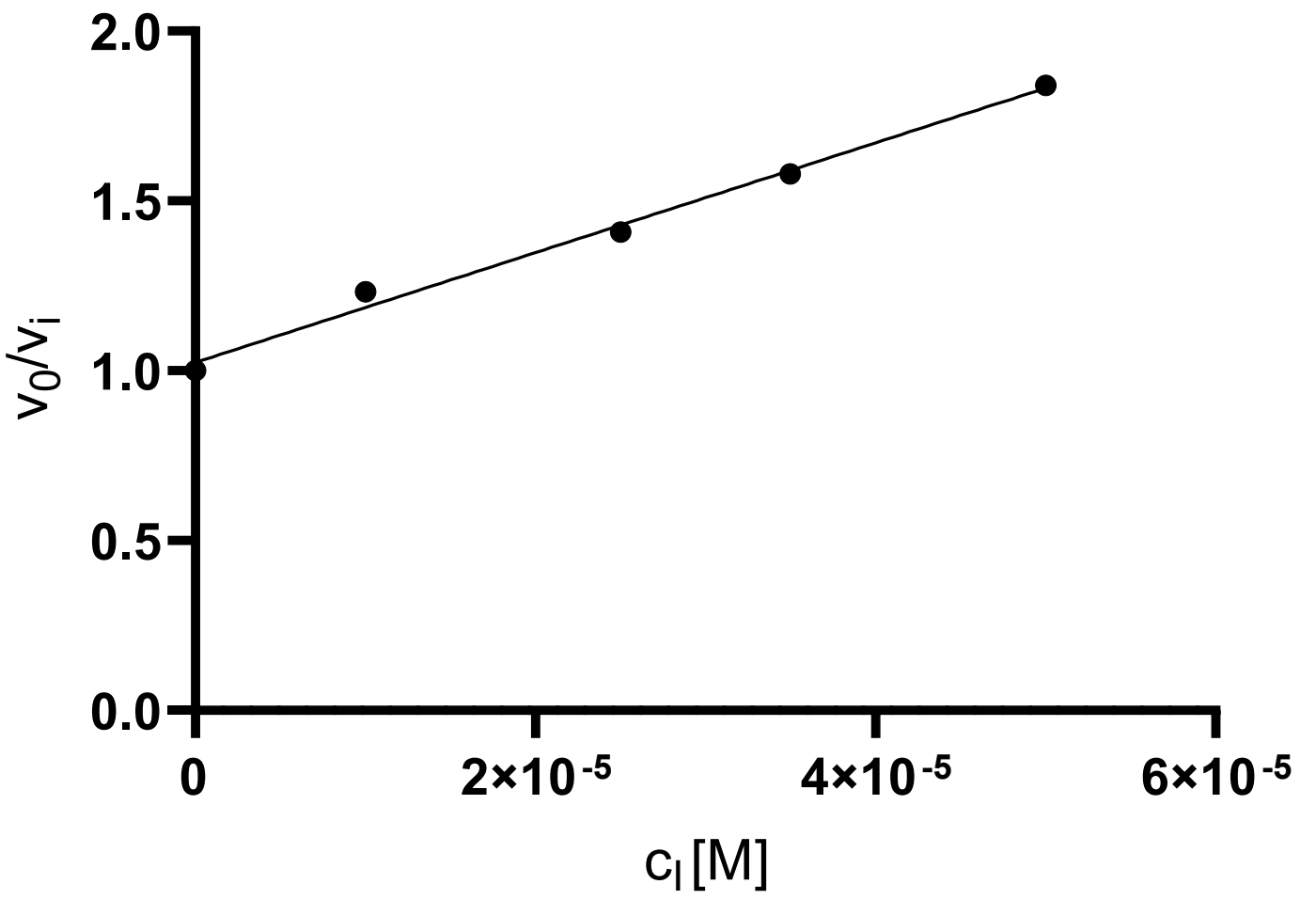

Figure **S4**. The dependence v_0/v_i vs. concentration of inhibitor **2a** inhibiting eeAChE.

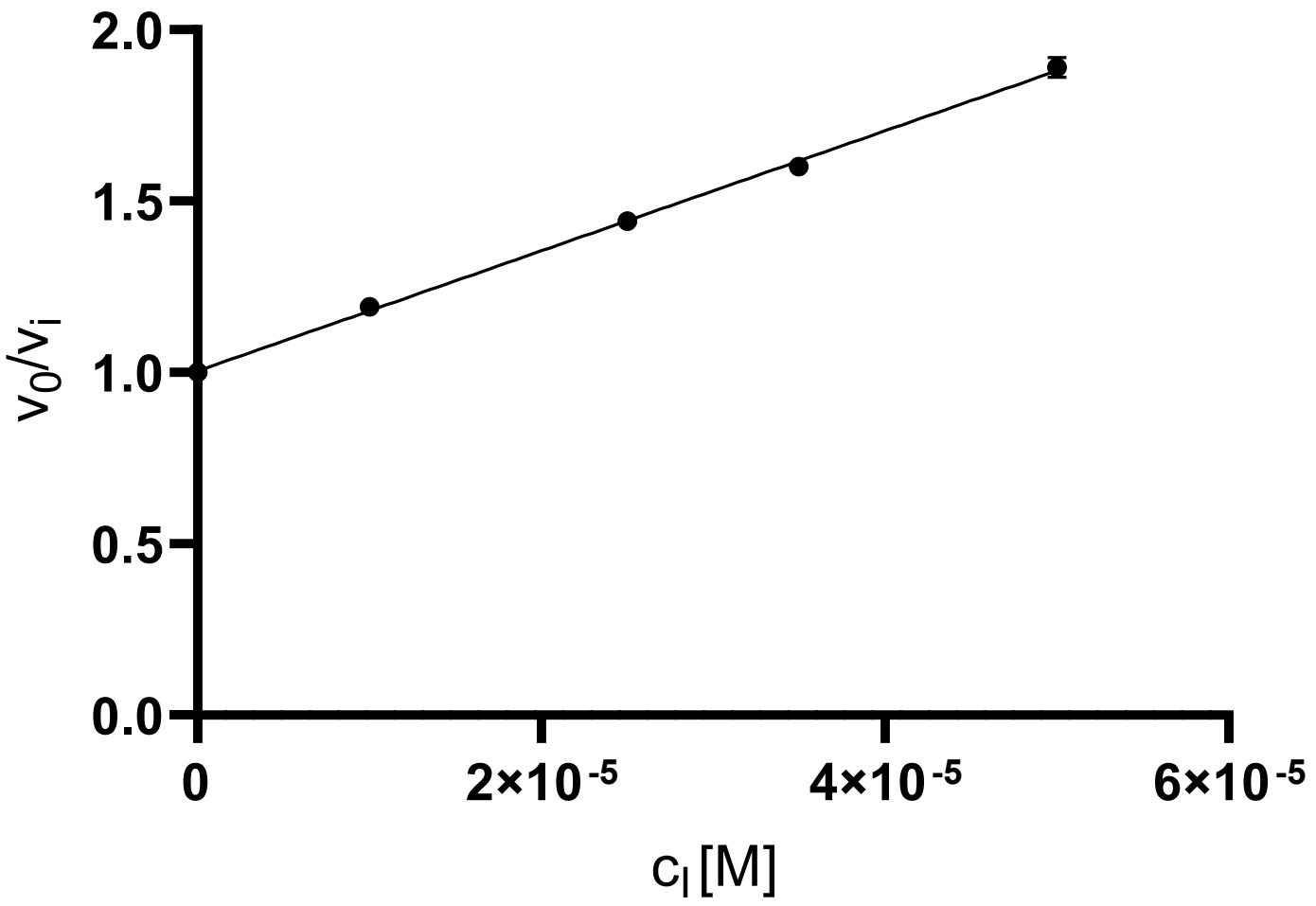

Figure **S5**. The dependence v_0/v_i vs. concentration of inhibitor **2a** inhibiting eqBChE.

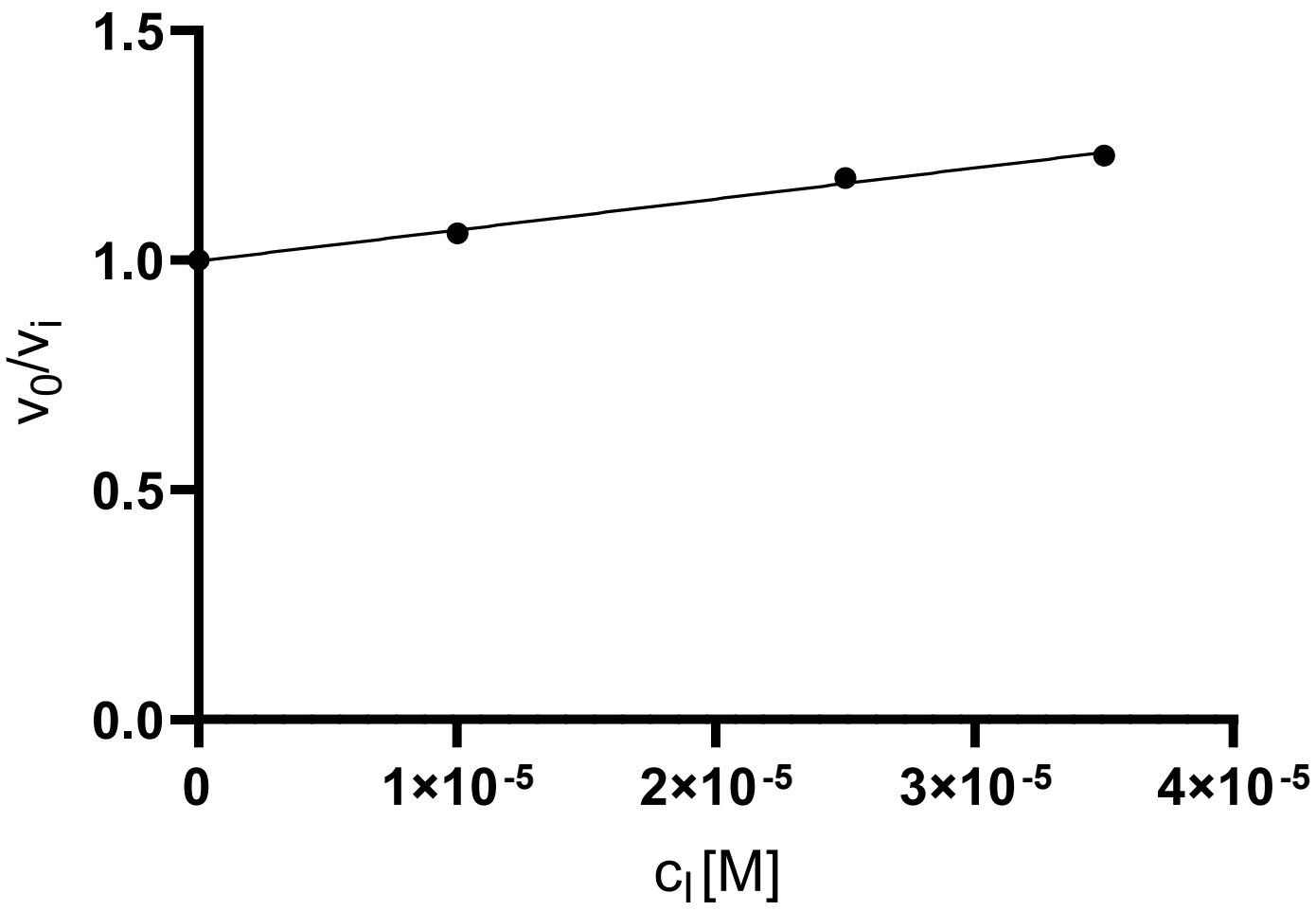

Figure **S6**. The dependence v_0/v_i vs. concentration of inhibitor **1b** inhibiting eeAChE.

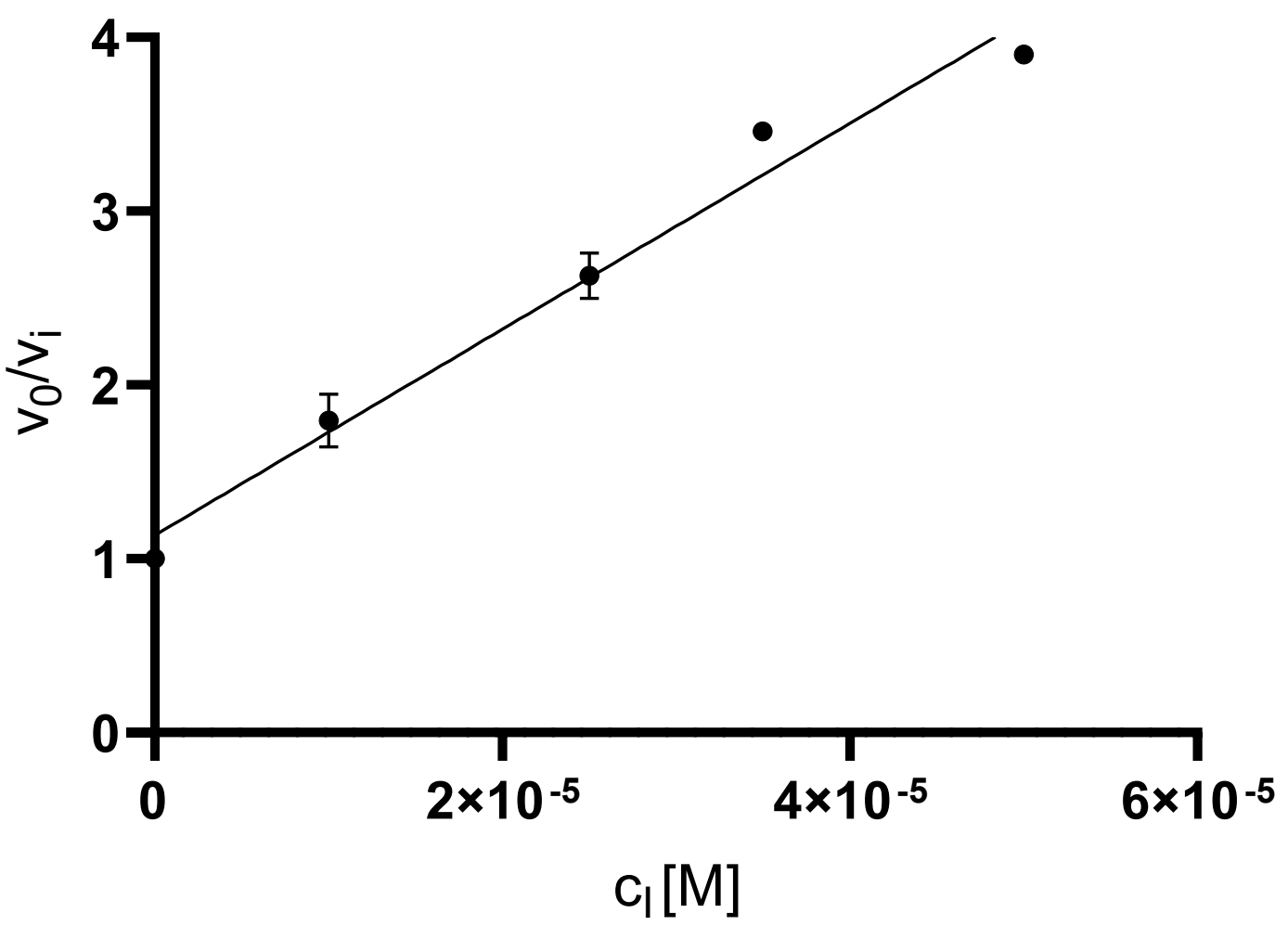

Figure **S7**. The dependence v_0/v_i vs. concentration of inhibitor **1b** inhibiting eqBChE.

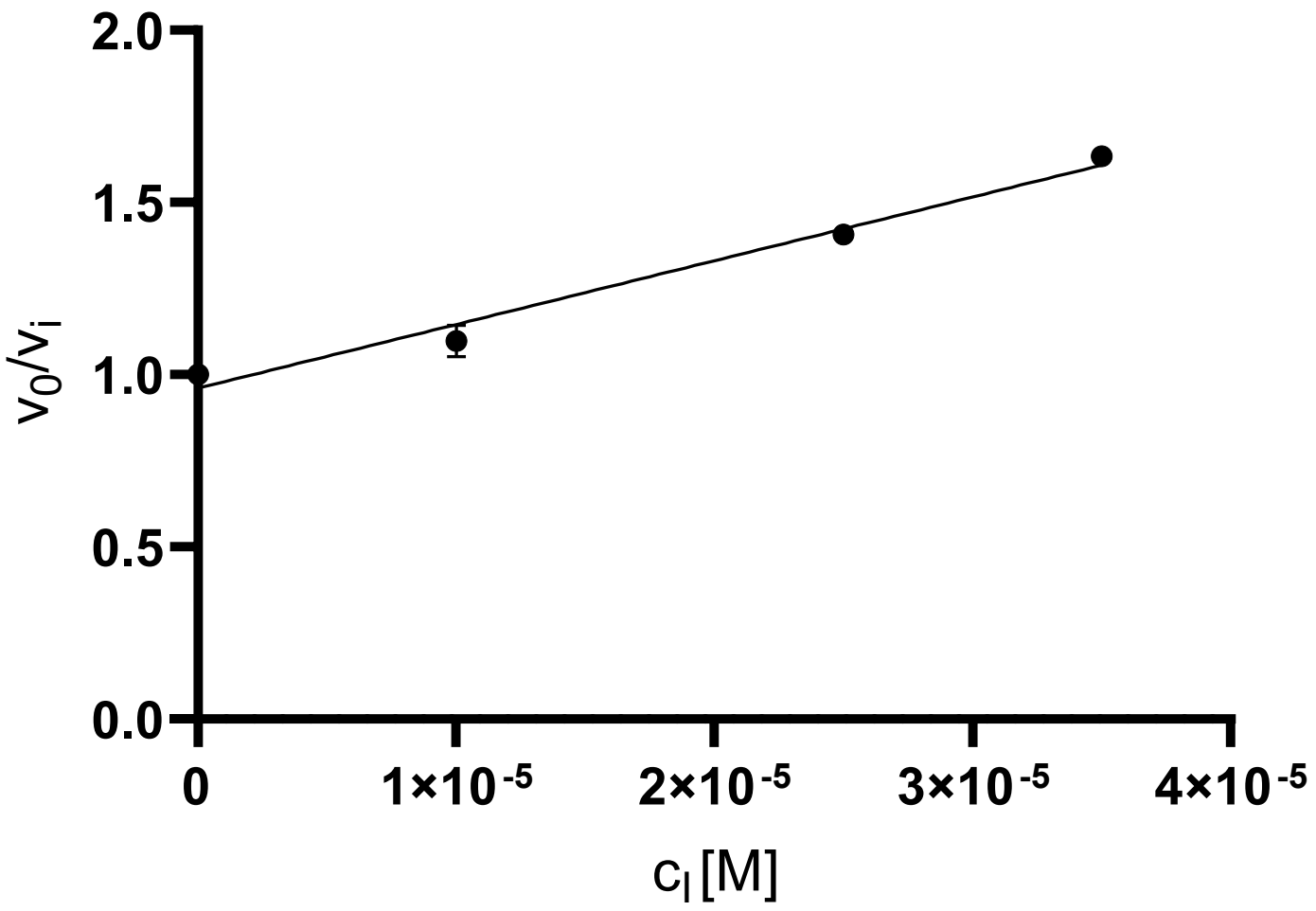

Figure **S8**. The dependence v_0/v_i vs. concentration of inhibitor **2b** inhibiting eeAChE.

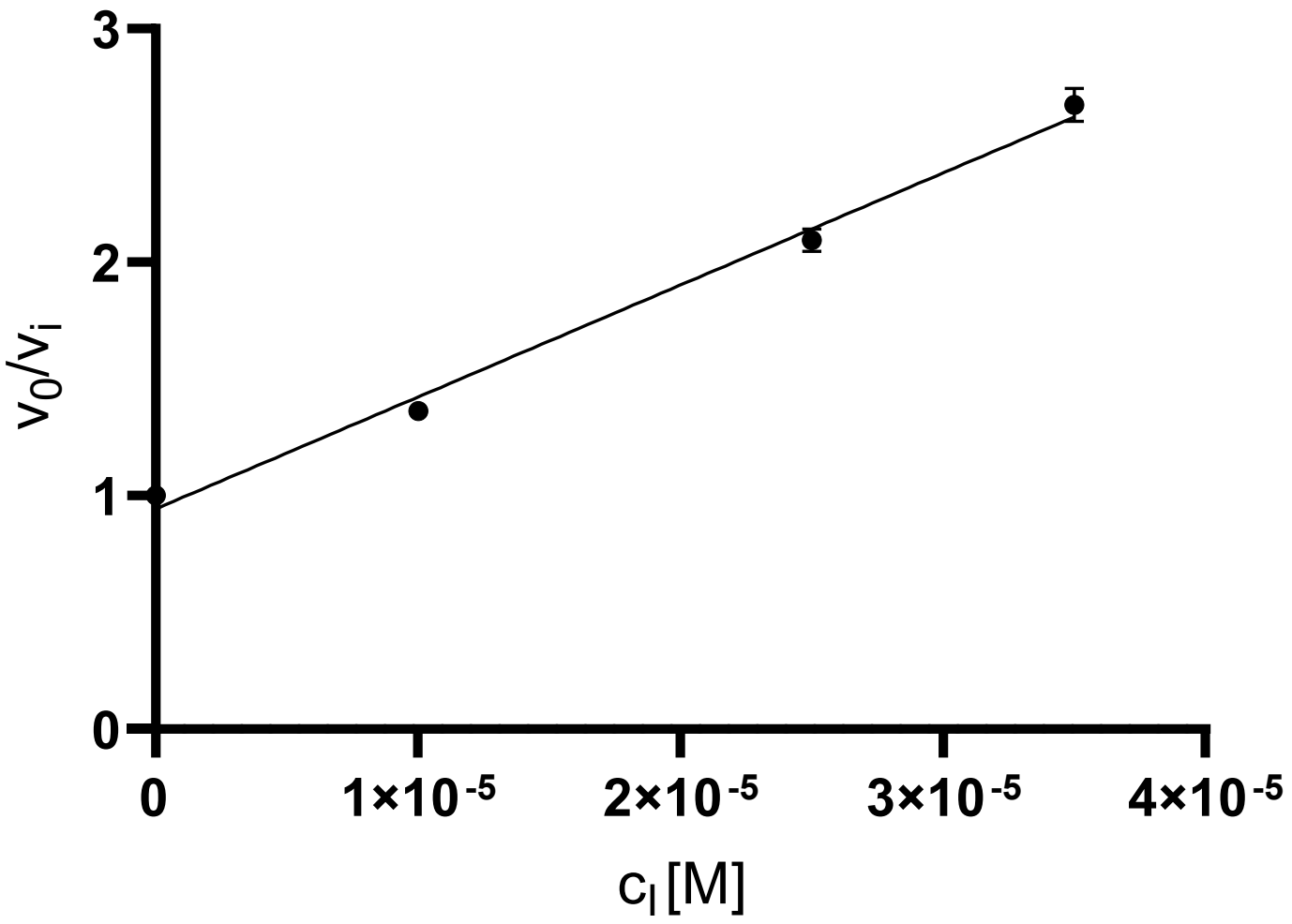

Figure **S9**. The dependence v_0/v_i vs. concentration of inhibitor **2b** inhibiting eqBChE.

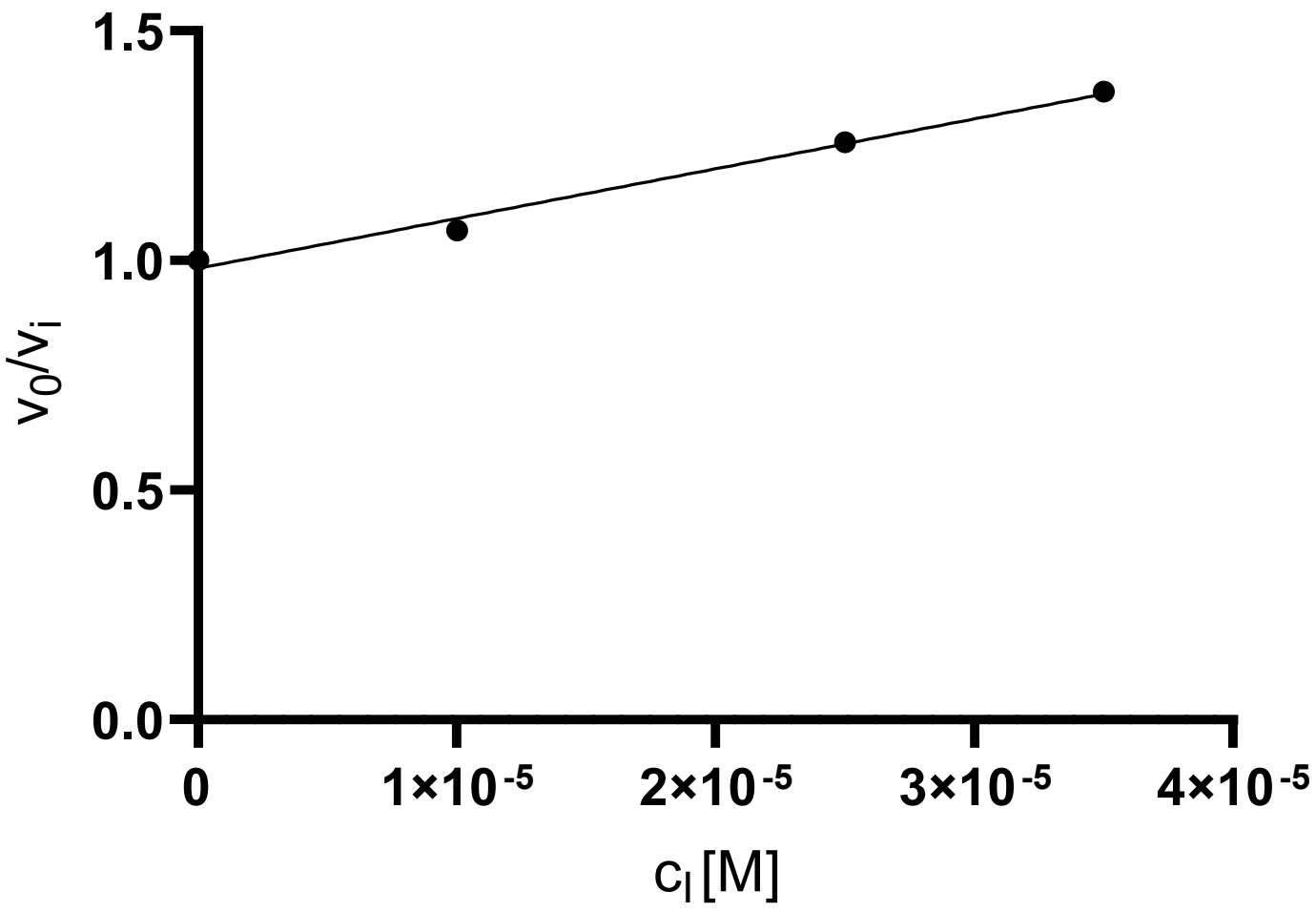

Figure **S10**. The dependence v_0/v_i vs. concentration of inhibitor **1c** inhibiting eeAChE

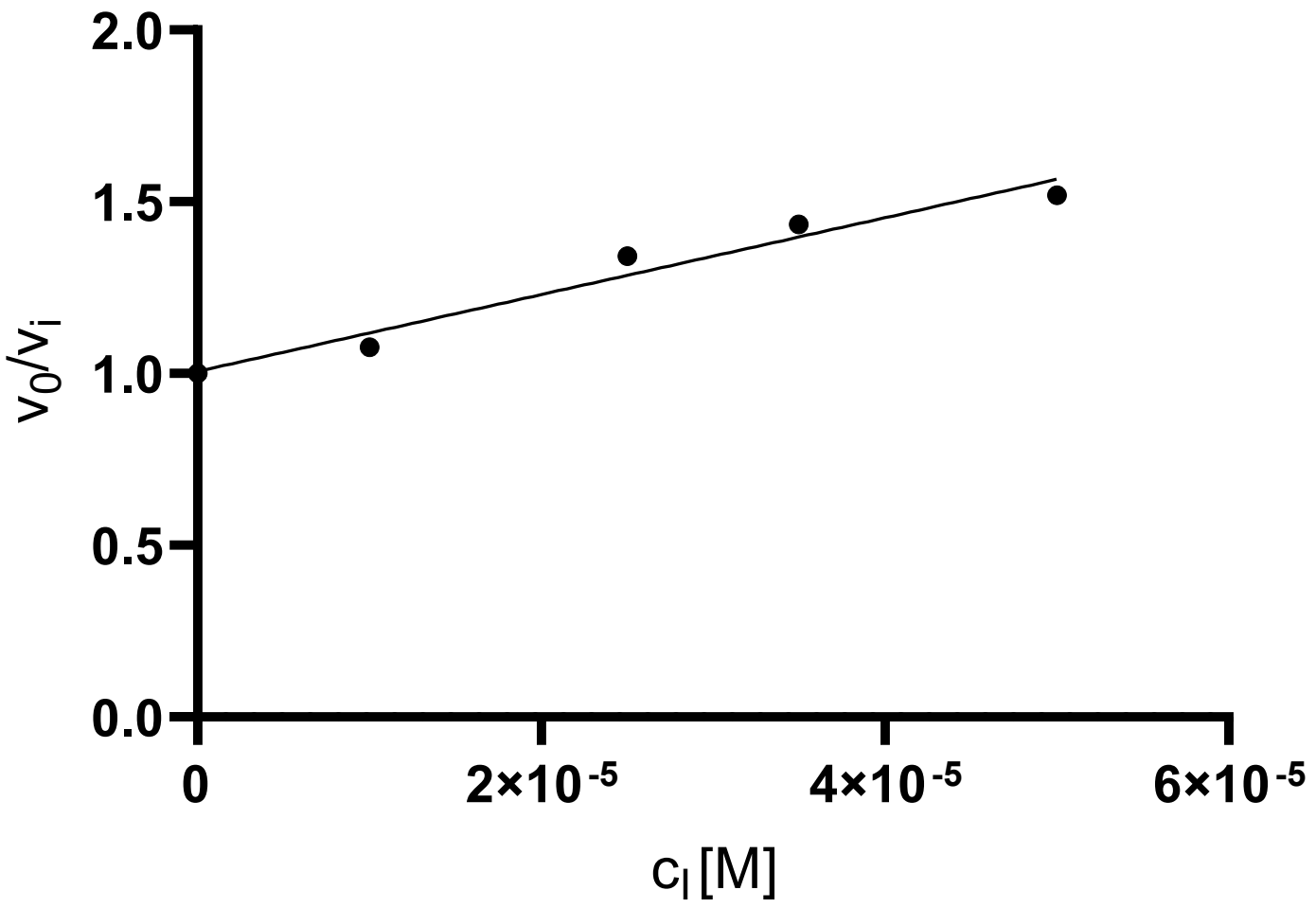

Figure **S11**. The dependence v_0/v_i vs. concentration of inhibitor **1c** inhibiting eqBChE.

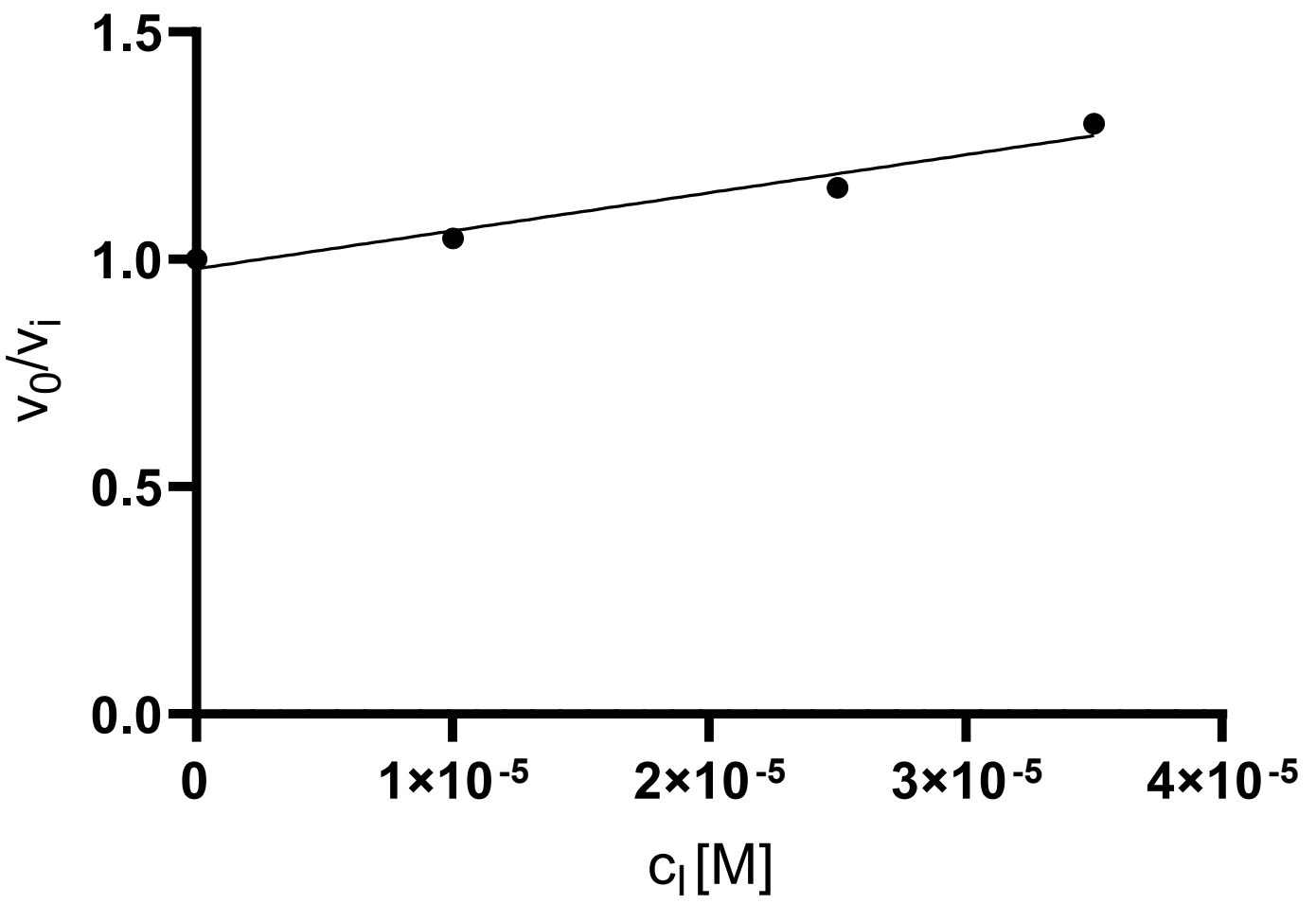

Figure **S12**. The dependence v_0/v_i vs. concentration of inhibitor **2c** inhibiting eeAChE.

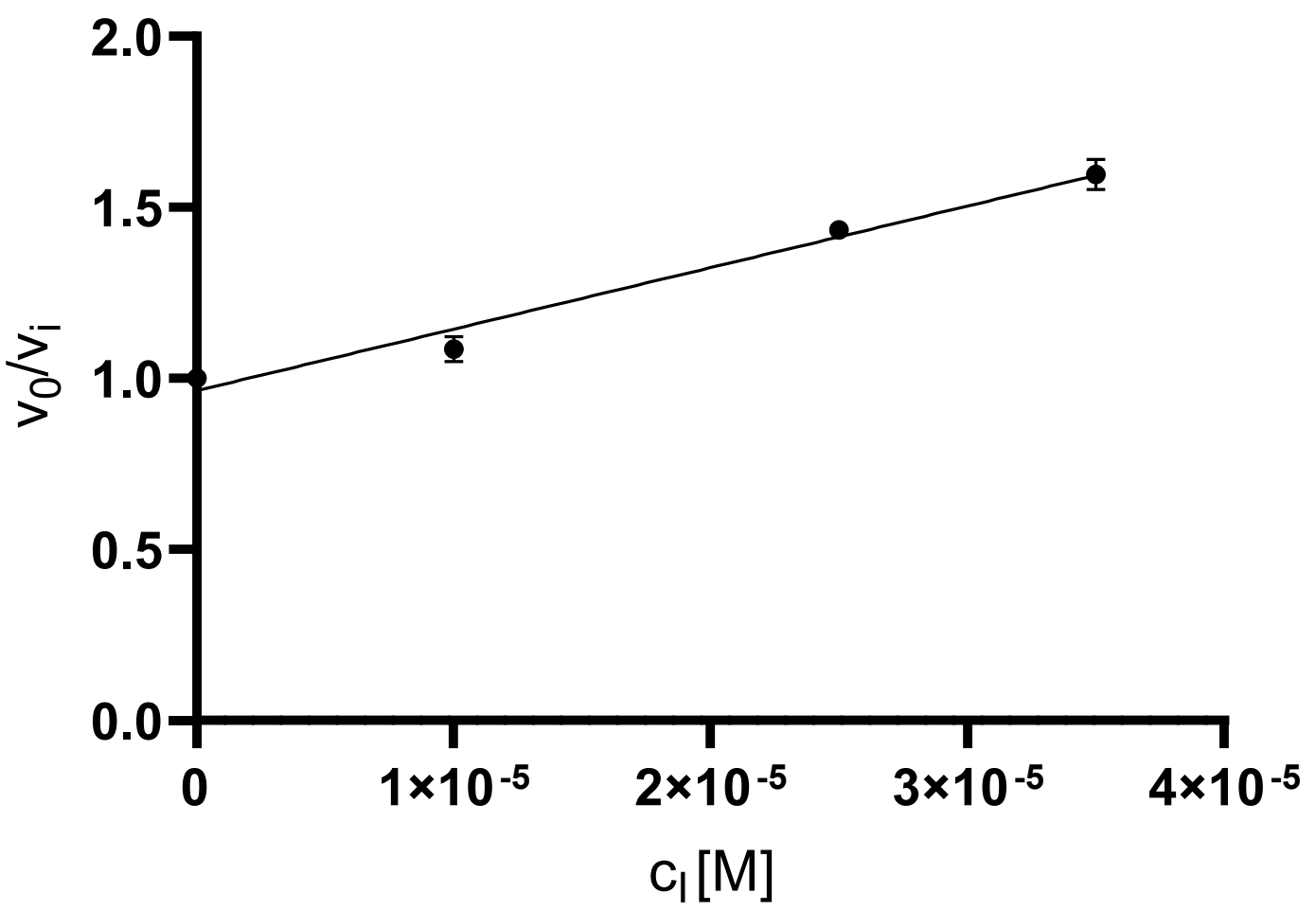

Figure **S13**. The dependence v_0/v_i vs. concentration of inhibitor **2c** inhibiting eqBChE.

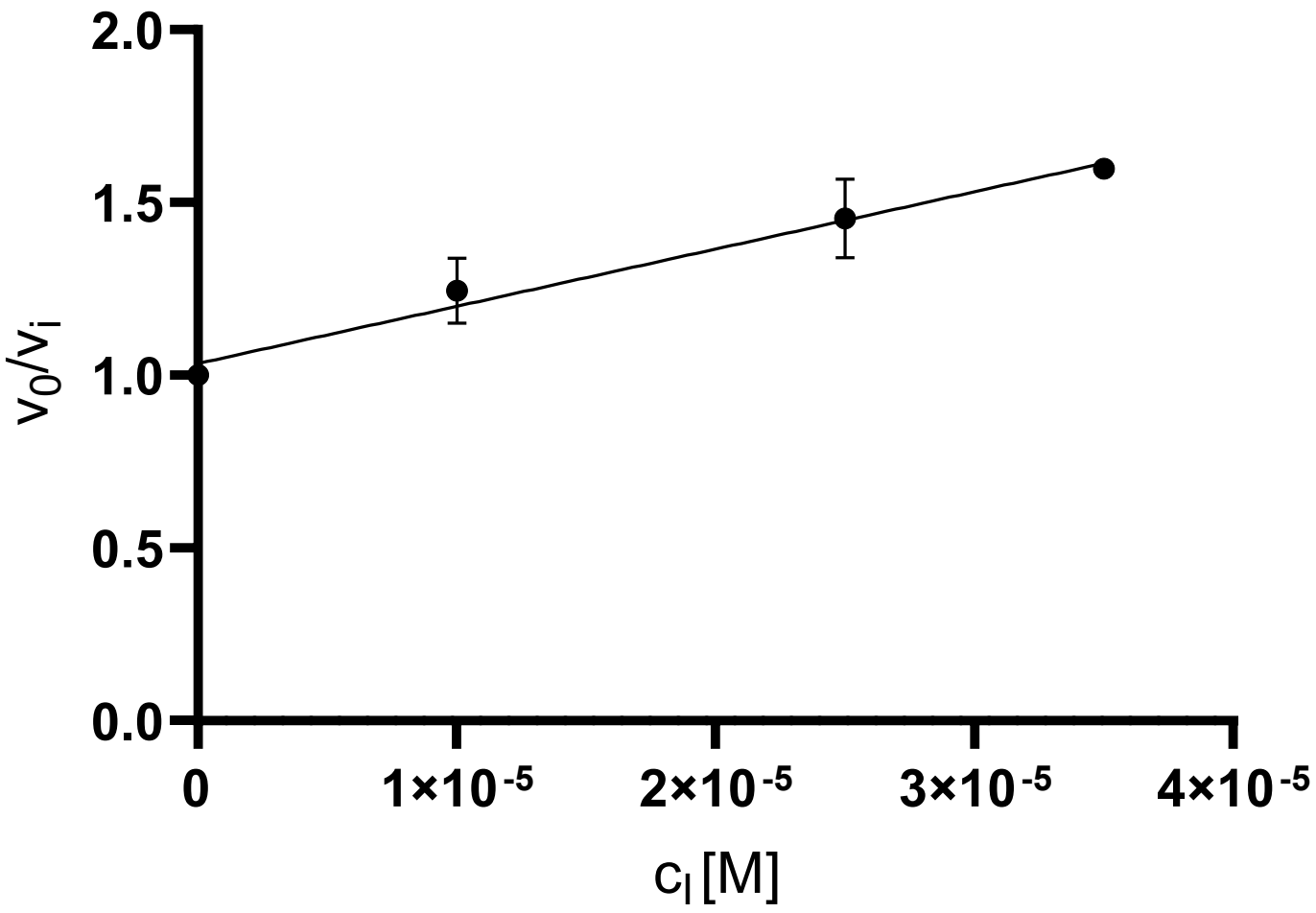

Figure **S14**. The dependence v_0/v_i vs. concentration of inhibitor **1d** inhibiting eeAChE.

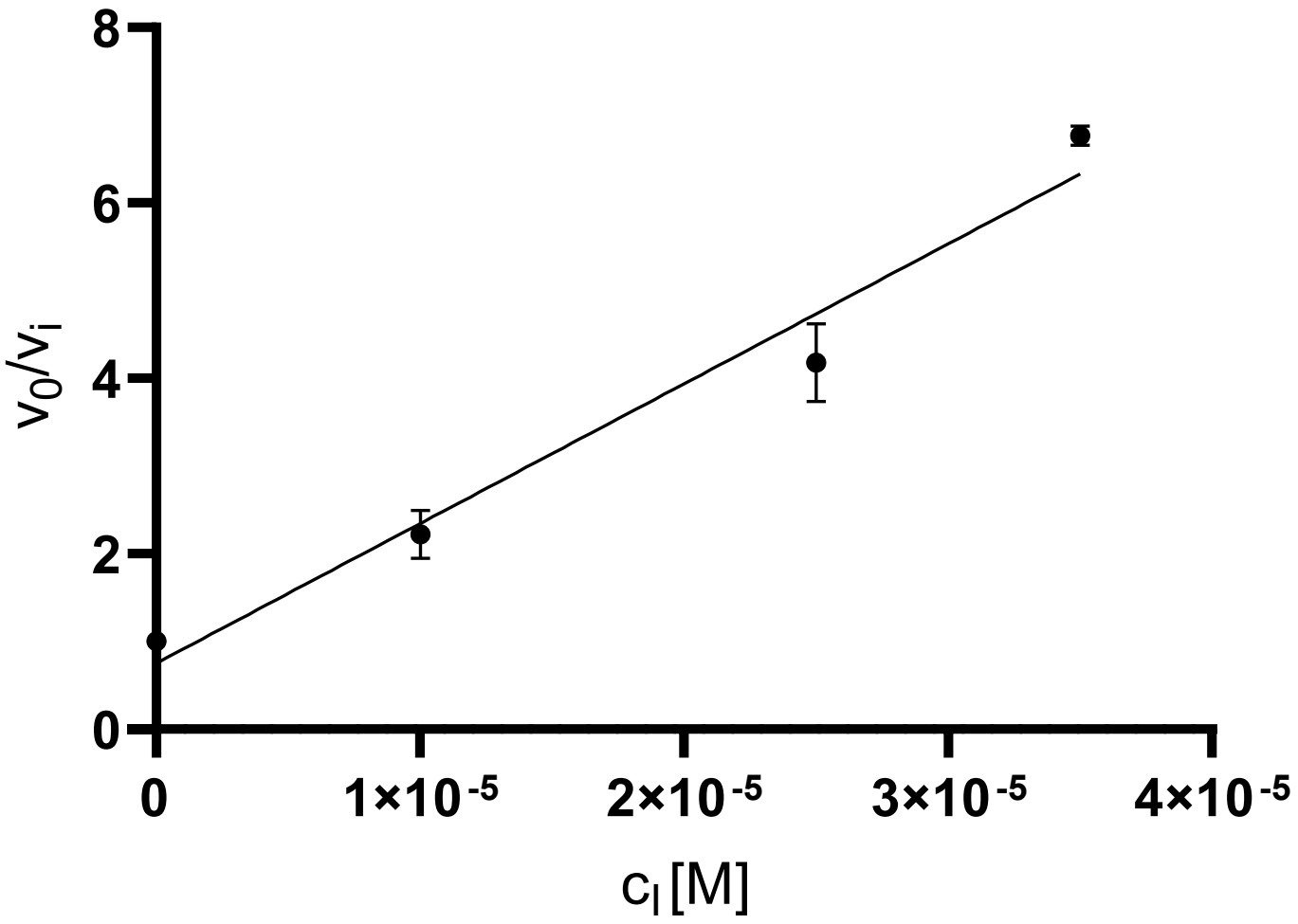

Figure **S15**. The dependence v_0/v_i vs. concentration of inhibitor **1d** inhibiting eqBChE.

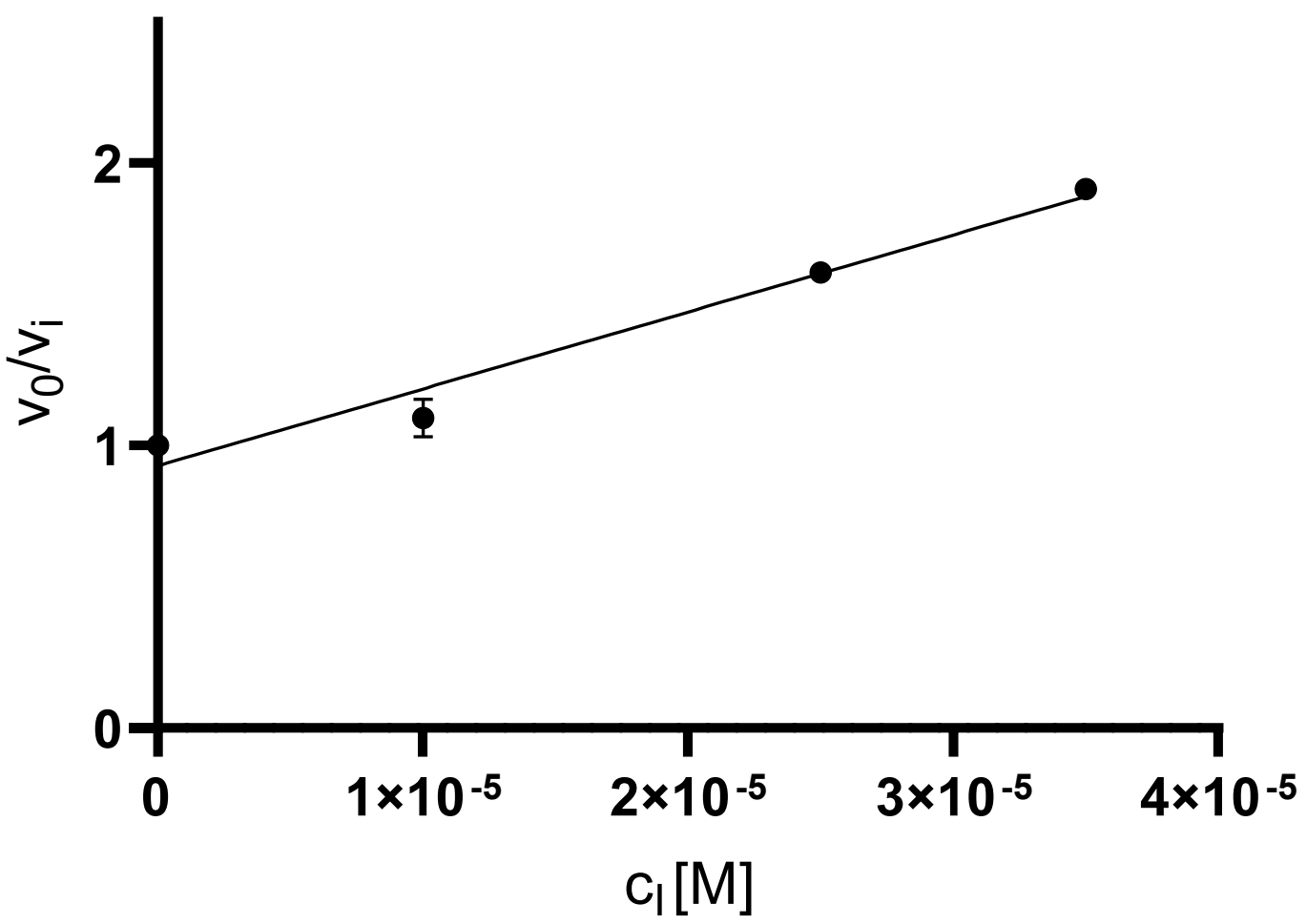

Figure **S16**. The dependence v_0/v_i vs. concentration of inhibitor **2d** inhibiting eeAChE.

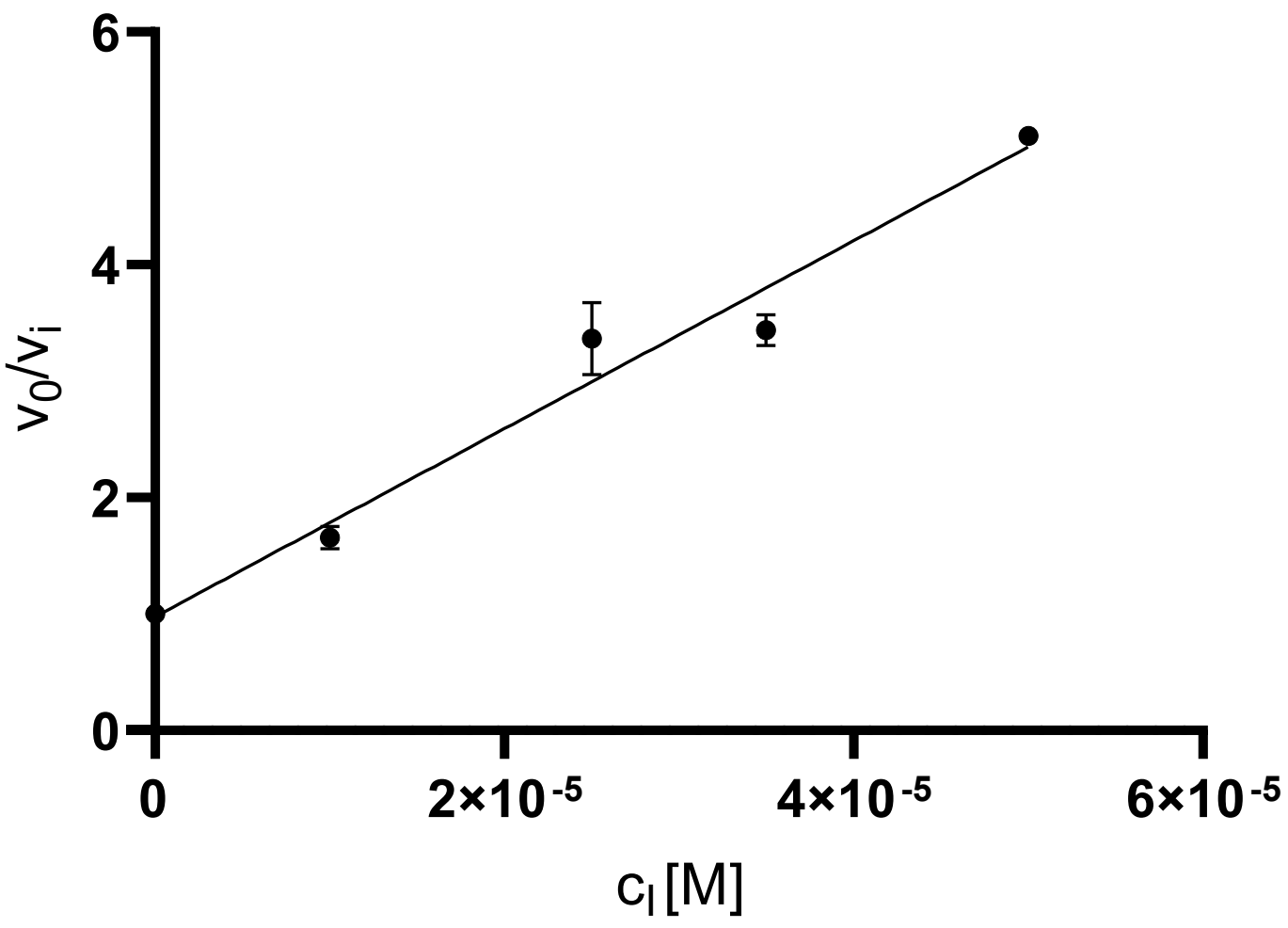

Figure **S17**. The dependence v_0/v_i vs. concentration of inhibitor **2d** inhibiting eqBChE.

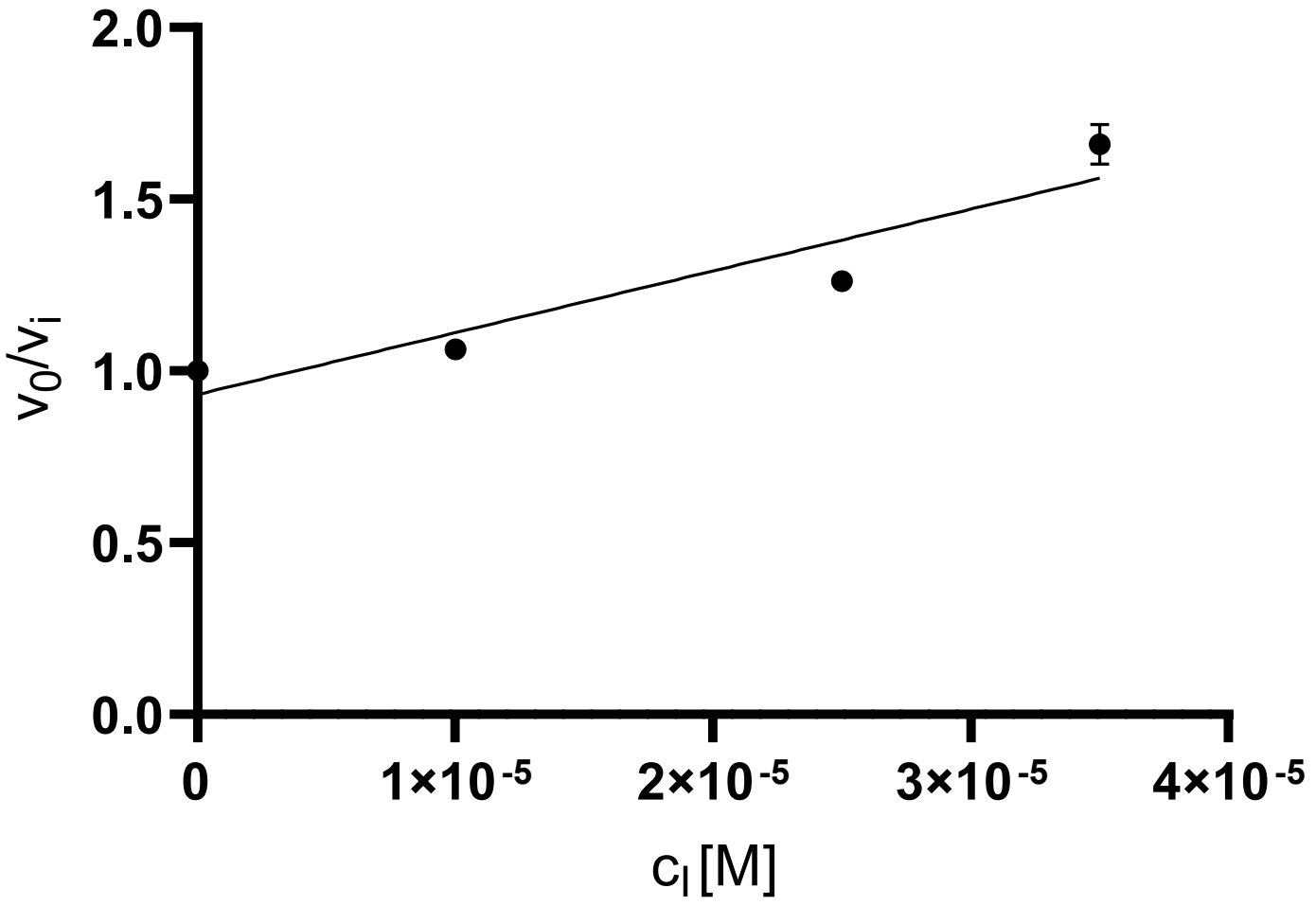

Figure **S18**. The dependence v_0/v_i vs. concentration of inhibitor **1e** inhibiting eeAChE.

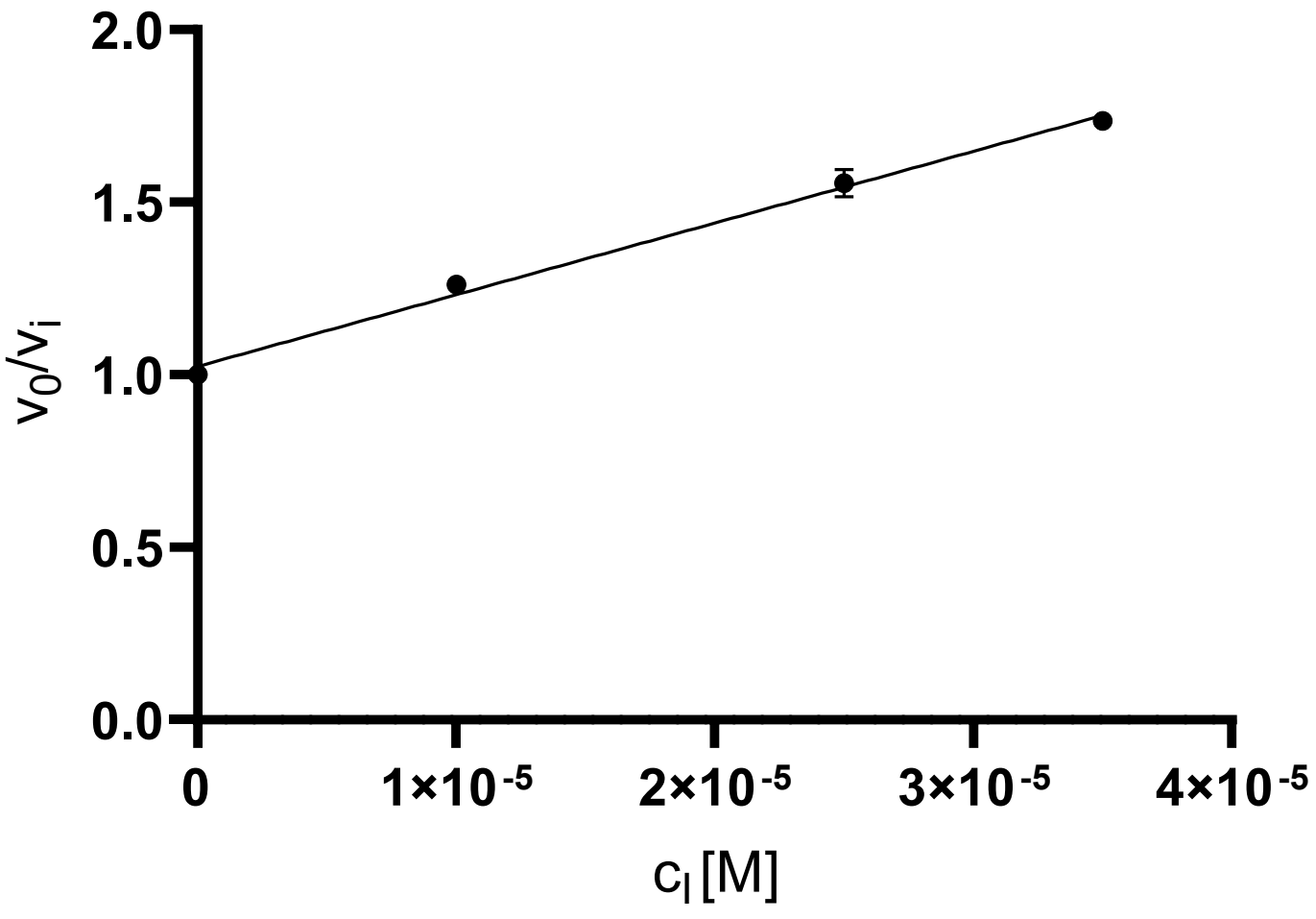

Figure **S19**. The dependence v_0/v_i vs. concentration of inhibitor **1e** inhibiting eqBChE.

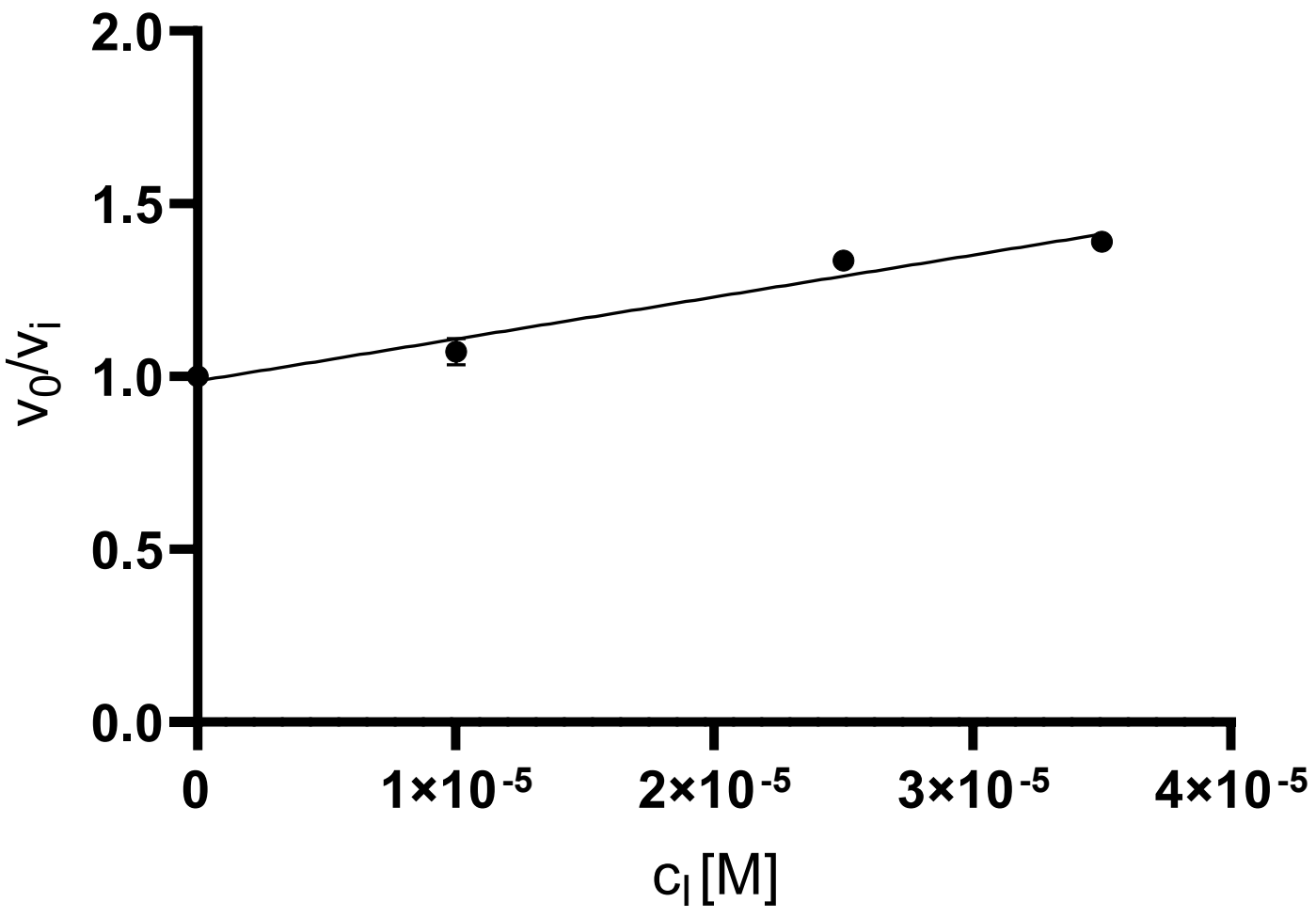

Figure **S20**. The dependence v_0/v_i vs. concentration of inhibitor **2e** inhibiting eeAChE.

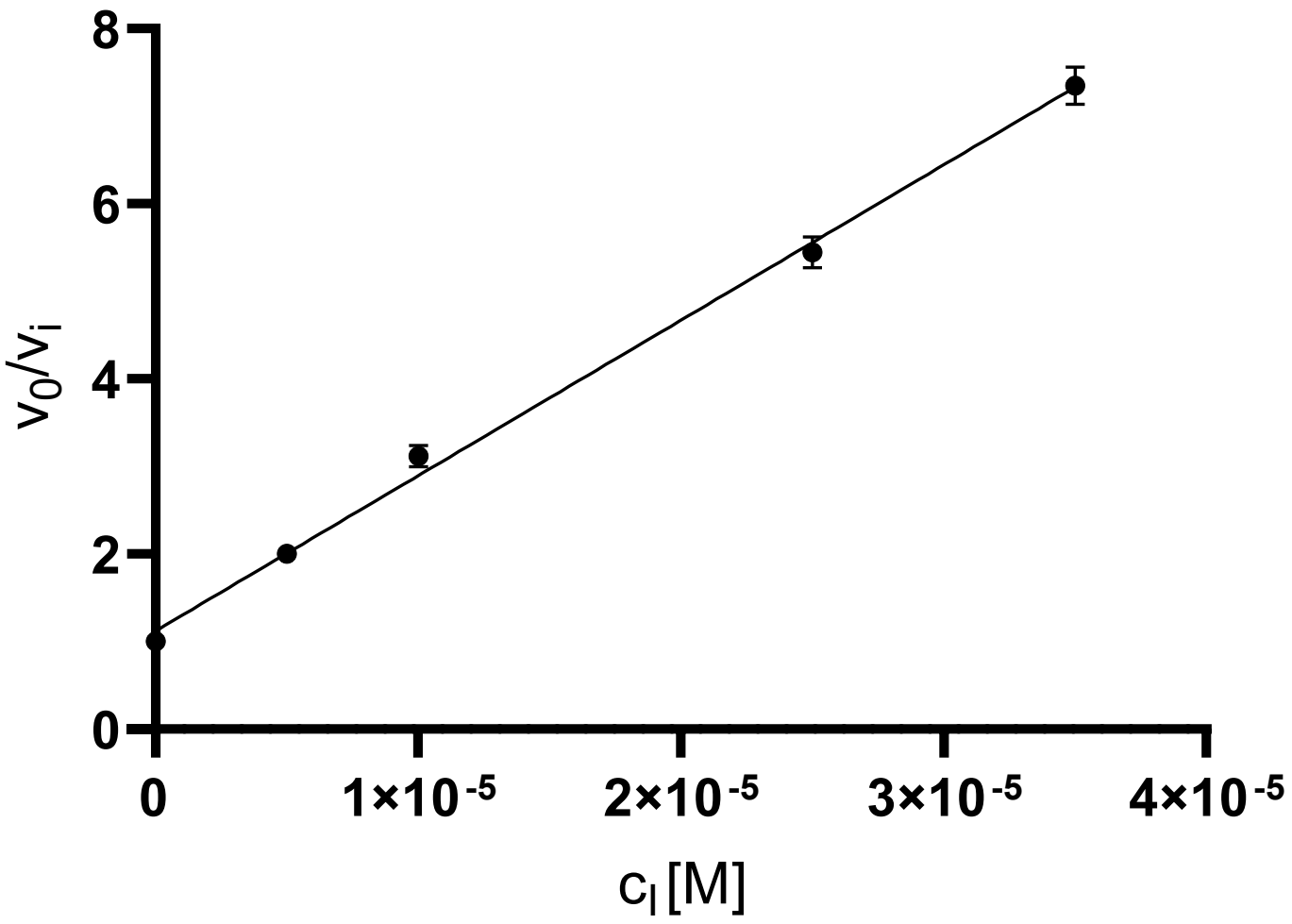

Figure **S21**. The dependence v_0/v_i vs. concentration of inhibitor **2e** inhibiting eqBChE.

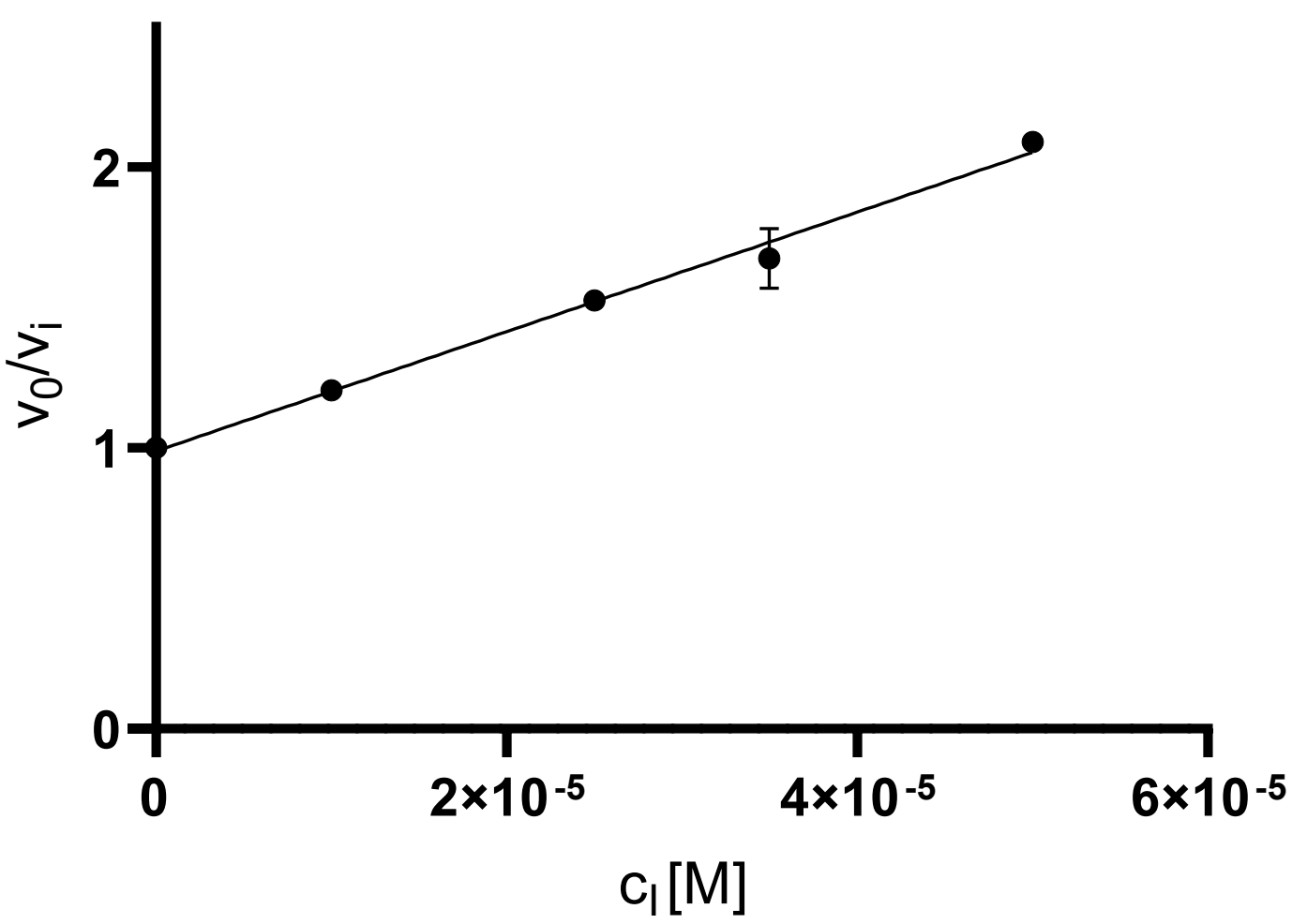

Figure **S22**. The dependence v_0/v_i vs. concentration of inhibitor **1f** inhibiting eeAChE.

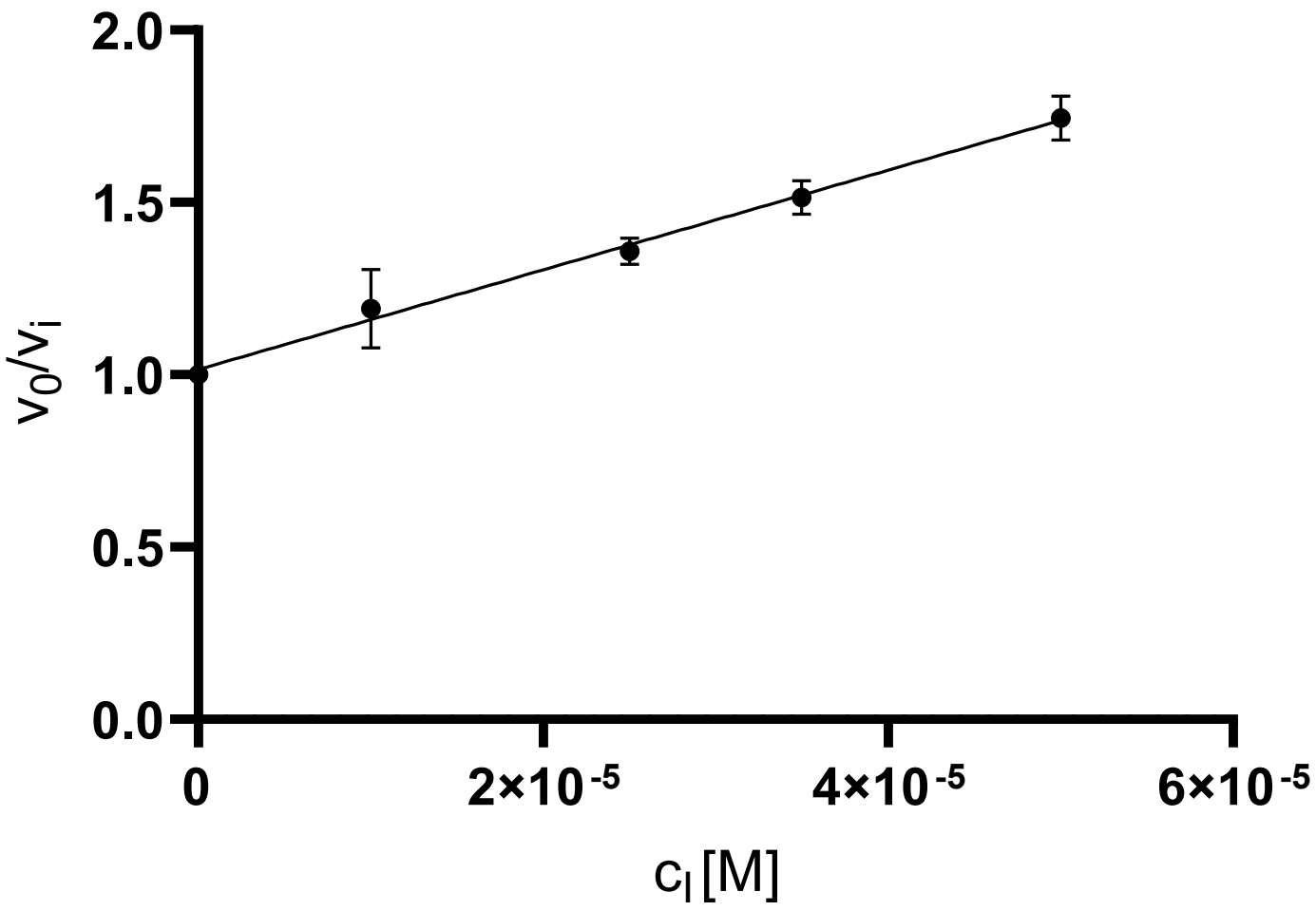

Figure **S23**. The dependence v_0/v_i vs. concentration of inhibitor **1f** inhibiting eqBChE.

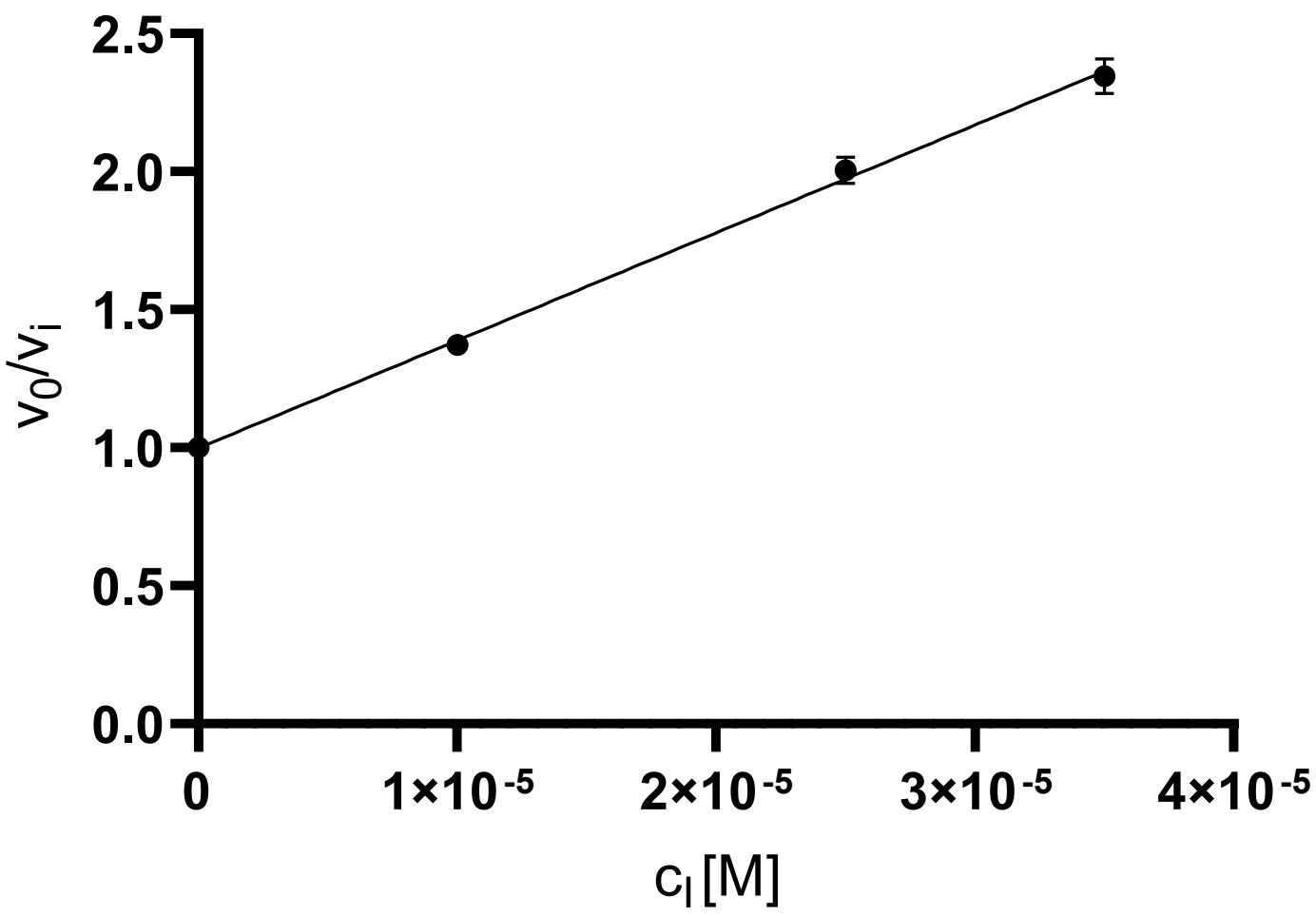

Figure **S24**. The dependence v_0/v_i vs. concentration of inhibitor **2f** inhibiting eeAChE.

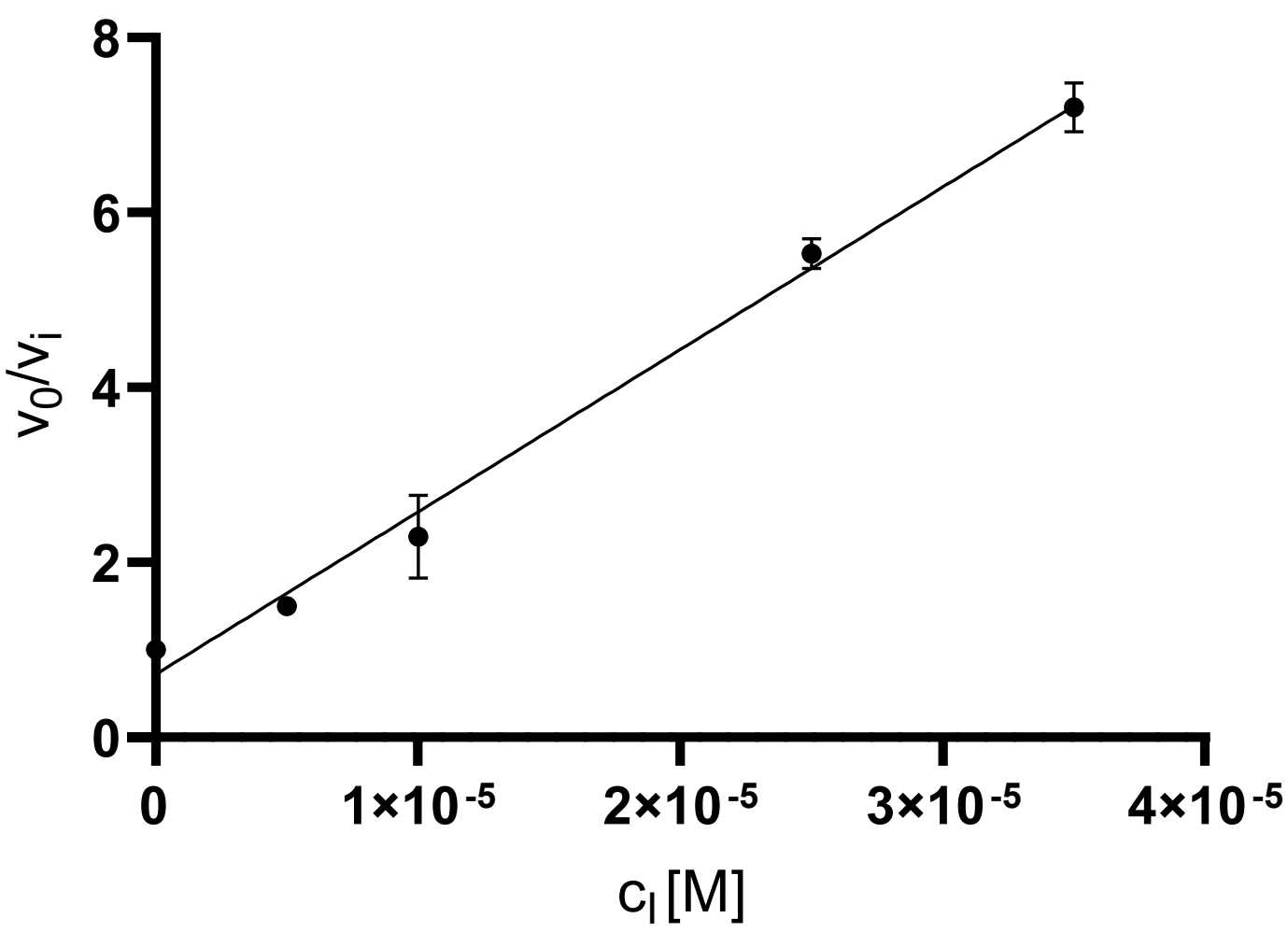

Figure **S25**. The dependence v_0/v_i vs. concentration of inhibitor **2f** inhibiting eqBChE.

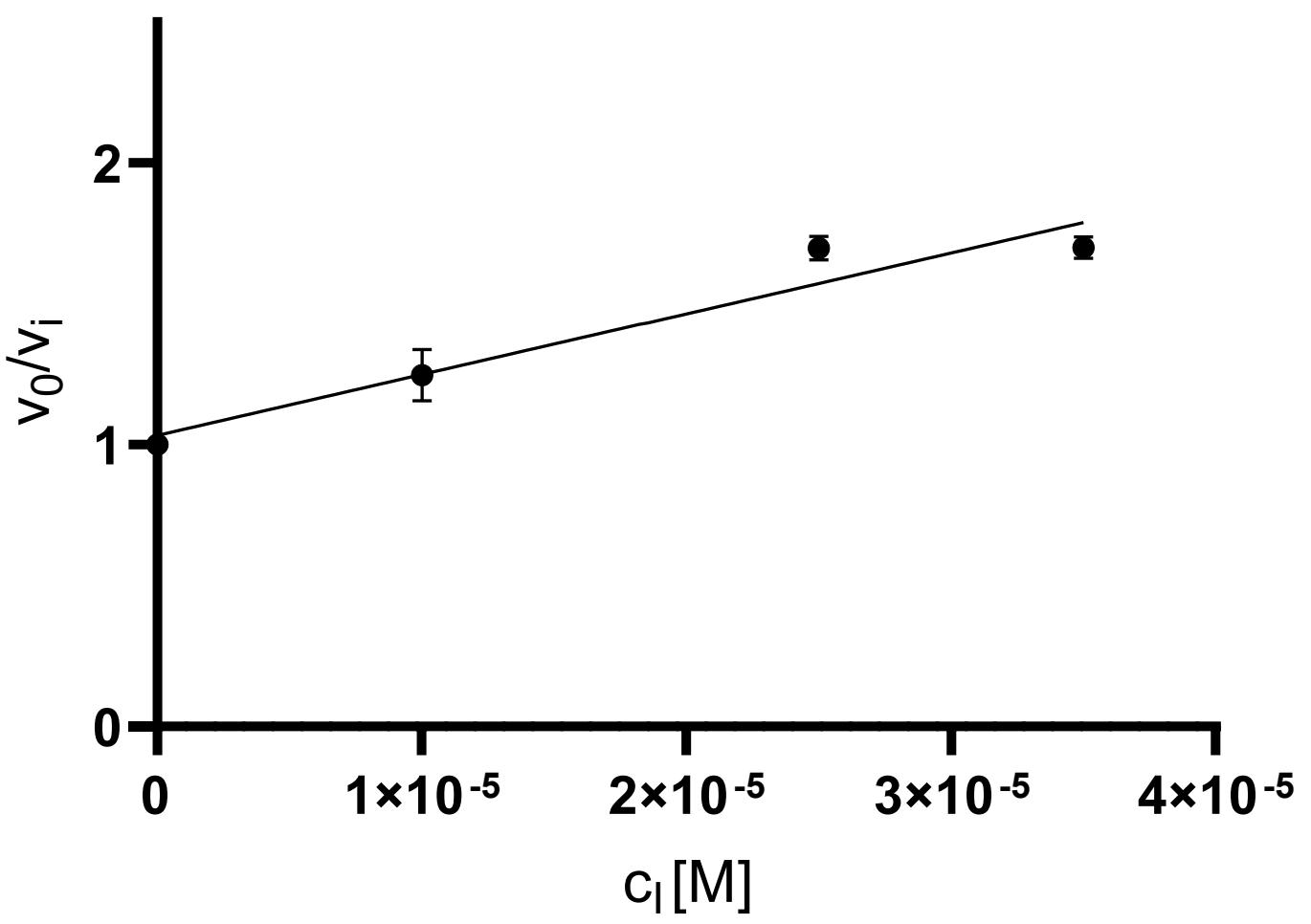

Figure **S26**. The dependence v_0/v_i vs. concentration of inhibitor **1g** inhibiting eeAChE.

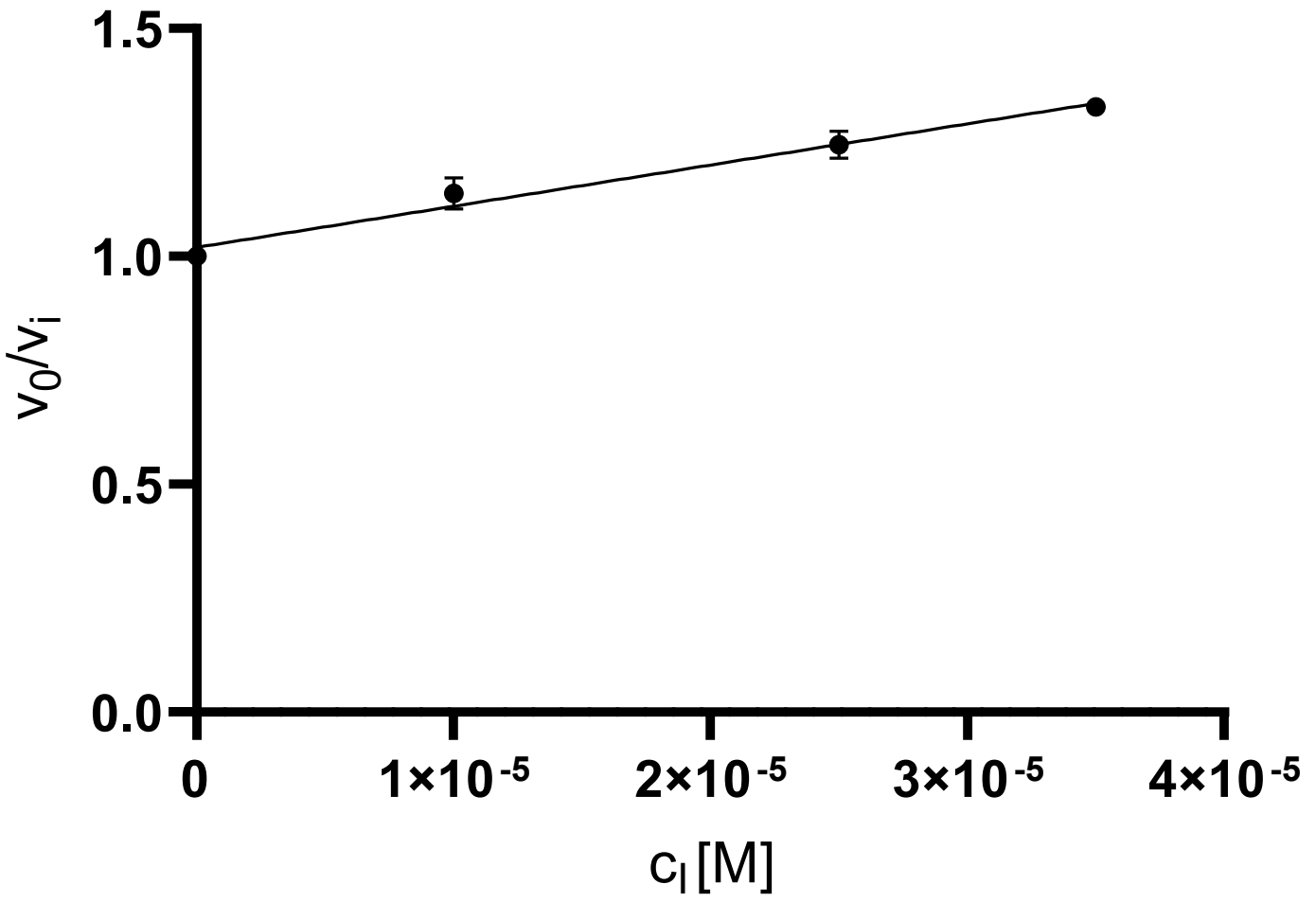

Figure **S27**. The dependence v_0/v_i vs. concentration of inhibitor **1g** inhibiting eqBChE.

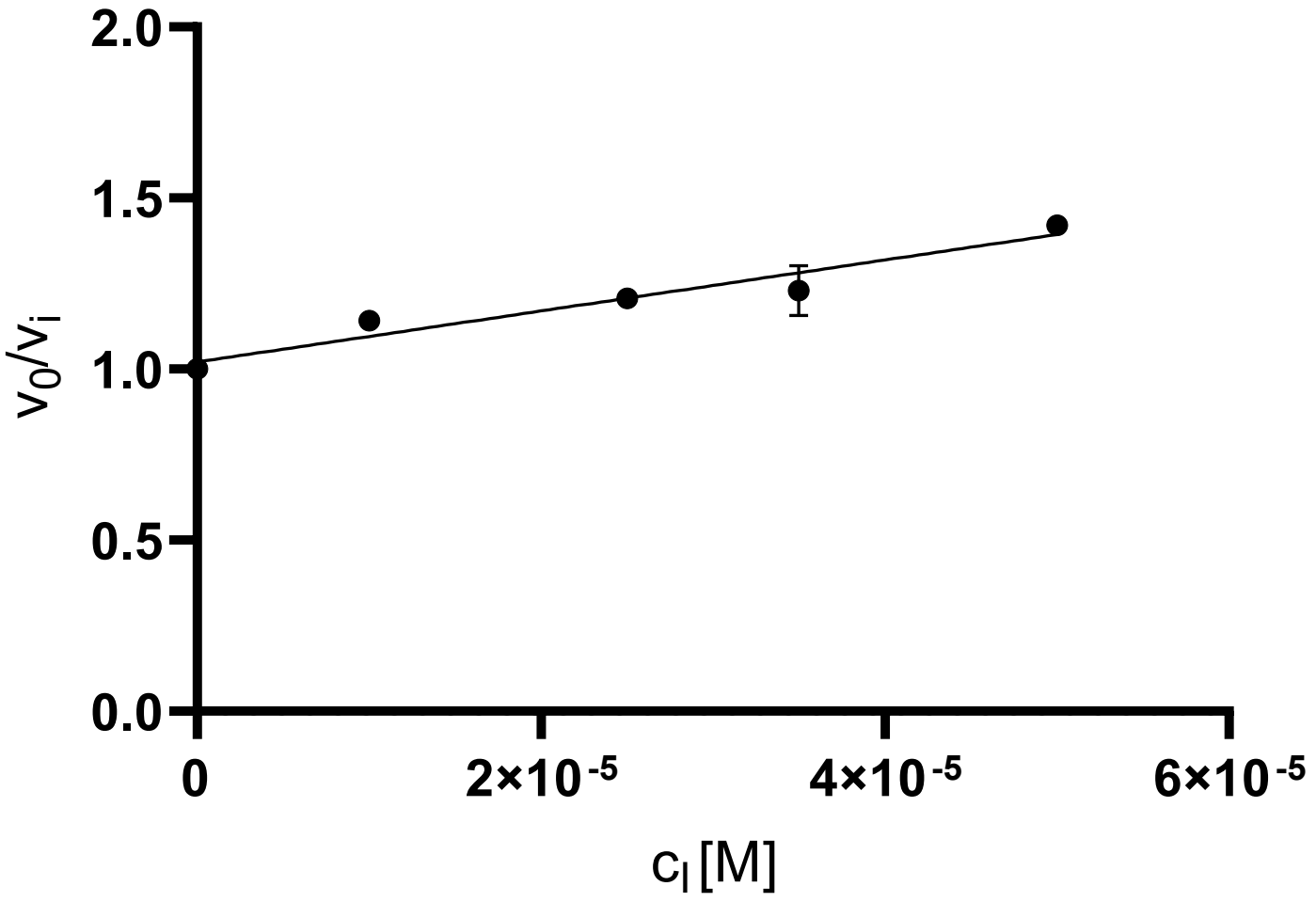

Figure **S28**. The dependence v_0/v_i vs. concentration of inhibitor **2g** inhibiting eeAChE.

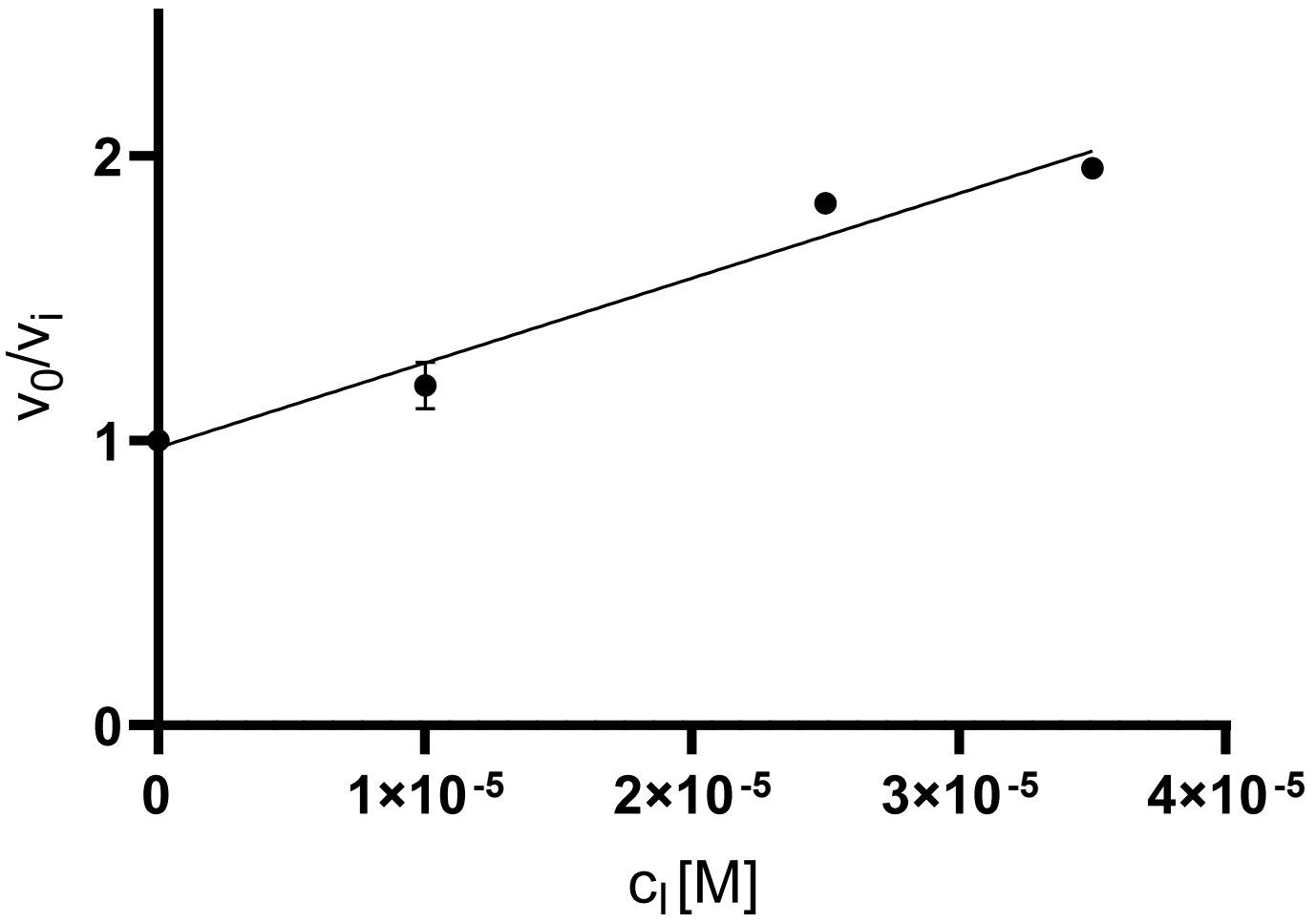

Figure **S29**. The dependence v_0/v_i vs. concentration of inhibitor **1h** inhibiting eeAChE.

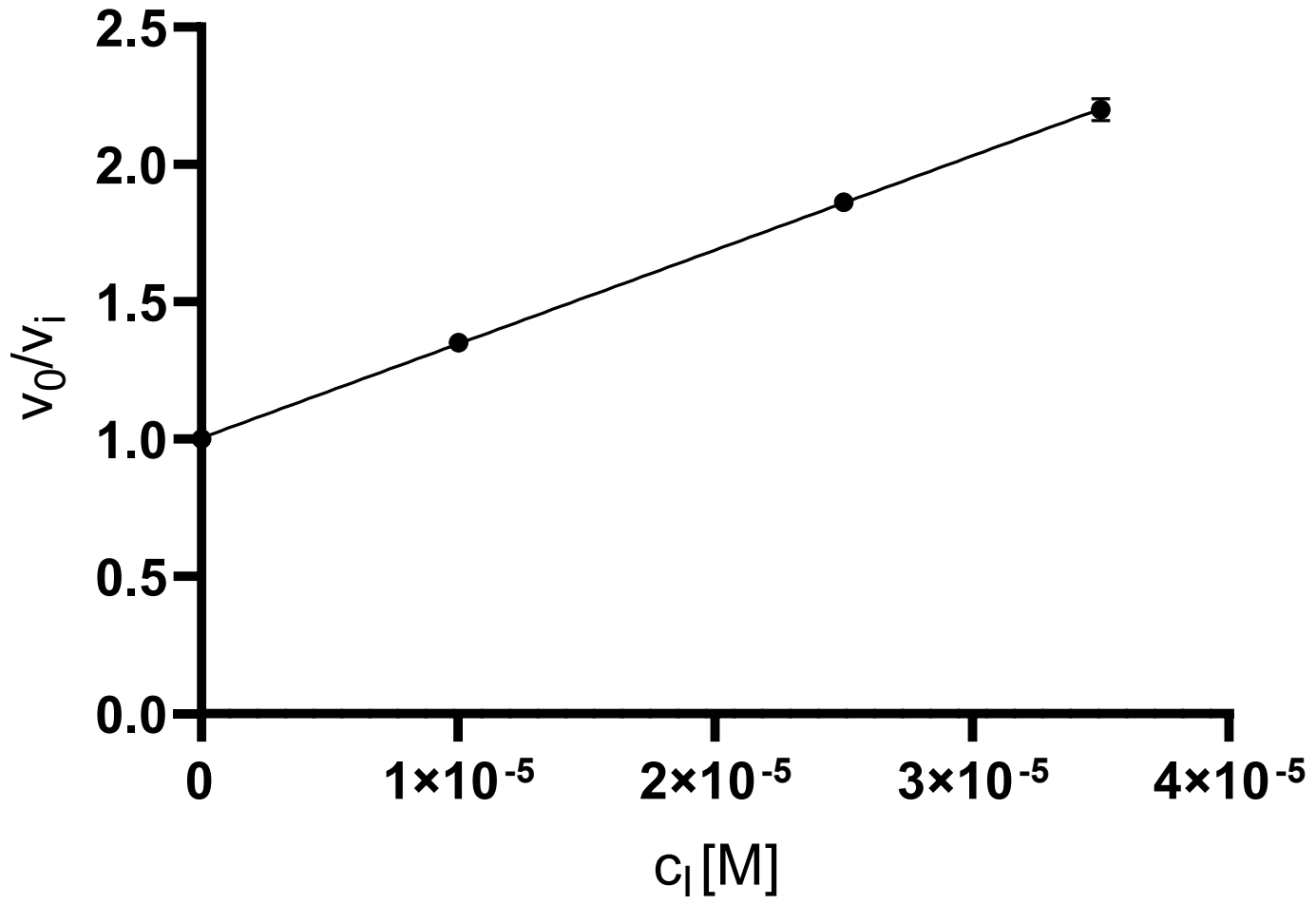

Figure **S30**. The dependence v_0/v_i vs. concentration of inhibitor **1h** inhibiting eqBChE.

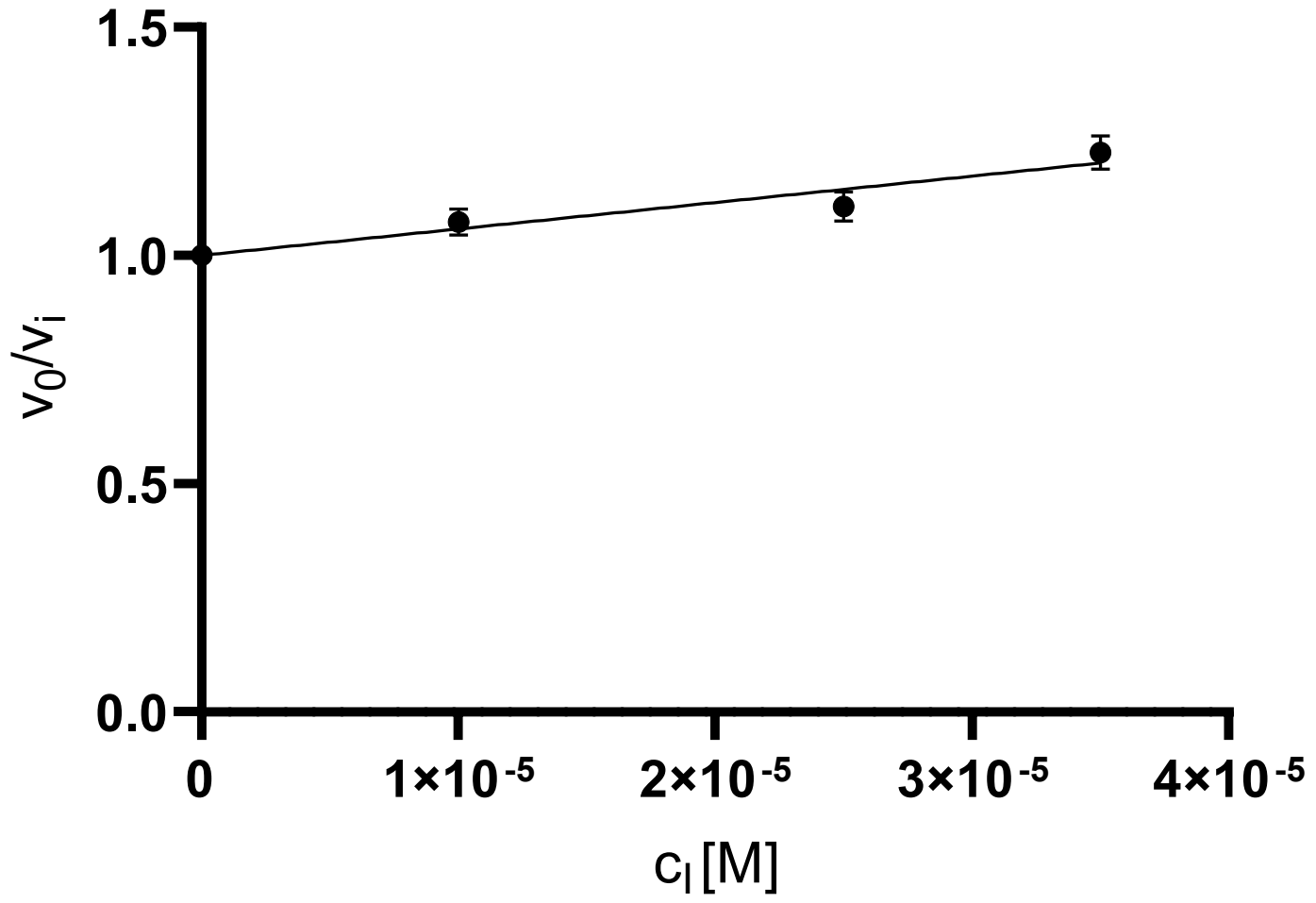

Figure **S31**. The dependence v_0/v_i vs. concentration of inhibitor **2h** inhibiting eeAChE.

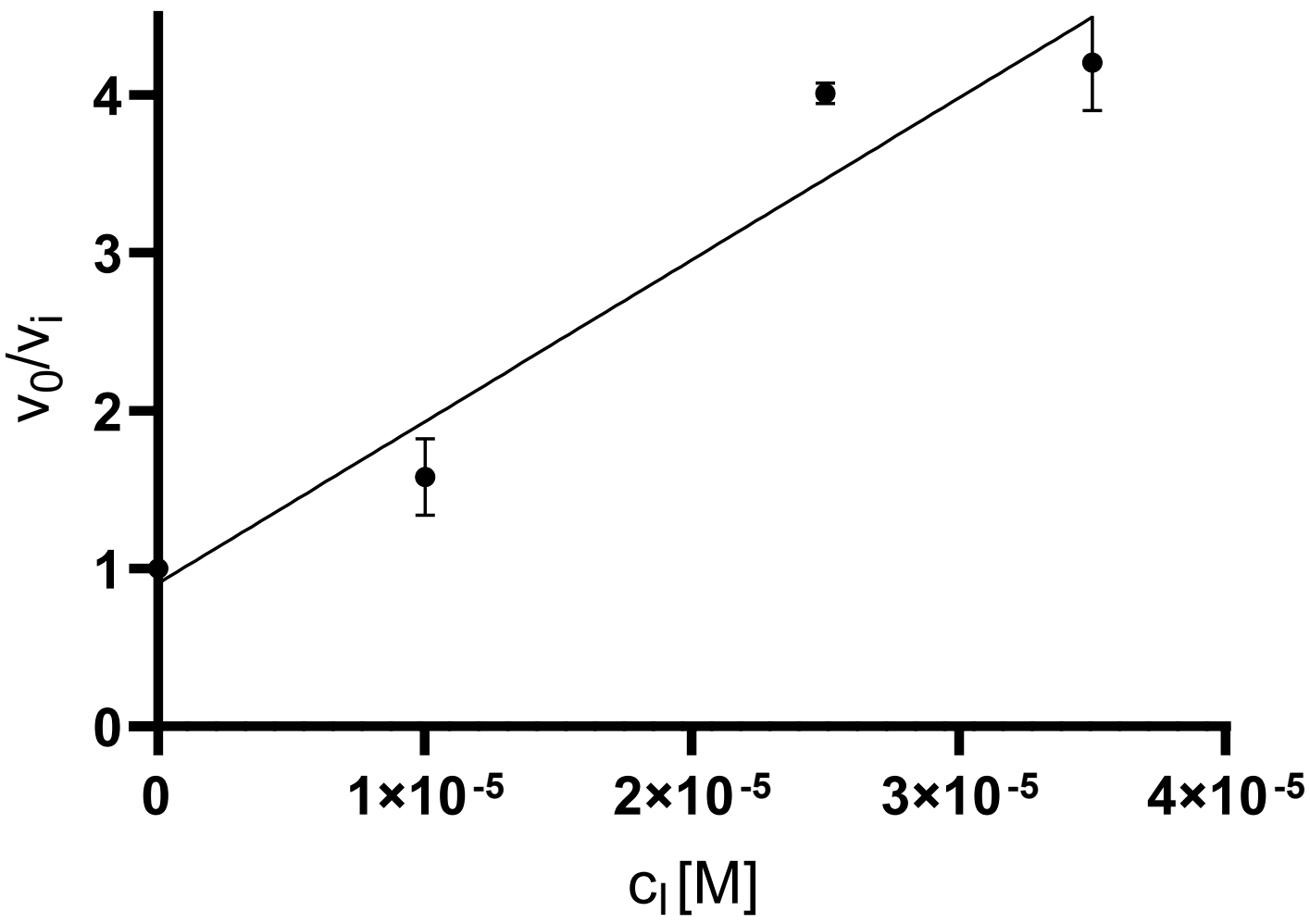

Figure **S32**. The dependence v_0/v_i vs. concentration of inhibitor **2h** inhibiting eqBChE.

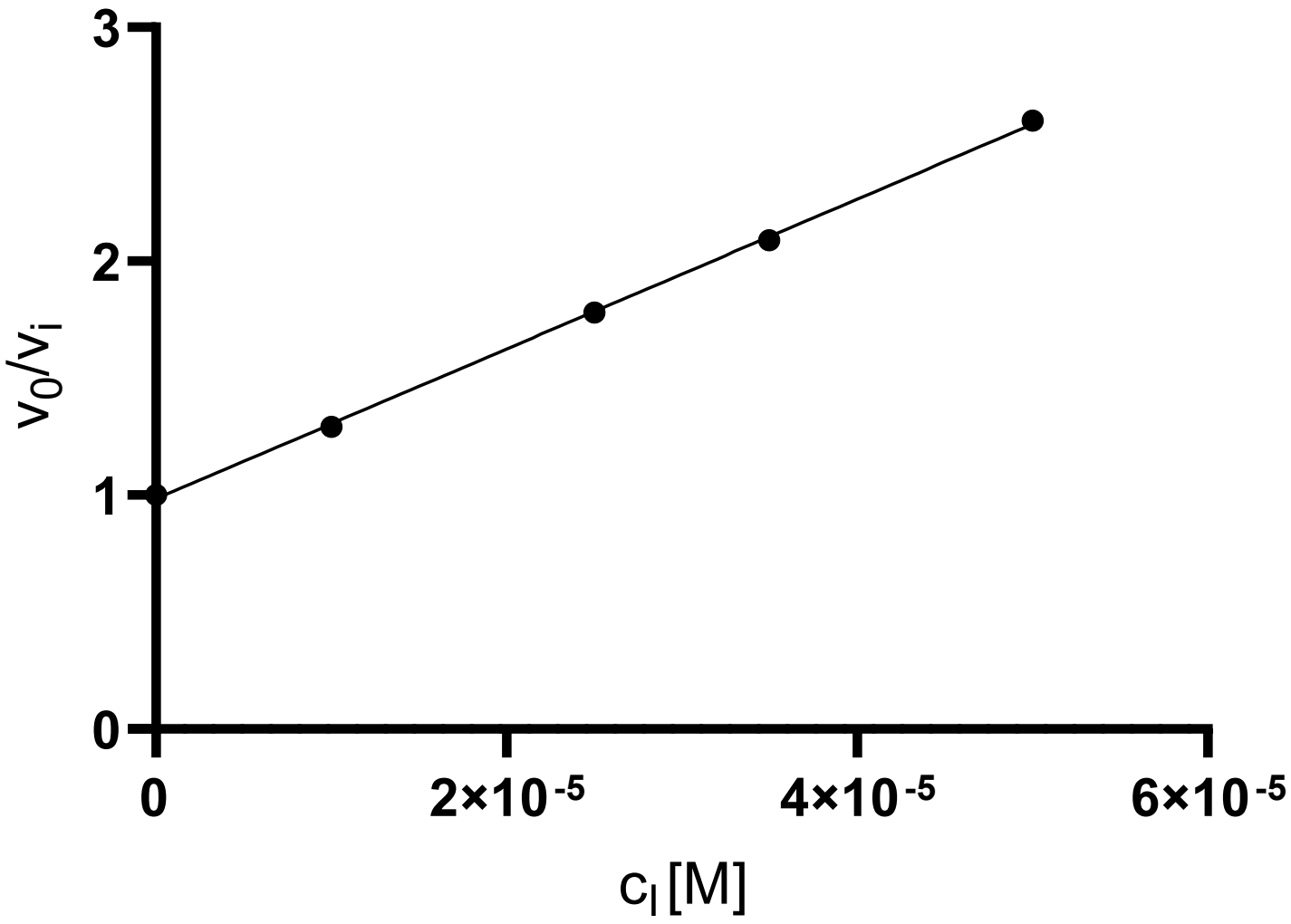

Figure **S33**. The dependence v_0/v_i vs. concentration of inhibitor **1i** inhibiting eeAChE.

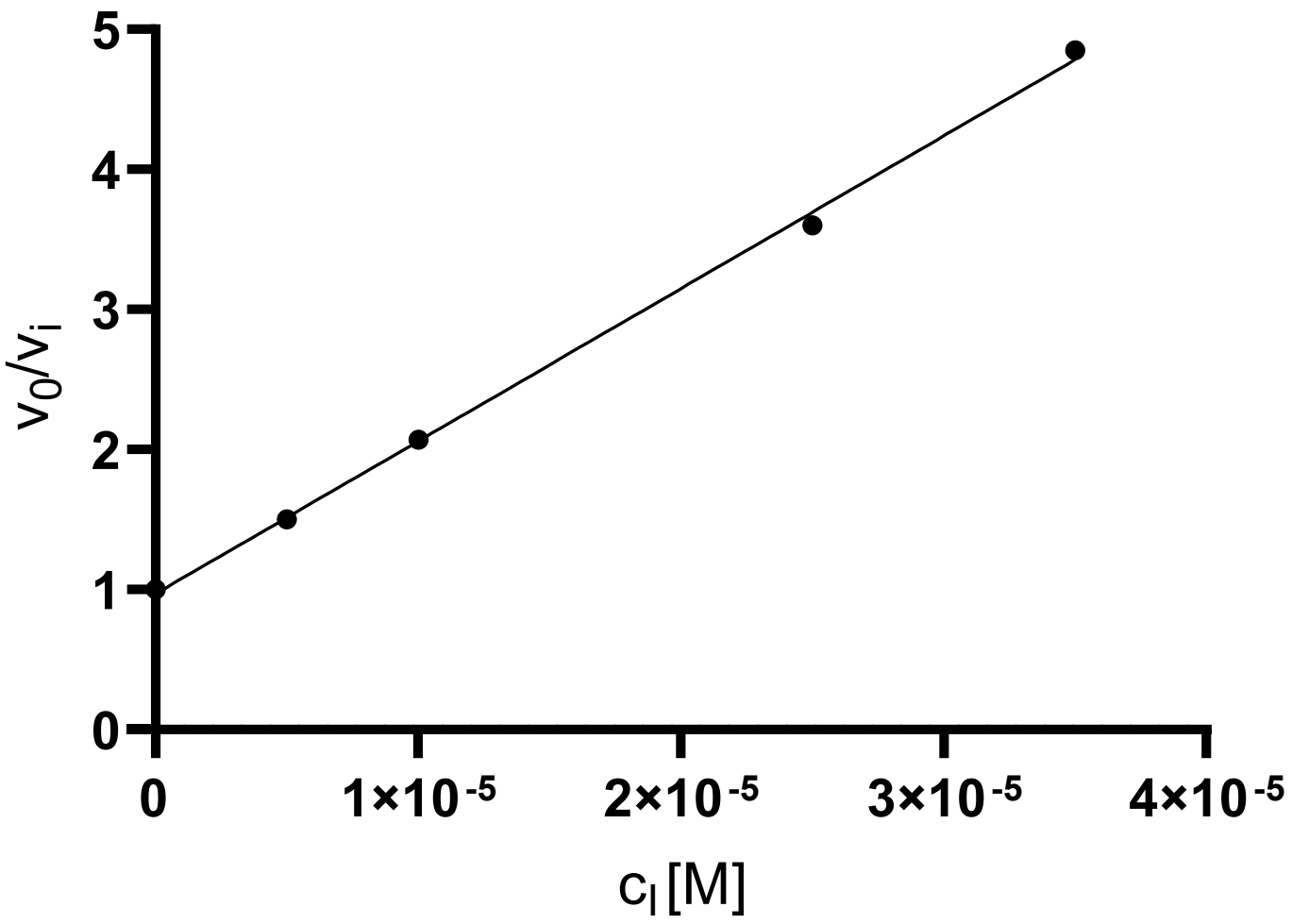

Figure **S34**. The dependence v_0/v_i vs. concentration of inhibitor **1i** inhibiting eqBChE.

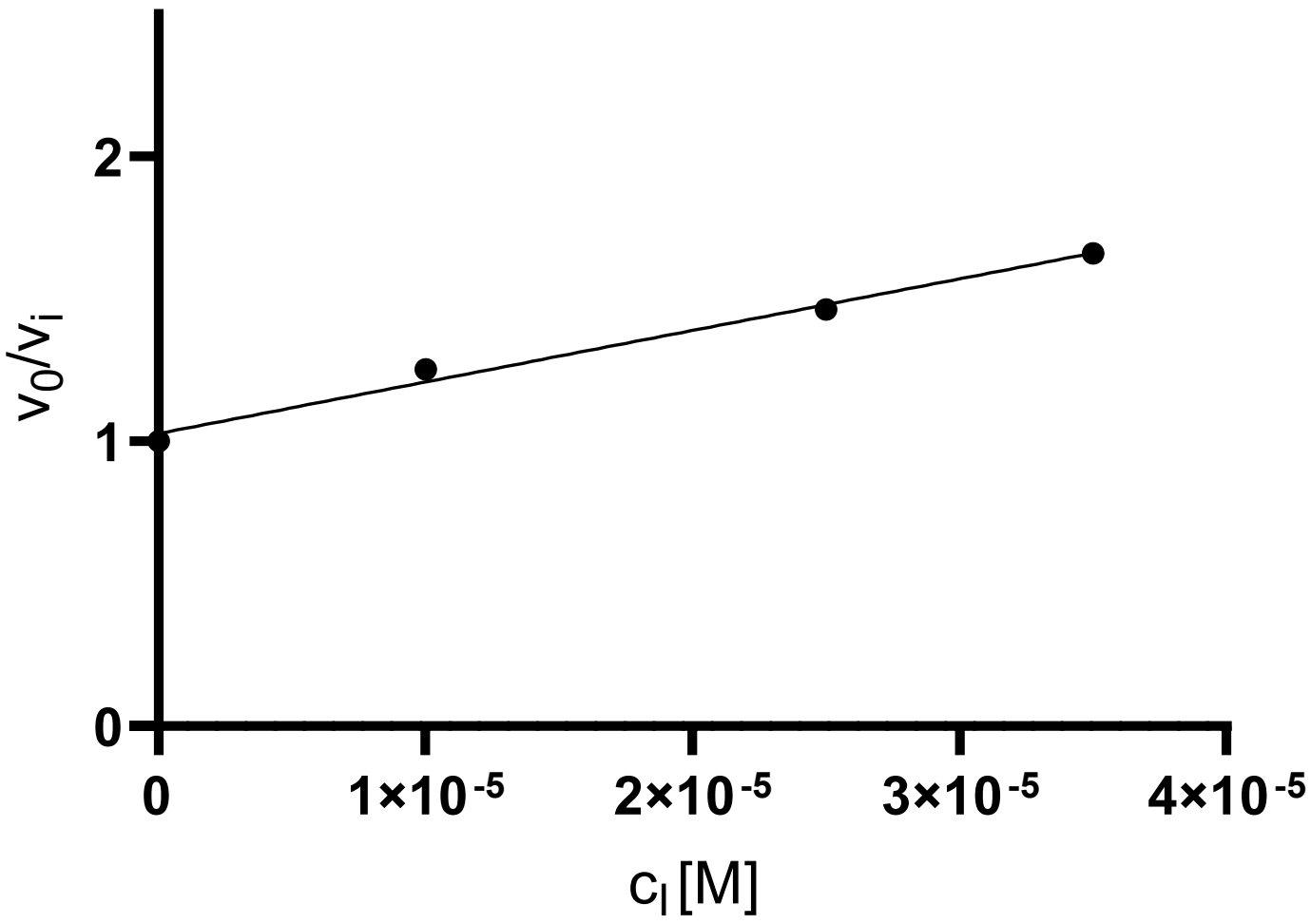

Figure **S35**. The dependence v_0/v_i vs. concentration of inhibitor **2i** inhibiting eeAChE.

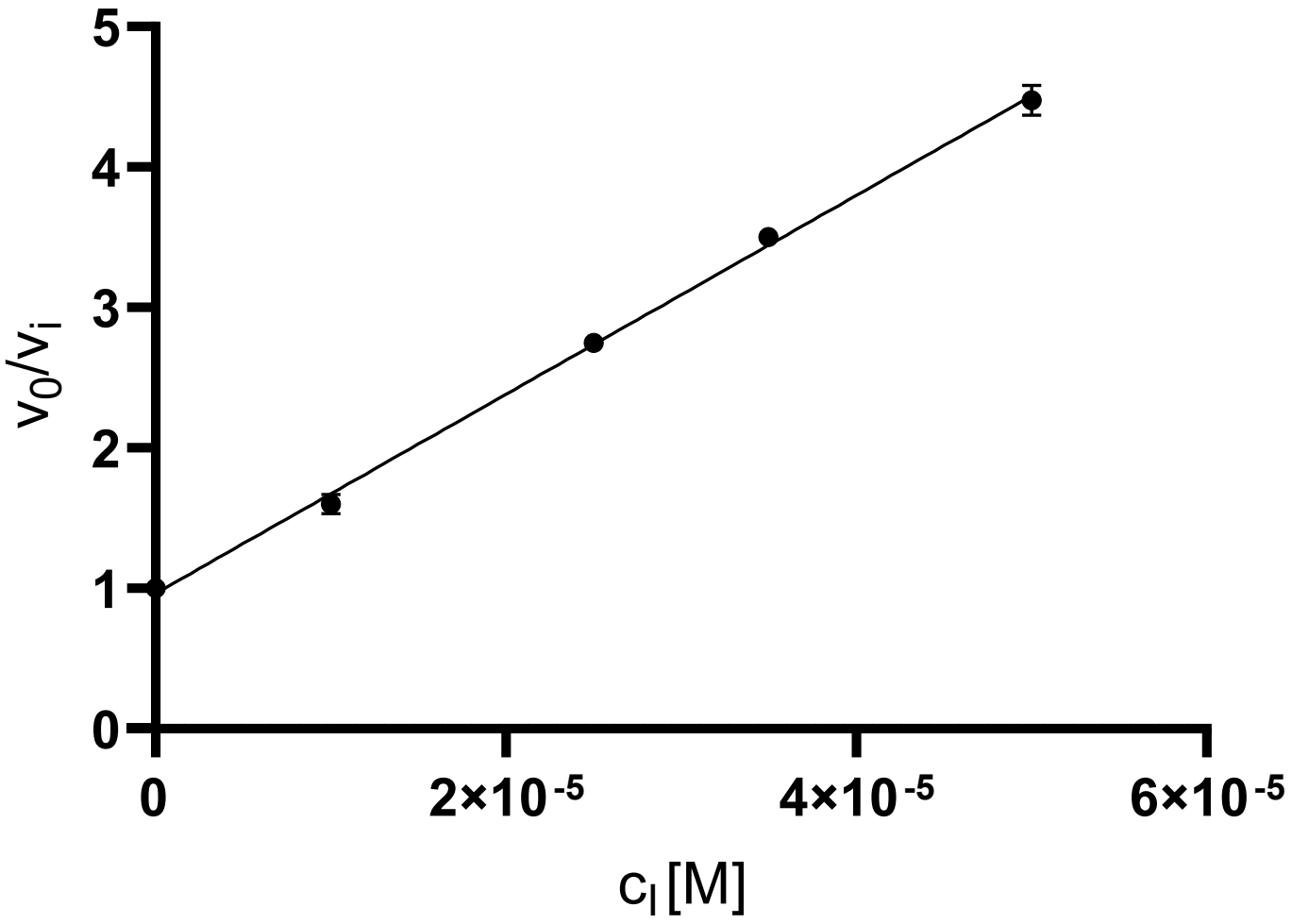

Figure **S36**. The dependence v_0/v_i vs. concentration of inhibitor **2i** inhibiting eqBChE.

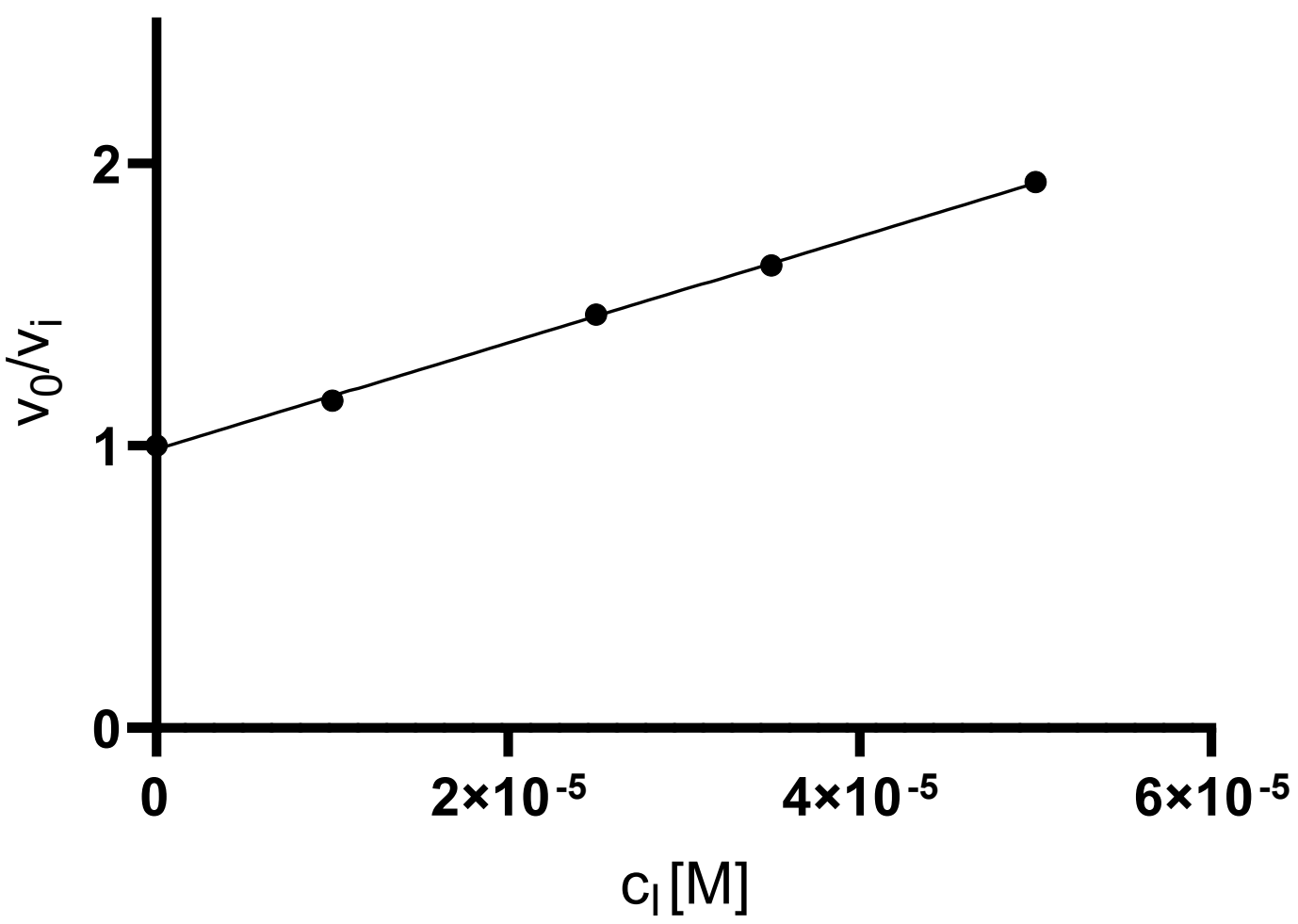

Figure **S37**. The dependence v_0/v_i vs. concentration of inhibitor **1j** inhibiting eeAChE.

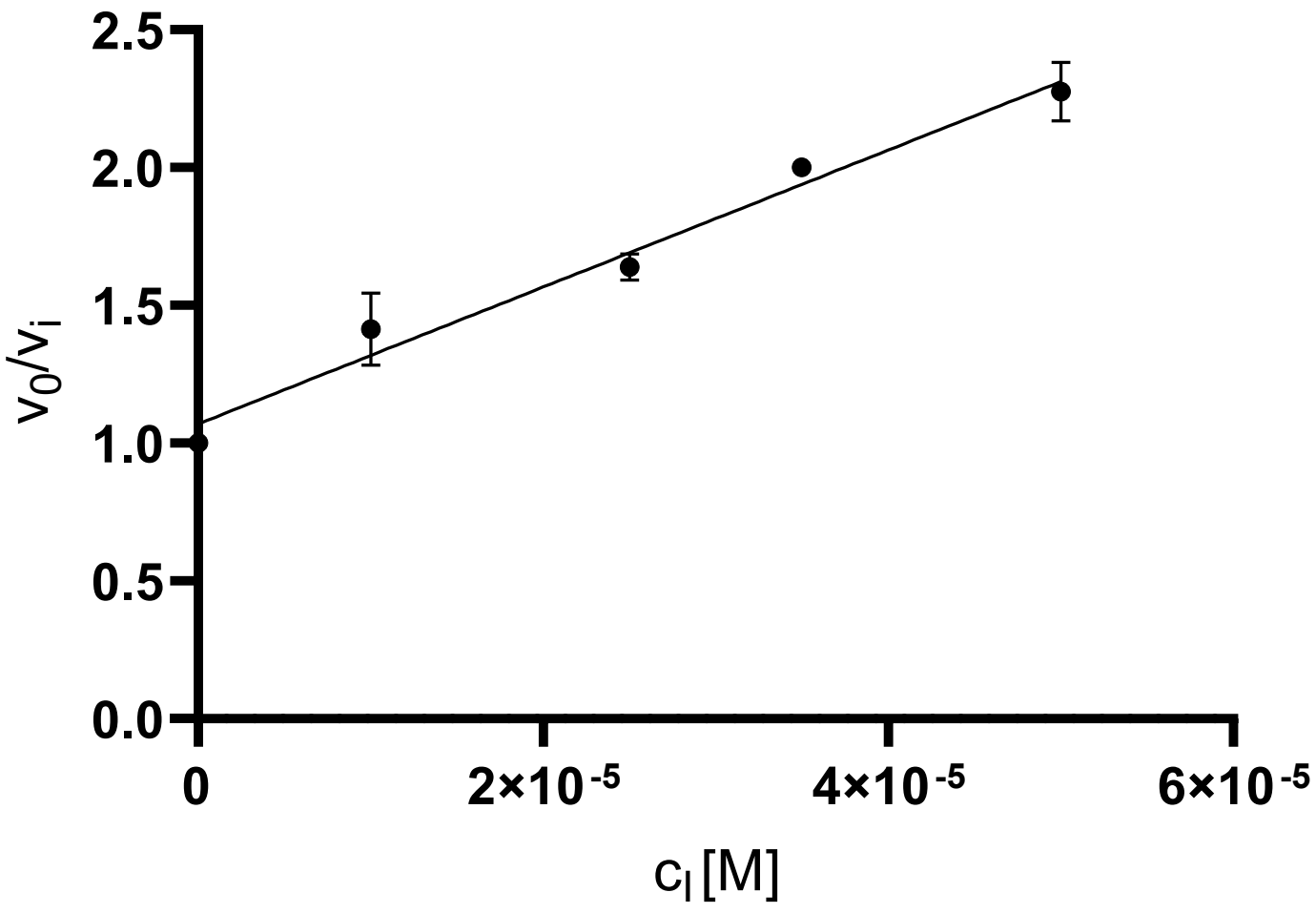

Figure **S38**. The dependence v_0/v_i vs. concentration of inhibitor **1j** inhibiting eqBChE.

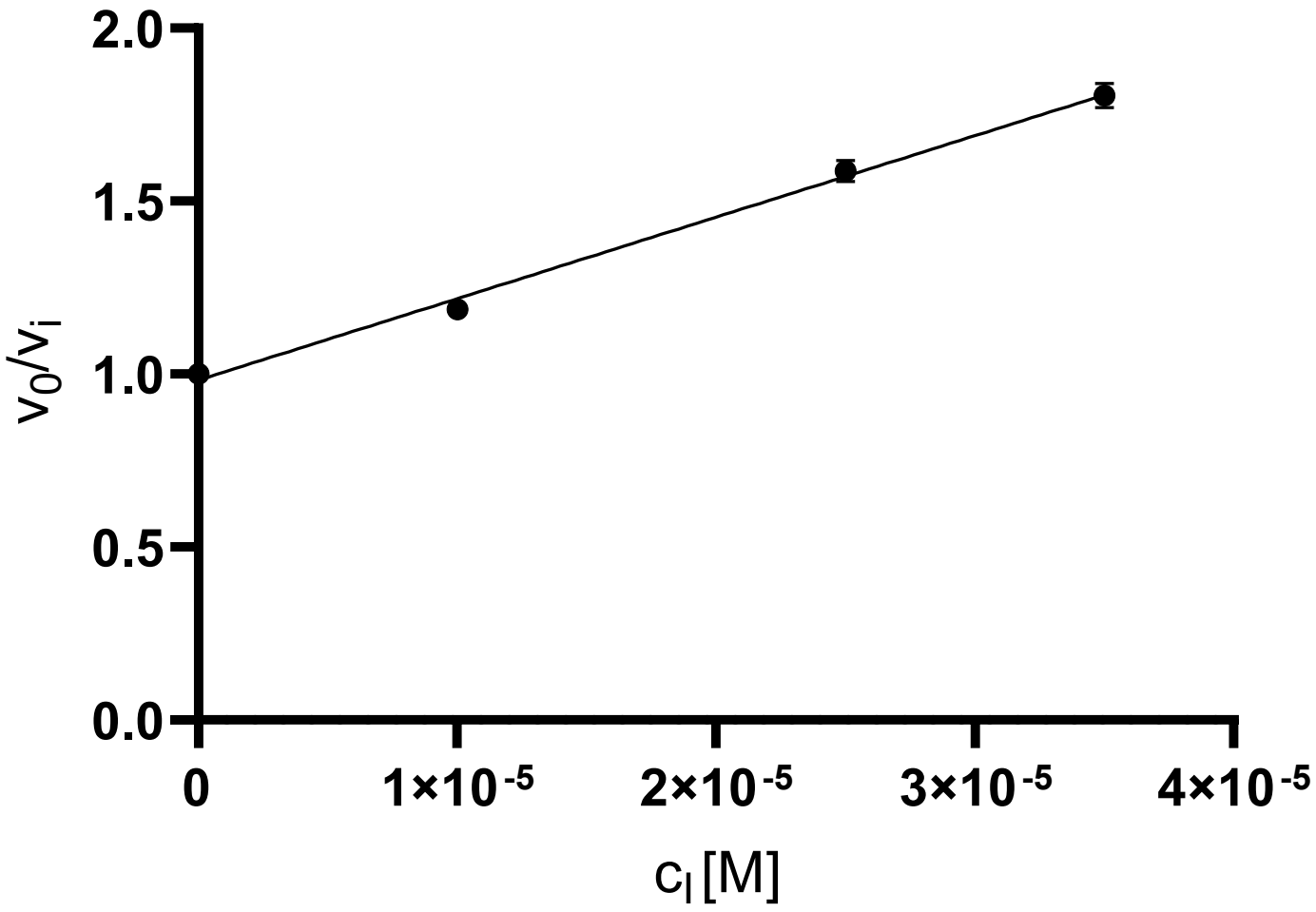

Figure **S39**. The dependence v_0/v_i vs. concentration of inhibitor **2j** inhibiting eeAChE.

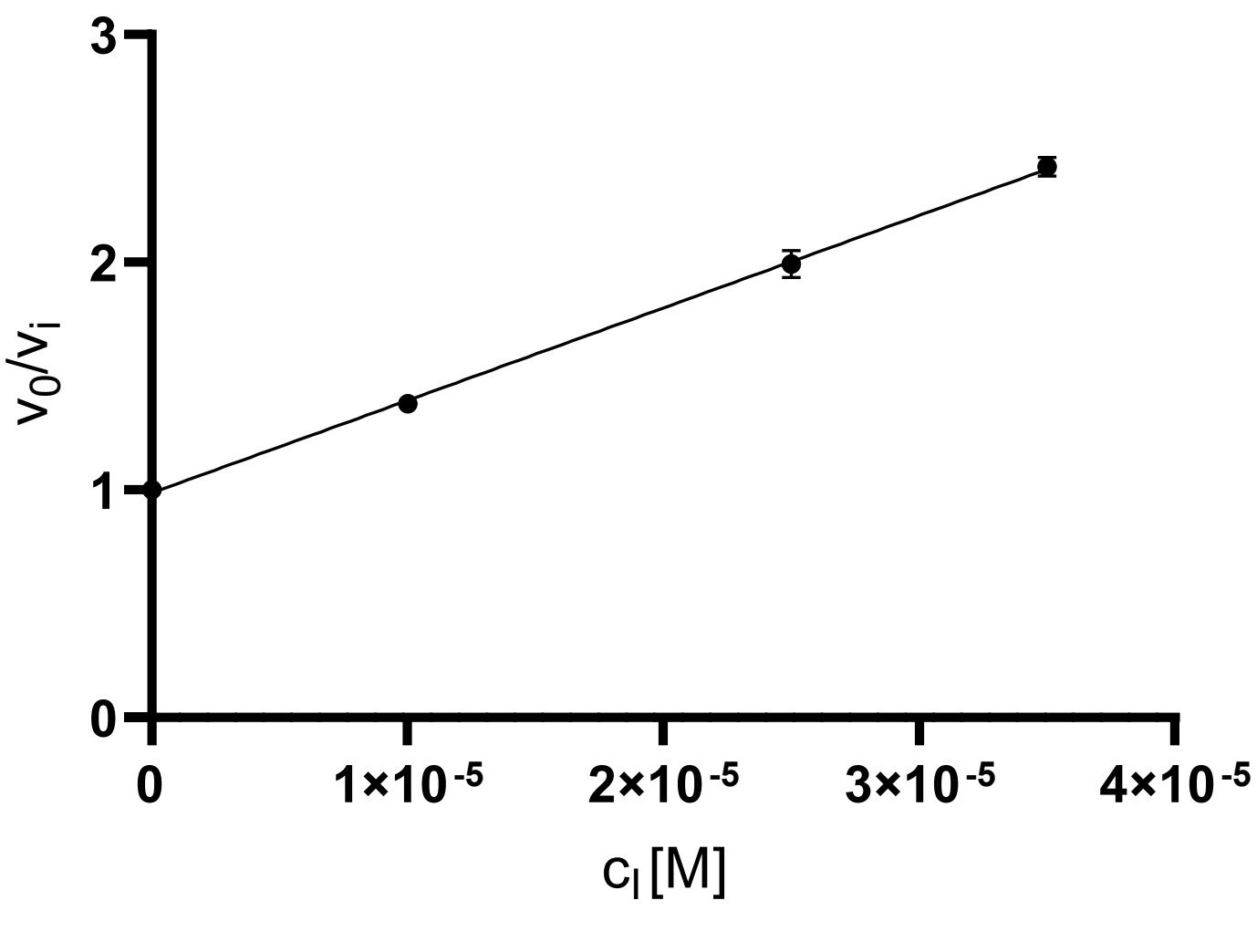

Figure **S40**. The dependence v_0/v_i vs. concentration of inhibitor **2j** inhibiting eqBChE.

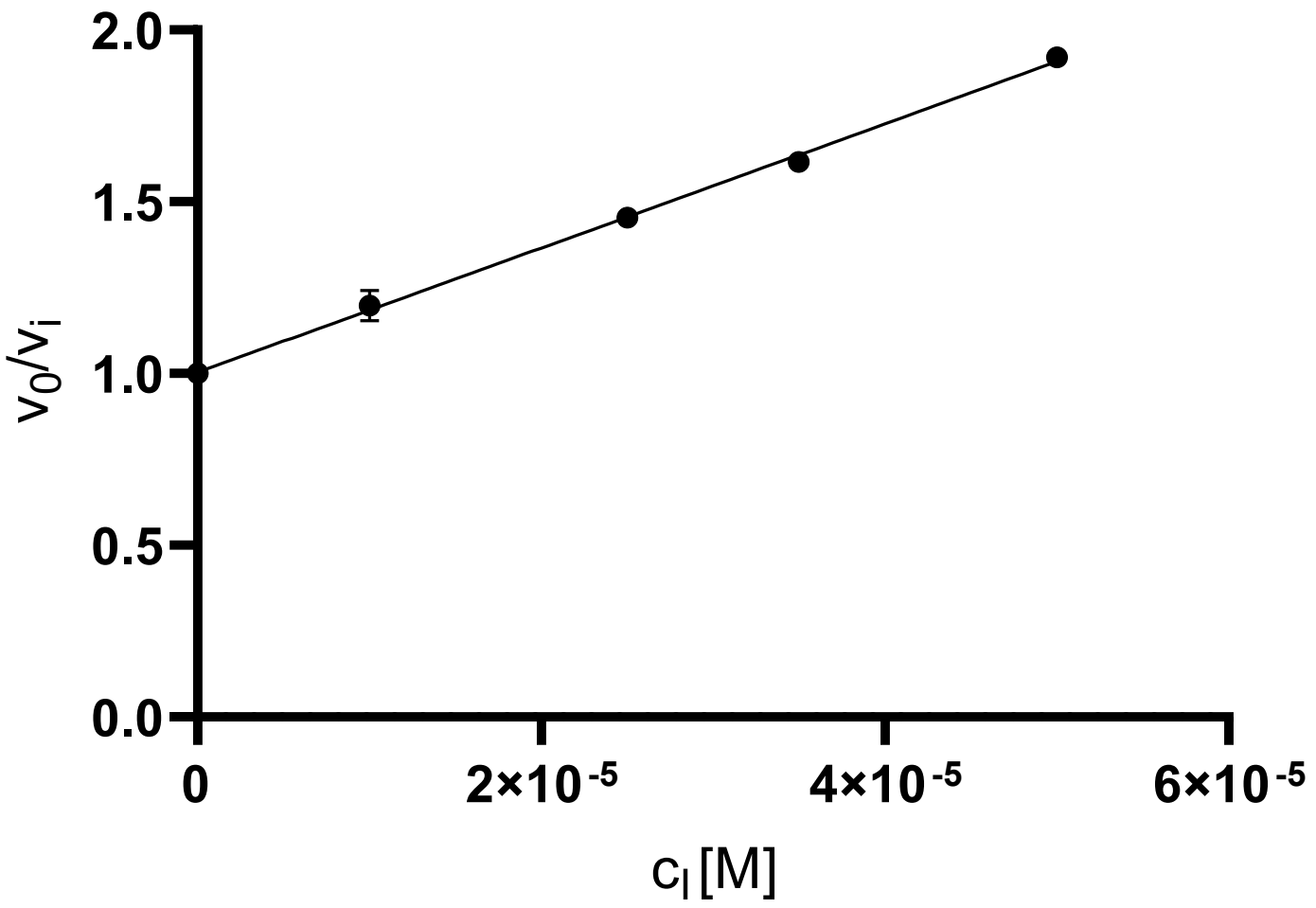

Figure **S41**. The dependence v_0/v_i vs. concentration of inhibitor **1k** inhibiting eeAChE.

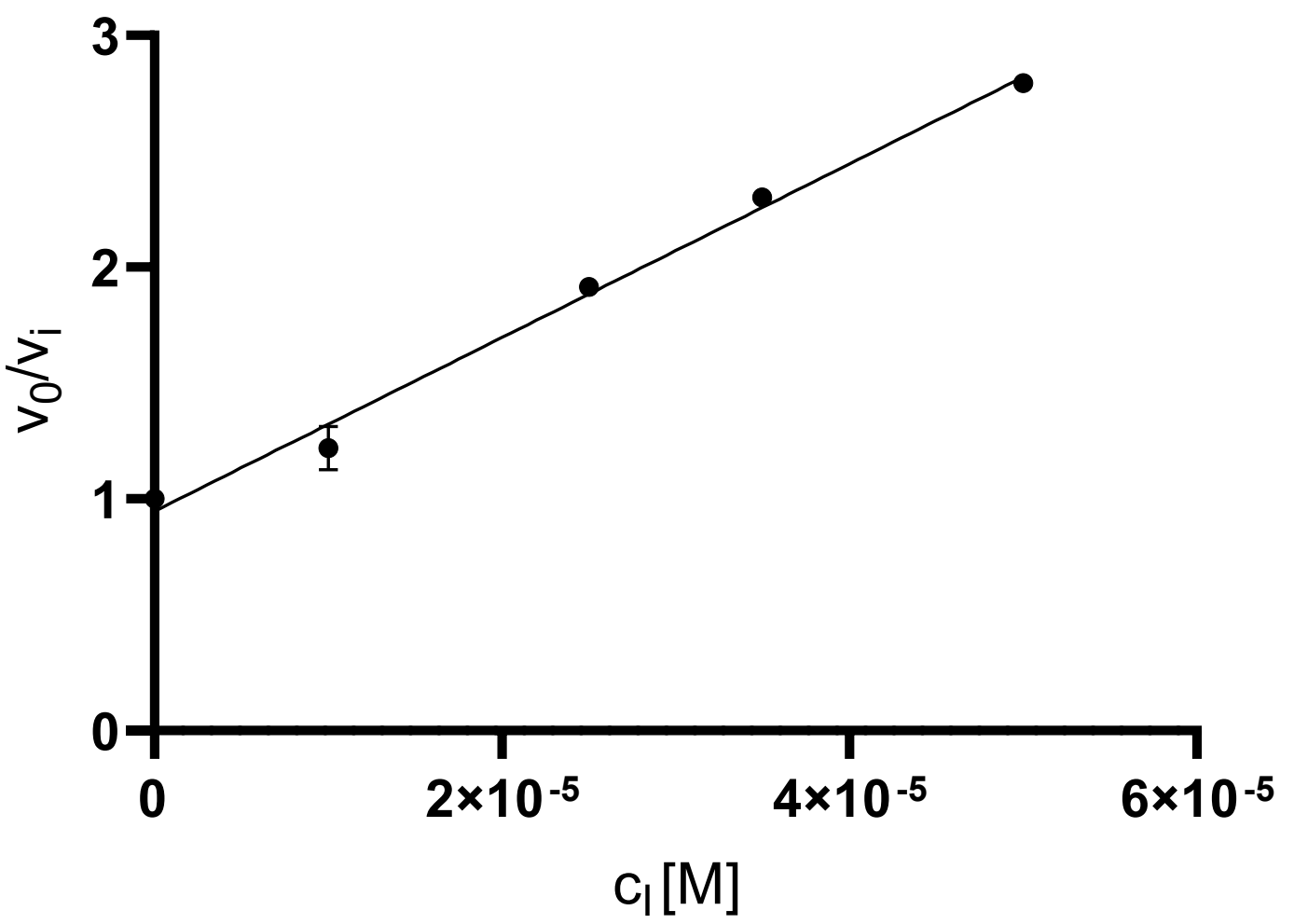

Figure **S42**. The dependence v_0/v_i vs. concentration of inhibitor **1k** inhibiting eqBChE.

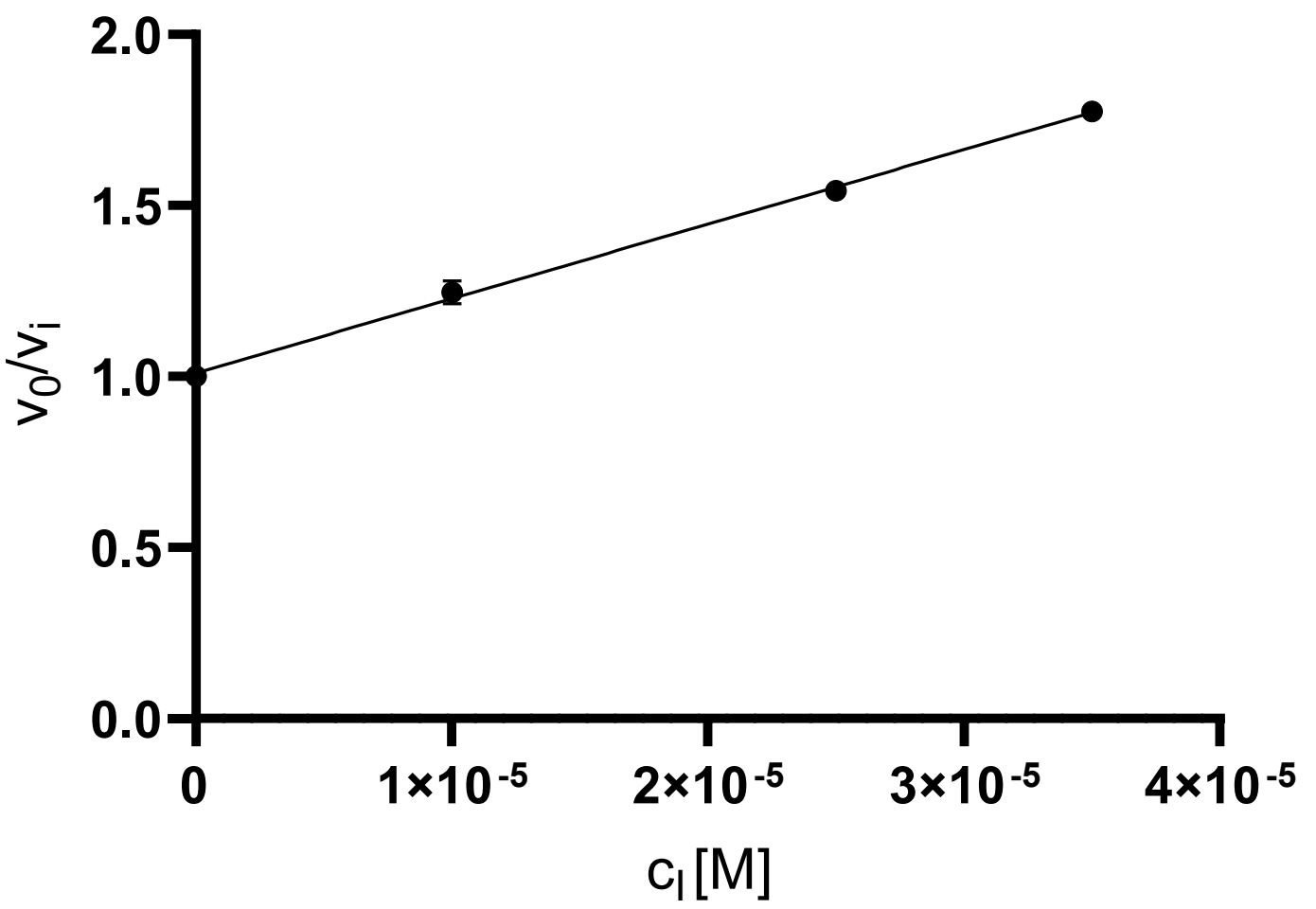

Figure **S43**. The dependence v_0/v_i vs. concentration of inhibitor **2k** inhibiting eeAChE.

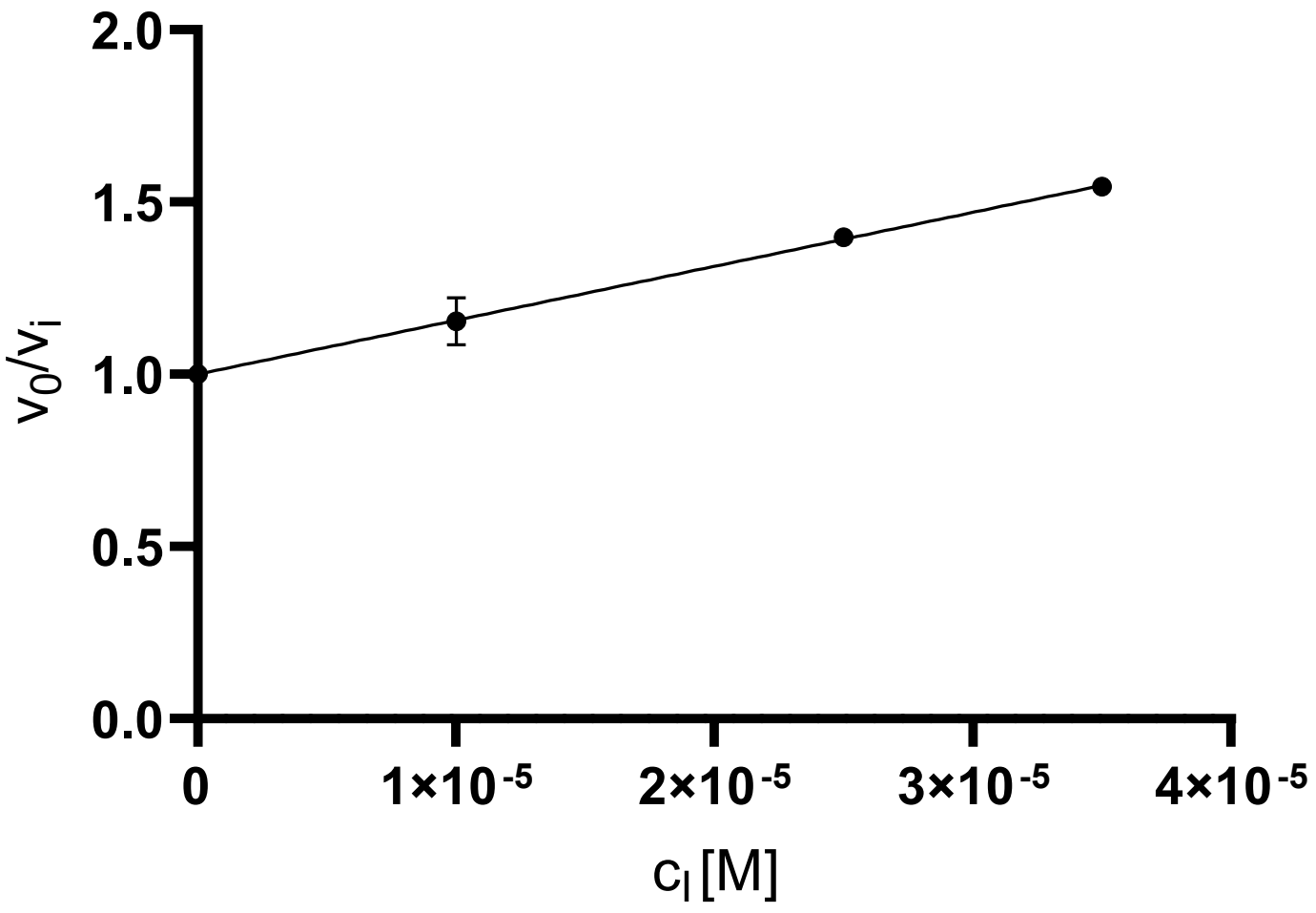

Figure **S44**. The dependence v_0/v_i vs. concentration of inhibitor **2k** inhibiting eqBChE.

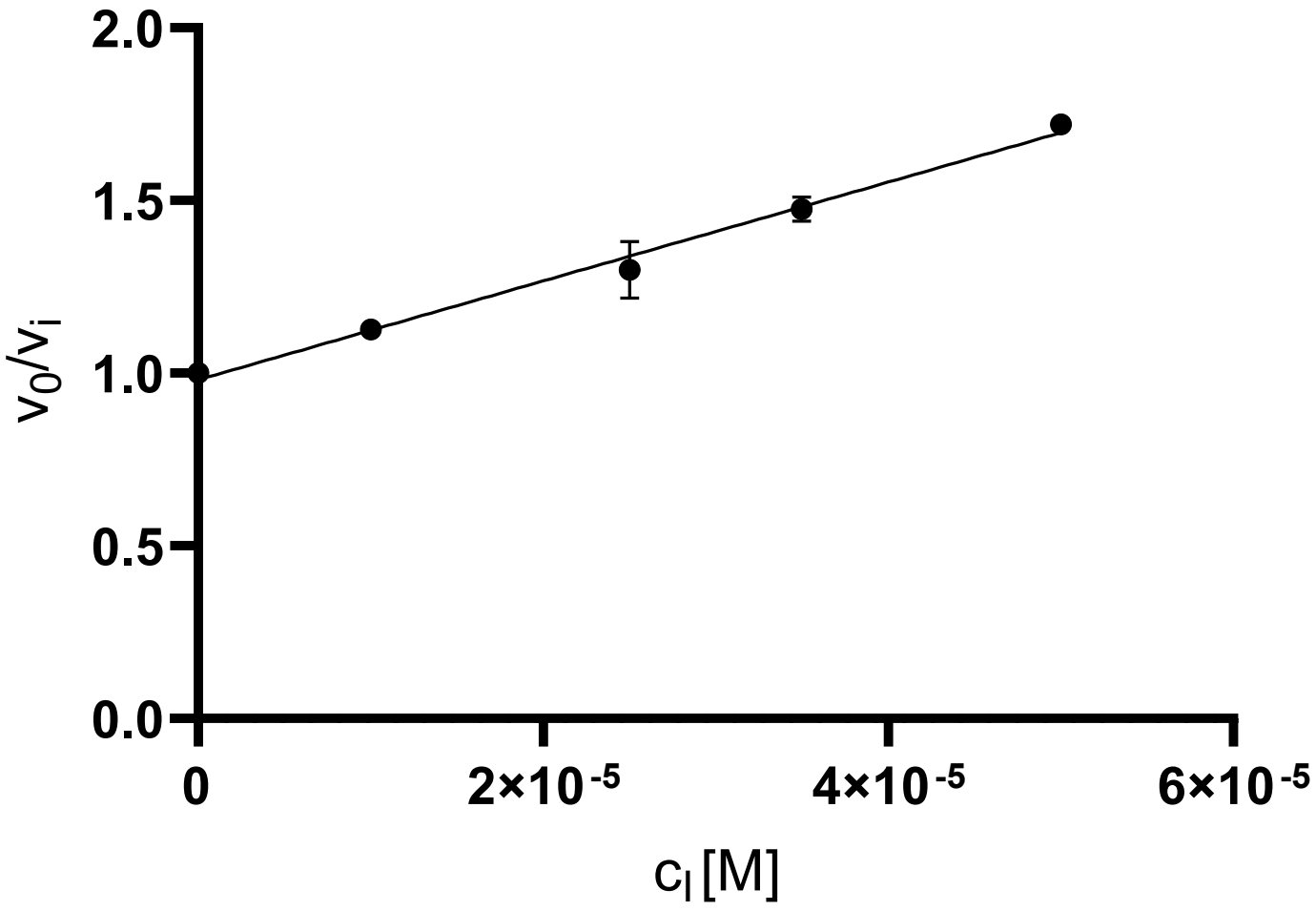

Figure **S45**. The dependence v_0/v_i vs. concentration of inhibitor **1l** inhibiting eeAChE.

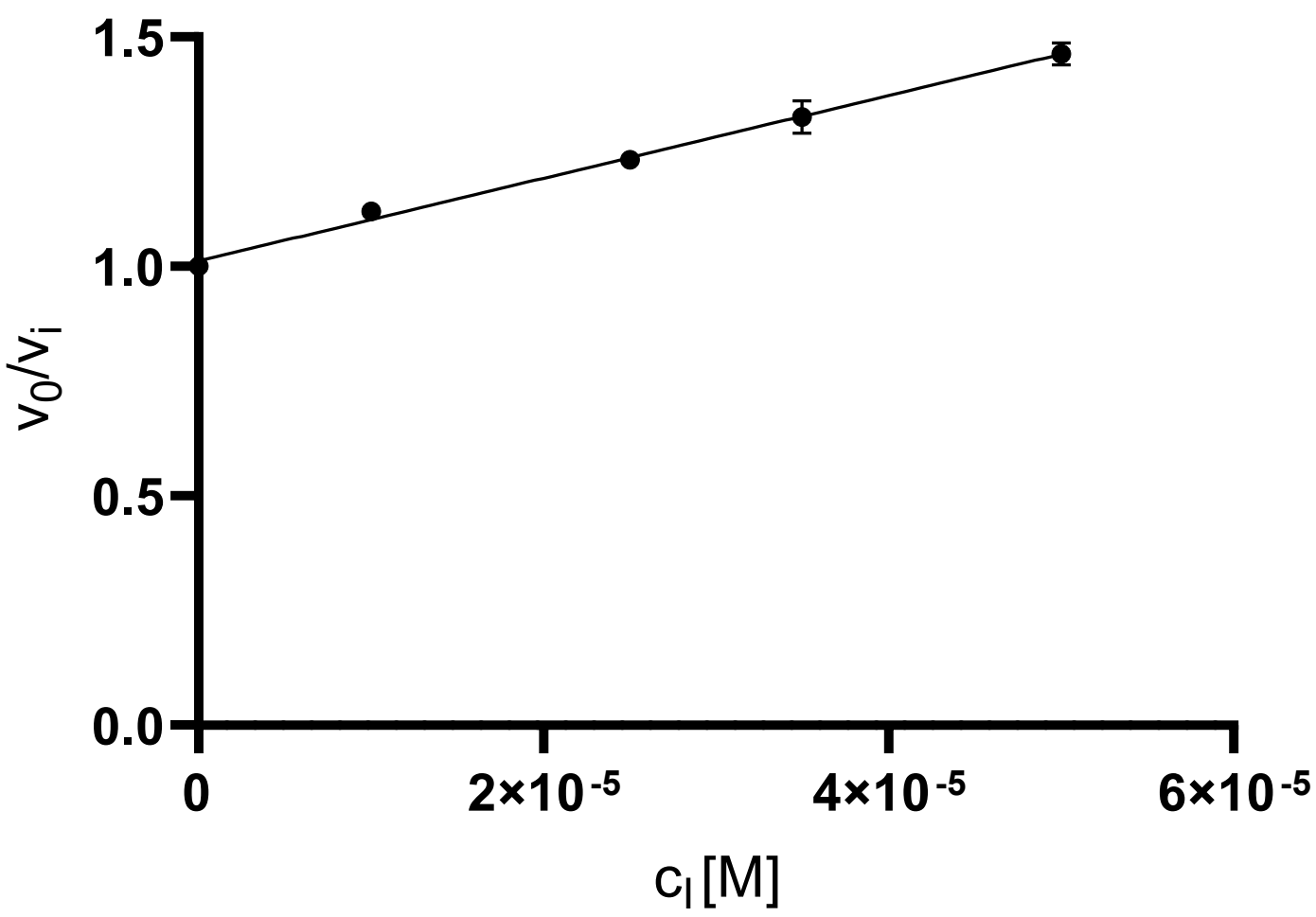

Figure **S46**. The dependence v_0/v_i vs. concentration of inhibitor **1l** inhibiting eqBChE.

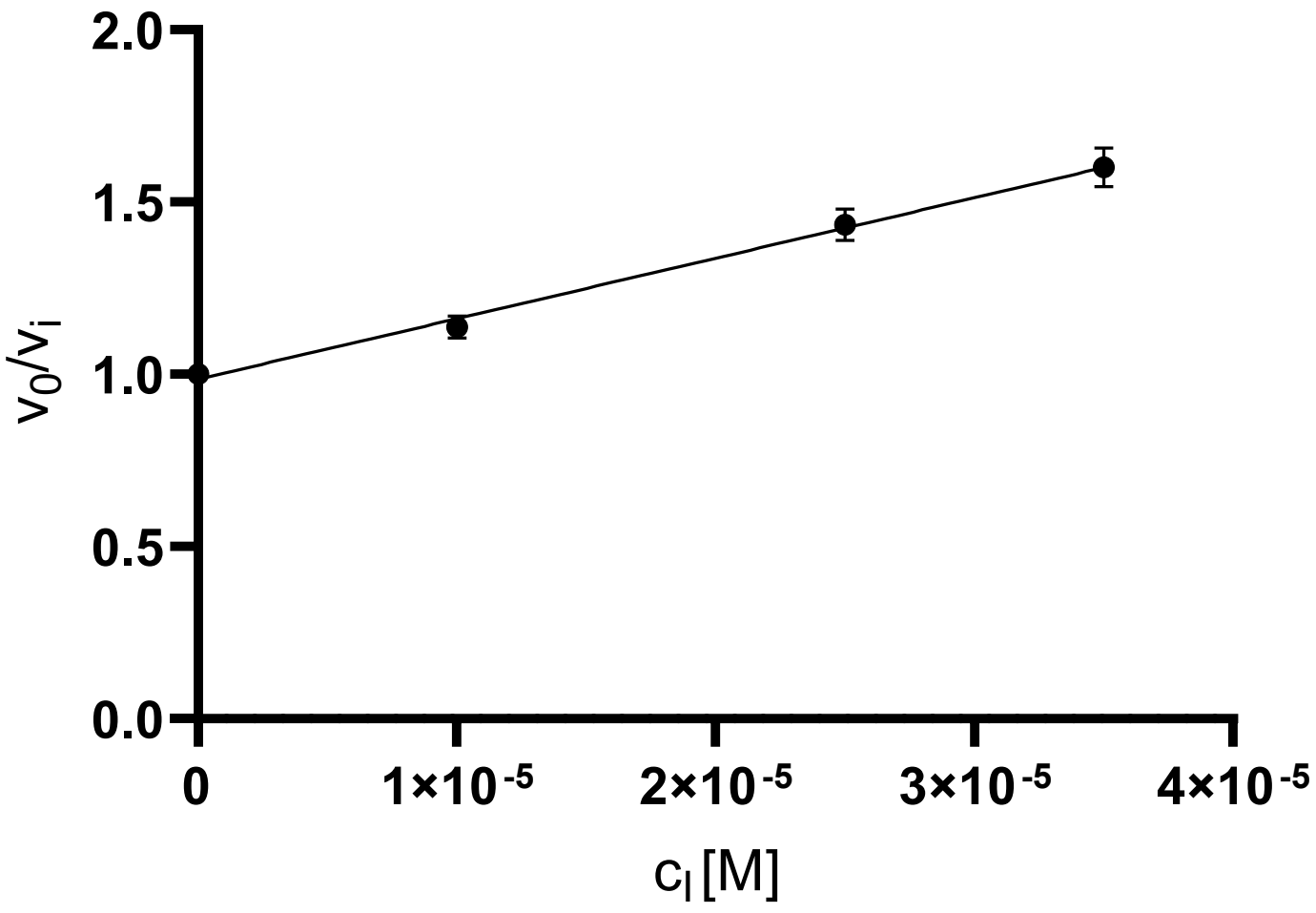

Figure **S47**. The dependence v_0/v_i vs. concentration of inhibitor **2l** inhibiting eeAChE.

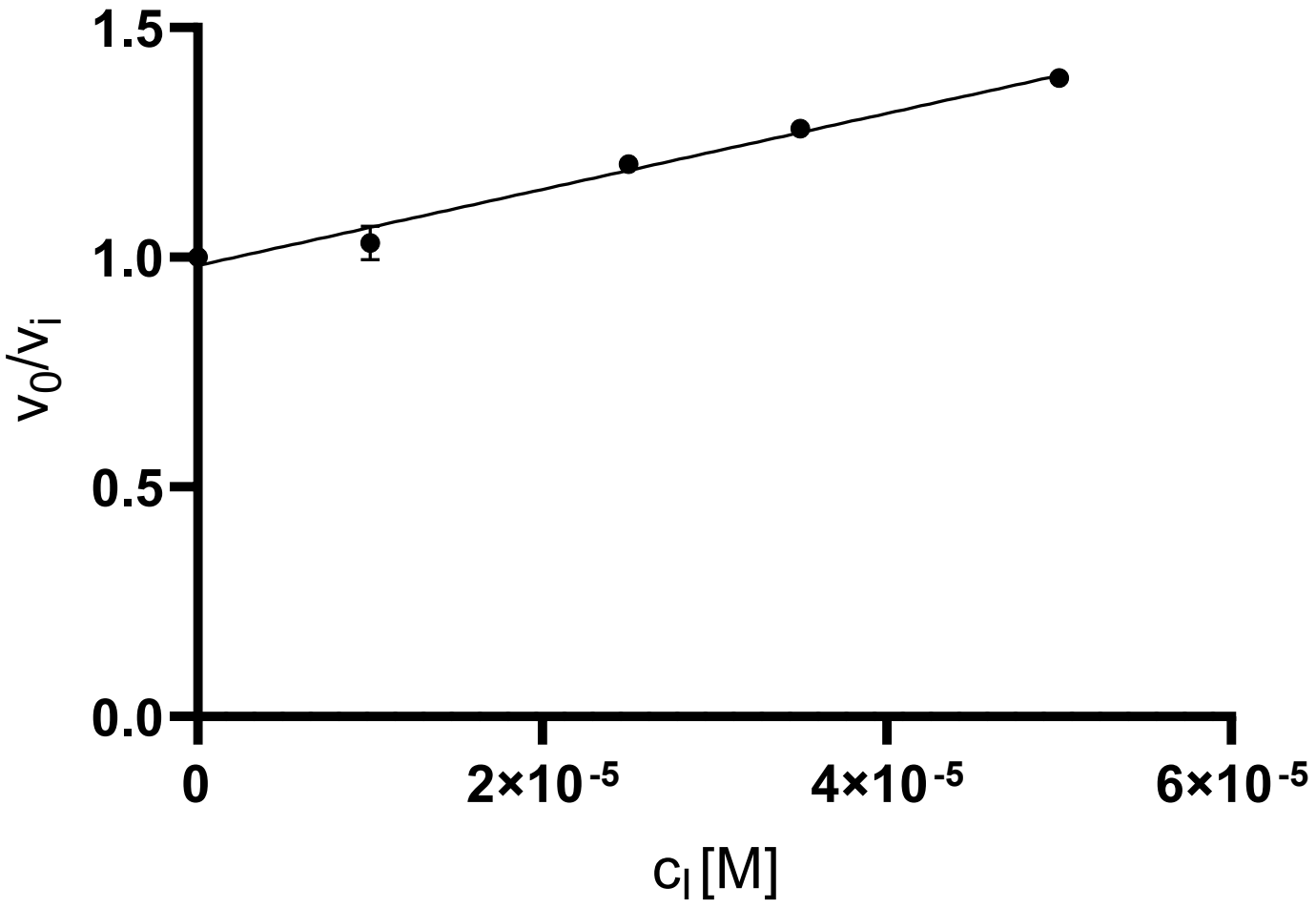

Figure **S48**. The dependence v_0/v_i vs. concentration of inhibitor **2l** inhibiting eqBChE.

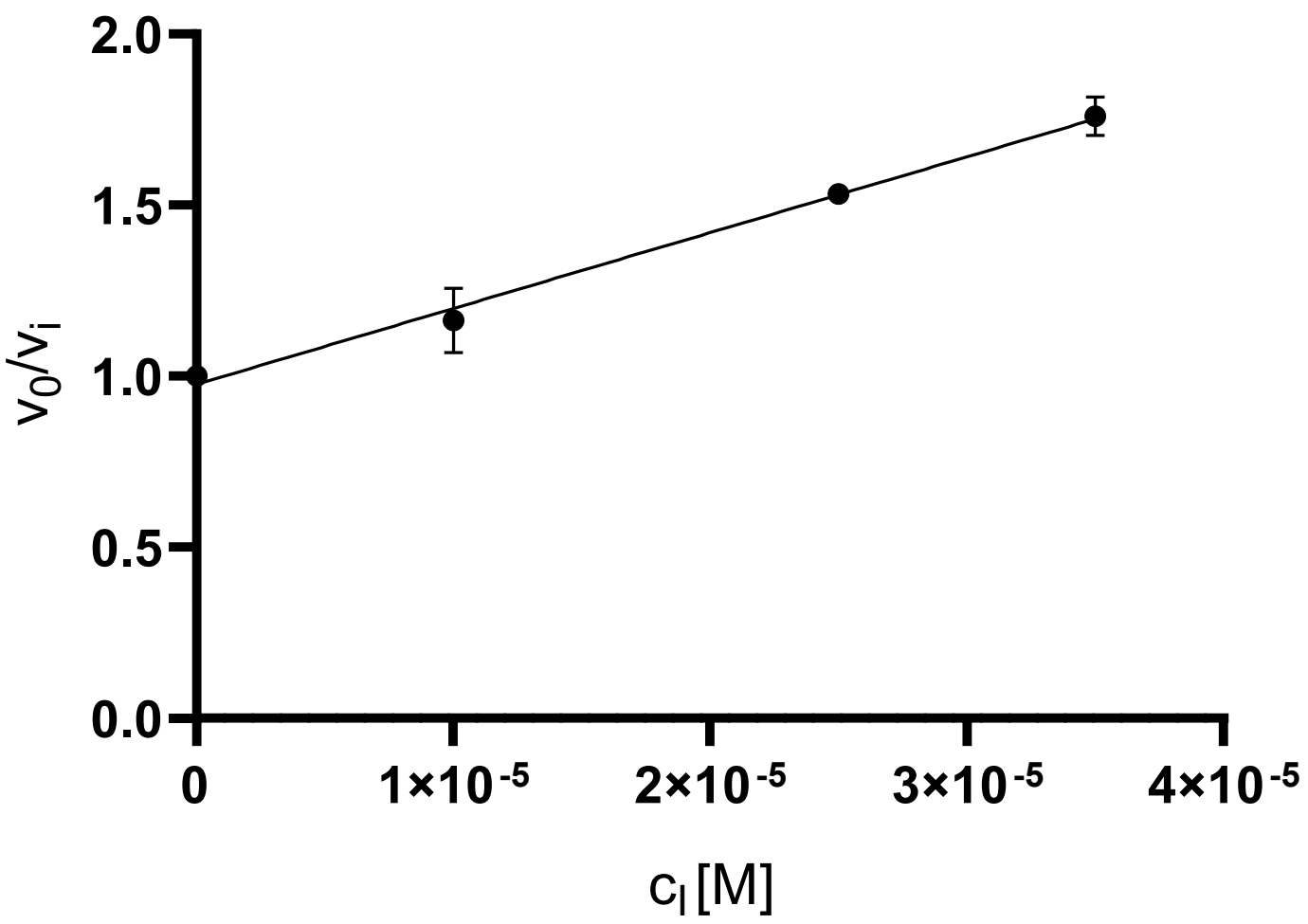

Figure **S49**. The dependence v_0/v_i vs. concentration of inhibitor **1m** inhibiting eeAChE.

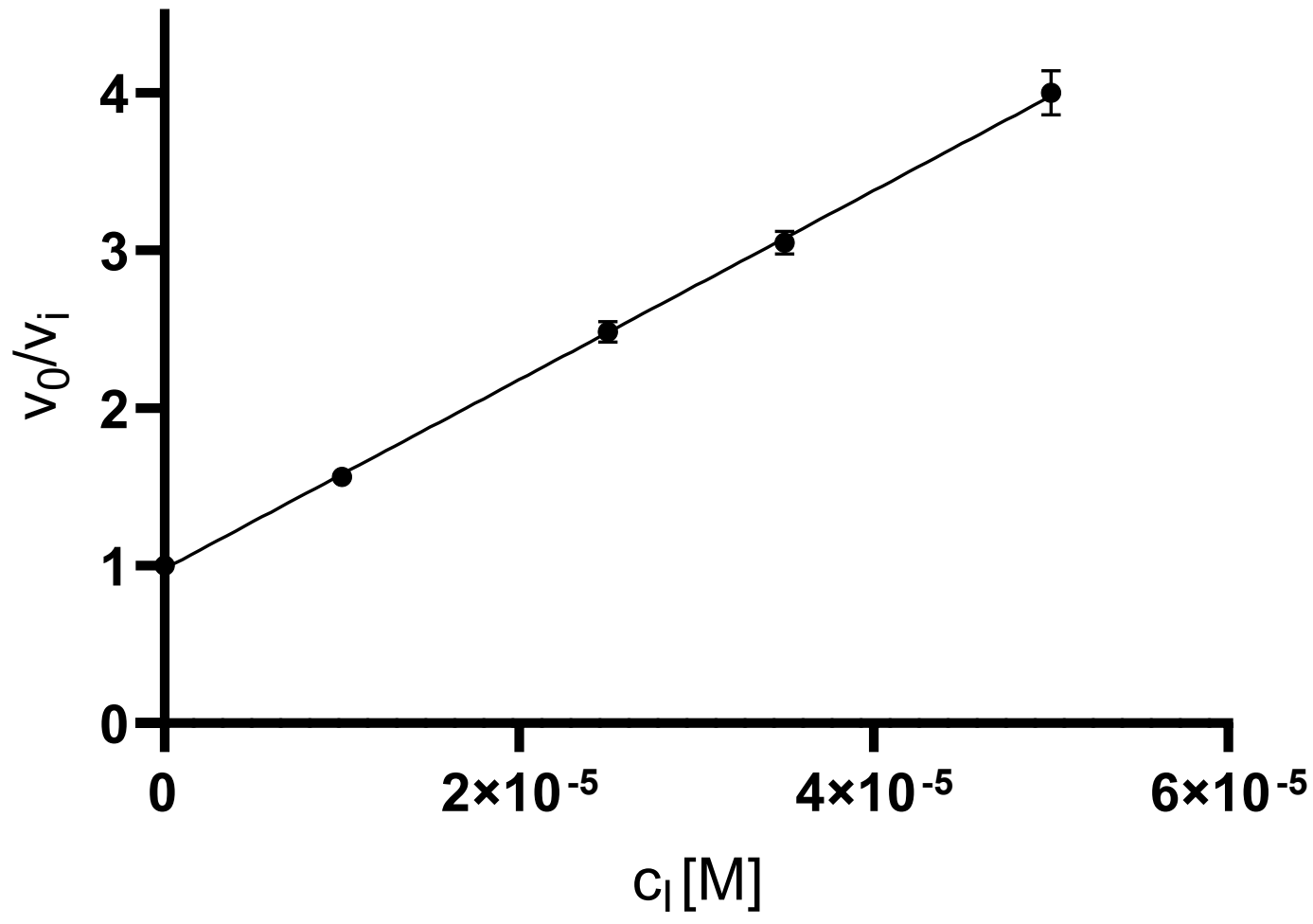

Figure **S50**. The dependence v_0/v_i vs. concentration of inhibitor **1m** inhibiting eqBChE.

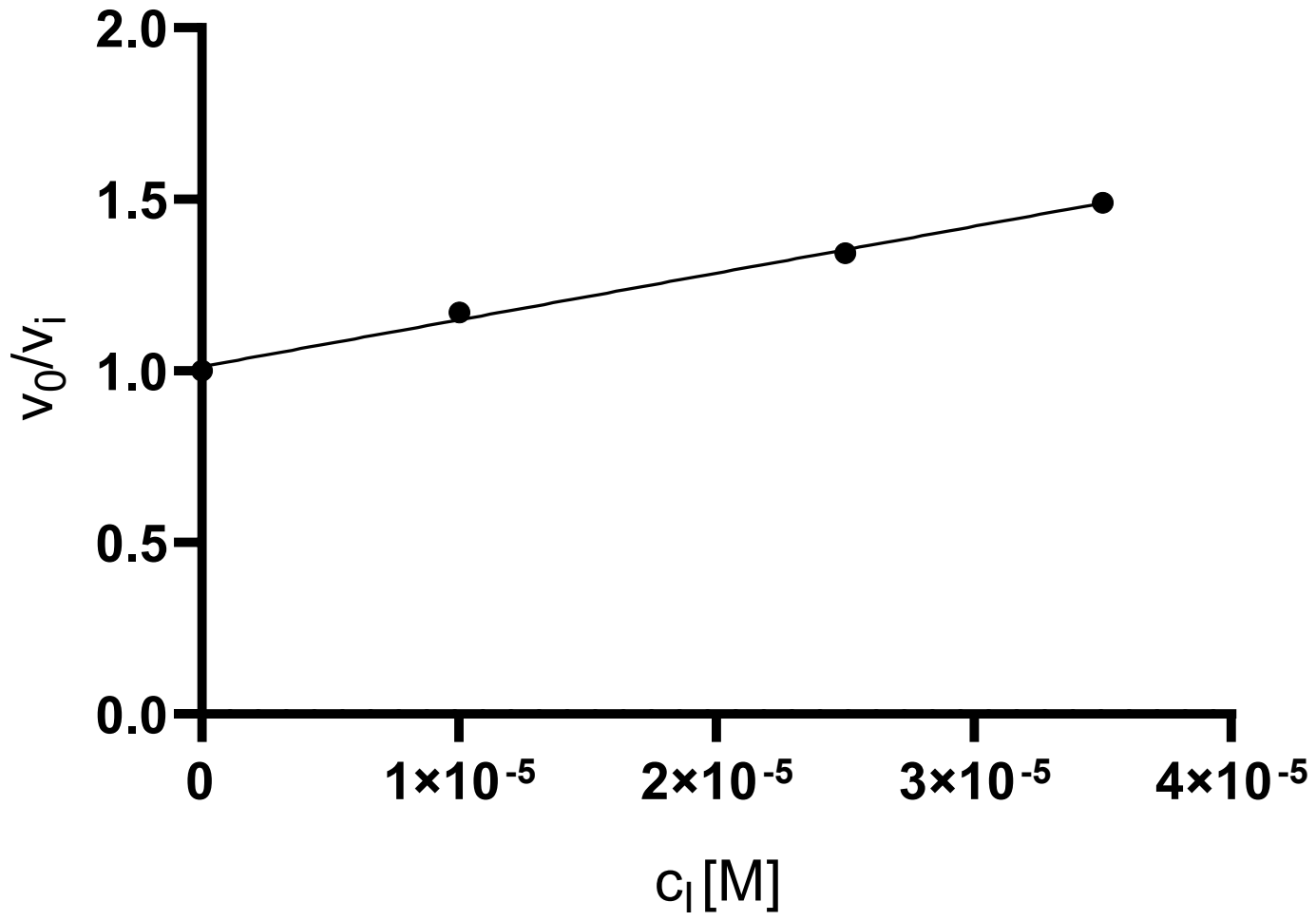

Figure **S51**. The dependence v_0/v_i vs. concentration of inhibitor **2m** inhibiting eeAChE.

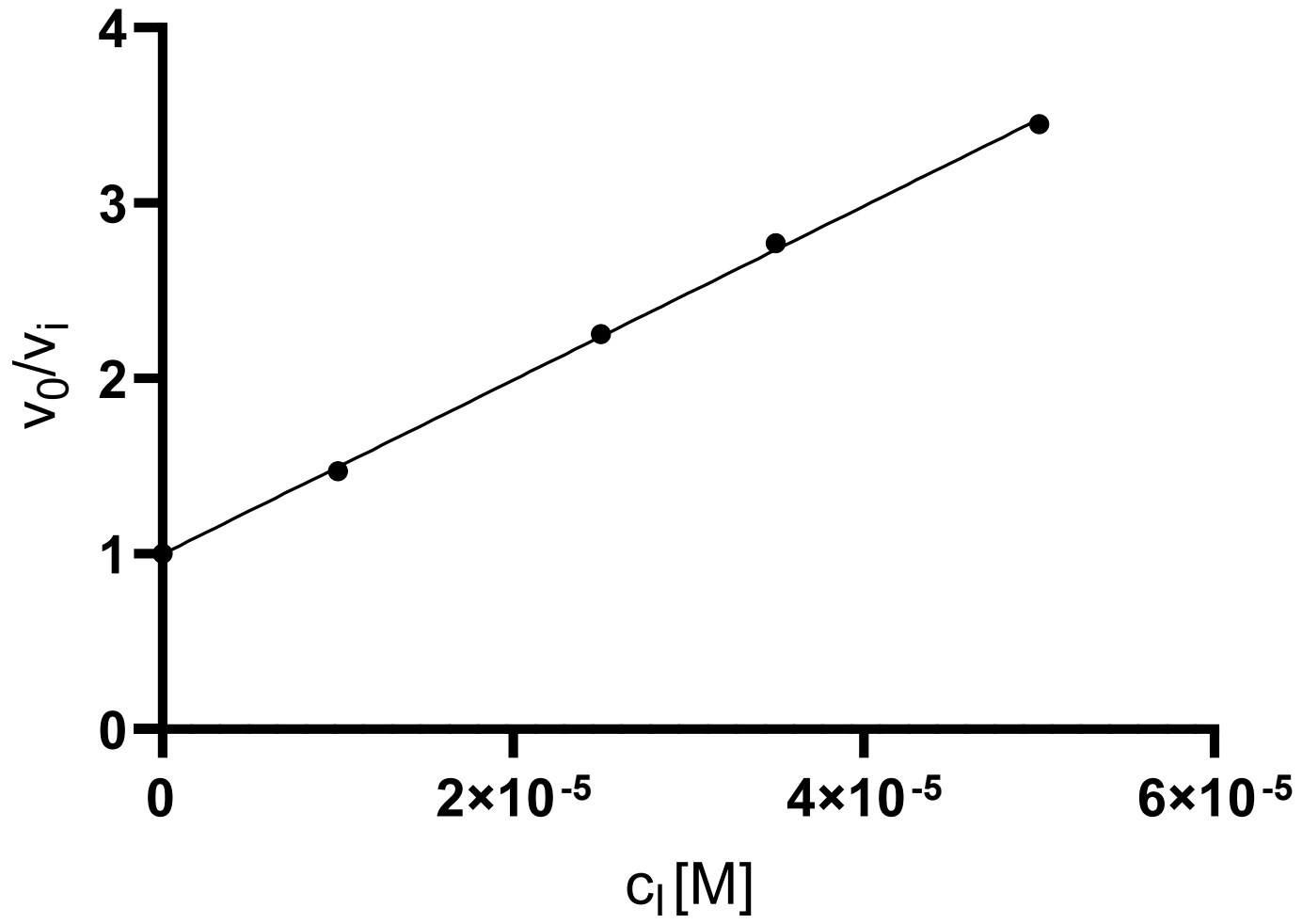

Figure **S52**. The dependence v_0/v_i vs. concentration of inhibitor **2m** inhibiting eqBChE.

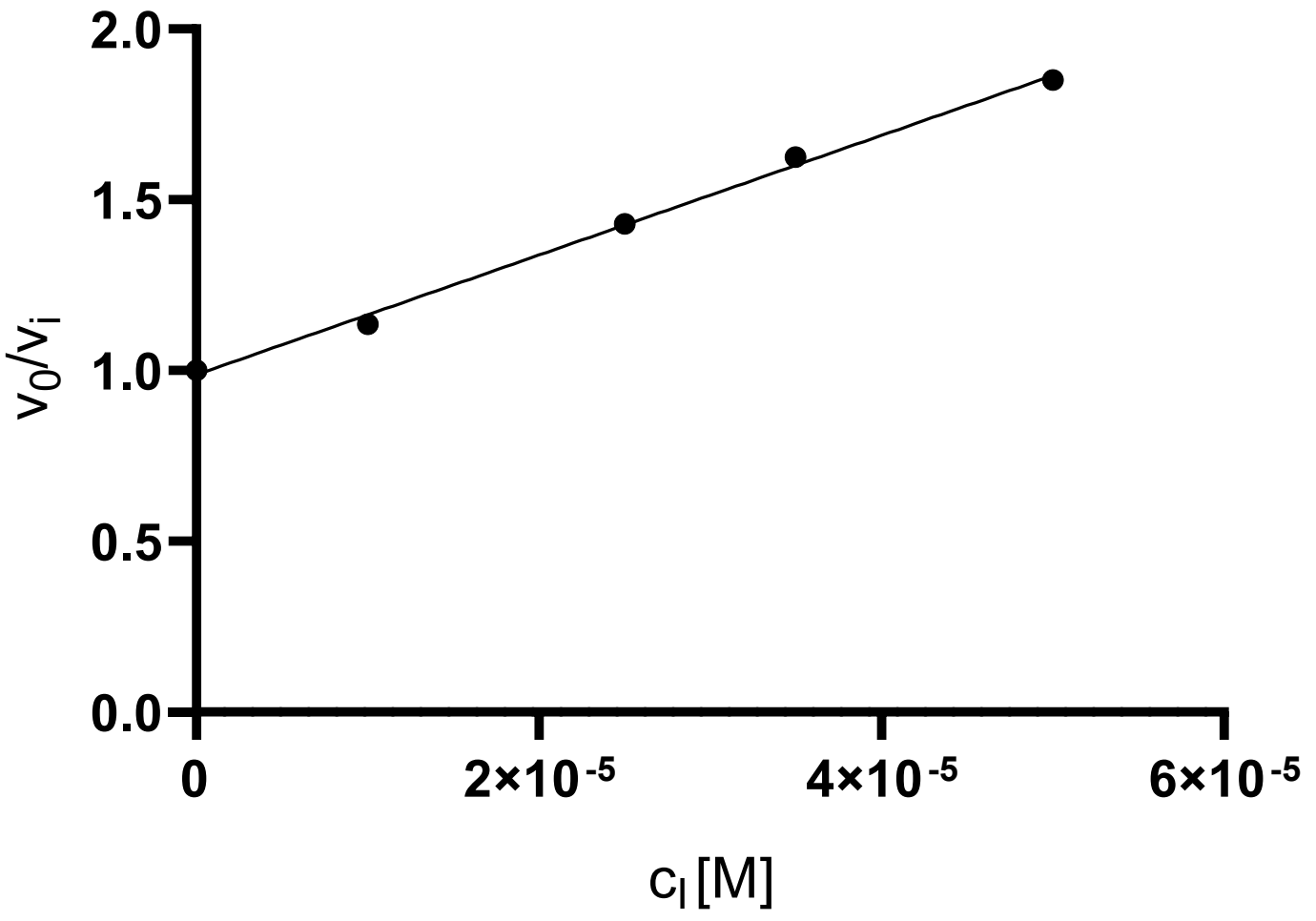

Figure **S53**. The dependence v_0/v_i vs. concentration of inhibitor **1n** inhibiting eeAChE.

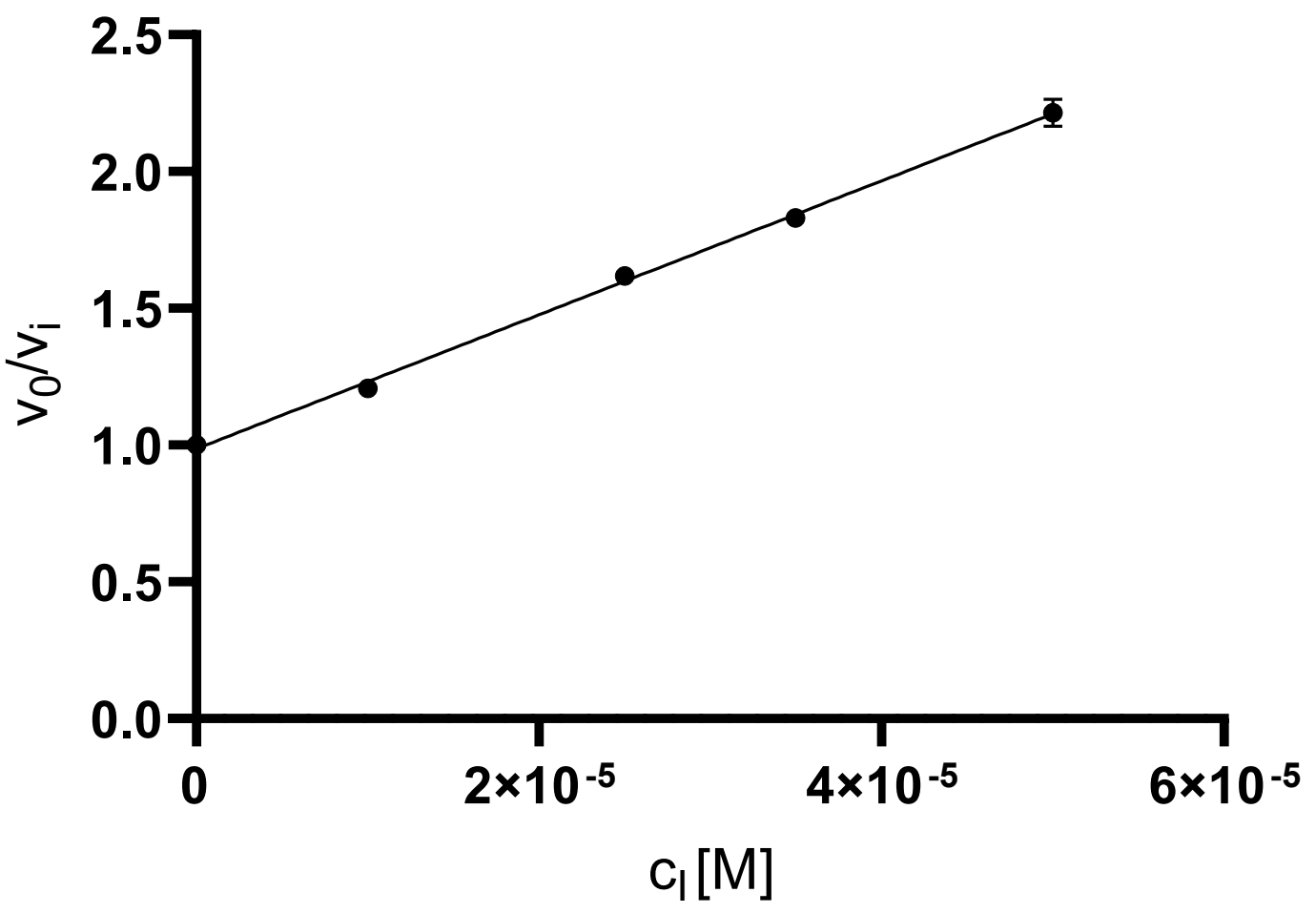

Figure **S54**. The dependence v_0/v_i vs. concentration of inhibitor **1n** inhibiting eqBChE.

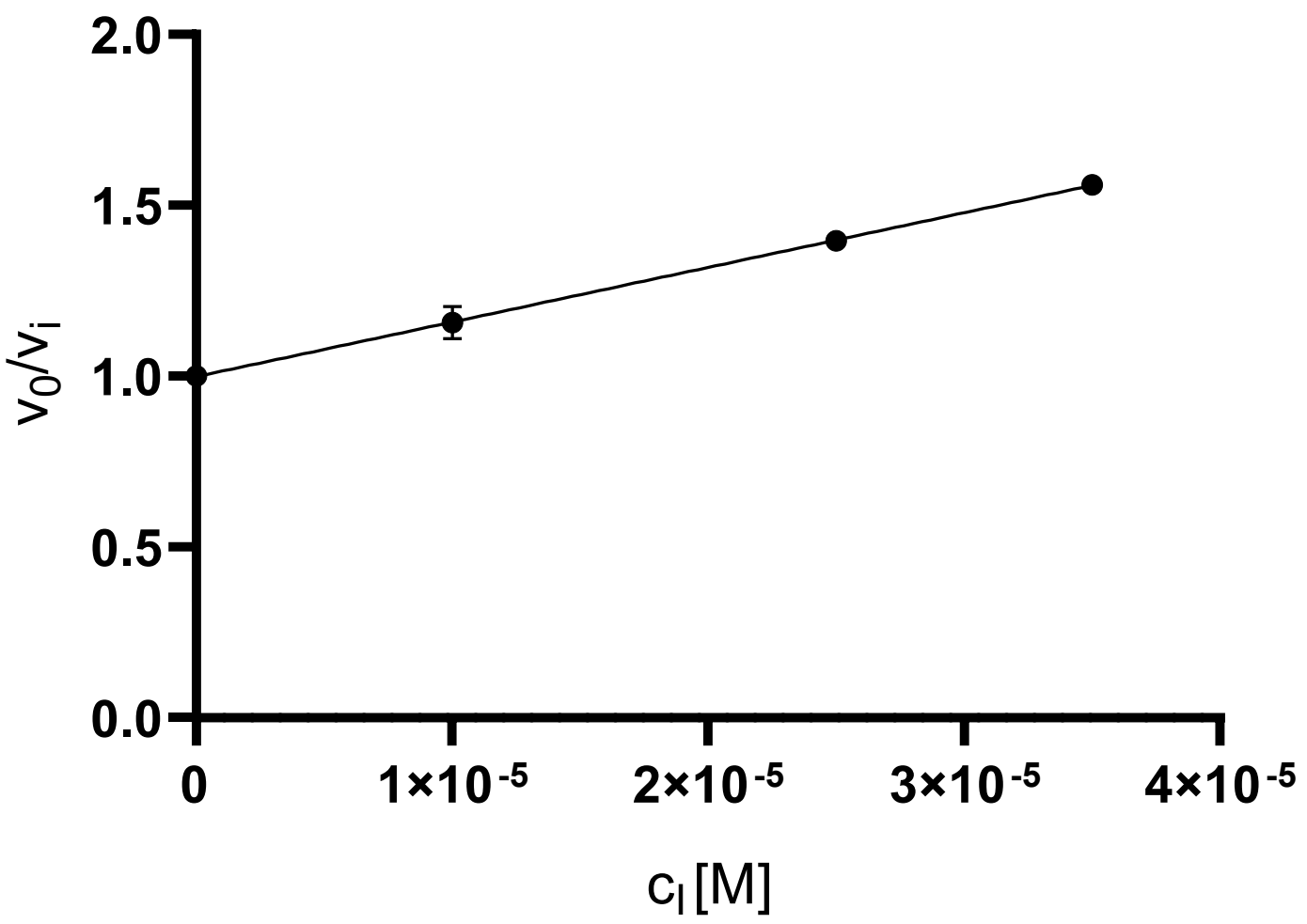

Figure **S55**. The dependence v_0/v_i vs. concentration of inhibitor **2n** inhibiting eeAChE.

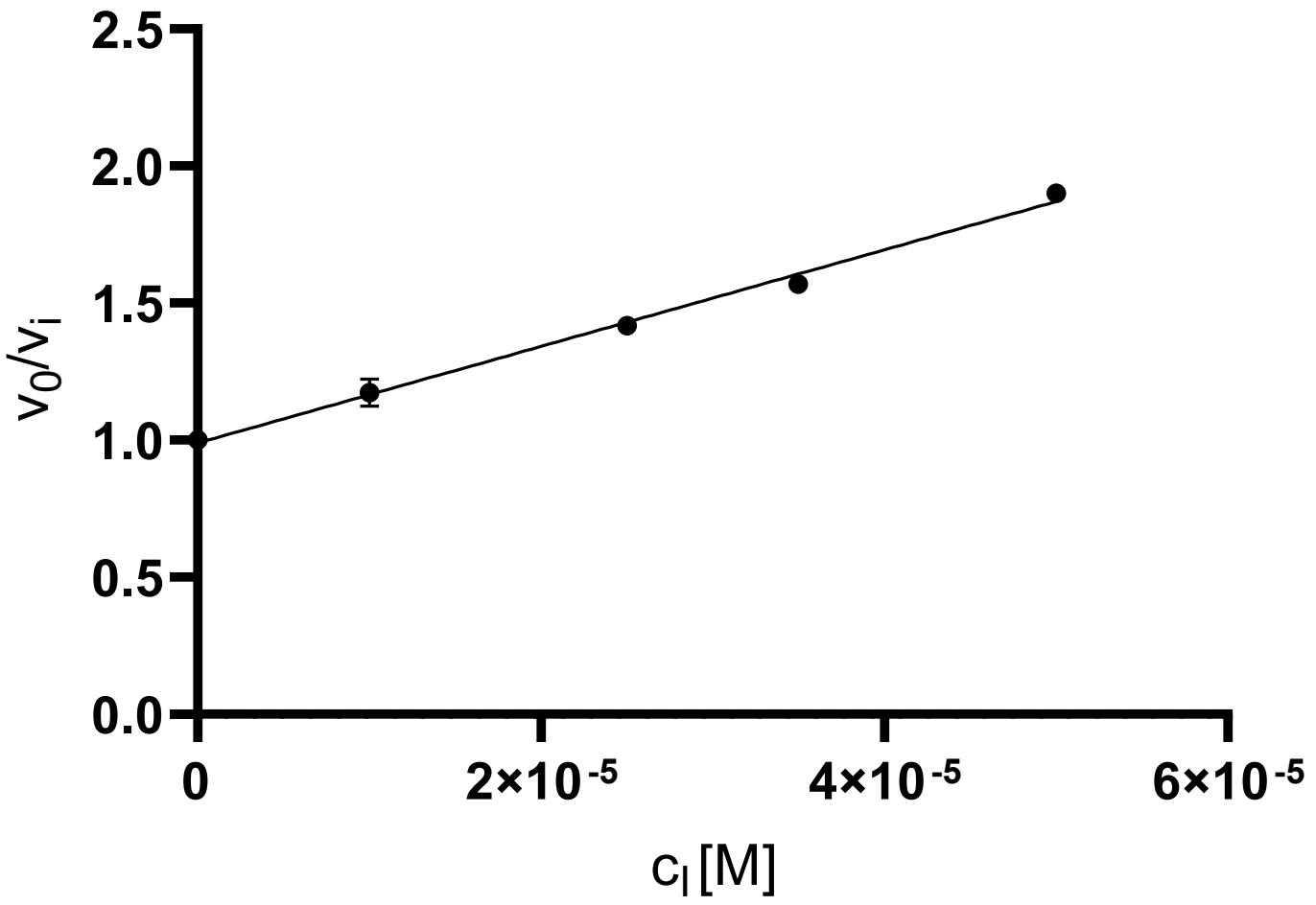

Figure **S56**. The dependence v_0/v_i vs. concentration of inhibitor **2n** inhibiting eqBChE.

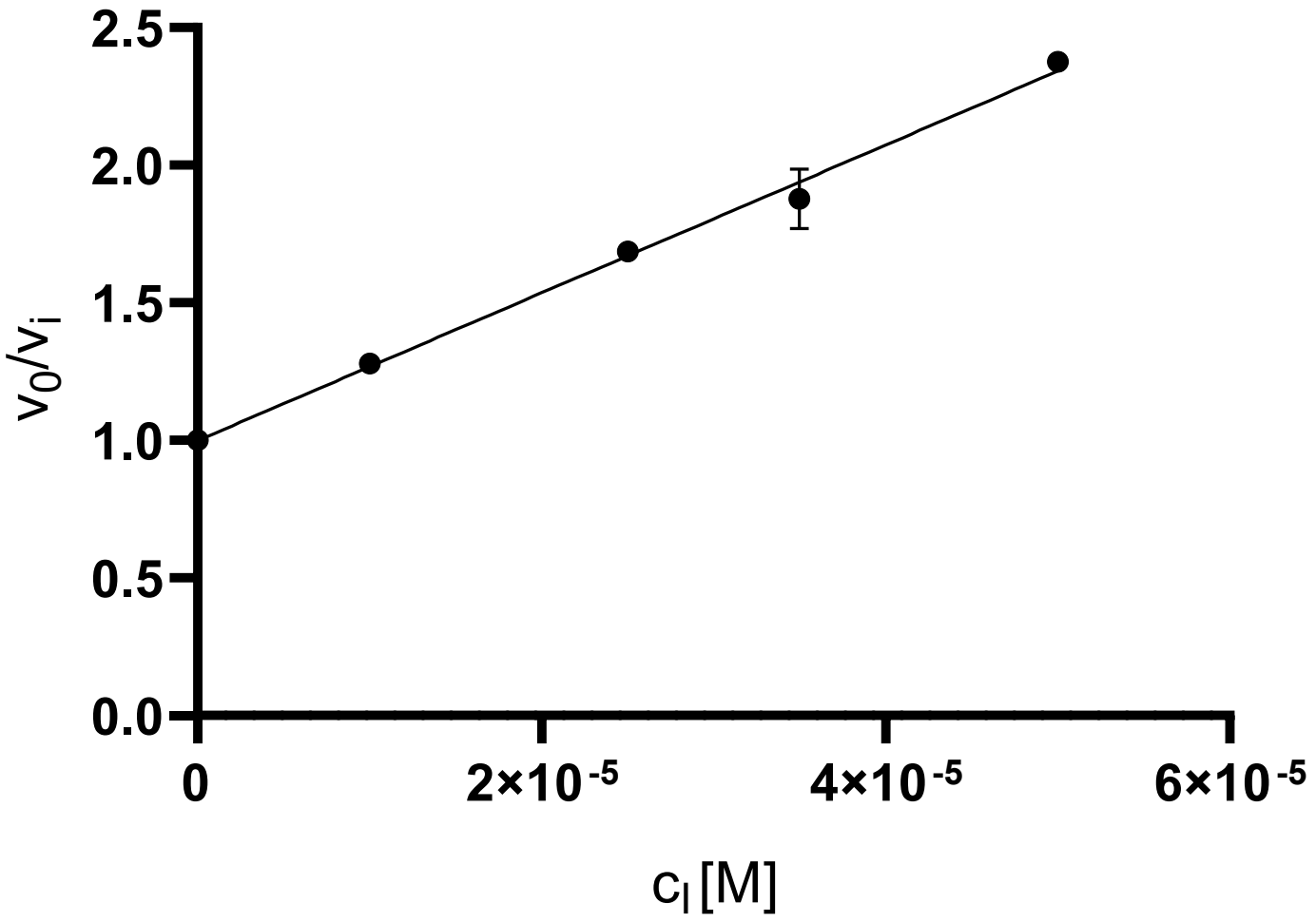

Figure **S57**. The dependence v_0/v_i vs. concentration of inhibitor **1o** inhibiting eeAChE.

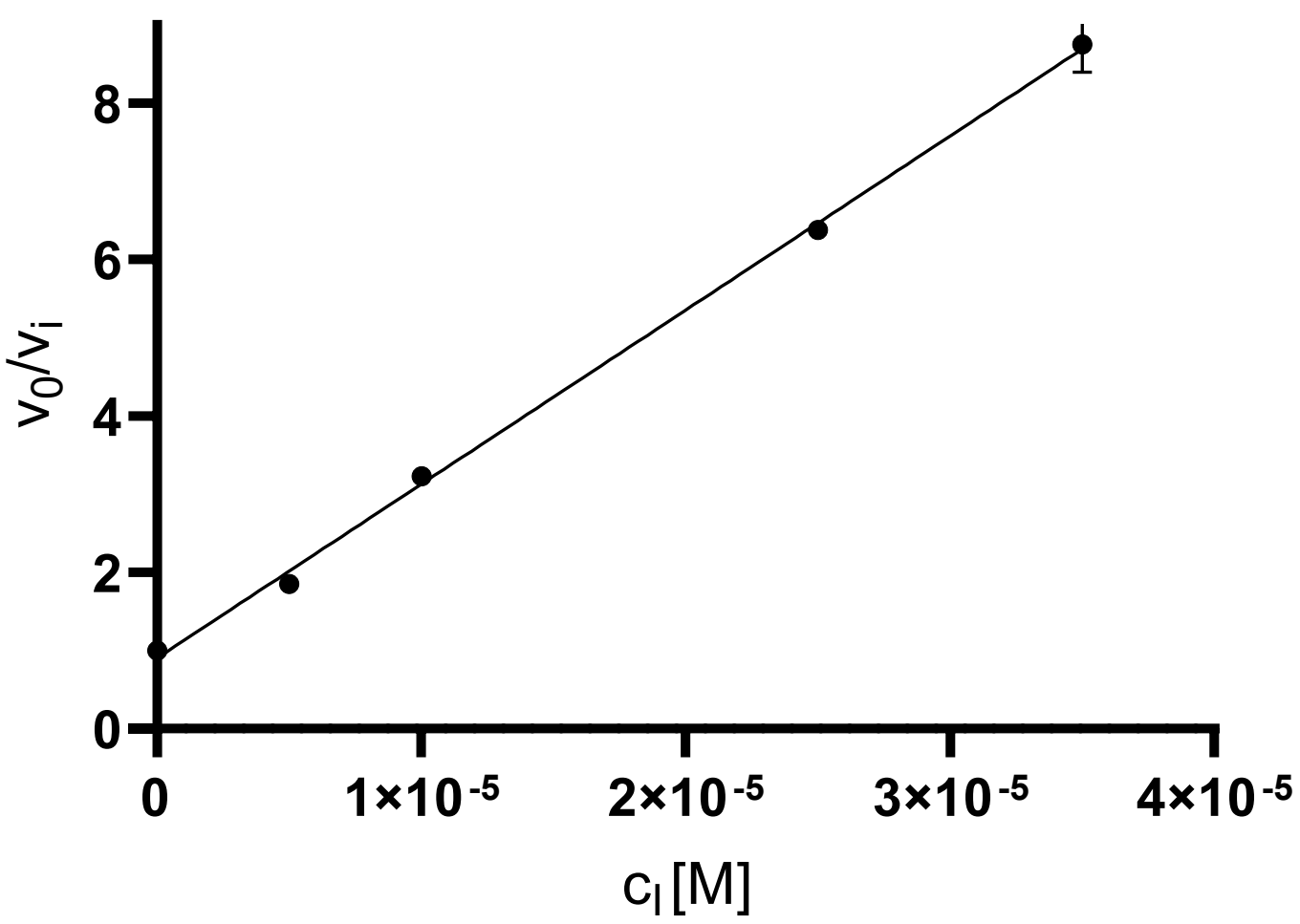

Figure **S58**. The dependence v_0/v_i vs. concentration of inhibitor **1o** inhibiting eqBChE.

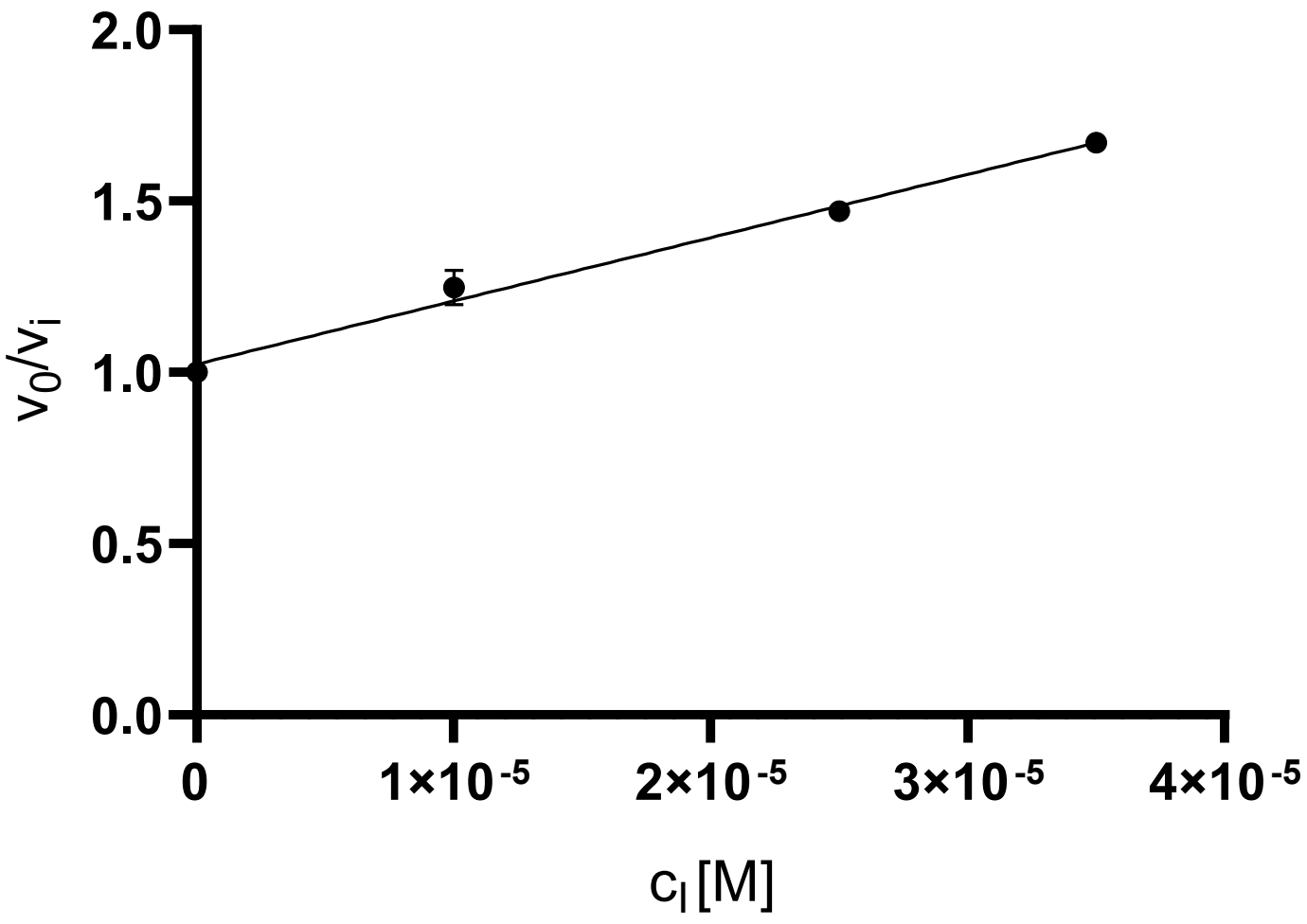

Figure **S59**. The dependence v_0/v_i vs. concentration of inhibitor **2o** inhibiting eeAChE.

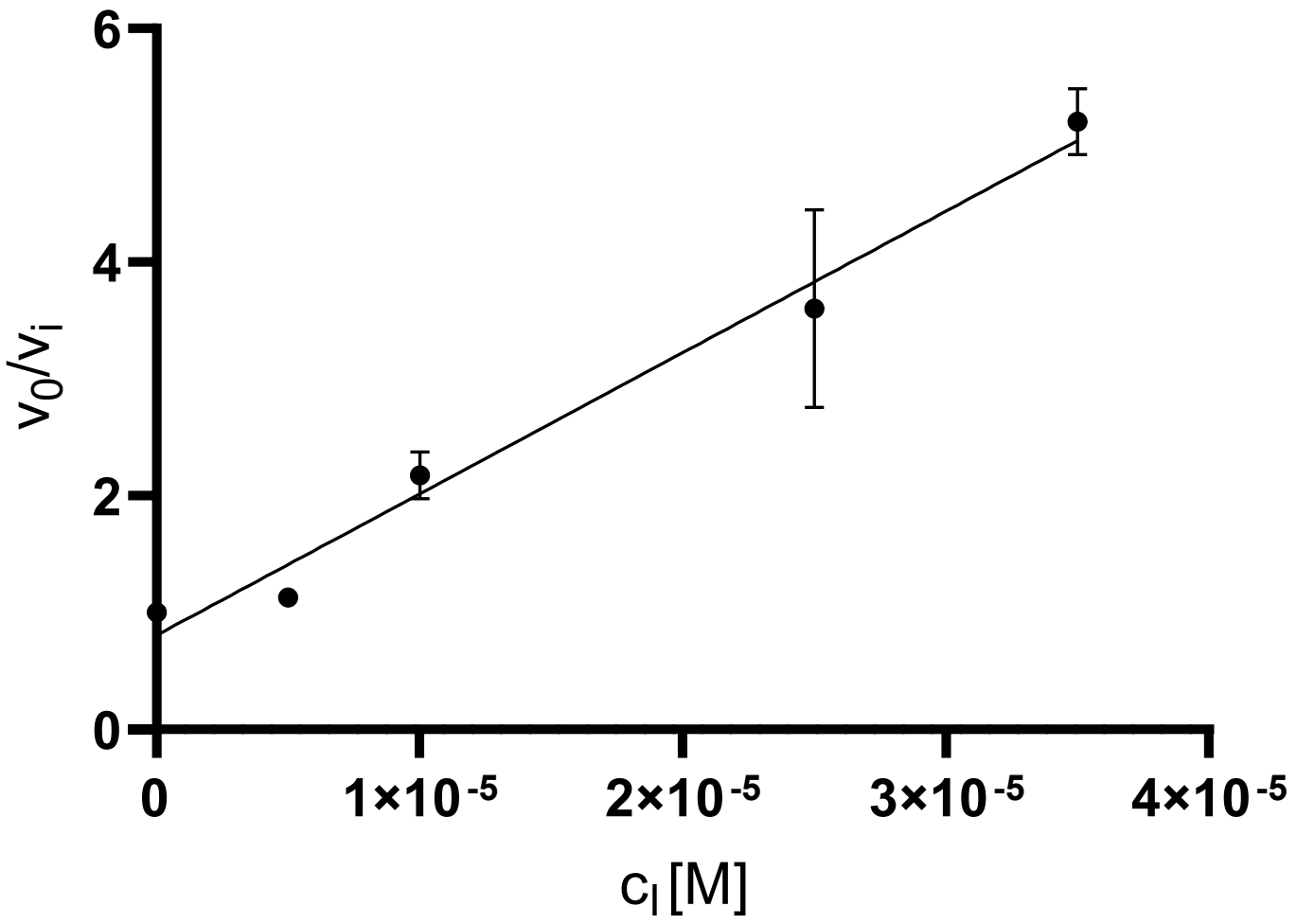

Figure **S60**. The dependence v_0/v_i vs. concentration of inhibitor **2o** inhibiting eqBChE.

Graph for **2g** inhibiting eqBChE is not provided (IC_{50} >500 μM).
